# Identification of restriction endonuclease with potential ability to cleave the HSV-2 genome: Inherent potential for biosynthetic versus live recombinant microbicides

**DOI:** 10.1186/1742-4682-5-18

**Published:** 2008-08-07

**Authors:** Misaki Wayengera, Henry Kajumbula, Wilson Byarugaba

**Affiliations:** 1 Restrizymes Biotherapeutics Uganda Limited, Kampala, Uganda; 2 Restrizymes Corporation- Toronto, Canada; 3 Division of Molecular Biology, Dept of Microbiology, College of Health Sciences, Makerere University, Upper Mulago Hill Road, P O Box 7072, Kampala, Uganda; 4 Division of Human and Molecular Genetics, Dept of Pathology College of Health Sciences Makerere University, Upper Mulago Hill Road, P O Box 7072, Kampala, Uganda

## Abstract

**Background:**

Herpes Simplex virus types 1 and 2 are enveloped viruses with a linear dsDNA genome of ~120–200 kb. Genital infection with HSV-2 has been denoted as a major risk factor for acquisition and transmission of HIV-1. Developing biomedical strategies for HSV-2 prevention is thus a central strategy in reducing global HIV-1 prevalence. This paper details the protocol for the isolation of restriction endunucleases (REases) with potent activity against the HSV-2 genome and models two biomedical interventions for preventing HSV-2.

**Methods and Results:**

Using the whole genome of HSV-2, 289 REases and the bioinformatics software Webcutter2; we searched for potential recognition sites by way of genome wide palindromics. REase application in HSV-2 biomedical therapy was modeled concomitantly. Of the 289 enzymes analyzed; 77(26.6%) had potential to cleave the HSV-2 genome in > 100 but < 400 sites; 69(23.9%) in > 400 but < 700 sites; and the 9(3.1%) enzymes: BmyI, Bsp1286I, Bst2UI, BstNI, BstOI, EcoRII, HgaI, MvaI, and SduI cleaved in more than 700 sites. But for the 4: PacI, PmeI, SmiI, SwaI that had no sign of activity on HSV-2 genomic DNA, all 130(45%) other enzymes cleaved < 100 times. In silico palindromics has a PPV of 99.5% for in situ REase activity (2) Two models detailing how the REase EcoRII may be applied in developing interventions against HSV-2 are presented: a nanoparticle for microbicide development and a "recombinant lactobacillus" expressing cell wall anchored receptor (truncated nectin-1) for HSV-2 plus EcoRII.

**Conclusion:**

Viral genome slicing by way of these bacterially- derived R-M enzymatic peptides may have therapeutic potential in HSV-2 infection; a cofactor for HIV-1 acquisition and transmission.

## Background

About 38.6 million people worldwide are now living with the Human Immunodeficiency Virus (HIV), which causes AIDS [1]. Heterosexual contact is the predominant mode of transmission of HIV infections worldwide. Women are at particularly increased risk of acquiring HIV through heterosexual contact. Despite this gender disparity, there are to date only limited options by which women may actively protect themselves against HIV. [2]. Recent studies have defined factors that are associated with increased susceptibility to HIV-1 [3,4]. Among these, genital infection with herpes simplex virus type 2 (HSV-2) is considered a major cofactor for both sexual transmission and acquisition of HIV-1 [5]. HSV-2 is a member of the genus of double-stranded DNA viruses called simplexvirus. HSV-2 together with its generic relative HSV-1 causes blistering lesions of the cervico-vaginal and oral mucosa, respectively. Fleming and Wasserheit recently provided biological, epidemiological and interventional evidence to support the view that infection with HSV-2 may significantly promote HIV transmission and acquisition [6]. Biologically, they show that HSV-2 does this by disrupting mucosal integrity [6], increasing the genital viral loads and numbers of activated immune cells that are susceptible to HIV-1 tropism [7,8]. Specifically, it increases the infectiousness of HIV-infected subjects through increased genital HIV load during a genital HSV-2 recurrence [7,8] by the transactivation of HIV-1 LTR through interaction with HSV proteins (ICPO, ICP4) or the production of pro-inflammatory chemokines known to enhance HIV-1 replication [9,10]. Similarly, HSV-2 may mediate the recruitment of activated CD4+ cells [11] that markedly up-regulate HIV replication in HSV-infected lesions [12]. It has recently been shown that HIV-1 interacts at the cellular level to form HIV-1 hybrid virions that are pseudotyped with HSV-1 envelope glycoproteins gD and gB, thus expanding HIV-1 cell tropism to include mucosal epithelial cells [13,14]. This has led to the hypothesis that HSV-2 may similarly interact with HIV-1 to form such "pseudotypes" with potential to infect other cells, although a recent study failed to provide evidence for such interaction [15].

In the light of the above evidence, developing biomedical strategies for the prevention of sexual transmission of HSV-2 has become recognized as a critical strategy in the control of sexual transmission of HIV-1 [16]. We recently described pre-integration viral genome slicing [PRINT_GSX] as a novel model for devising antiviral gene-based therapies using a retrovirus replication model (HIV cDNA) [17]. This approach explores the natural antiviral defense model inherent in bacteria through a nucleic-acid enzymatic system called the restriction modification (R-M) system [18]. Bacteria endowed with R-M systems have been shown to be remarkably resistant to tropism by bacteriophages. Four taxonomic classes of R-M systems are recognized to day, with type II being the most widespread [18]. Type II R-M systems comprise two distinct peptides functioning respectively as restriction endonuclease (REase) and cognate methyltransferase (MTase). As a model illustration of function, class I RMS systems, the evolutionary ancestors of R-M systems, are employed here. The class I RMS of Escherichia *coli strain *K-12 comprises 6 enzymes, of which the respective genes are located on the bacterial chromosome in a region called an immigration island: the hsdS gene, hsdR gene, hsdM gene, mcrB/C genes and mrr gene. Products of the first two genes play the central antiviral defense function (by recognizing and splicing the exogenous DNA through recognizing 4–12 base pair palindromes; that is nucleotide sequences that read the same in both directions). The site specific subunit hsdS product serves to recognize the specific 4–12 " palindromic" base pair sequence in the genome of the invading phage, while the hsdR restriction subunit product cleaves the DNA if this site is unmethylated. The other 4 gene products serve to protect the host genome as follows: the hsdM gene product is a methyltransferase that transfers a methyl group from S-adenosylmethionine (SAM) to the DNA at the indicated A residues; the mcrBC system restricts DNA containing methylcytosine residues; while the mrr system restricts DNA with m6-methyladenine or m6-methylcytosine [19,20].

The aim of this study is to extend our previous work on viral genome slicing (GSX) to HSV-2 by identifying REases (DNases) with potent ability to cleave the HSV-2 genome. Although the replicative cycles of some eukaryotic viruses such as HSV-2 do not necessary involve viral genome integration into the host nuclear DNA as occurs for retroviruses, we propose that these REases are equally worth exploring for the development of novel HSV-2 microbicides. Two models are proposed for using the REase EcoRII to target HSV-2: first, by cross-linking the enzyme through the formation of C31G (Savvy) and *EcoRII *PLGA-loaded nanoparticles (**nano-C31G-EcoRII**); second, by expressing EcoRII in Lactobacillus that also expresses a truncated recombinant form of the receptor nectin-1 (**xREPLAB-tN1**). The former are nanoparticles that may be explored to develop a model combinational microbicide, while the latter is a model "live" microbicide strategy for diverting and disrupting infectious HSV-2 particles.

## Results

### A. HSV-2 genome-wide in silico palindromics: REases with HSV-2 genome cleaving potential

Of the 289 enzymes from the REBASE database analyzed; 77 (26.6%) demonstrated potential to cleave the HSV-genome in > 100 but < 400 sites (see Table 1 for details) and 69 (23.9%) enzymes cleaved in > 400 but < 700 sites (see Table 2). Nine (3.1%) enzymes had more than 700 potential cleavage sites: BmyI, Bsp1286I, Bst2UI, BstNI, BstOI, EcoRII, HgaI, MvaI, and SduI, all of which are Type II restriction enzyme subtype P, derived respectively from the bacteria *Bacillus mycoides *[21], *Bacillus sphaericus *[22], *Bacillus stearothermophilus *2U, *Bacillus stearothermophilus *[23], *Bacillus stearothermophilus *O22, *Escherichia coli *R245 [24], *Haemophilus gallinarum *[25]* Micrococcus varians *RFL19 [26] and *Streptococcus durans *RFL3 [27] (see table 3). However, for the 4 that had no sign of activity on HSV-2 genomic DNA (PacI, PmeI, SmiI, SwaI – [for details see Additional file 1]), all 130 (45%) other enzymes cleaved < 100 times. We have previously demonstrated that in silico palindromics, a novel downstream science of genomics for analysis of restriction enzyme activity using Webcutter software version 2, has a PPV of 99.5% for in situ REase activity [18].

**Table 1:** REase (DNase) enzymes cutting HSV-2 genome in > 100, but < 400 sites

Enzyme name	Genomic splices (palindrome)
AccBSI	164(gagcgg)
AccI	111(gt/mkac)
AclWI	225(ggatc)
AflIII	127(a/crygt)
Alw21I	203(gwgcw/c)
Alw26I	308 (gtctc)
AlwI	225 (ggatc)
ApaI	267 (gggcc/c)
AspHI	203 (gwgcw/c)
BbeI	261 (ggcgc/c)
Bbv12I	203 (gwgcw/c)
BsaWI	131 (w/ccggw)
Bse1I	155 (actgg)
BseNI	155 (actgg)
BsePI	349 (g/cgcgc)
BseRI	213 (gaggag)
BsiHKAI	203 (gwgcw/c)
BsiI	111 (ctcgtg)
BsmAI	308 (gtctc)
BsmBI	149 (cgtctc)
Bsp120I	267 (g/ggccc)
BspMI	123 (acctgc)
BpmI	170 (ctggag)
BsaAI	155 (yac/gtr)
BsrBI	164 (gagcgg)
BsrI	155 (actgg
BsrSI	155 (actgg)
BssHII	349 (g/cgcgc)
BssSI	111 (ctcgtg)
BstZI	338 (c/ggccg)
BssT1I	124 (c/cwwgg)
BstD102I	164 (gagcgg)
BstDEI	139 (c/tnag)
BstF5I	292 (ggatg)
BstX2I	108 (r/gatcy)
BstYI	108 (r/gatcy)
Cfr9I	286 (c/ccggg)
DdeI	139 (c/tnag)
EagI	338 (c/ggccg)
EclXI	338 (c/ggccg)
Eco130I	124(c/cwwgg)
EcoT14I	124 (c/cwwgg)
EheI	261 (ggc/gcc)
ErhI	124 (c/cwwgg)
Esp3I	149 (cgtctc)
FokI	292 (ggatg)
HincII	105 (gty/rac)
HindII	105 (gty/rac)
HinfI	318 (g/antc)
HphI	280 (ggtga)
KasI	261 (g/gcgcc)
MaeIII	244 (/gtnac)
MboII	261 (gaaga)
MflI	108 (r/gatcy)
MroNI	250 (g/ccggc)
MseI	116 (t/taa)
MslI	124(caynn/nnrtg)
NaeI	250 (gcc/ggc)
NarI	261 (gg/cgcc)
NgoAIV	250 (g/ccggc)
NgoMI	250 (g/ccggc)
NspI	104 (rcatg/y)
PleI	212 (gagtc)
PpuMI	169 (rg/gwccy)
Psp5II	169 (rg/gwccy)
PspAI	286 (c/ccggg)
PspALI	286 (ccc/ggg)
PspOMI	267 (g/ggccc)
SfaNI	279 (gcatc)
SmaI	286 (ccc/ggg)
TfiI	106 (g/awtc)
Tru1I	116 (t/taa)
Tru9I	116 (t/taa)
Tsp45I	184 (/gtsac)
TspRI	109 (cagtg)
XhoII	108 (r/gatcy)
XmaI	286 (c/ccggg)
XmaIII	338 (c/ggccg)

77 total enzymes

**Table 2:** REase (DNase) enzymesHSV-2 genome cutting in > 400 but less 700 sites

Enzyme name	Genomic splices (palindrome)
AccB1I	403 (g/gyrcc)
AcyI	671(gr/cgyc)
AfaI	426 (gt/ac)
AluI	456 (ag/ct)
Ama87I	613 (c/ycgrg)
AvaI	613 (c/ycgrg)
AvaII	613 (g/gwcc)
BanI	403 (g/gyrcc)
BanII	520 (grgcy/c)
BbiII	671 (gr/cgyc)
BbvI	613 (gcagc)
BcoI	613 (c/ycgrg)
BglI	316 (gccnnnn/nggc)
Bme18I	613 (g/gwcc)
BsaHI	671 (gr/cgyc)
BsaOI	634 (cgry/cg)
Bse118I	428 (r/ccggy)
Bsh1285I	634 (cgry/cg)
BshNI	403 (g/gyrcc)
BsiEI	634 (cgry/cg)
BsmFI	668 (gggac)
BsoBI	613 (c/ycgrg)
Bsp143I	449 (/gatc)
Bsp143II	562 (rgcgc/y)
BsrFI	428 (r/ccggy)
BssAI	428 (r/ccggy)
Bst71I	613 (gcagc)
BstDSI	699 (c/crygg)
BstH2I	562 (rgcgc/y)
BstMCI	634 (cgry/cg)
Cfr10I	428 (r/ccggy)
Cfr42I	400 (ccgc/gg)
CfrI	698 (y/ggccr)
Csp6I	426 (g/tac)
DpnI	449 (ga/tc)
DpnII	449 (/gatc)
DraII	450 (rg/gnccy)
DsaI	699 (c/crygg)
EaeI	698 (y/ggccr)
Eco24I	520 (grgcy/c)
Eco47I	613 (g/gwcc)
Eco52I	338 (c/ggccg)
Eco64I	403 (g/gyrcc)
Eco88I	613 (c/ycgrg)
EcoO109I	450 (rg/gnccy)
FriOI	520 (grgcy/c)
GsuI	170 (ctggag)
HaeII	562 (rgcgc/y)
HgiEI	613 (g/gwcc)
Hin1I	671 (gr/cgyc)
Hsp92I	671 (gr/cgyc)
Hsp92II	434 (catg/)
KspI	400 (ccgc/gg)
Kzo9I	449 (/gatc)
MaeII	581 (a/cgt)
MboI	449 (/gatc)
Msp17I	671 (gr/cgyc)
MspA1I	633 (cmg/ckg)
NdeII	449 (/gatc)
NlaIII	434 (3168 catg/)
NspBII	633 (cmg/ckg)
RsaI	426 (gt/ac)
SacII	400 (ccgc/gg)
Sau3AI	449 (/gatc)
Sfr303I	400 (ccgc/gg)
SinI	613 (g/gwcc)
SstII	400 (ccgc/gg)
TaqI	503 (t/cga)
TthHB8I	503 (t/cga)

69 total enzymes

**Table 3:** REase enzymes cutting HSV-2 genome in 700 or more times

Enzyme name	Genomic splices (palindrome)
^1^BmyI*	773 (gdgch/c)
^2^Bsp1286I*^+#^	773 (gdgch/c)
^3^Bst2UI*^+^	824 (cc/wgg)
^4^BstNI*^+#^	824 (cc/wgg)
^5^BstOI*^+^	824 (cc/wgg)
^6^EcoRII*^+#^	824 (/ccwgg)
^7^HgaI*^+#^	831 (gacgc)
^8^MvaI*^+#^	824 (cc/wgg)
^9^SduI*^+#^	773 (gdgch/c)

*Type II restriction enzyme subtype: P; ^+^commercially available; ^#^Enzyme gene cloned ^1–9 ^Source of REase: Bacillus mycoides [21], Bacillus sphaericus [22], Bacillus stearothermophilus 2U, Bacillus stearothermophilus [23], Bacillus stearothermophilus O22, Escherichia coli R245 [24], Haemophilus gallinarum [25] Micrococcus varians RFL19 [26] and Streptococcus durans RFL3[27]

### B. Modeling nano-N-9-EcoRII; a nanoparticle that may be explored to develop microbicides against HSV-2

**Figure 1:**
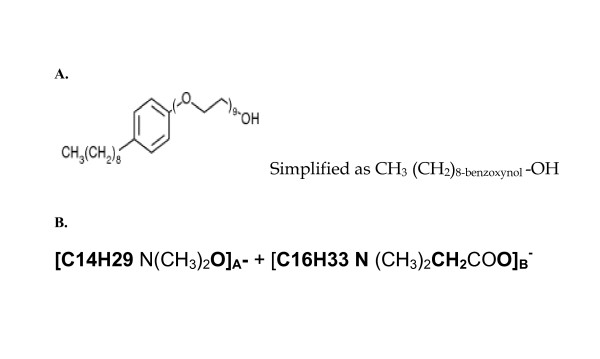
**This figure shows the chemical structures of nonoxynol-9 and C31G**. **A**. Note the hydrophilic end with the hydroxyl ion at the extreme left; and the hydrophobic hydrocarbon-benzene complex. This property confers on this molecule the ability to complex with both hydrophilic (ionized) and hydrophobic molecules. The chemical formula and molecular mass of a single nonoxynol-9 molecule are respectively C_33_H_60_O_10 _and 616.823 g/mol. **B**. C31G is a 1:1 mixed Micelle of Alkyl dimethyl amine oxide and Alkyl dimethylglycine (betaine).

A model of a nanoparticle that may be explored in microbicide development was conceptualized. We based that conception on the hypothesis that "for viral genome to be rendered susceptible to a REase with potent activity against the HSV-2 genome, the naked HSV-2 genome must be brought into proximity with the REase". For purposes of this modeling, we have theoretically employed chemical two surfactants, Nonoxynol-9 and Savvy (C31G); although several other synthetic detergents with demonstrated safe profiles following repeated application in vaginal mucosa of both humans and animals such as 1.0% Savvy (C31G) [28]; and plant derivative like Praneem polyherbal suppository and gossypol may serve the purpose. Note that meta-analysis of randomized controlled trials including more than 5000 women for N-9 safety have indicated some evidence of harm through genital lesions; with N-9 not being recommended for HIV and STI prevention[29]; while no serious adverse event was attributable to SAVVY(C31G) use by a Phase 3, double-blind, randomized, placebo-controlled trial [30]. To this regard, for purposes of in-vivo viral envelope-disruption, Savvy and other surfactants with safe profiles in humans may be a better and safer option. The chemical structure and molecular weight of both N-9 and Savvy are shown in Figure 1.

**Figure 2:**
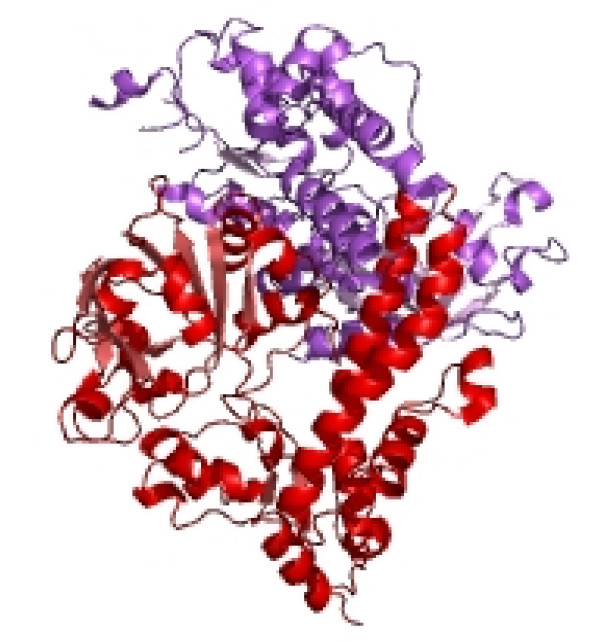
**This figure shows the deposited crystal structure of restriction endonuclease EcoRII mutant R88A in the European Molecular Biology Laboratory (EMBL) Protein database (entry 1nas6)**. A detailed structure of the N-domain, which contains the effector-binding cleft of EcoRII with putative DNA-binding residues H36, Y41, K92, R94, E96, K97 and R98, can be found from work by Zhou et al. [34].

We obtained the chemical formula and molecular weights of the enzyme EcoRII by using its complete gene and protein sequences [31-33]. Protparam software (Expasy, Swissprot) tool was used for this modeling, as described elsewhere [34]. For details of results of the physicochemical parameters of EcoRII, see Table 4 and [see Additional file 2]. From these results, specifically the values of the anionic and cationic amino acid composition, it may be noticed that EcoRII is overall negatively charged (-52, +43; overall molecule charge is -9), providing anions that could bind free H^+ ^in the lactic acid of "PLGA". The other measured EcoRII variables included number of atoms, amino acid composition, instability index, aliphatic index, theoretical PI, in vivo half life and grand average hydropathy (GRAVY) and are shown in Table 4. The 3-dimensional structure of EcoRII was modeled from that previously reported [35]; and is available as PDB entry 1nas6 in the EMBL protein database (see Figure 2).

**Table 4:** Physiochemical parameters of EcoRII as predicted from the amino acid sequence alignments

Physicochemical parameter	Value
**Number of amino acids:**	**404**
**Molecular Weight**	**45611**
**Theoretical PI**	**6.10**
**Total number of negatively charged residues (Asp+Glu)**	**52**
**Total number of positive residues (Arg+Lys)**	**43**
**Atomic composition:**	
• Carbon(C)	2053
• Hydrogen(H)	3205
• Nitroge(N)	565
• Oxygen(O)	393
• Sulfur	10
**Total number of atoms:**	**6426**
	
**Formula:**	**C**_2053_**H**_3205_**N**_565_**O**_593_**S**_10_
	
**Extinction coefficients:**	**47120(46870)**
	
**Estimated half-life(hours)**	
• (mammalian reticulocytes, in vitro)	30 hour
• (yeast, in vivo)	> 20 hours
• (Escherichia coli, in vivo)	> 10 hours
	
**Instability index:**	**45.04**
	
**Aliphatic index:**	**97.05**
	
**Grand average of hydropathicity (GRAVY)**	**-0.183**

**Figure 3:**
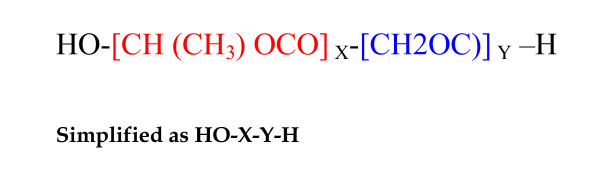
**The figure shows a simplified chemical structure of PGLA**. X represents lactic acid while y represents glycolic acid. Notice the availability of the hydroxyl (-OH) and free hydrogen (+H) ions at lactic and glycolic extremities of the PLGA molecule respectively. This possibly accounts for diversity of PLGA solvent solubility. PLGA may thus effectively be used to complex both EcoRII and nonoxynol-9 by a two step emulsion of EcoRII first in PLGA; followed by a final emersion in nonoxynol-9.

**Figure 4:**
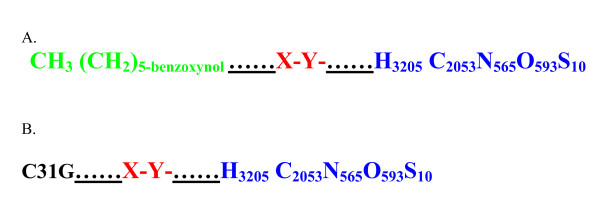
**This figure attempts to model the molecular binding of EcoRII to nonoxynol-9 or C31G (savvy) through the polyester PLGA**. **A**. N-9 and EcoRII PLGA loaded nanoparticles: Note the orientation of the hydrogen and hydroxyl ions in the glycolic and lactic acids monomers of PLGA towards the hydroxyl and hydrogen ions in the N-9 and the REase nanoparticles model. The underlined dots signify that it is unknown which, covalent or hydrogen, bonds are involved. **B**. C31G and EcoRII PLGA loaded nanoparticles. Note that the chemical structure of Savvy is has been abbreviated to C31G, but is [C14H29 N(CH_3_)_2_O]_A_^- ^+ [C16H33 N (CH_3_)_2_CH_2_COO]_B_^-^.

For the purposes of achieving conjugation and chemical binding between either Savvy or Nonoxynol-9) and EcoRII, we further hypothesized that the aliphatic polyester poly(lactic-*co*-glycolic acid) (PLGA) may suffice [35]. PLGA is a copolymer that is synthesized by random ring-opening co-polymerization of two different monomers, the cyclic dimers (1,4-dioxane-2,5-diones) of glycolic acid and lactic acid on either tin (II) 2-ethylhexanoate, tin(II) alkoxides, or aluminum isopropoxide as catalysts. Owing to its wide solubility, bio-degradability and compatibility, PLGA is used in drug delivery by the formation of nanoparticles [36]. A simplified chemical structure of PLGA is shown in Figure 3. We finally derived a likely chemical structure of a single molecule of the nanoparticles: 1) **nano-N-9-EcoRII** and **Nano-C31G-EcoRII**. Both Theses model nanoparticle structures are shown in Figure 4. We believe that such nanoparticles may be synthesized practically using a two-step emulsion of EcoRII in PLGA followed by addition of N-9 or C31G rather than polyacrylic acid (PAA) as described elsewhere [35]. Note that it has been assumed that only a single molecule of EcoRII, C31G or N-9 and PLGA will form the nanoparticle, although practically speaking, the relative proportions of the constituent molecules may vary.

### C. Modeling a "recombinant lactobacillus" able to attract and destroy HSV-2

Additionally, we propose that a recombinant Lactobacillus expressing "truncated nectin-1 and EcoRII" may achieve a "divert and destroy" strategy against HSV-2. That strategy is based on two hypotheses.

First, surface anchoring of the HSV-2 cellular receptor on the cell walls of native vaginal bacteria (and not merely secretory expression) is possible, and may realise a "divert" strategy for HSV-2 genital infection. This hypothesis is based on the following observations and conceptualizations: (i) Lactobacilli exist as a biofilm that acts as a first line of defence over the genital mucosa. This biofilm forms a potential antimicrobial barrier over the epithelia lining. (ii) Enhancing the antiviral properties of *Lactobacilli *has recently become a strategy for protecting underlying susceptible mucosal cells from viral tropism [36-40]. Specifically, we believe that making these cells mimic "susceptible cells" may divert primary HSV-2 infection. Liu et al. [38] have recently engineered Human vaginal Lactobacilli for surface expression of two domain CD 4 using native sequences of a defined length upstream of the unique C-terminal LPQTG cell wall sorting signal and the positively charged C-terminus in a *Lactobacillus*-based expression system. The modified *L. jensenii *displayed 2D CD4 molecules that were uniformly distributed on the bacterial surfaces, and recognized by a conformation dependent anti-CD4 antibody, suggesting that the expressed proteins adopted a native conformation. Such *Lactobacillus*-based surface expression systems, with potential broad applicability, represent a major step toward developing an inexpensive, yet durable approach to topical microbicides for mitigation of heterosexual transmission of HIV and other mucosally transmitted viral pathogens [38]. Heterologous proteins have been expressed on the surfaces of other Gram-positive bacteria via the sortase23-catalyzed cell wall anchoring mechanism [41], including 5 *Streptococcus gordonii, Lactobacillus paracasei *and *Staphylococcus carnosus *[41-45]. Assuming that this approach can be used to anchor the HSV-2 surface receptor on their cell walls, these bacteria may "mimic" susceptible underlying cells and become infected with HSV-2. This is what we refer to as the "divert strategy". Although HSV-2 attachment and entry into epithelial cells is mediated through a chain of events, a member of the immunoglobulin (Ig) superfamily closely related to the poliovirus receptor (Pvr), PRR1 (also known as HveC, CD111, CLPED1, ED4, HIgR, HVEC, MGC142031, MGC16207, OFC7, PRR, PRR1, PVRR, PVRR1, SK-12, nectin-1), has been found to be the most effective mediator of HSV-2 attachment and viral entry. HveC also mediates the entry of other *alphaherpesviruses *[46-52]. Krummenacher et al. [52] have cloned and expressed a "truncated" form of HveC (HveCt) in non-permissive insect cell lines (*Spodoptera frugiperda *or Sf9) using plasmid pCK285 [46,52] to purify soluble proteins. Given that both CD4 and HveCt are members of the immunoglobulin (Ig) superfamily, we predict that cell wall anchored truncated nectin-1 (HveCt) can be expressed in *Lactobacillus *using a modified form of plasmid pCK285 and the approach recently devised by Liu et al. [38]. Such additional modifications are necessary because the promoter previously used (polyhedrin) to express HveCt in insect cells is specific for baculovirus [46,52]; a construct using a bacterial promoter active in *Lactobacillus *is needed. For instance, the P23 promoter from *Lactococcus lactis *created by PCR amplification with the primers 5'-GTGGAGCTCCCCGAAAAGCCCTGACAACCC-3' and 5'-GGAAACACGCTAGCACTAACTTCATT-3', as described by Liu et al., may suffice [38].

Second, we have hypothesized that by further modifying these truncated nectin-1(or HveC)-expressing lactobacilli to express restriction enzymes with potent genome slicing potential such as the EcoRII shown here, integration of the HSV-2 genome into them can be halted (through the disruption or destruction of its genome). This further modification would allow for a "divert and destroy" strategy similar to that being explored in HIV [38-40]. It is likely that EcoRII can be expressed in *Lactobacilli *because a previous genome-wide analysis of the Lac. *Plantinuum *protein database revealed the presence of Mtase and REase activities derived from *Staphylococcus aureus *[37]. Plasmid-mediated transfer of R-M activity is common in bacteria [19,20], and because EcoRII is originally encoded on a plasmid rather than the *E. coli *chromosome [24], recombinant transfer of plasmid R245 to Lactobacilli is likely achievable. The additional "destroy" conception is suggested by the approach that bacteria use to resist tropism bacteriophages [17,18]. The resultant model recombinant *Lactobacillus *has been dubbed "**xREPLAB-tN1".**

## Discussion

This work extends the concept of viral genome slicing (GSX), previously described for human retroviruses as a module for research and development of novel antivirals at the genome level [17], to HSV-2. Because HSV-2 has been noted as a major cofactor in the sexual acquisition and transmission of HIV-1 [5-15], preventing HSV-2 infection in this way may be a potential strategy for reducing the sexual transmission and acquisition of HIV-1.

Here, we detail the first focused effort to identify REases with potential splicing activity against the HSV-2 genome (more than 700 sites) – BmyI, Bsp1286I, Bst2UI, BstNI, BstOI, EcoRII, HgaI, MvaI and SduI – which may be applied to research and the development of HSV-2 biomedical prevention strategies. All 9 of these REase are Type II restriction enzyme subtype P, derived respectively from the bacteria *Bacillus mycoides *[21], *Bacillus sphaericus *[22], *Bacillus stearothermophilus *2U, *Bacillus stearothermophilus *[23], *Bacillus stearothermophilus *O22, *Escherichia coli *R245 [24], *Haemophilus gallinarum *[25]*Micrococcus varians *RFL19 [26] and *Streptococcus durans *RFL3 [27] (see Table 3; details of other cutting enzymes and frequency of splices are shown in Tables 1, 2 and [Additional file 1]). However, it should be noted that some of these enzymes are isoschizomers that are not significantly active under human physiological conditions. For instance, the three REases derived from *Bacillus stearothermophilus *have optimal activity at 60°C [21-23]. Such characteristics make them impractical for use in the design of microbicides. Therefore, not all these suggested restriction enzymes may actually be successfully applied in both approaches modeled. The enzyme EcoRII was selected because: (1) it is metabolically stable at temperature ranges inclusive of normal human body temperature(see Table 4 and Additional file 2) [24]; (2) its source, the bacterium *Escherichia coli*, is similarly a Gram positive bacteria of which the cell wall anchoring system can be modified to express heterologous proteins as in *Lactobacillus *strains; (3) it exhibits one of the highest slicing potentials against the HSV-2 genome (a strategy that may be beneficial in avoiding spontaneous ligation-see tables 1, 2 and 3); (4) The REase is encoded on plasmids rather than the bacterial chromosome, making its transfer to other bacterial strains possible.

Several questions remain to be answered about the two proposed models. However, many of them can be addressed fully through in situ experimentation rather than modeling approaches. In both proposed models, it is possible to question whether the additional modifications – (i) cross linking EcoRII to N-9 or C31G (ii) expressing EcoRII in HveCt-expressing *Lactobacilli *– are relevant. For instance, while it is reasonable to propose that the EcoRII and N-9 or C31G PLGA-loaded nanoparticles may disrupt the viral envelope and possibly the viral capsid, bringing the naked genome into contact with the REase, one could nevertheless argue that the virus is no longer infectious by the time the genome is released from the virion, which would make the REase redundant. A similar argument could be made for the *Lactobacillus *approach. Once the virus has infected *Lactobacillus*, it cannot infect the vaginal epithelium, so destruction of the genome by REase appears unnecessary. Moreover, the N-9 comprised nanoparticles are used here for theoretical purposes, as their use in humans is bound to raise safety concerns emanating from the previous evidence of mucosal irritation and enhancement of both HIV and STI transmission [28]. Never the less, in the absence of experimental evidence based on such nanoparticles, one could still argue their case from the fact that chemotherapeutic agents with noted in-vivo toxicity have been observed to exhibit extensively reduced such adverse effects when complexed into nanoparticles. For instance, DiJoseph *et al *have recently shown that conjugation of calicheamicin to rituximab with an acid-labile or acid stable linker vastly enhances its growth inhibitory activity against BCL in vitro, has no deleterious effect on the effector functional activity of rituximab, and exhibited greater anti-tumor activity against B cell lymphoma(BCL) xenografts and improved survival of mice with disseminated BCL over that of unconjugated rituximab. Such demonstrated reduced adverse effects of a calicheamicin immunoconjugate of rituximab demonstrate the safety advantage nanoparticles confer to initially unsafe bioactive agents [53].

In the case of the proposed nanoparticle model, it is not fully known by which bonds the REase will combine with the polymer (whether convalent or hydrogen bonds, as shown in Figure 4). Such bonds would presumably influence or affect the pattern of release of the components (covalent bonds are stronger and harder to break than hydrogen bonds). Moreover, the chemical models of "N-9 or C13G and EcoRII" PLGA-loaded nanoparticles shown in Figure 4 propose a single nonoxynol-9 or C31G molecule per REase. However, that may not be the case in the resultant nanoparticles (in situ evaluation of the composition of the nanoparticles is required). In addition, whether the molar concentrations of the respective active ingredients (N-9 or C31G and EcoRII) are sufficient to destabilize the viral envelope and genome, respectively, can only be decided by in situ experiments. Because of its previously demonstrated unsafe profiles in humans [29], any attempts to employ N-9 in such nanoparticles strategies are likely to exploit much lesser concentrations so as to achieve safety. In so doing, that may compromise efficacy for viral envelope disruption. Further still, it is not known whether such polymerization may affect enzyme or surfactant activity. Enzyme activities depend on active site conformations, and any changes in the 3D structure will probably influence activity. We have assumed that, since REases are stored in the simple ester construct glycerol, and PLGA is in essence a poly-ester, EcoRII may remain active despite copolymerization. Also, in the proposed nanoparticle model, the involvement of the hydrophilic hydroxyl group of N-9 or C31G or any other detergents in the interaction with PLGA could possibly affect the amphiphatic properties required to disrupt the viral envelope and capsid.

Irrespective of the answers to these questions, such nanoparticles would have advantages of their own. For instance: (i) they help to increase the stability of drugs and possess useful release-control properties; (ii) they offer an increased surface area of action for the drug iii) and enhance efficacy considerably; thereby involve use of lower concentrations of the bioactive agent relative to when used alone [53-55]. Nano-properties i-iii may avail one reason for experimental re-trial of agents like N-9 which has been previously found unsafe for use to prevent HIV or other STI [29]. For such nanoparticles to be applicable in human conditions, it is imperative that we not only determine their size and Zeta potential but safety. In the past, dynamic laser light scattering from the Malvern Zetasizer 3000HAs system (Malvern Instruments, Worcestershire, UK) at 25°C at a 90° angle using PCS 1.61 software has been used to determine both nanoparticle size and Zeta potential [54,55].

The "live microbicide" model also raises unique questions that can only be answered experimentally. First, there is still a need for in situ experiments to evaluate the efficacy of surface anchored HveCt expression by xREPLAB-tN1 in the same way that Liu et al have for 2D CD4 [38]. Previous expression of HveCt in insect line lines does not guarantee that it will be successfully expressed in *Lactobacillus*. Therefore, the efficiency of xREPLAB-tN1 engineering in respective to HveCt surface expression needs be determined by either (i) Partial purification of HveCt(tN1), (ii) Western analysis of HveCt expression in xREPLAB-N1, (iii) growth phase evaluation of HveCt productivity, or (iv) HSV-2 gD binding assays using whole-cell *Lactobacillus *extracts and affinity-purified anti-nectin1 antibodies (R7), as has been done elsewhere [38,52]. In situ experiments are also required to evaluate potential EcoRII expression, say by Phage (λ) DNA digestion assays following REase elution from *L*. *jensenni *whole cell extracts using electrophoresis, as described elsewhere [56]. Lastly, testing the in vitro safety and efficacy of **"xREPLAB-tN1" **is mandatory prior to clinical application in humans. We have found no example of a eukaryotic virus infecting a bacterium, so it cannot be guaranteed outright that surface anchoring of HveCt would enable HSV-2 to be diverted into *Lactobacilli*.

Finally, many genomes of bacteriophages contain unusual nucleic acids bases [19,20]. For example, the T-even coliphage DNA contains not cytosine but 5-hydroxymethylcytosine, and most of the hydroxymethylcytosine residues in these DNAs are glycosylated as well [20]. The genome of the *B*. *subtilis *phage contains a diversity of thymidine replacements, including uracil, 5-hydroxymethylcytosine, glycosylated or phosphorylated 5 uracil and alpha-glutamyl thymine. These unusual bases serve to render the phage genome resistant to degradation by host restriction enzymes [19,20]. It is likely that HSV-2 may become resistant to REase cleavage through similar variations in the viral genomes. This is a likely mechanism for the evolution of resistance to REase-based microbicides. Moreover, R-M systems do not operate with 100% efficiency, and a small number of phages have been noted to survive and produce progeny in bacteria [19,20]. This too may be a shortcoming of REase-based microbicides. We believe that such resistance may be overcome in future by altering the specificity of EcoRII. This concept is based on the fact that among R-M systems of the same class, transfer of the hsdS specificity gene (or protein) occurs naturally and serves to alter the specificity of the "R-M progeny" [19,20]. Similar alterations may be achieved through recombinant engineering, which implies application of the other 8 REases with potent cleavage potential against the HSV-2 genome, but with characteristics that make them less than ideal for use in either proposed model. Again, whether the transfer of specificity subunits from REase such as those derived from the *Bacillus spp*. would entail the persistence of unfavorable characteristics, such as functioning best at temperature ranges outside the normal human physiological range, can only be answered by experiments in situ.

## Conclusion

We identify the REase EcoRII as a potential ingredient of HSV-2 microbicides. Modeled for the first time ever are (i) a nanoparticle for use in research and development of microbicides against HSV-2, and (ii) a "live microbicide" for diverting primary HSV-2 infection from genital mucosal cells coupled to genome disruption. Surfactants with safer profiles may form better candidates for conjugating to EcoRII.

## Methods

### A. Identification of REase with potential activity against HSV-2 genome

#### Design

In silco genome-wide palindromics

#### Materials and software

the whole genome of HSV-2 (PAN = NCBI| NC_001798|); 289 REases and the bioinformatics software Webcutter2 

#### Interventions

we searched for genome splicing sites in a linear pattern in order to recognize 6 or more base-pair palindromes compatible with recognition sites of the 289 REase.

#### Measured Variables

cutting enzymes; frequency of splices and specificity palindrome

### B. Modeling of the chemical bonding of the nanoparticle nano-N-9-EcoRII

B1. Chemical structure of nonoxynol-9: Was modeled from that available literature on surfactant groups of microbicides [29]. The Chemical structure of Savvy C31G was also modeled from that available in literature [28,30]

B2. Physicochemical properties of EcoRII

#### Design

In silco Proteomics

#### Material and Software

Protparam Software ; and the EcoRII enzyme accession number = SWISS PROT |P14633|

#### Interventions

Direct feeding of amino acid sequences of EcoRII into the protparam interface

#### Measured variables

chemical formula of EcoRII and its possible molecular structure Other measured variables included number of atoms, amino acid composition, instability index, aliphatic index, theoretical PI, in-vivo half life and grand average hydropathy (GRAVY).

B3. The likely 3-D structure of EcoRII was obtained from the EMBL protein database using the entry number 1nas6 

#### C. Modeling of a recombinant lactobacillus for diverting primary mucosal HSV infection

C1. Primary accession of CD258 antigen; also known as tumor necrosis factor ligand superfamily member 14, which acts as herpesvirus entry mediator-ligand and nectin-1 (also CD111 antigen; herpes virus entry mediator C) were obtained to show that proteins are readily recognized.

C2. A review of the strategies for modifying the plasmid vectors (i) pLEM7, (ii) pOSEL144 pOSEL651, (iii) pVT-Bac, (iv) PBG38 and (v) pCK285 to generate super plasmids for expression of heterologous proteins in Lactobacillus was done as described elsewhere [38,52].

## Competing interests

All authors are affiliated to Restrizymes Biotherapeutics, a Ugandan biotech pioneering PRINT_GSX for antiviral therapy R&D.

## Authors' contributions

WM conceived of the study, carried out the boinformatics analysis and participated in writing the draft manuscript. WM, BW and KH participated in the modeling, coordinating and writing the final manuscript. All authors read and approved the final manuscript.

## Accession Numbers

HSV-2 whole genome = NCBI| NC_001798|; EcoRII enzyme protein sequence = SWISS PROT |P14633|; HVEM ligand(aka CD258 antigen) primary accession number(PAN) = SWISSPROT = |O43557|; nectin-1(aka CD111 antigen) PAN = SWISSPROT|Q15223|, EcoRII mutant R88A 3-D structure PDB entry = EMBL |1nas6|

## Availability & requirements







## Supplementary Material

Additional File 1 In-silico palindromic analysis of the 287 study REases in the HSV-2 genome. The data provided represents the various potential cleavage sites in the HSV-2 genome by the 287 REases analyzed. Click here for file

Additional File 2 Protparam physicochemical characterization of EcoRII. The data provided represents the protein parameter prediction on the REase EcoRII computed using the protparam software. Click here for file
