# Supplementary material for: Identification of restriction endonuclease with potential ability to cleave the HSV-2 genome: Inherent potential for biosynthetic versus live recombinant microbicides
Source: Theor Biol Med Model. 2008 Aug 7;5:18. doi: 10.1186/1742-4682-5-18 (PMC2526989; doi:10.1186/1742-4682-5-18)
Supplement: Additional File 1 — In-silico palindromic analysis of the 287 study REases in the HSV-2 genome. The data provided represents the various potential cleavage sites in the HSV-2 genome by the 287 REases analyzed. [file 1742-4682-5-18-S1.doc]

# HSV-2 "live" microbicide

## 154746 base pairs

[Graphic map](http://rna.lundberg.gu.se/cgi-bin/cutter2/cutter#GraphicMap%23GraphicMap) | [Table by enzyme name](http://rna.lundberg.gu.se/cgi-bin/cutter2/cutter#TableByEnzyme%23TableByEnzyme)

AccB1I NarI HaeII MspA1I
 KasI BbiII EheI DsaI Cfr42I
 BsmFI Eco64I BsaHI BbeI NspBII
agtccccgtcctgccgcgcgggggcgggcgcgggaaaaaagccgcgcgggggcgcccgcgggaaggcagccccgc base pairs
tcaggggcaggacggcgcgcccccgcccgcgcccttttttcggcgcgcccccgcgggcgcccttccgtcggggcg 1 to 75
 BanI Msp17I Bsp143II SstII
 BshNI Hsp92I BstDSI Sfr3
 Hin1I AcyI BstH2I KspI

 Bst71I Sfr303I AccB1I NarI HaeII MspA1I BstZI AccBSI BstD102I NaeI
 DsaI SstII KasI BbiII EheI DsaI Cfr42I EagI BsrBI Bse118I BsaOI
 BbvI MspA1I BsePI Eco64I BsaHI BbeI NspBII EaeI EclXI NgoMI BsiEI BsmFI
ggcgcgcggggggaggggcggcgcccgcgggggagcggccggctccgggggagggacggggaagggggcgcgcgg base pairs
ccgcgcgcccccctccccgccgcgggcgccccctcgccggccgaggccccctccctgccccttcccccgcgcgcc 76 to 150
 BstDSI Cfr42I BanI Msp17I Bsp143II SstII CfrI MroNI NgoAIV BstMCI BsePI
03I NspBII SacII BshNI Hsp92I BstDSI Sfr303I XmaIII BssAI Cfr10I BssHII
SacII KspI BssHII Hin1I AcyI BstH2I KspI SacII Eco52I BsrFI Bsh1285I

 Eco88I HindII
 PspAI PspALI
 BbvI Ama87I HincII
ggctgccctgccgcccgcccgccgccgccgcccgccttcgcgcccccccccaaaaaacaccccccccgggggttg base pairs
ccgacgggacggcgggcgggcggcggcggcgggcggaagcgcgggggggggttttttgtggggggggcccccaac 151 to 225
 Bst71I BcoI XmaI
 Cfr9I SmaI
 AvaI BsoBI

 Eco88I MspCI PspOMI BmyI Cfr9I
 PleI AvaI Bst98I Bsp120I Eco24I BcoI
 PspAI BsoBI HinfI BsmFI BspTI MseI DraII BanII
actccccgggggaaaagaggcggggcgggagtccccgtcctgccgccgccccttaagagggcccgcaacacggcc base pairs
tgaggggcccccttttctccgccccgccctcaggggcaggacggcggcggggaattctcccgggcgttgtgccgg 226 to 300
HinfI Cfr9I PspALI PleI AflII Tru1I Bsp1286I Ama87I
 Ama87I SmaI Vha464I EcoO109I ApaI PspAI
 BcoI XmaI BfrI Tru9I SduI FriOI Eco88I

 SrfI AviII MaeIII PmlI Hsp92II
 XmaI BbvI Bse1I Eco72I PaeI
AvaI BsoBI Acc16I BsmFI HphI MaeI BsrSI AflIII BbrPI NspI
cgggctgcgcacgccagccgggacgggtgagttcgctaggcaagcacggactggcggttacacgtgcatgcgtgc base pairs
gcccgacgcgtgcggtcggccctgcccactcaagcgatccgttcgtgcctgaccgccaatgtgcacgtacgcacg 301 to 375
 SmaI Bst71I BfaI BseNI MaeII MslI SphI
 PspALI BsrI BsaAI NlaIII
 FspI PmaCI BbuI

 FriOI
 BmyI BsmFI
 HgaI SduI NlaIII
cgagtgaactctcccgccccgacgcgctccggctccgggcctacgccgagcccagccgcccgccatgtcccgccg base pairs
gctcacttgagagggcggggctgcgcgaggccgaggcccggatgcggctcgggtcggcgggcggtacagggcggc 376 to 450
 Bsp1286I Hsp92II
 Eco24I
 BanII

 EcoO109I EcoO109I BanI BbiII EheI Bse118I EaeI NaeI BstMCI BpmI
 PpuMI Psp5II PpuMI Psp5II AccB1I AcyI NgoAIV BbeI EagI Bsh1285I Bsp143II
 SinI AvaII SinI AvaII KasI Hsp92I BssAI Bsp143II EclXI BsaOI GsuI
ccggggtccccgccgccggggtccccggcgccggccgcgccccggcgctccagccgtgccgcgccccggcgctcc base pairs
ggccccaggggcggcggccccaggggccgcggccggcgcggggccgcgaggtcggcacggcgcggggccgcgagg 451 to 525
 Bme18I Bme18I Eco64I Msp17I MroNI Cfr10I BstZI Eco52I HaeII HaeII
 HgiEI Eco47I HgiEI Eco47I Hin1I NarI NgoMI HaeII CfrI BsiEI BstH2I
 DraII BsmFI DraII BsmFI BshNI BsaHI BsrFI BstH2I XmaIII Bsp143II BstH2I

 Eco47I BsiWI Ama87I KspI
 HgiEI PspLI HinfI PleI MspA1I
 GsuI BssHII HinfI SinI BsmFI Csp6I Eco88I NspBII
agccgtgccgcgccccggcgcgctcccaaccgcagactcccaaatggtccctgcgtacgactcgggaaccgcggt base pairs
tcggcacggcgcggggccgcgcgagggttggcgtctgagggtttaccagggacgcatgctgagcccttggcgcca 526 to 600
 BsePI PleI Bme18I SplI RsaI AvaI BstDSI
 BpmI AvaII Pfl23II BcoI DsaI SstII
 SunI AfaI BsoBI Sfr3

 SacII BsaOI NgoMI BstZI BsiEI BshNI EcoRII
 BsiEI BssHII NgoAIV EagI Bsh1285I BstH2I Bst71I Bsp1286I
 TthHB8I MroNI Cfr10I XmaIII HgaI Bsp143II Eco64I BstNI
cgagagcgcgccggccgcgtcctcgctcctgcggcgctggctgctggtgccccaggcggacgacagcgacgacgc base pairs
gctctcgcgcggccggcgcaggagcgaggacgccgcgaccgacgaccacggggtccgcctgctgtcgctgctgcg 601 to 675
 Cfr42I BstMCI BsrFI CfrI Eco52I HaeII BbvI SduI Bst2UI
 Bsh1285I BssAI EaeI NaeI BstMCI BanI BmyI MvaI
 03I TaqI BsePI Bse118I EclXI BsaOI AccB1I BstOI

 NgoAIV BsoBI AccB1I NarI
 NgoMI Eco88I KasI BbiII
HgaI MroNI Cfr10I HgaI Ama87I Eco64I BsaHI
ggactacgccggcaacgacgacgcagagtgggcgaacagccccccgagcgagggcggggggaaggcgccggaggc base pairs
cctgatgcggccgttgctgctgcgtctcacccgcttgtcggggggctcgctcccgccccccttccgcggcctccg 676 to 750
 BssAI NaeI BcoI BanI Msp17I
 BsrFI AvaI BshNI Hsp92I
 Bse118I Hin1I AcyI

 HaeII BstH2I
EheI DraII Bsp143II
 BbeI BbvI
cccgcacgccgcgcctgccgccgcctgccccccgccgccgccgcgcaaggagcgcgggccgcagcgcccccttcc base pairs
gggcgtgcggcgcggacggcggcggacggggggcggcggcggcgcgttcctcgcgcccggcgtcgcgggggaagg 751 to 825
 Bsp143II Bst71I
 EcoO109I HaeII
 BstH2I

 MvaI Bst71I BstNI BsePI BsrFI
 BstOI BstH2I AviII AfaI AscI BssAI NaeI
 EcoRII Bsp143II FspI Csp6I Bst2UI DdeI SgrAI Bse118I
gccccacctggcgctacggctgcgcaccacgacggagtacctggcgcgcctgagcctgcgccggcggcggccccc base pairs
cggggtggaccgcgatgccgacgcgtggtgctgcctcatggaccgcgcggactcggacgcggccgccgccggggg 826 to 900
 BstNI HaeII BbvI RsaI MvaI BstDEI MroNI Cfr10I
 Bst2UI Acc16I EcoRII BssHII NgoMI
 BstOI NgoAIV

 KspI SacII BsaHI
 DsaI SstII AfaI BbiII
 HgaI BstDSI Cfr42I Csp6I Hin1I
cgcgtccccgcccgcggacgcgccgcgcgggaaggtacgcctcccctccgaccccctgacgcccctccgaccccc base pairs
gcgcaggggcgggcgcctgcgcggcgcgcccttccatgcggaggggaggctgggggactgcggggaggctggggg 901 to 975
 BsmFI NspBII HgaI RsaI Msp17I
 MspA1I Hsp92I
 Sfr303I AcyI HgaI

 BsaHI BsaHI BsaHI BsaHI
 BbiII BbiII BbiII BbiII
 Hin1I Hin1I Hin1I Hin1I
tgacgcccctccgaccccctgacgcccctccgaccccctgacgcccctccgaccccctgacgcccctccgacccc base pairs
actgcggggaggctgggggactgcggggaggctgggggactgcggggaggctgggggactgcggggaggctgggg 976 to 1050
 Msp17I Msp17I Msp17I Msp17I
 Hsp92I Hsp92I Hsp92I Hsp92I
 AcyI HgaI AcyI HgaI AcyI HgaI AcyI HgaI

 BsaHI
 BbiII Esp1396I
 Hin1I DrdI Alw26I BspMI BsgI AccB7I
ctgacgcccctccgacccccgtgtctccccgcccgcaggtgtgcttctcgccgcgcgtgcaggtgcgccatctgg base pairs
gactgcggggaggctgggggcacagaggggcgggcgtccacacgaagagcggcgcgcacgtccacgcggtagacc 1051 to 1125
 Msp17I BsmAI BspMI PflMI
 Hsp92I Van91I
 AcyI HgaI

 EcoRII EagI Eco52I BssHII MvaI PpuMI Psp5II MvaI BstMCI BsrFI
 CfrI EclXI Bsh1285I PshAI DraII BstNI BstD102I PinAI BsiEI
 Bst2UI EaeI XmaIII BstMCI Bst2UI EcoO109I BstOI AccBSI AgeI Cfr1
tggcctgggagacggccgcgcgcctggcccgacgggggtcctgggcgcgcgagcgggccgaccgcgaccggttcc base pairs
accggaccctctgccggcgcgcggaccgggctgcccccaggacccgcgcgctcgcccggctggcgctggccaagg 1126 to 1200
 BstNI BstZI BsmBI BsaOI BstNI SinI AvaII Bst2UI BsrBI BsiEI BssAI Bs
 MvaI Alw26I BsiEI EcoRII Bme18I EcoRII BsePI Bsh1285I Bse118I
 BstOI BsmAI Esp3I BsePI BstOI HgiEI Eco47I BssHII BsaOI BsaWI BstMCI

BsaOI BshNI BsaHI BstH2I Eco52I Bme18I EcoRII BpmI SduI BanII Ecl136II BsoBI
 BanI Msp17I Bsp143II EclXI CpoI AvaII MvaI Eco88I FriOI AvaI Ama87I AspHI
 0I Eco64I Hsp92I HaeII EagI BstMCI HgiEI BstNI Ama87I BmyI BcoI AluI AvaI
ggcgccgcgtggcggcggccgaggcggtcatcggaccgtgcctggagcccgaggcccgagctcgggcccgagccc base pairs
ccgcggcgcaccgccgccggctccgccagtagcctggcacggacctcgggctccgggctcgagcccgggctcggg 1201 to 1275
 h1285I Hin1I AcyI EaeI XmaIII BsaOI CspI BstOI BcoI Bsp1286I Eco88I Eco88I
 KasI BbiII EheI BstZI BsiEI SinI Eco47I GsuI BsoBI Ama87I EcoICRI SduI
 AccB1I NarI BbeI CfrI Bsh1285I RsrII Bst2UI AvaI Eco24I BsoBI BcoI Bsp1286I

 Psp124BI PspOMI SduI BanII SduI FriOI Cfr9I BmyI SrfI Bsp1286I BpuAI AvaII
 FriOI Bsp120I BsoBI ApaI BsoBI Ama87I XmaI Eco24I Bsp120I ApaI BpiI Eco47I
BmyI SacI SstI BcoI Bsp1286I BcoI BmyI BcoI BsoBI BanII PspOMI BanII SinI BstDSI
gagcccgggcccacgaagacggcggacccgcggaggaggaggaggcggcggcggcggcgcgcgggtcctccgccg base pairs
ctcgggcccgggtgcttctgccgcctgggcgcctcctcctcctccgccgccgccgccgcgcgcccaggaggcggc 1276 to 1350
 Bbv12I Alw21I Eco88I Eco24I Eco88I BanII AvaI SduI PspALI BmyI MboII Bme18I
 Eco24I BsiHKAI AvaI FriOI AvaI Eco24I PspAI Bsp1286I SduI FriOI BbsI HgiEI
 BanII Ama87I BmyI Ama87I Bsp1286I Eco88I FriOI SmaI Eco24I Bbv16II DsaI

 KspI SacII Bme18I Eco47I Sfr303I PspAI SduI FriOI BfaI BstZI EclXI BsrFI
 SstII BssHII DraII DsaI SstII Ama87I BsoBI ApaI SmaI Cfr10I MroNI Bse118I
 Cfr42I SinI EcoO109I MspA1I PspOMI Eco88I BanII BssAI CfrI Eco52I Cfr1
ccgcgggcccgggccgtcgggcggtctagggttgaaccggcgagggcggcctcggccggcggagccccggagctc base pairs
ggcgcccgggcccggcagcccgccagatcccaacttggccgctcccgccggagccggccgcctcggggcctcgag 1351 to 1425
 NspBII BseRI PpuMI Psp5II KspI Bsp120I AvaI BmyI PspALI Bse118I BssAI BsiE
 MspA1I BseRI HgiEI BstDSI Cfr42I BcoI XmaI Eco24I MaeI EaeI EagI NgoMI Bsh1285I
 Sfr303I BsePI AvaII NspBII SacII Cfr9I Bsp1286I SrfI BsrFI XmaIII NgoAIV

 BstMCI FriOI SduI Eco24I BsiHKAI Ksp632I Eco24I SduI ApaI
 Bsp1286I EcoICRI Psp124BI BstD102I BsmBI FriOI EcoO109I BanII
 0I SduI Ecl136II BmyI FriOI AccBSI Alw26I SduI Bsp120I FriOI
cgaaggtctgcgcgaggccgctctccgaagagacgatgggagccccgcgtatatatccgcgagggcccggcgccg base pairs
gcttccagacgcgctccggcgagaggcttctctgctaccctcggggcgcatatataggcgctcccgggccgcggc 1426 to 1500
 I NaeI BmyI AluI Bbv12I Alw21I Eam1104I Bsp1286I PspOMI Eco24I
 Eco24I AspHI SacI SstI MboII BsmAI BmyI DraII BmyI Eco64I
 BsaOI BanII Bsp1286I BanII BsrBI EarI Esp3I BanII Bsp1286I

 AccB1I AcyI AccBSI BstOI AccB1I NarI BssAI Bsp143II BglI Eco24I PleI
 Hin1I NarI BbeI BstD102I KasI BbiII EheI Bse118I EaeI Bsp120I ApaI
 KasI Hsp92I HaeII EcoRII Eco64I BsaHI BsrFI BstH2I BsePI SduI BanII
ccccgccgctccgcccgccccagggggcggcgccggccaaccgcgcgccgccgcgcgggcccggactccgccccg base pairs
ggggcggcgaggcgggcggggtcccccgccgcggccggttggcgcgcggcggcgcgcccgggcctgaggcggggc 1501 to 1575
 BshNI BsaHI BstH2I BstNI BanI Msp17I MroNI Cfr10I NaeI Bsp1286I
 Msp17I Bsp143II Bst2UI BshNI Hsp92I NgoAIV BbeI BssHII BmyI HinfI
 BanI BbiII EheI BsrBI MvaI Hin1I AcyI NgoMI HaeII CfrI PspOMI FriOI

 NgoAIV Eco47I EclXI BstMCI
 BstMCI NgoMI Tsp509I Tru1I HgiEI CfrI BsmFI FokI
 BsiEI MroNI Cfr10I Sse9I Tru9I SinI EaeI Eco52I BstF5I
gcgaccgccccgcgccggcttcccggtatggtaattagaaacttttaataggcggtcccggccgccatccccgcg base pairs
cgctggcggggcgcggccgaagggccataccattaatctttgaaaattatccgccagggccggcggtaggggcgc 1576 to 1650
 Bsh1285I BssAI NaeI TspEI MseI Bme18I XmaIII BsaOI
 BsaOI BsrFI AvaII EagI BsiEI
 Bse118I BstZI Bsh1285I

 Tsp509I NgoAIV TspEI MseI
 Sse9I Tru1I NgoMI BstD102I Bst71I
 NlaIII Tru9I MroNI Cfr10I AccBSI AluI
catggtaattagcaacttttaatgggccggcgttcccgctcgcggtaattagcagcttttaacgggccgccattc base pairs
gtaccattaatcgttgaaaattacccggccgcaagggcgagcgccattaatcgtcgaaaattgcccggcggtaag 1651 to 1725
 Hsp92II MseI BssAI NaeI BsrBI BbvI
 TspEI BsrFI Sse9I Tru9I
 Bse118I Tsp509I Tru1I

 MseI BsoBI
 Tsp509I Tsp509I Eco88I
 Sse9I MaeII Sse9I Ama87I
ccgcttatggtaattaaaaacgttcggacggcccctcgctccccgcgtaattactccctcggggttccgggttat base pairs
ggcgaataccattaatttttgcaagcctgccggggagcgaggggcgcattaatgagggagccccaaggcccaata 1726 to 1800
 TspEI Psp1406I TspEI BcoI
 Tru9I AvaI
 Tru1I

 Bsp143I Eco47I
 MboI DpnI HgiEI
 BssHII DpnII SinI
gctgattactttcttggcagaacacgcagagcctcgcgcgccgccgggtgggtgggctgatcggcccctattggt base pairs
cgactaatgaaagaaccgtcttgtgcgtctcggagcgcgcggcggcccacccacccgactagccggggataacca 1801 to 1875
 BsePI NdeII Bme18I
 Sau3AI AvaII
 Kzo9I

 MvaI Tsp509I Eco88I BshNI BstDEI BsrFI Eco64I
 BstNI TspEI PspAI PspALI SduI BssAI BglI KasI
 BsmFI MaeI Sse9I Ama87I Eco64I DdeI SgrAI Bse118I
cccctgggcttcctagtatgctaatgaatttttccccgggggcgggcaccactcagggccgcgccggcggggcgc base pairs
ggggacccgaaggatcatacgattacttaaaaaggggcccccgcccgtggtgagtcccggcgcggccgccccgcg 1876 to 1950
 Bst2UI BfaI AcsI BcoI XmaI BanI BmyI MroNI Cfr10I
 BstOI ApoI Cfr9I SmaI AccB1I NgoMI NaeI BshNI
 EcoRII AvaI BsoBI Bsp1286I NgoAIV BanI

 BbiII Bsp143II NspI Eco47I
 Hsp92I HaeII PleI Hsp92II AfaI HgiEI
 Msp17I EheI BsmFI HgaI PaeI MslI Csp6I SinI
cggggggactcccatctgcgtcggcggggggcggcgcatgctaatggggttcttggagtacacccggttggtccc base pairs
gcccccctgagggtagacgcagccgccccccgccgcgtacgattaccccaagaacctcatgtgggccaaccaggg 1951 to 2025
 AccB1I AcyI HinfI NlaIII RsaI Bme18I
 BsaHI BstH2I BbuI AvaII
 Hin1I NarI BbeI SphI Ama87I

 AvaI SmaI BsoBI
 PspAI BsmFI Eco88I
BcoI XmaI Ama87I HinfI BssHII HinfI
cggggacggggccgccccgagagggggggattccctccctccgcccccgccggggcgcgcggctattgggggaat base pairs
gcccctgccccggcggggctctccccccctaagggagggaggcgggggcggccccgcgcgccgataaccccctta 2026 to 2100
 Eco88I BsmFI BcoI TfiI BsePI TfiI
 Cfr9I PspALI AvaI
 BsoBI

 AccB1I NarI HaeII
 KasI BbiII EheI Bst71I BstMCI
 Eco64I BsaHI BbeI BbvI Tsp45I BsiEI
cgtaaatgccgcccctttgggggagtggataggcgccgggtataaggcagccccgtgtgacggtcgggccgcatt base pairs
gcatttacggcggggaaaccccctcacctatccgcggcccatattccgtcggggcacactgccagcccggcgtaa 2101 to 2175
 BanI Msp17I Bsp143II MaeIII Bsh1285I
 BshNI Hsp92I BsaOI
 Hin1I AcyI BstH2I


 BsaMI BstD102I BsmAI BsmAI
 BsmI TspRI AccBSI BseRI Eco31I Alw26I
cgcaccccggcactgcgagcgacggagcggcggcccggcgggaggaggagacccggagagacagagactaaaacc base pairs
gcgtggggccgtgacgctcgctgcctcgccgccgggccgccctcctcctctgggcctctctgtctctgattttgg 2176 to 2250
 Mva1269I BsrBI Alw26I BsmAI Alw26I
 BsaI


 Eco31I Cfr42I Eco88I Alw26I
 BstDSI SstII Ama87I TthHB8I PleI
 Alw26I Sfr303I BcoI BsoBI BsmBI NlaIII
cggcaagagagagaccgcgggccgccgtctcgagtctaccctaccccggctcatggaaccccggcccggcacgag base pairs
gccgttctctctctggcgcccggcggcagagctcagatgggatggggccgagtaccttggggccgggccgtgctc 2251 to 2325
 BsmAI MspA1I Sfr274I TaqI HinfI Hsp92II
 DsaI NspBII XhoI PaeR7I Esp3I
 BsaI KspI SacII AvaI BsmAI AccI

EcoICRI Eco24I SstI Cfr9I SmaI Eco47I BstZI EclXI Bsh1285I KspI SacII Eco88I
BssSI BmyI SacI BsiHKAI XmaI HgiEI Eco88I EagI BsrBI BsaOI MspA1I BcoI XmaI
BsiI Bsp1286I BanII PspAI PspALI Ama87I CfrI Eco52I BstMCI NspBII HphI AvaI
ctcccgggcggaccccggccccgagcggccgccgcggcagacccccggcacggtgagagggcgacccccgggtct base pairs
gagggcccgcctggggccggggctcgccggcggcgccgtctgggggccgtgccactctcccgctgggggcccaga 2326 to 2400
Ecl136II Psp124BI BcoI AvaI SinI BcoI EaeI XmaIII BsiEI DsaI SstII Ama87I BsoBI
AluI AspHI FriOI Ama87I BsoBI AvaII BsoBI NotI BstD102I BglI Cfr42I PspAI PspALI
 SduI Bbv12I Alw21I Eco88I Bme18I AvaI CciNI AccBSI BstDSI Sfr303I Cfr9I DrdI

 BstDEI DraII Eco47I BsoBI Eco24I
 DdeI EcoO109I HgiEI Eco88I
 SmaI BsaI SinI BbvI Ama87I BanII
caggcccccccttttccccggaccacccggctgcgggttgggggtggtcgcgggcggtgggctcgggggcgggga base pairs
gtccgggggggaaaaggggcctggtgggccgacgcccaacccccaccagcgcccgccacccgagcccccgcccct 2401 to 2475
 Eco31I Bme18I Bst71I BcoI Bsp1286I
 BsmAI AvaII AvaI BmyI
 Alw26I SduI FriOI

 MspCI BstMCI HgiEI BsmFI
 Bst98I BsiEI PpuMI Psp5II
 BsmFI BspTI MseI BsaOI EcoO109I
cgcttgacggggccgacccccggcccgcttaagcggtcgggggacccccgtgggccgtgcgccgccccccgaccc base pairs
gcgaactgccccggctgggggccgggcgaattcgccagccccctgggggcacccggcacgcggcggggggctggg 2476 to 2550
 HgaI AflII Tru1I SinI AvaII DsaI
 Vha464I Bsh1285I DraII BstDSI
 BfrI Tru9I Bme18I Eco47I

 AvaI BmyI BsrBI AhdI
 BcoI Bsp1286I BsmFI BsmBI HinfI
 BseRI SduI AccBSI Alw26I AspEI
tctgggggggcgagggaggcagggaggagcccgagagcgggggacagggggggagacgaggggtcggaatccaaa base pairs
agacccccccgctccctccgtccctcctcgggctctcgccccctgtccccccctctgctccccagccttaggttt 2551 to 2625
 Ama87I FriOI BstD102I BsmAI Eam1105I
 Eco88I BanII Esp3I TfiI
 BsoBI Eco24I EclHKI

 AvaII
 Bme18I BbvI
 HgaI MaeIII NspBII
ggacgcagaccacctttggttacggacccctttctcccccccttccgaacaaaaagcagcgggcggggggccggg base pairs
cctgcgtctggtggaaaccaatgcctggggaaagagggggggaaggcttgtttttcgtcgcccgccccccggccc 2626 to 2700
 SinI MspA1I
 HgiEI Bst71I
 Eco47I

 EcoO109I
 PpuMI Psp5II
 HphI BsmFI BsmFI SinI AvaII
gtgagggagggacacgggggacacggcgcgggggtcccgcctcacgccccgcgccctctaaatcccccccgttgc base pairs
cactccctccctgtgccccctgtgccgcgcccccagggcggagtgcggggcgcgggagatttagggggggcaacg 2701 to 2775
 Bme18I
 HgiEI Eco47I
 DraII BsmFI

 MvaI
 BstOI SfaNI NspI TspRI HinfI
 BbvI EcoRII BstF5I NlaIII BbvI PleI
tttgtcaagcagcccgccgccccgcacgcctgggggatgctcaacgacatgcagtggctcgccagcagcgactcg base pairs
aaacagttcgtcgggcggcggggcgtgcggaccccctacgagttgctgtacgtcaccgagcggtcgtcgctgagc 2776 to 2850
 Bst71I BstNI FokI Hsp92II Bst71I
 Bst2UI


 BsmAI
 BseRI Eco31I HinfI HphI PleI BbvI
gaggaggagaccgaggtgggaatctctgacgacgaccttcaccgcgactccacctccgaggcgggcagcacggac base pairs
ctcctcctctggctccacccttagagactgctgctggaagtggcgctgaggtggaggctccgcccgtcgtgcctg 2851 to 2925
 Alw26I TfiI HinfI Bst71I
 BsaI


 XmaIII EcoRII Bst2UI
 CfrI CfrI BsiEI Bsp143II
 MslI TthHB8I EaeI EaeI Eco52I HaeII MvaI
acggagatgttcgaggcgggcctgatggacgcggccacgcccccggcccggcccccggccgagcgccagggcagc base pairs
tgcctctacaagctccgcccggactacctgcgccggtgcgggggccgggccgggggccggctcgcggtcccgtcg 2926 to 3000
 TaqI HgaI BstZI BstMCI BstNI
 EagI Bsh1285I BstOI
 EclXI BsaOI BstH2I

 MboI Bsp143I PspOMI FriOI
 BstX2I XhoII Bsp120I BanII
 HgaI BstYI BamHI AlwI SduI BstDSI BseRI
cccacgcccgccgacgcgcagggatcctgtgggggtgggcccgtgggtgaggaggaagcggaagcgggagggggg base pairs
gggtgcgggcggctgcgcgtccctaggacacccccacccgggcacccactcctccttcgccttcgccctcccccc 3001 to 3075
BbvI DpnII MflI DpnI Bsp1286I HphI
 NdeII BstI AclWI BmyI DsaI
Bst71I Sau3AI Kzo9I Eco24I ApaI

 Bsp1286I Sau3AI
 VneI BmyI DpnII DpnI
 MaeII BcgI SduI BsiHKAI Kzo9I BbvI BsgI
ggcgacgtgtgtgccgtgtgcacggacgagatcgccccgcccctgcgctgccagagttttccctgcctgcacccc base pairs
ccgctgcacacacggcacacgtgcctgctctagcggggcggggacgcgacggtctcaaaagggacggacgtgggg 3076 to 3150
 AflIII Alw44I Alw21I Bsp143I Bst71I
 ApaLI Bbv12I MboI
 AspHI NdeII

 BpuAI Bst2UI PmlI
 FokI MboII BstNI Acc16I BsaAI AfaI
 SfaNI NlaIII BbsI MvaI FspI MaeII BsmFI MslI Csp6I
ttctgcatcccgtgcatgaagacctggattccgttgcgcaacacgtgtcccctgtgcaacaccccggtggcgtac base pairs
aagacgtagggcacgtacttctggacctaaggcaacgcgttgtgcacaggggacacgttgtggggccaccgcatg 3151 to 3225
 BstF5I Hsp92II BpiI HinfI AviII Eco72I RsaI
 EcoRII BstOI AflIII BbrPI
 Bbv16II TfiI PmaCI

 Eco47I
 HgiEI
 Tsp45I NspBII BstF5I SinI
ctgatagtgggcgtgaccgccagcgggtcgttcagcaccatcccgatagtgaacgacccccggacccgcgtggag base pairs
gactatcacccgcactggcggtcgcccagcaagtcgtggtagggctatcacttgctgggggcctgggcgcacctc 3226 to 3300
 MaeIII MspA1I FokI Bme18I
 AvaII


 CfrI BsiEI BssAI NaeI BstZI BsiEI KspI SacII
 EaeI Eco52I NgoMI SfiI CfrI MslI BstMCI NspBII
 SfiI XmaIII MroNI Cfr10I EagI Bsh1285I BstDSI Cfr42I
gccgaggcggccgtgcgggccggcacggccgtggactttatctggacgggcaacccgcggacggccccgcgctcc base pairs
cggctccgccggcacgcccggccgtgccggcacctgaaatagacctgcccgttgggcgcctgccggggcgcgagg 3301 to 3375
 BglI EclXI BsaOI Bse118I XmaIII DsaI DsaI SstII
 BstZI BstMCI BsrFI BglI EclXI BstDSI MspA1I
 EagI Bsh1285I NgoAIV EaeI Eco52I BsaOI Sfr303I

 AspEI HgiEI
 EclHKI CspI
 BsmFI SinI AvaII BstDSI
ctgtcgctggggggacacacggtccgcgccctgtcgcccacccccccgtggcccggcacggacgacgaggacgat base pairs
gacagcgacccccctgtgtgccaggcgcgggacagcgggtgggggggcaccgggccgtgcctgctgctcctgcta 3376 to 3450
 AhdI Bme18I DsaI
 Eam1105I Eco47I
 CpoI RsrII

 MvaI
 BstOI
 EcoRII CfrI HphI
gacctggccgacggtgagggcgggcgggggtcgggcggggggcgggcgggggtcgggcgggggtcgggcgggggt base pairs
ctggaccggctgccactcccgcccgcccccagcccgccccccgcccgcccccagcccgcccccagcccgccccca 3451 to 3525
 BstNI
 Bst2UI
 EaeI


cgggcgggggtcgggcgggggtcgggcgggggtcgggcgggggtcgggcgggggtcgggcgggggtcgggcgggg base pairs
gcccgcccccagcccgcccccagcccgcccccagcccgcccccagcccgcccccagcccgcccccagcccgcccc 3526 to 3600


 FriOI Esp3I MaeII
 BmyI BmyI BsmAI BsmAI AspI Ksp632I
 SduI SduI Alw26I SfcI TspRI BsmFI MboII
gtcgggcactaaccgggggctcccgtctctgtctccctctgcagtggactacgtcccgcccgccccccgaagagc base pairs
cagcccgtgattggcccccgagggcagagacagagggagacgtcacctgatgcagggcgggcggggggcttctcg 3601 to 3675
 Bsp1286I Bsp1286I Alw26I PstI Tth111I Eam1104I
 Eco24I BsmBI BstSFI AtsI EarI
 BanII

SapI KspI SacII BsiEI BanI
 NspBII Bse118I KasI
 HaeII BstDSI Cfr42I BssAI BstMCI
gccccggcgcgggggcggcggtgcgggggcgacccgcggaacctcccagcccgccgcgacccgaccggcgccccc base pairs
cggggccgcgcccccgccgccacgcccccgctgggcgccttggagggtcgggcggcgctgggctggccgcggggg 3676 to 3750
 DsaI SstII BsrFI BsaOI
 BstH2I MspA1I Cfr10I Eco64I
 Bsp143II Sfr303I Bsh1285I BshNI

 Hin1I NarI BbeI Bst2UI Msp17I Bsp143II KspI SacII Eco64I Msp17I Bsp143II Sau3AI
 BbiII EheI BstNI BanI Hin1I AcyI BstDSI Cfr42I BbvI KasI Hsp92I HaeII BstX2I
 Msp17I Bsp143II MvaI AccB1I NarI BbeI MspA1I Bst71I BanI BbiII EheI DpnII
tggcgccccgcggagcagcagcagcggcggcgccccgttgcgggcgggggtgggatctgggtctgggggcggccc base pairs
accgcggggcgcctcgtcgtcgtcgccgccgcggggcaacgcccgcccccaccctagacccagacccccgccggg 3751 to 3825
 AccB1I AcyI EcoRII KasI BbiII EheI DsaI Sfr303I MspA1I AccB1I NarI BbeI NdeII
 Hsp92I HaeII BstOI BshNI BsaHI BstH2I SstII NspBII BshNI BsaHI BstH2I MboI
 BsaHI BstH2I Eco64I Hsp92I HaeII NspBII BbvI Bst71I Hin1I AcyI BstYI Bsp143I

 AlwI EagI BstMCI CfrI MroNI Bse118I BsePI
 DpnI EaeI Eco52I EarI EaeI Eco52I BsiEI NaeI
 MflI AclWI XmaIII BsaOI Eam1104I EclXI BsrFI BstMCI
tgccgtcgcggccgtcgtgccgagagtggcctctcttccccctgcggccggcggggggcgcgcgcaggcgcggcg base pairs
acggcagcgccggcagcacggctctcaccggagagaagggggacgccggccgccccccgcgcgcgtccgcgccgc 3826 to 3900
 Kzo9I CfrI BsiEI Ksp632I XmaIII NgoAIV BsaOI
 XhoII BstZI Bsh1285I MboII BstZI NgoMI Bsh1285I
 BglI EclXI EagI BssAI Cfr10I BssHII

 BsaHI BbsI BstDSI SacII AcyI MvaI FriOI
 BbiII Bbv16II Sfr303I Msp17I BstOI BmyI
 Hin1I BpuAI NspBII BglI Hsp92I Alw26I EcoRII SduI
ggtgggcgaagacgccgcggcggcggagggcaggacgccccccgcgagacagccccgcgcggcccaggagccccc base pairs
ccacccgcttctgcggcgccgccgcctcccgtcctgcggggggcgctctgtcggggcgcgccgggtcctcggggg 3901 to 3975
 Msp17I BpiI MspA1I Hin1I HgaI BsmAI BstNI Bsp1286I
 Hsp92I HgaI KspI Cfr42I BsaHI Bst2UI Eco24I
 AcyI MboII DsaI SstII BbiII BanII

 KspI BglI DraII BcoI AvaI ApaI
 BsmBI NspBII SacII SduI PspAI FriOI AccBSI
 HinfI Alw26I BstDSI Cfr42I Ama87I Eco24I BsoBI
catagtcatcagcgactctcccccgccgtctccgcgccgccccgcgggccccgggccgctctcctttgtctcctc base pairs
gtatcagtagtcgctgagagggggcggcagaggcgcggcggggcgcccggggcccggcgagaggaaacagaggag 3976 to 4050
 PleI BsmAI DsaI SstII PspOMI BmyI Eco88I PspALI
 Esp3I MspA1I Bsp120I Bsp1286I BanII BsrBI
 Sfr303I EcoO109I Cfr9I XmaI SmaI

 Alw26I BsoBI DraII PspAI FriOI SmaI
 BseRI Eco88I SduI BcoI AvaI ApaI BsePI
 BstD102I Ama87I Ama87I Cfr9I BanII AscI BssHII
ctcctccgcacaggtgtcctcgggccccggggggggaggtctgccacagtcgtcggggcgcgccgcgcgcccccg base pairs
gaggaggcgtgtccacaggagcccggggcccccccctccagacggtgtcagcagccccgcgcggcgcgcgggggc 4051 to 4125
 BseRI BcoI PspOMI BmyI Eco88I PspALI BssHII BsePI
 AvaI EcoO109I Eco24I BsoBI
 BsmAI Bsp120I Bsp1286I XmaI

 XmaIII BsaWI HinfI
 CfrI BsiEI MroI Bsp13I
 EaeI Eco52I BspEI BsiMI BstDSI
cgcggccgtcgccccgcgcgtccggagtccgccccgcgccgccgccgcccccgtggtgtctgcgagcgcggacgc base pairs
gcgccggcagcggggcgcgcaggcctcaggcggggcgcggcggcggcgggggcaccacagacgctcgcgcctgcg 4126 to 4200
 BstZI BstMCI AccIII PleI DsaI
 EagI Bsh1285I Kpn2I HgaI
 EclXI BsaOI BseAI

EagI Bsh1285I Eco24I BstOI
HgaI Eco52I SduI FriOI Bse118I BstMCI Hsp92II DdeI
EaeI EclXI Bsp120I BssAI HgaI BssHII BsiEI NlaIII MvaI
ggccgggcccgcgccgcccgccgtgccggtggacgcgcaccgcgcgccccggtcgcgcatgacccaggctcagac base pairs
ccggcccgggcgcggcgggcggcacggccacctgcgcgtggcgcgcggggccagcgcgtactgggtccgagtctg 4201 to 4275
BstZI BstMCI Bsp1286I BsrFI BsePI Bsh1285I EcoRII BstDEI
CfrI BsiEI PspOMI BanII Cfr10I BsaOI BstNI
XmaIII BsaOI BmyI ApaI Bst2UI

 BssHII
 PleI BstMCI
 HinfI BglI BsiEI HgaI
cgacacccaagcacagagtctgggccgggcaggcgcgaccgacgcgcgcgggtcgggagggccgggcgcggaggg base pairs
gctgtgggttcgtgtctcagacccggcccgtccgcgctggctgcgcgcgcccagccctcccggcccgcgcctccc 4276 to 4350
 AlwNI Bsh1285I
 BsaOI
 BsePI

 EcoO109I PspAI PspALI Bme18I Eco47I KspI SacII BanI NspBII
 PpuMI Psp5II AvaI AhdI PpuMI Psp5II MspA1I BanI BshNI KspI SacII
 SinI AvaII Cfr9I SmaI SinI DraII BstDSI Cfr42I Eco64I MspA1I
aggacccggggtcccccgcggcaccaacacccccggtgccgccccccacgccgcggagggggcggcggcccgccc base pairs
tcctgggccccagggggcgccgtggttgtgggggccacggcggggggtgcggcgcctcccccgccgccgggcggg 4351 to 4425
 Bme18I BcoI Eco88I Eam1105I AvaII DsaI SstII BshNI BstDSI Cfr42I
 HgiEI Eco47I XmaI EclHKI HgiEI BsmFI Sfr303I AccB1I DsaI SstII
 DraII Ama87I BsoBI AspEI EcoO109I NspBII Eco64I AccB1I Sfr303I

 AvaI PspOMI Eco24I KspI SacII
 Ksp632I BcoI Bsp120I BanII MspA1I EarI
 MboII HinfI PleI Bsp1286I DsaI SstII Eam1104I
ccggaagaggcgcgggtcggactcgggccccgcggcctcgtcctccgcctcttcctccgccgccccgcgctcgcc base pairs
ggccttctccgcgcccagcctgagcccggggcgccggagcaggaggcggagaaggaggcggcggggcgcgagcgg 4426 to 4500
 Eam1104I Ama87I DraII FriOI NspBII Ksp632I
 EarI Eco88I SduI BmyI BstDSI Cfr42I MboII
 BsoBI EcoO109I ApaI Sfr303I

 MvaI PflMI AccB1I NarI HaeII DraII FriOI Ama87I Bsh1285I
 BstOI KasI BbiII EheI PspOMI Eco24I HinfI BsiEI
 EcoRII AccB7I BglI Eco64I BsaHI BbeI EcoO109I HinfI AvaI BstDSI
cctcgccccccagggggtgggggccaagagggcggcgccgcgccgggccccggactcggactcgggcgaccgcgg base pairs
ggagcggggggtcccccacccccggttctcccgccgcggcgcggcccggggcctgagcctgagcccgctggcgcc 4501 to 4575
 BstNI Van91I BanI Msp17I Bsp143II Bsp1286I PleI BsoBI
 Bst2UI BshNI Hsp92I Bsp120I BmyI ApaI Eco88I DsaI
 Esp1396I Hin1I AcyI BstH2I SduI BanII BcoI PleI BstMCI

 CfrI BstDSI BbiII BstDSI Cfr42I Msp17I Bsp143II BsaHI BsmBI BsmFI KspI
 NspBII SstII AccBSI BsaHI NspBII SacII Hin1I NarI BbeI Hsp92I BstNI BstDSI
 EaeI Cfr42I Hin1I HgaI Sfr303I BshNI BsaHI BstH2I HgaI EcoRII DsaI
ccacgggccgctcgccccggcgtccgcgggcgccgcgcccccgtcggcgtctccgtcgtcccaggccgcggtcgc base pairs
ggtgcccggcgagcggggccgcaggcgcccgcggcgcgggggcagccgcagaggcagcagggtccggcgccagcg 4576 to 4650
BsaOI KspI DsaI Msp17I DsaI SstII KasI Hsp92I HaeII BbiII BsmAI Bst2UI
 MspA1I SacII BstD102I AcyI KspI Eco64I BbiII EheI Msp17I Alw26I BstOI NspBII
 Sfr303I BsrBI Hsp92I MspA1I BanI AccB1I AcyI Hin1I AcyI Esp3I MvaI MspA1I

 BsiEI
 Cfr42I BseRI
Sfr303I BsaOI BseRI BseRI BseRI BseRI BseRI BseRI BseRI BseRI
cgccgcctcctcctcctccgcctcctcctcctccgcctcctcctcctccgcctcctcctcctccgcctcctcctc base pairs
gcggcggaggaggaggaggcggaggaggaggaggcggaggaggaggaggcggaggaggaggaggcggaggaggag 4651 to 4725
SstII BstMCI
 SacII
 Bsh1285I


 EarI
 BseRI BseRI BseRI BseRI BseRI Eam1104I HgaI
ctccgcctcctcctcctccgcctcctcctcctccgcctcttcctctgcgggcggggctggtgggagcgtcgcgtc base pairs
gaggcggaggaggaggaggcggaggaggaggaggcggagaaggagacgcccgccccgaccaccctcgcagcgcag 4726 to 4800
 Ksp632I
 MboII


 KspI SacII
 BstH2I BsmBI DsaI SstII
HgaI HgaI Bsp143II Alw26I BbvI BstDSI Cfr42I
cgcgtccggcgctggggagagacgagaaacctccctcggcccccgcgctgctgcgccgcgggggccgaggaagtg base pairs
gcgcaggccgcgacccctctctgctctttggagggagccgggggcgcgacgacgcggcgcccccggctccttcac 4801 to 4875
 HaeII BsmAI Bst71I NspBII BglI
 Esp3I MspA1I
 Sfr303I

 MvaI BpiI BsoBI PspAI SduI BanII SduI BmyI Eco64I Hsp92I
 BstOI Bbv16II Eco88I AvaI BsoBI SmaI DraII BanII BshNI BsaHI
 EcoRII MboII Ama87I Eco88I Eco24I Bsp120I FriOI KasI BbiII
tgccaggaagacgcgccacgcggagggcggccccgagcccggggcccgcgacccggcgcccggcctcacgcgcta base pairs
acggtccttctgcgcggtgcgcctcccgccggggctcgggccccgggcgctgggccgcgggccggagtgcgcgat 4876 to 4950
 BstNI BpuAI BcoI Ama87I BmyI PspALI Bsp1286I BanI Msp17I
 Bst2UI BbsI AvaI BcoI XmaI FriOI EcoO109I ApaI Hin1I NarI
 HgaI Cfr9I Bsp1286I PspOMI Eco24I AccB1I AcyI

 BbeI Eco88I Alw26I EcoRII BanI BbiII Bsp143II
 HaeII XhoI PaeR7I HgaI MvaI BshNI BsaHI BstH2I
 EheI Sfr274I Eco31I BstNI KasI Hsp92I HaeII MaeIII BsmFI
cctgcccatcgcgggggtctcgagcgtcgtggccctggcgccttacgtgaacaagacggtcacgggggactgcct base pairs
ggacgggtagcgcccccagagctcgcagcaccgggaccgcggaatgcacttgttctgccagtgccccctgacgga 4951 to 5025
 Bsp143II Ama87I TaqI BsmAI Eco64I Msp17I EheI BsaAI
 BstH2I BcoI BsoBI BsaI BstOI AccB1I NarI BbeI Tsp45I
 BspMI AvaI TthHB8I Bst2UI Hin1I AcyI MaeII

 MvaI BsmBI HgiEI HgiEI Eco47I
 BstOI Alw26I BsaAI Bme18I HgiEI
 EcoRII NlaIII EcoO109I SinI BsiI Eco47I MaeII SinI
gcccgtcctggacatggagacgggccacataggggcctacgtggtcctcgtggaccagacggggaacgtggcgga base pairs
cgggcaggacctgtacctctgcccggtgtatccccggatgcaccaggagcacctggtctgccccttgcaccgcct 5026 to 5100
 BstNI Hsp92II DraII MaeII Eco47I SinI Bme18I
 Bst2UI BsmAI Bme18I BssSI AvaII
 Esp3I AvaII AvaII

 MspA1I BsoBI Bbv12I
 Bst71I KspI SacII Eco88I AspHI BsePI
 BspMI BstDSI Cfr42I Ama87I SduI BsiHKAI EcoO1
cctgctgcgggccgcggcccccgcgtggagccgccgcaccctgctccccgagcacgcgcgcaactgcgtgaggcc base pairs
ggacgacgcccggcgccgggggcgcacctcggcggcgtgggacgaggggctcgtgcgcgcgttgacgcactccgg 5101 to 5175
 BbvI NspBII BcoI Bsp1286I DraII
 DsaI SstII AvaI BmyI BssHII
 Sfr303I Alw21I

 BsaHI
 BbiII NspI
 09I Hin1I HgaI AlwNI BstF5I NlaIII
ccccgactacccgacgccccccgcgtcggagtggaacagcctctggatgaccccggtgggcaacatgctctttga base pairs
ggggctgatgggctgcggggggcgcagcctcaccttgtcggagacctactggggccacccgttgtacgagaaact 5176 to 5250
 Msp17I FokI Hsp92II
 Hsp92I
 AcyI HgaI

 MvaI BshNI BstNI BsePI BsaOI BsoBI BsmAI
 BstOI SduI MslI MvaI Bsh1285I Eco88I BsePI
EcoRII BanI BmyI Bst2UI BstDSI BsaWI BstDSI Ama87I AscI
ccagggcaccctggtgggcgcgctggacttccacggcctccggtcgcgccacccgtggtctcgggagcagggcgc base pairs
ggtcccgtgggaccacccgcgcgacctgaaggtgccggaggccagcgcggtgggcaccagagccctcgtcccgcg 5251 to 5325
 BstNI AccB1I BstOI DsaI BsiEI DsaI BcoI Eco31I BssHII
 Bst2UI Bsp1286I BssHII BstMCI AvaI BsaI
 Eco64I EcoRII Alw26I

 NgoAIV EagI BsiEI Msp17I DsaI SstII
 NgoMI BstZI Eco52I BbiII BstDSI Cfr42I
 MroNI Cfr10I Bsh1285I Hsp92I Sfr303I AluI BbvI
gcccgcgccggccggcgacgcccccgcgggccacggggagtagggggagctaacactcggcttgctgcccgaagg base pairs
cgggcgcggccggccgctgcgggggcgcccggtgcccctcatccccctcgattgtgagccgaacgacgggcttcc 5326 to 5400
 BssAI EaeI EclXI Hin1I HgaI MspA1I DsaI Bst71I
 BsrFI CfrI NaeI BsaOI BsaHI KspI SacII
 Bse118I XmaIII BstMCI AcyI NspBII BstDSI

 AvaII Cfr10I EclXI Eco64I Hsp92I HaeII Eco88I
 Bme18I EaeI XmaIII BsaOI Hin1I NarI BbeI AvaI
 BsaWI BssAI CfrI Bsh1285I AccB1I EheI BcoI BglI
aagccgccccccaccggaccaccggccgaggcgcctcgggggcagggggaggtggggggggggaaagacggggag base pairs
ttcggcggggggtggcctggtggccggctccgcggagcccccgtccccctccacccccccccctttctgcccctc 5401 to 5475
 SinI BsrFI EagI BstMCI BshNI BsaHI BstH2I
 HgiEI Bse118I BsiEI BanI Msp17I Bsp143II BsoBI
 Eco47I BstZI Eco52I KasI BbiII AcyI Ama87I


 BsmAI
 BseRI BsmFI
gagacaggaagtgggggtgggagtgggggggggggacggacacggccccgaacagcaacacacaccagcattttg base pairs
ctctgtccttcacccccaccctcaccccccccccctgcctgtgccggggcttgtcgttgtgtgtggtcgtaaaac 5476 to 5550
 Alw26I


 Tsp509I
 TspEI
 Sse9I
ttatggactttctggccttgttgaaaacttgaggaaaaaaaaaactttatatttataaaaattttacaataaagt base pairs
aatacctgaaagaccggaacaacttttgaactccttttttttttgaaatataaatatttttaaaatgttatttca 5551 to 5625
 AcsI
 ApoI


 BbvI
 SfaNI SfaNI BstF5I
tttgtgatgcttttgacacactttgttgttggcctttgatgcagctcccccgcgcaggggggccggggatggggg base pairs
aaacactacgaaaactgtgtgaaacaacaaccggaaactacgtcgagggggcgcgtccccccggcccctaccccc 5626 to 5700
 AluI FokI
 Bst71I


 BssSI BsoBI AatI
 BmyI Eco88I
 BseRI BseRI SduI Ama87I SseBI
ggaagggaggaggaggagggggggcgggcacgagaagccgcccccacccccgaggcctgttggtctttatcatag base pairs
ccttccctcctcctcctcccccccgcccgtgctcttcggcgggggtgggggctccggacaaccagaaatagtatc 5701 to 5775
 Bsp1286I BcoI Pme55I
 BsiI AvaI StuI
 Eco147I

 SduI ApaI
 EcoO109I BanII
 Bsp120I FriOI
aacagagccggggcccggcctcgttctggctccctgtcttggtgggtgggcgggctggctggcgggtaaaaaaag base pairs
ttgtctcggccccgggccggagcaagaccgagggacagaaccacccacccgcccgaccgaccgcccatttttttc 5776 to 5850
 PspOMI Eco24I
 DraII BmyI
 Bsp1286I

 SduI ApaI Bsp143I BstMCI AspHI EaeI BsrFI EcoRII
 EcoO109I BanII Kzo9I Bsh1285I BmyI MaeII NgoAIV AlwNI
 HincII Bsp120I FriOI MboI BspCI BsaOI Alw21I BssAI NaeI
agtgtgtccgtgttgacagggaggggggcccgatcgtgcagagcacgcacgtctggccggccagaccctgggggt base pairs
tcacacaggcacaactgtccctccccccgggctagcacgtctcgtgcgtgcagaccggccggtctgggaccccca 5851 to 5925
 HindII PspOMI Eco24I Sau3AI Ple19I Bsp1286I CfrI Bse118I
 DraII BmyI DpnII DpnI PvuI SduI BsiHKAI NgoMI FseI
 Bsp1286I NdeII BsiEI BsgI Bbv12I MroNI Cfr10I

 BstNI AccB1I NarI Bsp143II BcoI Cfr10I
 BstOI KasI BbiII EheI BstNI Eco88I BsrFI
AccB7I Bst2UI Eco64I BsaHI BstH2I Ama87I SgrAI EcoRII
ggtgggcaggagtgggagggcgcctggctcggggagggaggagggggggggtcagccgcaccaccggcgcgaagc base pairs
ccacccgtcctcaccctcccgcggaccgagcccctccctcctcccccccccagtcggcgtggtggccgcgcttcg 5926 to 6000
 PflMI MvaI BanI Msp17I EcoRII MvaI AvaI BssAI
 Van91I BshNI Hsp92I BbeI BstOI BsoBI Bse118I
 Esp1396I Hin1I AcyI HaeII Bst2UI BseRI

 MvaI MvaI NspI
 BstOI BstOI Hsp92II
 EcoRII HinfI PaeI
caggggccagggaactttgatagagaggggggaaagtggggcgggggcgagggcggttgaatcacaacgcatgca base pairs
gtccccggtcccttgaaactatctctcccccctttcaccccgcccccgctcccgccaacttagtgttgcgtacgt 6001 to 6075
 BstNI BstNI TfiI NlaIII
 Bst2UI Bst2UI BbuI
 SphI

 Eco88I MboII Eco88I NspI
 PspAI PspALI Ksp632I Bbv16II BbuI
 Ama87I BsmFI BseRI MboII BpuAI AvaI Hsp92II
cgccctctgcccccggggacgggtgggaggaaggaggagggagaagagaagacccgaggcatgcacccgcactta base pairs
gcgggagacgggggcccctgcccaccctccttcctcctccctcttctcttctgggctccgtacgtgggcgtgaat 6076 to 6150
 BcoI XmaI Eam1104I BbsI PaeI
 Cfr9I SmaI EarI Ama87I BsoBI SphI
 AvaI BsoBI BcoI BpiI NlaIII

 AccB1I NarI HaeII BstOI
 BmyI KasI BbiII EheI HphI
 SduI Eco64I BsaHI BbeI BspMI BseRI
cgcccgtgcccacccccgccccggcgcccaccccgcccgcacacctgcccgccacgcccgcccctcctcaccctg base pairs
gcgggcacgggtgggggcggggccgcgggtggggcgggcgtgtggacgggcggtgcgggcggggaggagtgggac 6151 to 6225
 Bsp1286I BanI Msp17I Bsp143II EcoRII
 BshNI Hsp92I BstNI
 Hin1I AcyI BstH2I Bst2UI

 BseRI AvaI Hsp92II
 Ksp632I Eco88I NlaIII
MvaI BseRI MboII Ama87I Eco31I SphI HphI
gctgggagaaaggaggaggagcaggaagaggagacccgaggcatgcaaccgcactcaccccaccccgcccgcaca base pairs
cgaccctctttcctcctcctcgtccttctcctctgggctccgtacgttggcgtgagtggggtggggcgggcgtgt 6226 to 6300
 Eam1104I BsmAI PaeI
 EarI Alw26I BsaI NspI
 BcoI BsoBI BbuI

 MvaI FriOI
 BstOI BsmAI BmyI
 BspMI EcoRII BbvI HinfI SduI
cctgcccgccacgcccgcccctccttaccctggctgcggggagactcccatcggggcgagggggctcgcgcgttc base pairs
ggacgggcggtgcgggcggggaggaatgggaccgacgcccctctgagggtagccccgctcccccgagcgcgcaag 6301 to 6375
 BstNI Bst71I Alw26I Bsp1286I
 Bst2UI PleI Eco24I
 BanII


 HgaI
 AflIII
gcaacaccacaccacaccacacggcccaccacaacacggcccaccacgacacaacacgacacgacgcgttttgcg base pairs
cgttgtggtgtggtgtggtgtgccgggtggtgttgtgccgggtggtgctgtgttgtgctgtgctgcgcaaaacgc 6376 to 6450
 MluI


 NspI AccI
 Hsp92II HindII
 PaeI SalI BcgI BssHII MaeI
gggcatgcaagtcgacacaccgcgcgcgtgcctacctttccctagcggccccggcccccggcccgtttccttccg base pairs
cccgtacgttcagctgtgtggcgcgcgcacggatggaaagggatcgccggggccgggggccgggcaaaggaaggc 6451 to 6525
 NlaIII HincII BsePI BfaI
 BbuI TthHB8I
 SphI TaqI

 BshNI Hsp92I BbeI MslI
 BanI Msp17I Bsp143II
 BsmFI Hin1I AcyI BstDSI
ccaccactaccaccacccccccgcccgcgcccacgcggtagaggaaggggacgggcgccacacccacggctgtgg base pairs
ggtggtgatggtggtgggggggcgggcgcgggtgcgccatctccttcccctgcccgcggtgtgggtgccgacacc 6526 to 6600
 Eco64I BsaHI BstH2I EaeI
 KasI BbiII EheI DsaI
 AccB1I NarI HaeII BstXI

 PspEI BssAI SunI AfaI Eco24I
CfrI BmyI BstPI MaeIII Pfl23II SduI ApaI
 SduI Eco91I BsrFI SplI RsaI BmyI
ccgggcacgcgcctttggggttgttgggggggggtgaccggcgcgtgggggcggtgggcgtacgggcccgacccg base pairs
ggcccgtgcgcggaaaccccaacaacccccccccactggccgcgcacccccgccacccgcatgcccgggctgggc 6601 to 6675
 Bsp1286I BstEII HphI PspLI Bsp120I BanII
 EcoO65I Cfr10I BsiWI PspOMI FriOI
 Tsp45I Bse118I Csp6I Bsp1286I

 Eco88I
 PspAI PspALI Ksp632I
 Ama87I MboII
cgcctgcccccccgggaacgacgacgggggggggggaaacgggggtgggtggaagggaagaggaaggagaaaggg base pairs
gcggacgggggggcccttgctgctgccccccccccctttgcccccacccaccttcccttctccttcctctttccc 6676 to 6750
 BcoI XmaI Eam1104I
 Cfr9I SmaI EarI
 AvaI BsoBI

 MboI Bsp143I DpnII BamHI AlwI
 BstX2I XhoII BstYI Bsp143I
 BstYI BamHI AlwI MboI MflI DpnI
ggggtggatccgaacacgccggatccgcgaaaataataacaaaacaaacaaaaacagaaacaaaaacaaaaacac base pairs
ccccacctaggcttgtgcggcctaggcgcttttattattgttttgtttgtttttgtctttgtttttgtttttgtg 6751 to 6825
 DpnII MflI DpnI NdeII BstI AclWI
 NdeII BstI AclWI Sau3AI Kzo9I
 Sau3AI Kzo9I BstX2I XhoII

 EcoO109I
 PpuMI Psp5II
 MaeI BspMI SinI AvaII
ctagaaaaaaaggatacgggttggctcgcgggcggtgcggctgacctgcctgccctttctgggacccccgcctcg base pairs
gatctttttttcctatgcccaaccgagcgcccgccacgccgactggacggacgggaaagaccctgggggcggagc 6826 to 6900
 BfaI Bme18I
 HgiEI Eco47I
 DraII BsmFI

 EarI
 EarI Eam1104I
 BsiI MboII Eam1104I
tgtttcttgaaagggggaggaagaacagttctcccccaacccctgctctcttctctcttccgcccgccccccccc base pairs
acaaagaactttccccctccttcttgtcaagagggggttggggacgagagaagagagaaggcgggcggggggggg 6901 to 6975
 BssSI Ksp632I
 MboII Ksp632I
 MboII


 RsaI
 DdeI AluI HphI BspMI TthHB8I
cctctccccgccgcctcagcagaagctcacctgtacgaccctaaacctacctgcgagaacgcgcggcgttcgagg base pairs
ggagaggggcggcggagtcgtcttcgagtggacatgctgggatttggatggacgctcttgcgcgccgcaagctcc 6976 to 7050
 BstDEI Csp6I TaqI
 AfaI


 AccB1I NarI HaeII MvaI PpuMI Psp5II
 BsePI Alw26I KasI BbiII EheI BstOI EcoO109I
 BsiI Eco64I BsaHI BbeI EcoRII Bme18I Eco47I
ggcgcgctctctcacacgagacacacgcaggcgccccccccccccggagcctgggtcccccggcggacggctcac base pairs
ccgcgcgagagagtgtgctctgtgtgcgtccgcggggggggggggcctcggacccagggggccgcctgccgagtg 7051 to 7125
 BssHII BssSI BanI Msp17I Bsp143II BstNI HgiEI BsmFI
 BsmAI BshNI Hsp92I Bst2UI DraII
 Hin1I AcyI BstH2I SinI AvaII

 BsaHI BsmBI
 BbiII BsmAI
 Hin1I Alw26I BsmFI BseRI
gcggcgcggcgtctcggtgggacgcgggcaaagggcggcggcggcgggggggggggggggaaatgtgaggagagc base pairs
cgccgcgccgcagagccaccctgcgcccgtttcccgccgccgccgccccccccccccccctttacactcctctcg 7126 to 7200
 Msp17I Esp3I HgaI
 Hsp92I
 AcyI HgaI

 BpiI
 Ksp632I Bbv16II
 Alw26I MboII BsmFI MboII BseRI
gagacagagagagagaaggaagagggaaggggcgcggcgggacgggggaagacgaggagaagggaaggggcgagg base pairs
ctctgtctctctctcttccttctcccttccccgcgccgccctgcccccttctgctcctcttcccttccccgctcc 7201 to 7275
 BsmAI Eam1104I BpuAI
 EarI BbsI


 PspAI BsoBI ApaI BstD102I BsoBI BsrFI Ama87I
 Ama87I Bsp1286I SmaI BcoI XmaI BssAI NaeI PspAI
 Bsp120I AvaI BmyI PspALI PspAI PspALI SgrAI Bse118I AvaI
gtcgggcccgggagcggggcggcccgggagggagaagaaacggaacgcggaaacgccgccggcgcggcccggggc base pairs
cagcccgggccctcgccccgccgggccctccctcttctttgccttgcgcctttgcggcggccgcgccgggccccg 7276 to 7350
 PspOMI Eco88I FriOI AccBSI Eco88I MboII MroNI Cfr10I Eco88I
 BcoI XmaI Eco24I BsrBI Cfr9I SmaI NgoMI BglI Cfr9I
 Cfr9I SduI BanII Ama87I AvaI NgoAIV BcoI XmaI

SmaI DraII BcoI AvaI ApaI DraII FriOI EcoO109I BsoBI BanII CpoI AvaII BsaHI
BsoBI Ama87I Eco24I BsoBI EcoO109I ApaI BcoI Eco88I FriOI SrfI CspI Msp17I
 Bsp120I Bsp1286I XmaI SmaI SduI BanII Ama87I XmaI Eco24I SmaI RsrII Hsp92I
cccggggcccccgcgctccgccgggggcccgggccggaccgccgggcgggggacgccttccgcccggcgccgggc base pairs
gggccccgggggcgcgaggcggcccccgggcccggcctggcggcccgccccctgcggaaggcgggccgcggcccg 7351 to 7425
 PspOMI BmyI Eco88I PspALI Bsp1286I PspOMI Cfr9I BmyI PspALI HgiEI BbiII
PspALI SduI PspAI FriOI Bsp120I Eco24I DraII AvaI SduI ApaI Bme18I Hin1I BsmFI
 EcoO109I Cfr9I BanII PspOMI BmyI Bsp120I PspAI Bsp1286I SinI Eco47I AcyI

 BanI Msp17I Bsp143II AvaI SinI DraII EaeI Eco52I TfiI DraII Eco24I AccB1I
Eco64I Hsp92I BstH2I Eco88I PpuMI Psp5II EagI BstMCI TaqI SduI BanII KasI
 BshNI BsaHI HaeII PspAI PspALI AvaII BstZI BsiEI TthHB8I Bsp1286I Eco64I
ggctacccgggacccccggccgggaatcgaaaaaagcctccgggggcccctttcgcgcctttcgcgaacgcgcgg base pairs
ccgatgggccctgggggccggcccttagcttttttcggaggcccccggggaaagcgcggaaagcgcttgcgcgcc 7426 to 7500
HgaI Hin1I NarI BbeI Cfr9I SmaI EcoO109I CfrI Bsh1285I EcoO109I ApaI BanI
 AccB1I AcyI Ama87I BsoBI HgiEI BsmFI XmaIII HinfI PspOMI BmyI Bsp68I BshNI
 KasI BbiII EheI BcoI XmaI Bme18I Eco47I EclXI BsaOI Bsp120I FriOI NruI

 NarI HaeII CciNI Bsh1285I Bsp1286I BglI EclXI
 BbiII EheI BstZI EclXI PspOMI Eco24I BstZI BstMCI CfrI
 BsaHI BbeI CfrI Eco52I Bsp120I FriOI EaeI Eco52I EaeI
cgccggagggggcggccgccgaggtgcgggggcccctccggccggggcgcacctcggcggccaagccccggcccg base pairs
gcggcctcccccgccggcggctccacgcccccggggaggccggccccgcgtggagccgccggttcggggccgggc 7501 to 7575
 Msp17I Bsp143II XmaIII BsaOI SduI BanII EagI Bsh1285I
 Hsp92I EaeI EagI BsiEI EcoO109I ApaI CfrI BsiEI
Hin1I AcyI BstH2I NotI BstMCI DraII BmyI SfiI XmaIII BsaOI

 Eco88I Bme18I Psp5II BsoBI Eco47I BpiI
 PspAI PspALI AvaII Eco88I AvaII Bbv16II
 Ama87I SinI DraII BcoI BsmFI MboII
cccgggggtccccgaggcaagaggcggaccctcggaggcgcggaagaagacgggaggcgggggaaaaaaggggga base pairs
gggcccccaggggctccgttctccgcctgggagcctccgcgccttcttctgccctccgcccccttttttccccct 7576 to 7650
 BcoI XmaI PpuMI Eco47I SinI BpuAI
 Cfr9I SmaI HgiEI Ama87I Bme18I BbsI
 AvaI BsoBI EcoO109I AvaI HgiEI

 BssAI BsaWI
 Ksp632I BssHII MroI Bsp13I
 MboII BseRI SgrAI BbvI BspEI BsiMI
agagagggggaggtagggaggggagaggagaagggcgcgccggtgcgcggagcagccttccttctccggagtccc base pairs
tctctccccctccatccctcccctctcctcttcccgcgcggccacgcgcctcgtcggaaggaagaggcctcaggg 7651 to 7725
 Eam1104I AscI Bse118I Bst71I AccIII PleI
 EarI BsePI Cfr10I Kpn2I HinfI
 BsrFI BseAI

BsmFI Sau3AI Ple19I PspOMI Eco24I
 DpnII DpnI Bsh1285I Bsp1286I HgaI
 TthHB8I BsiEI Bsp120I BmyI BbvI BstF5I BbvI
tctcgatcggcggcgggcccctgcgttcgttgctgccgcgcccccggttttataaagacagggatgacgcagcag base pairs
agagctagccgccgcccggggacgcaagcaacgacggcgcgggggccaaaatatttctgtccctactgcgtcgtc 7726 to 7800
 TaqI Bsp143I BstMCI SduI FriOI Bst71I FokI Bst71I
 NdeII BspCI BsaOI DraII BanII
 MboI Kzo9I PvuI EcoO109I ApaI

 BsoBI Eco24I BanII BstX2I DpnI Bsp1286I Hsp92I
 Eco88I SduI BmyI MseI BstYI MflI AlwI KasI BbiII
 Ama87I BanII FriOI Tru1I Sau3AI AclWI BanI Hin1I
aaatgcccacagcaacacgcgggcggggctcgggctctccggcggcttaatggatctccgggcacggcgcccgca base pairs
tttacgggtgtcgttgtgcgcccgccccgagcccgagaggccgccgaattacctagaggcccgtgccgcgggcgt 7801 to 7875
 BcoI Bsp1286I Tru9I MboI Bsp143I Eco64I Msp17I
 AvaI BmyI Bsp1286I DpnII XhoII SduI BshNI
 SduI FriOI Eco24I NdeII Kzo9I BmyI AccB1I

 HaeII AspHI BstDEI BssHII NgoAIV EagI
 EheI Bsp1286I PvuII Tru1I NgoMI BstZI
 AcyI SduI BsiHKAI AscI MaeII Tru9I MroNI Cfr1
accgcagagcactcagctggcgcgcccccccccaacgtgggagtgtttaatggaagggcgtggggccggccgccg base pairs
tggcgtctcgtgagtcgaccgcgcgggggggggttgcaccctcacaaattaccttcccgcaccccggccggcggc 7876 to 7950
 Bsp143II Alw21I NspBII MseI BssAI EaeI
BsaHI BstH2I Bbv12I AluI BsePI BsrFI CfrI
 NarI BbeI BmyI DdeI MspA1I Bse118I XmaIII

 FseI BstF5I MspA1I DraII KasI BbiII EheI DsaI SstII NspBII
 Eco52I SfaNI Sfr303I Eco64I BsaHI HaeII NspBII BstDSI
0I BsiEI FokI NspBII EcoO109I NspBII AccB1I AcyI BstDSI Cfr42I KspI
gatgcccgcgggggcctaatgcggcgggaggcgtgggccgctggcgccgcggcccgtctgctggcccgcggcccg base pairs
ctacgggcgcccccggattacgccgccctccgcacccggcgaccgcggcgccgggcagacgaccgggcgccgggc 7951 to 8025
 EclXI BsaOI BstDSI SacII MspA1I Hin1I NarI BbeI MspA1I DsaI
 NaeI BstMCI DsaI SstII BanI Msp17I Bsp143II Sfr303I MspA1I
 Bsh1285I KspI Cfr42I BshNI Hsp92I BstH2I KspI SacII Sfr3

 SacII EaeI SstII EaeI KspI SacII NgoAIV BsaOI
 Cfr42I NspBII SacII NspBII Sfr303I NgoMI Bsh1285I
 BstDSI Sfr303I BstDSI EagI EclXI BsrFI BstMCI
tctgctggcccgcggccacgtaaacaatgacacaggggttctctccgccgcggccggcgcggggcgttgccggcc base pairs
agacgaccgggcgccggtgcatttgttactgtgtccccaagagaggcggcgccggccgcgccccgcaacggccgg 8026 to 8100
SstII DsaI KspI BsaAI DsaI CfrI XmaIII BssAI BsiEI
 MspA1I MaeII MspA1I SstII Eco52I Bse118I
 03I CfrI Cfr42I BstZI Cfr42I MroNI Cfr10I

 NgoAIV FriOI NspBII Bsp143II CciNI Bst71I NspBII SacII Eco24I
 BsrFI BmyI DsaI SstII BstH2I EagI Eco52I BsaOI SstII BmyI Bsp1286I
 NgoMI BglI SduI BstDSI Cfr42I EaeI NotI BsiEI BstMCI Cfr42I BanII BanII
cggcccggccccggagcccgcggcgctgctcggctgcggccgcgggctccgggggctccgcactctgcccggctc base pairs
gccgggccggggcctcgggcgccgcgacgagccgacgccggcgcccgaggcccccgaggcgtgagacgggccgag 8101 to 8175
 NaeI Bse118I Bsp1286I Sfr303I BbvI CfrI BbvI BstDSI KspI SduI SduI Eco24I
 BssAI NaeI Eco24I KspI SacII Bst71I XmaIII DsaI MspA1I Bsp1286I BmyI
 MroNI Cfr10I BanII MspA1I HaeII BstZI EclXI Bsh1285I Sfr303I FriOI FriOI


 BsmFI BbvI BssHII
gccccgtcccccctcttgctgcttttccgcgcgcctctctttcccgttgctttccctctccccccccccccctct base pairs
cggggcaggggggagaacgacgaaaaggcgcgcggagagaaagggcaacgaaagggagagggggggggggggaga 8176 to 8250
 Bst71I BsePI


 XmaIII
 CfrI BsiEI BstD102I
 BstF5I EaeI Eco52I AccBSI BglI BbvI
ctctctctctctctctctctccgccatcctcccgcccggccgcccactccccgctcggcctctccggctgcggtg base pairs
gagagagagagagagagagaggcggtaggagggcgggccggcgggtgaggggcgagccggagaggccgacgccac 8251 to 8325
 FokI BstZI BstMCI BsrBI Bst71I
 EagI Bsh1285I
 EclXI BsaOI

 BsaHI
 BsaI BbiII PleI
 Alw26I Hin1I HinfI
cttgggtctccttcgtcgggcggcggggggggggcgtcgggactcgcggagggccggagaatggaaggcgagggg base pairs
gaacccagaggaagcagcccgccgccccccccccgcagccctgagcgcctcccggcctcttaccttccgctcccc 8326 to 8400
 Eco31I Msp17I BsmFI
 BsmAI Hsp92I
 AcyI HgaI

 Bsp143I BsmFI
 SfaNI MboI Kzo9I
 BstF5I DpnII DpnI PleI MboII BstF5I
atgcaggaggaggatcgggactccccatcttctgcccttccatcctccgtttttccgctttccaccgccgccgcc base pairs
tacgtcctcctcctagccctgaggggtagaagacgggaaggtaggaggcaaaaaggcgaaaggtggcggcggcgg 8401 to 8475
 FokI NdeII AclWI FokI
 Sau3AI HinfI
 BseRI AlwI

 Eco47I
 HgiEI
 SinI HphI
accacccccccttccttcgcccgcccgcctcgccccggacccctcccccccgtgttccccccatcgttcaccacc base pairs
tggtgggggggaaggaagcgggcgggcggagcggggcctggggagggggggcacaaggggggtagcaagtggtgg 8476 to 8550
 Bme18I
 AvaII


 EcoT14I
 StyI
 Eco130I BbvI BbvI
acgccccccaccgcgccttggctgtttggggggtggcggcggtggtcggcgtgctgccggaggctgcgggcgcgg base pairs
tgcggggggtggcgcggaaccgacaaaccccccaccgccgccaccagccgcacgacggcctccgacgcccgcgcc 8551 to 8625
 ErhI Bst71I Bst71I
 BssT1I


 SduI ApaI Esp3I Bsp143I BsrFI
 EcoO109I BanII BsmAI MboI AclWI BglI
 Bsp120I FriOI HgaI DpnII DpnI Bse118I
ggtaggtgggtgggcgggtggtgggggggggcccggctgcgtctcgccgcgatcccgccggtggggcgcggcggc base pairs
ccatccacccacccgcccaccacccccccccgggccgacgcagagcggcgctagggcggccaccccgcgccgccg 8626 to 8700
 PspOMI Eco24I Alw26I Kzo9I SgrAI
 DraII BmyI BbvI BsmBI Sau3AI BssAI
 Bsp1286I Bst71I NdeII AlwI Cfr10I


 BstMCI BstMCI
 BsiEI BsmFI BsiEI
ggtcggggtggggggagagtgtcgtgggtgtgttttcgtgtcccccaccaccactcccaccccgaccgccgccgc base pairs
ccagccccacccccctctcacagcacccacacaaaagcacagggggtggtggtgagggtggggctggcggcggcg 8701 to 8775
 Bsh1285I Bsh1285I
 BsaOI BsaOI


 Eco47I AvaI BstDSI
 HgiEI PspAI PspALI BshNI
 BssHII SinI BcoI XmaI Eco64I
gcccgcgtttctgccgcccgcgcgctcctgtgtggaccccggggtgggcggcgggggggggtgccgtgggtgtgg base pairs
cgggcgcaaagacggcgggcgcgcgaggacacacctggggccccacccgccgcccccccccacggcacccacacc 8776 to 8850
 BsePI Bme18I Eco88I BanI
 AvaII Cfr9I SmaI AccB1I
 Ama87I BsoBI DsaI

 FriOI HgiEI Bsp143I
 BmyI Bme18I MboI DpnI
 SduI SinI DpnII DraIII
cggcggggcgcgggccggggccggggctcgctggtccgccgaagtaaagaaaagatcgccaccgtgtgttcgtct base pairs
gccgccccgcgcccggccccggccccgagcgaccaggcggcttcatttcttttctagcggtggcacacaagcaga 8851 to 8925
 Bsp1286I NdeII
 Eco24I Eco47I Sau3AI
 BanII AvaII Kzo9I

 AccB1I NarI HaeII DraII FriOI
 KasI BbiII EheI PspOMI Eco24I
 Eco64I BsaHI BbeI EcoO109I
gtgtgttctgcgcggcgccggggcccccctgccgggcggggcggtggggcggggtcggggtcgcggcggggaagg base pairs
cacacaagacgcgccgcggccccggggggacggcccgccccgccaccccgccccagccccagcgccgccccttcc 8926 to 9000
 BanI Msp17I Bsp143II Bsp1286I
 BshNI Hsp92I Bsp120I BmyI ApaI
 Hin1I AcyI BstH2I SduI BanII

 AccB1I NarI MroNI Cfr10I HgaI EclXI BsaOI EagI
 BstH2I KasI BbiII EheI NgoAIV BbeI EagI BstMCI EaeI
 Bsp143II Eco64I BsaHI NgoMI HaeII NaeI XmaIII BssHII XmaIII
aaggaaagaccccggaagcgccgggagggggcgccggcgcgacgcgggcggccgggcgggggcgcgcggcggccg base pairs
ttcctttctggggccttcgcggccctcccccgcggccgcgctgcgcccgccggcccgcccccgcgcgccgccggc 9001 to 9075
 HaeII BanI Msp17I SgrAI Bse118I EaeI Eco52I BsePI EclXI
 BshNI Hsp92I BsrFI BstH2I BstZI BsiEI BstZI
 Hin1I AcyI BssAI Bsp143II CfrI Bsh1285I CfrI

 BstMCI XmaIII XmaIII XmaIII
Eco52I BsePI CfrI BsiEI CfrI BsiEI CfrI BsiEI
 BssHII EaeI Eco52I BssHII EaeI Eco52I BssHII EaeI Eco52I BssHII
ggcgggggcgcgcggcggccgggcgggggcgcgcggcggccgggcgggggcgcgcggcggccgggcgggggcgcg base pairs
ccgcccccgcgcgccgccggcccgcccccgcgcgccgccggcccgcccccgcgcgccgccggcccgcccccgcgc 9076 to 9150
 BsaOI BstZI BstMCI BsePI BstZI BstMCI BsePI BstZI BstMCI BsePI
 BsiEI EagI Bsh1285I EagI Bsh1285I EagI Bsh1285I
 Bsh1285I EclXI BsaOI EclXI BsaOI EclXI BsaOI

 XmaIII XmaIII BsoBI
 CfrI BsiEI CfrI BsiEI Eco88I
 EaeI Eco52I BssHII EaeI Eco52I BssHII HgaI Ama87I
cggcggccgggcgggggcgcgcggcggccgggcgggggcgcgctttccccgcgtcgcccctcgggttcccaagac base pairs
gccgccggcccgcccccgcgcgccgccggcccgcccccgcgcgaaaggggcgcagcggggagcccaagggttctg 9151 to 9225
 BstZI BstMCI BsePI BstZI BstMCI BsePI BcoI
 EagI Bsh1285I EagI Bsh1285I AvaI
 EclXI BsaOI EclXI BsaOI

 PmlI Acc16I
 Eco72I
 MaeII BbrPI HgaI HgaI HgaI
ctatcacgtgtgcgcaggggaggggaggacgcgggggaggggaggacgcgggggaggggaggacgcgggggatat base pairs
gatagtgcacacgcgtcccctcccctcctgcgccccctcccctcctgcgccccctcccctcctgcgccccctata 9226 to 9300
 AflIII FspI
 BsaAI AviII
 PmaCI

 Eco47I
 HgiEI
 SinI HinfI
ataaagcggtagaaagcgcgggaatgggcatattggacccgcgtgattcggttgctcgcggttgtcttgtttgga base pairs
tatttcgccatctttcgcgcccttacccgtataacctgggcgcactaagccaacgagcgccaacagaacaaacct 9301 to 9375
 Bme18I TfiI
 AvaII


 BsrFI
 BssAI BstD102I
MaeII AgeI Cfr10I TspRI AccBSI
cgttttttatgcgggaacaagggggcttaccggttacactgtccgctcgctatggggttcgtctgtctgtttggg base pairs
gcaaaaaatacgcccttgttcccccgaatggccaatgtgacaggcgagcgataccccaagcagacagacaaaccc 9376 to 9450
 PinAI MaeIII BsrBI
 BsaWI
 Bse118I

 MvaI
 BstOI
 EcoRII Tsp45I Asp700I MboII
cttgtcgttatgggagcctggggggcgtggggtgggtcacaggcaaccgaatatgttcttcgtagtgttattgcc base pairs
gaacagcaataccctcggaccccccgcaccccacccagtgtccgttggcttatacaagaagcatcacaataacgg 9451 to 9525
 BstNI MaeIII XmnI
 Bst2UI
 BglI

 NspI HgiEI NspBII
 BstDEI Hsp92II BstDSI Cfr42I BstH2I
 BstXI BsmFI PaeI SinI DsaI SstII Bsp143II
aaagaggtgggggacatactaagagtgccttgcatgcggacccccgcggacgatgtttcttggcgctacgaggcc base pairs
tttctccaccccctgtatgattctcacggaacgtacgcctgggggcgcctgctacaaagaaccgcgatgctccgg 9526 to 9600
 DdeI NlaIII Eco47I Sfr303I HaeII EcoO109I
 BbuI Bme18I KspI SacII DraII
 SphI AvaII MspA1I

 Cfr9I PspALI
 BcoI XmaI
 SspI MboII TspRI AvaI AflIII
ccgtccgttattgactatgcccgcatagacggaatatttcttcgctatcactgcccggggttggacacgtttttg base pairs
ggcaggcaataactgatacgggcgtatctgccttataaagaagcgatagtgacgggccccaacctgtgcaaaaac 9601 to 9675
 Ama87I SmaI MaeII
 PspAI BsoBI
 Eco88I

 HincII
 Tru1I Tsp45I
 BglI Tru9I PshAI
tgggataggcacgcccagagggcgtatctggttaacccctttctctttgcggcgggatttttggaggacttgagt base pairs
accctatccgtgcgggtctcccgcatagaccaattggggaaagagaaacgccgccctaaaaacctcctgaactca 9676 to 9750
 MseI HinfI
 HpaI MaeIII
 HindII

 PleI XmaIII EcoRII SduI BanII
 CfrI BsiEI BstNI PspOMI BmyI
 DraIII EaeI Eco52I BstOI HgaI EcoO109I ApaI SfaNI
cactctgtgtttccggccgacacccaggaaacaacgacgcgccgggccctttataaagagatacgcgatgcgttg base pairs
gtgagacacaaaggccggctgtgggtcctttgttgctgcgcggcccgggaaatatttctctatgcgctacgcaac 9751 to 9825
 BstZI BstMCI MvaI Bsp120I FriOI
 EagI Bsh1285I DraII Eco24I
 EclXI BsaOI Bst2UI Bsp1286I


 TthHB8I
ggcagtcgaaaacaggccgtcagccacgcacccgtcagggccgggtgtgtaaactttgactactcacgcactcgc base pairs
ccgtcagcttttgtccggcagtcggtgcgtgggcagtcccggcccacacatttgaaactgatgagtgcgtgagcg 9826 to 9900
 TaqI


 BbvI HindII
 NspBII HgaI HgaI MaeII MaeII
cgctgcgtcgggcgacgcgatttacggcctgccaacaccacgtcaacgtgggaaccgcctgtgtcgtcggacgat base pairs
gcgacgcagcccgctgcgctaaatgccggacggttgtggtgcagttgcacccttggcggacacagcagcctgcta 9901 to 9975
 MspA1I HincII
 Bst71I


 Bsp1286I BanII TaqI BstBI Bpu14I
 EcoICRI Eco24I BsiHKAI LspI NspV
 Ecl136II SacI Alw21I TthHB8I BstDSI
gaagcgagctcgcagtcgaagcccctcgccacccagccgcccgtcctcgccctttcgaacgcccccccacggcgg base pairs
cttcgctcgagcgtcagcttcggggagcggtgggtcggcgggcaggagcgggaaagcttgcgggggggtgccgcc 9976 to 10050
 AluI BmyI FriOI TthHB8I SfuI TaqI DsaI
 SduI Bbv12I SstI Csp45I
 AspHI Psp124BI Bsp119I

 AspEI KasI BbiII EheI Bse118I NaeI
 BsaI EclHKI AccB1I NarI NgoMI HaeII BfaI
 Alw26I HgaI Eco64I BsaHI BssAI Bsp143II MaeI MaeII SfaNI EcoRII
gtctccccgacgcgaggtcggcgccggcatactcgcctccgacgcaactagccacgtctgcatcgcaagccaccc base pairs
cagaggggctgcgctccagccgcggccgtatgagcggaggctgcgttgatcggtgcagacgtagcgttcggtggg 10051 to 10125
 Eco31I AhdI BanI Msp17I MroNI Cfr10I HgaI
 BsmAI Eam1105I Hin1I AcyI NgoAIV BbeI
 BshNI Hsp92I BsrFI BstH2I

MvaI CfrI BsiEI
BstOI EaeI Eco52I BglI
 MaeI XmaIII BglI HgaI MaeI
tgggtcgggagcaggacagccgacccgtctagcggccgggtcggctgtccagcgtcgtcgccctagaggctgtcc base pairs
acccagccctcgtcctgtcggctgggcagatcgccggcccagccgacaggtcgcagcagcgggatctccgacagg 10126 to 10200
BstNI BfaI EclXI BsaOI BfaI
Bst2UI BstZI BstMCI
 EagI Bsh1285I

 Bst2UI Hsp92I
 BstNI Msp17I
 SfaNI EcoRII BglI SfaNI AcyI
gccgggcgtgatgttttccgcatctacgacccccgaacagcccctggggctgtcgggcgatgcgacgccgcccct base pairs
cggcccgcactacaaaaggcgtagatgctgggggcttgtcggggaccccgacagcccgctacgctgcggcgggga 10201 to 10275
 AlwNI Hin1I HgaI
 BstOI BbiII
 MvaI BsaHI

 EcoRII BsrI Bsp143I Msp17I BstNI
 Bsp1286I BseNI MboI DpnI Hsp92I Bst2UI
 BcgI BstOI BsrSI BssHII DpnII TthHB8I AcyI MvaI
gccgacttccgtgcccctggactgggccgcgtttcggcgcgcgtttctgatcgacgacgcctggcggcccctgtt base pairs
cggctgaaggcacggggacctgacccggcgcaaagccgcgcgcaaagactagctgctgcggaccgccggggacaa 10276 to 10350
 SduI Bst2UI BsePI NdeII TaqI BsaHI BstOI
 BmyI MvaI Bse1I Sau3AI Hin1I EcoRII
 BstNI Kzo9I BbiII HgaI

 Bsp1286I BanII NruI
 EcoICRI Eco24I BsiHKAI BshNI Ksp632I
 Ecl136II SacI Alw21I BseRI Eco64I MboII
ggagccggagctcgcgaaccccctaaccgcgcgcctcctcgcggagtatgaccgtcggtgccagaccgaagaggt base pairs
cctcggcctcgagcgcttgggggattggcgcgcggaggagcgcctcatactggcagccacggtctggcttctcca 10351 to 10425
 AluI BmyI FriOI Bsp68I BanI Eam1104I
 SduI Bbv12I SstI BssHII AccB1I EarI
 AspHI Psp124BI BsePI

 MspA1I MvaI
 DsaI SstII FokI BstOI AfaI
 BbvI NspBII BstF5I BstNI HgaI Csp6I MaeII
gctgccgccgcgggaggatgtgttctcctggacgcggtattgtacccccgacgacgtgcgcgtggttatcatcgg base pairs
cgacggcggcgccctcctacacaagaggacctgcgccataacatgggggctgctgcacgcgcaccaatagtagcc 10426 to 10500
 BstDSI Cfr42I EcoRII RsaI
 Bst71I Sfr303I Bst2UI
 KspI SacII

 EcoO109I AfaI EcoRII MvaI Bse118I
 PpuMI Psp5II EaeI Bst2UI BstOI BssAI BshNI
 SinI AvaII BstF5I EcoRII BstF5I BanI
gcaggacccgtaccaccatcccggccaggcgcacggcctggcgtttagcgtgcgtgcggatgtgccggtgcctcc base pairs
cgtcctgggcatggtggtagggccggtccgcgtgccggaccgcaaatcgcacgcacgcctacacggccacggagg 10501 to 10575
 Bme18I RsaI FokI BstNI BstNI FokI Eco64I
 HgiEI Eco47I CfrI MvaI Bst2UI BsrFI AccB1I
 DraII Csp6I BstOI Cfr10I

 TspEI CfrI EclXI BsiEI
 PleI Tru1I HgaI EaeI XmaIII Bsh1285I
 HinfI MaeII Tru9I MaeIII BssHII NlaIII EagI BsrBI BstMCI
gagtctacggaacgtgctggcggcggttaaaaattgttaccccgacgcgcgcatgagcggccgcggctgcctgga base pairs
ctcagatgccttgcacgaccgccgccaatttttaacaatggggctgcgcgcgtactcgccggcgccgacggacct 10576 to 10650
 AccI MseI BsePI Hsp92II NotI BstD102I
 Sse9I BstZI Eco52I BstDSI
 Tsp509I CciNI AccBSI DsaI

BsaOI Cfr42I BstOI FriOI BsaHI
 Sfr303I BstNI BmyI NruI BbiII
 SstII Bst71I Bsp1286I Hin1I BsmFI
aaagtgggctcgcgacggcgtgctgttgttgaacacgaccctgaccgtcaagcgcggggcggcggcgtcccactc base pairs
tttcacccgagcgctgccgcacgacaacaacttgtgctgggactggcagttcgcgccccgccgccgcagggtgag 10651 to 10725
 MspA1I BbvI Bst2UI Bsp68I Msp17I
 KspI SacII MvaI Eco24I Hsp92I
 NspBII EcoRII SduI BanII AcyI HgaI

 HgiEI Eco47I EaeI Eco88I SmaI MvaI
 AluI SinI BsmFI HgiEI Esp1396I PspAI PspALI
 XcmI BstF5I SinI AccB7I BssHII Ama87I SrfI Bst2UI
caagcttggatgggaccgttttgtgggcggggtggtccaacggctggccgcgcgccgcccgggcctggtctttat base pairs
gttcgaacctaccctggcaaaacacccgccccaccaggttgccgaccggcgcgcggcgggcccggaccagaaata 10726 to 10800
 HindIII FokI Eco47I Bme18I PflMI BsePI BcoI XmaI EcoRII
 Bme18I AvaII Van91I Cfr9I BglI BstNI
 AvaII CfrI AvaI BsoBI BstOI

 AccB1I NarI HaeII NdeII
 KasI BbiII EheI Hsp92II Kzo9I
 Eco64I BsaHI BbeI DpnII MaeII HphI
gctctggggcgcccatgcccagaacgcgatcaggcccgaccctcgccaacactacgtcctcaagttttctcaccc base pairs
cgagaccccgcgggtacgggtcttgcgctagtccgggctgggagcggttgtgatgcaggagttcaaaagagtggg 10801 to 10875
 BanI Msp17I Bsp143II Bsp143I
 BshNI Hsp92I NlaIII Sau3AI
 Hin1I AcyI BstH2I MboI DpnI

 BssT1I HgiEI BsmFI Cfr9I
 ErhI Bme18I Eco47I BcoI XmaI
 EcoNI SinI AvaII MaeII HinfI TthHB8I AvaI
gtcgcccctctccaaggtcccgtttgggacgtgccagcatttcctcgccgcgaatcgctacctcgaaacccggga base pairs
cagcggggagaggttccagggcaaaccctgcacggtcgtaaaggagcggcgcttagcgatggagctttgggccct 10876 to 10950
 Eco130I EcoO109I BsmFI TfiI TaqI Ama87I
 StyI PpuMI Psp5II PspAI
 EcoT14I DraII Eco88I

PspALI Bsp143I Bsh1285I BsrI VspI
 MboI DpnI TaqI BseNI Tsp509I MseI
 SmaI DpnII BspCI PvuI Bse1I SfaNI BstF5I Sse9I PshBI
cattatgccgatcgactggtcggtataagatgccgacatccggggtcttgatttacgagggggcaattaataaag base pairs
gtaatacggctagctgaccagccatattctacggctgtaggccccagaactaaatgctcccccgttaattatttc 10951 to 11025
 BsmFI NdeII TthHB8I BsaOI FokI TspEI AseI
BsoBI Sau3AI Ple19I BsrSI AsnI Tru1I
 Kzo9I BsiEI BstMCI Tru9I

 BsoBI AgeI Cfr10I AvaII SinI DraII
 Tru1I Eco88I BsaI BssAI SinI Eco47I Ksp632I EcoO109I
 Tru9I Ama87I Alw26I BsrFI HgiEI MboII PpuMI Eco47I
actgttgatggttaaatctcgggtctcataccggtccgtgatgtcgggcgtgggggaagagagggtcccctctgc base pairs
tgacaactaccaatttagagcccagagtatggccaggcactacagcccgcacccccttctctcccaggggagacg 11026 to 11100
 MseI BcoI Eco31I Bse118I RsrII Eam1104I AvaII
 AvaI BsmAI BsaWI Bme18I EarI HgiEI BsmFI
 PinAI CpoI CspI Bme18I Psp5II

 Bsp143I BstNI HgaI BsaHI
 MboI NlaIII AclWI BbiII
 BsiI MaeII DpnII Hsp92II MvaI Hin1I
gtttactatccttgcctcgtggggctggacgtttgcaccccagaaccatgatcctggcgcgtcgccgaatacgac base pairs
caaatgataggaacggagcaccccgacctgcaaacgtggggtcttggtactaggaccgcgcagcggcttatgctg 11101 to 11175
 BssSI NdeII DpnI BstOI Msp17I
 Sau3AI BstXI Bst2UI Hsp92I
 Kzo9I EcoRII AlwI AcyI

 Eco47I PmlI DraII CspI
 TaqI HgiEI Eco72I TaqI Bme18I BstF5I
 HinfI SinI BsaWI HgaI BbrPI TthHB8I CpoI AvaII
gcccatagagtcgattgcggggaccgcaccggacgcgcacgtggggcctctcgacggagagccggaccgggatgc base pairs
cgggtatctcagctaacgcccctggcgtggcctgcgcgtgcaccccggagagctgcctctcggcctggccctacg 11176 to 11250
 TthHB8I Bme18I MaeII EcoO109I SinI Eco47I
 PleI AvaII BsaAI HgiEI FokI
 HgaI BsmFI PmaCI RsrII

NdeII NgoMI
 DpnI TaqI CfrI Bse118I MaeIII
DpnII Kzo9I MaeII EaeI NgoAIV MaeII HphI
gatctccccgcttacgtcgagcgtggccggcgacccgccgggggcggacggcccctacgtcacctttgatactct base pairs
ctagaggggcgaatgcagctcgcaccggccgctgggcggcccccgcctgccggggatgcagtggaaactatgaga 11251 to 11325
 Sau3AI TthHB8I MroNI Cfr10I Tsp45I
 Bsp143I BssAI NaeI
MboI SfaNI BsrFI

 MboI MboII Ple19I Bse1I AccB1I NarI BstH2I Bsp143I HgiEI BspMI
 DpnII Kzo9I TaqI BsaOI BanI Msp17I Bsp143II Kzo9I Bme18I Eco47I
 TthHB8I BspCI PvuI BseNI BshNI BsaHI HaeII MboI AclWI DraII
gtttatggtatcttcgatcgacgaactggggcgccgccagctcacggatacgatccgtaaggacctgcggctgtc base pairs
caaataccatagaagctagctgcttgaccccgcggcggtcgagtgcctatgctaggcattcctggacgccgacag 11326 to 11400
 TaqI Bsp143I Bsh1285I BsrI Hin1I AcyI AluI Sau3AI SinI AvaII
 NdeII DpnI BsiEI BsrSI KasI BbiII EheI DpnII DpnI PpuMI Psp5II
 Sau3AI TthHB8I BstMCI Eco64I Hsp92I BbeI NdeII AlwI EcoO109I

 BalI XmaIII BsaOI
 MscI AfaI AtsI CfrI BsmFI BssHII
 EaeI SfaNI RsaI Tth111I EaeI Eco52I
gctggccaagttcagcatcgcgtgtaccaagacctcgtcgttttcggggacggccgcgcgccagcgcaagcgcgg base pairs
cgaccggttcaagtcgtagcgcacatggttctggagcagcaaaagcccctgccggcgcgcggtcgcgttcgcgcc 11401 to 11475
 CfrI Csp6I AspI BstZI Bsh1285I
 MluNI EagI BsiEI BsePI
 EclXI BstMCI

 Bbv12I RsaI
 AspHI NspI
 SduI BsiHKAI NlaIII BbvI GsuI BssHII
agcaccgccgcaacgcacatgcgtaccacgcagcaacaagagcctccagatgttcgttttgtgcaagcgcgccaa base pairs
tcgtggcggcgttgcgtgtacgcatggtgcgtcgttgttctcggaggtctacaagcaaaacacgttcgcgcggtt 11476 to 11550
 Bsp1286I Hsp92II Bst71I BpmI BsePI
 BmyI Csp6I
 Alw21I AfaI

 BbvI Eco47I
 NspBII BstMCI HgiEI
 BspMI PvuII BsiEI SinI
cgccgcgcaggtgcgcgagcagctgcgggcggttattcggtcgcgcaagccgcgcaagtattacacgcggtcctc base pairs
gcggcgcgtccacgcgctcgtcgacgcccgccaataagccagcgcgttcggcgcgttcataatgtgcgccaggag 11551 to 11625
 AluI Bsh1285I Bme18I
 MspA1I BsaOI AvaII
 Bst71I

 XmaIII BsmFI RsaI
 CfrI BsiEI BsrGI
 BstF5I EaeI Eco52I SspBI BsiI NlaIII TthHB8I
ggatgggcggctctgcccggccgtccccgtgtttgtacacgagtttgtttcgtccgaacccatgcgcctccatcg base pairs
cctacccgccgagacgggccggcaggggcacaaacatgtgctcaaacaaagcaggcttgggtacgcggaggtagc 11626 to 11700
 FokI BstZI BstMCI Bsp1407I BssSI Hsp92II TaqI
 EagI Bsh1285I Csp6I
 EclXI BsaOI AfaI


 AccI
 MaeII NlaIII DdeI BsmFI
agataacgtcatgctgtctacggaaccagactaagcacccccgccgtcccctttcttttccccctacccttcccc base pairs
tctattgcagtacgacagatgccttggtctgattcgtgggggcggcaggggaaagaaaagggggatgggaagggg 11701 to 11775
 Hsp92II BstDEI


 AfaI BsaAI
 MaeIII Csp6I AflIII AluI BstF5I
cgttactgatgtgttgtacgtttcaataaataacacgtagcttattttgttggatgatggattgattgattttat base pairs
gcaatgactacacaacatgcaaagttatttattgtgcatcgaataaaacaacctactacctaactaactaaaata 11776 to 11850
 RsaI MaeII FokI
 MaeII


 BshNI
 Eco64I BssHII
tgaccgttcgttcgcccggcggtgccgtcgccgcgcgcagagggaatatgcaagcgggcggggtggggaggaaag base pairs
actggcaagcaagcgggccgccacggcagcggcgcgcgtctcccttatacgttcgcccgccccacccctcctttc 11851 to 11925
 BanI BsePI
 AccB1I


 Bst2UI Bsp143I Tsp509I
 BstNI MboI DpnI
 HgaI DpnII Sse9I TthHB8I
aaggtttcaggttccgggggttgggtctgcgtcgtccagggtggggctgatctgaatttcccgcagaacctcgac base pairs
ttccaaagtccaaggcccccaacccagacgcagcaggtcccaccccgactagacttaaagggcgtcttggagctg 11926 to 12000
 EcoRII NdeII AcsI TaqI
 BstOI Sau3AI ApoI
 MvaI Kzo9I TspEI

 DrdI MaeII
 Bse1I BbiII
 BsrSI Hin1I
cagtaggtctgttgtgtttgctgggaactcgcccgccgttggggatacgggggcggggggtgtggtcgggcggac base pairs
gtcatccagacaacacaaacgacccttgagcgggcggcaacccctatgcccccgccccccacaccagcccgcctg 12001 to 12075
 BseNI Msp17I
 BsrI Hsp92I
 BsaHI

 EcoRII BsoBI Sau3AI
 AatII Eco88I NdeII
 AcyI MvaI Ama87I BsmFI Kzo9I HphI
gtccaggggtgcgttatcgcacccccgcgccgcctcgggggccgtcccgtagatcgttgcggtgatgtagatggt base pairs
caggtccccacgcaatagcgtgggggcgcggcggagcccccggcagggcatctagcaacgccactacatctacca 12076 to 12150
 BstOI BcoI DpnII
 Bst2UI AvaI MboI DpnI
 BstNI BglI Bsp143I

 Eco47I FokI NgoAIV XmaIII BspEI BseAI MvaI
 HgiEI BssAI Cfr10I NaeI BstMCI BsaWI BstOI
 SinI BstF5I BstZI EclXI BsaOI Kpn2I AluI EcoRII
gtccggggtccacaccaccgtcaggatgccggccgtcgcactccggacgctttcgccgtgcgatgagctgaccca base pairs
caggccccaggtgtggtggcagtcctacggccggcagcgtgaggcctgcgaaagcggcacgctactcgactgggt 12151 to 12225
 Bme18I MroNI Bse118I Eco52I AccIII HgaI BstNI
 AvaII NgoMI SfaNI EagI Bsh1285I Bsp13I Bst2UI
 BsrFI EaeI CfrI BsiEI MroI BsiMI

 MluI NdeI Hsp92I HaeII AccB7I MaeI
 AfaI AfaI BbiII BsmFI AfeI GsuI Van91I BfaI
 HinfI Csp6I Csp6I Hin1I HgaI Aor51HI BpmI Csp6I HgaI
ggagtcaaaggggtacgcgtacatatgggcgtcccaccagcgctccagcctctgggtactagcgcgtcctataaa base pairs
cctcagtttccccatgcgcatgtatacccgcagggtggtcgcgaggtcggagacccatgatcgcgcaggatattt 12226 to 12300
 PleI RsaI RsaI Msp17I Eco47III AlwNI RsaI
 AflIII FauNDI AcyI Bsp143II Esp1396I
 DrdI BsaHI BstH2I PflMI AfaI

 AcsI
 Acc16I BcgI Bst71I
 FspI ApoI TthHB8I HphI XcmI HphI
gcggtatgcgcaaaattcggcacgacagtcgataatcaccagcagcccgatgggggtgtgttgtatcaccacgcc base pairs
cgccatacgcgttttaagccgtgctgtcagctattagtggtcgtcgggctacccccacacaacatagtggtgcgg 12301 to 12375
 AviII Tsp509I TaqI BbvI
 Sse9I
 TspEI

 KspI SacII EcoRII BsePI AspEI PmlI VneI
 NspBII Bme18I MvaI TaqI EclHKI HinfI Eco72I
 BstDSI Cfr42I Eco47I BssHII AhdI HgaI BssHII HgaI PleI MaeII Alw44I
tccgcggggcaggcggtcctggcgcgctcgaccccgcgtcagaaccgcgcgcgtccctgactcaaacacgtgcac base pairs
aggcgccccgtccgccaggaccgcgcgagctggggcgcagtcttggcgcgcgcagggactgagtttgtgcacgtg 12376 to 12450
 DsaI SstII SinI BstNI TthHB8I BsePI BsmFI AflIII BbrPI
 MspA1I HgiEI BstOI Eam1105I BsaAI
 Sfr303I AvaII Bst2UI PmaCI

 BmyI BsaHI BstOI
 Bsp1286I BbvI BbiII BstNI AfaI
 Alw21I HgaI BssHII BcgI Hin1I EcoRII HphI Csp6I
cacctgtgccgcgtccggcagcgcgctcgttagcgacgccctggggtgatgtaggctgtacgcgatggtcgtctg base pairs
gtggacacggcgcaggccgtcgcgcgagcaatcgctgcgggaccccactacatccgacatgcgctaccagcagac 12451 to 12525
 AspHI BsePI Msp17I Bst2UI RsaI
ApaLI BsiHKAI Bst71I Hsp92I MvaI
SduI Bbv12I AcyI HgaI

 Eco88I Cfr9I SmaI
 Ama87I MaeIII BcoI XmaI
 NlaIII BsoBI HphI HphI AvaI BsoBI
ggggttccccatgtctcgggggggtgggggtgaatgtcacccggcccgggtgcggtgggaacgcgagggaatgga base pairs
ccccaaggggtacagagcccccccacccccacttacagtgggccgggcccacgccacccttgcgctcccttacct 12526 to 12600
 Hsp92II Tsp45I Ama87I
 BcoI Alw26I PspAI PspALI
 AvaI BsmAI Eco88I

 Bsp143I Bbv12I Hsp92I
 Tru1I MboI DpnI AspHI Msp17I
 Tru9I DpnII AclWI SduI BsiHKAI BsaHI
gggttaatagacaatgaccacattcggatcgcgtagagcagatagtatgtgctcgctaatgacgtcatcgcgttc base pairs
cccaattatctgttactggtgtaagcctagcgcatctcgtctatcatacacgagcgattactgcagtagcgcaag 12601 to 12675
 MseI NdeII AlwI Bsp1286I Hin1I AcyI
 Sau3AI BmyI BbiII AatII
 Kzo9I Alw21I MaeII

 SplI SnaBI
 BstH2I BstD102I BsgI Hsp92II Pfl23II
 Bsp143II AccBSI NlaIII BstF5I BsmFI MaeII BstSNI
gtggcgctcccggagcgggtttagattcatgtgcaggaactcggatgaggtggtgcgggacatggctacgtacgc base pairs
caccgcgagggcctcgcccaaatctaagtacacgtccttgagcctactccaccacgccctgtaccgatgcatgcg 12676 to 12750
 HaeII BsrBI HinfI Hsp92II FokI NlaIII PspLI
 TfiI Eco105I
 BsaAI

 AfaI Eco24I MvaI
 RsaI AtsI Bsp1286I
 BspMI FauNDI Tth111I DdeI SduI EcoRII
gctgtttaggcgcaggtttccgggcgtgaagcatatggcgaccttgtccagactgagcccctgggagcgcgtgat base pairs
cgacaaatccgcgtccaaaggcccgcacttcgtataccgctggaacaggtctgactcggggaccctcgcgcacta 12751 to 12825
 SunI NdeI AspI BstDEI BanII Bst2UI
 Csp6I BmyI BstNI
BsiWI FriOI BstOI

 Bse8I BalI NcoI EaeI MluNI BstXI Sau3AI
 BsrBRI MscI BssT1I CfrI AluI TthHB8I Bsp143I
 MamI Bsp68I AluI SfaNI EaeI ErhI BstDSI MscI BstDSI NdeII
ggtcatcgcgagtttggagctgatgccgtagtcggcgttgatggccatggccagctccgtggagtcgatcgactc base pairs
ccagtagcgctcaaacctcgactacggcatcagccgcaactaccggtaccggtcgaggcacctcagctagctgag 12826 to 12900
 BsaBI NruI CfrI StyI DsaI NlaIII DsaI DpnII
 Bsh1365I MluNI EcoT14I Hsp92II TaqI MboI
 Eco130I Bsp19I BalI HinfI PleI

 BsiEI BstMCI Eco47I
 TaqI HinfI HgiEI
 TthHB8I BsaOI TspRI NlaIII SinI BsmFI
gacaaactcactgatgttggtattgacgacagacatgaagccgtgctggtcccgcaggacgatgtagggcagggg base pairs
ctgtttgagtgactacaaccataactgctgtctgtacttcggcacgaccagggcgtcctgctacatcccgtcccc 12901 to 12975
Kzo9I Ple19I TaqI Hsp92II Bme18I
 DpnI Bsh1285I PleI AvaII
 BspCI PvuI TthHB8I

 NgoAIV BstZI Eco52I Eco88I
 PleI NgoMI BglI XmaIII Bst71I PspAI
 HinfI BseRI EaeI MroNI Cfr10I NaeI BsaOI AluI BseRI Ama87I
ggactcctccaagaactcggccacgccggccgtcgcgtgccgccgccgcagctcctccgcgaacgcgaacacccg base pairs
cctgaggaggttcttgagccggtgcggccggcagcgcacggcggcggcgtcgaggaggcgcttgcgcttgtgggc 12976 to 13050
 BsmFI CfrI BssAI SfiI EagI Bsh1285I BbvI BcoI
 BsrFI EaeI EclXI BstMCI Cfr9I
 Bse118I CfrI BsiEI AvaI

 Csp6I Csp6I
 MslI AfaI AfaI PstI Bse118I
 SmaI AflIII MslI SfcI BstDSI BssAI
ggtgtacgtgtaccccatcagcgtgtagttgtccgtctgcagggccacggacatcagccccccgcgcggcgagcc base pairs
ccacatgcacatggggtagtcgcacatcaacaggcagacgtcccggtgcctgtagtcggggggcgcgccgctcgg 13051 to 13125
 XmaI RsaI RsaI BstSFI DsaI BsrFI
 PspALI BsaAI Cfr1
 BsoBI MaeII


 Bst71I AtsI MslI BstH2I
 AluI BbvI Tth111I BsaAI Eco57I Bsp143II
ggtcagcagctcgcagccccggaaaatgacattgtccacgtaggtgctgaagggggcgctctcaaacacctcccc base pairs
ccagtcgtcgagcgtcggggccttttactgtaacaggtgcatccacgacttcccccgcgagagtttgtggagggg 13126 to 13200
 BbvI Bst71I AspI MaeII HaeII
 0I


 Ksp632I SduI Psp124BI Eco57I BanII Bse1I XmaIII
 Eam1104I BmyI SacI BsiHKAI EarI SduI XcmI CfrI BsiEI
 MboII EarI Bbv12I BanII EcoO109I Eam1104I FriOI BsrSI EaeI Eco52I
gaagagctcccgtaggataaggtatcgccccagaaaggccctcttcaggagcccaaactgggcgtggacggccgc base pairs
cttctcgagggcatcctattccatagcggggtctttccgggagaagtcctcgggtttgacccgcacctgccggcg 13201 to 13275
 Ecl136II AspHI FriOI DraII Ksp632I Eco24I BseNI BstZI
 AluI SapI Eco24I Alw21I MboII Bsp1286I EagI Bsh1285I
 EcoICRI Bsp1286I SstI BmyI BsrI EclXI

 BstMCI SstII Alw26I Eam1104I AlwNI
 BsaOI Cfr42I TthHB8I NspBII
 MspA1I BstDEI TaqI MboII AflIII PvuII MboII
ggtggtctcaggctcttcgagggcgtagtggcagtagaacacgtccagctgctgttcgtccagcccggcgaagat base pairs
ccaccagagtccgagaagctcccgcatcaccgtcatcttgtgcaggtcgacgacaagcaggtcgggccgcttcta 13276 to 13350
BstDSI KspI SacII BsmAI EarI MaeII AluI Bst71I
 Sfr303I Eco31I Ksp632I MspA1I
DsaI NspBII DdeI BsaI SapI BbvI

 SduI BanII KspI SacII SduI BanII MseI Tsp5
 EcoO109I ApaI MspA1I PspOMI Eco24I MvaI Sse9I
 Bsp120I FriOI BsmFI Cfr42I BmyI ApaI Tru9I
aacgtcaaggtcgtcgtcggggaagtcgtccgggcccccgtcccgcgggcccaggtgcttaaaattgaacgcacg base pairs
ttgcagttccagcagcagccccttcagcaggcccgggggcagggcgcccgggtccacgaattttaacttgcgtgc 13351 to 13425
 MaeII PspOMI BmyI BstDSI SstII EcoRII BstNI Tru1I
 DraII Eco24I NspBII Bsp120I FriOI BstOI TspEI
 Bsp1286I DsaI Sfr303I Bsp1286I Bst2UI

 09I Bsh1285I MvaI NgoMI Hin1I AcyI
 BstD102I BstOI MroNI Cfr10I BsaHI
 AccBSI EcoRII SfaNI NgoAIV Hsp92I
ctcccccggagagcggtcgctggtgtcggcggccctggttgccgatgcgccggcggcgtcccggcgtagcgacag base pairs
gagggggcctctcgccagcgaccacagccgccgggaccaacggctacgcggccgccgcagggccgcatcgctgtc 13426 to 13500
 BsrBI BsaOI BstNI SgrAI Bse118I HgaI
 BsiEI Bst2UI BssAI NaeI BbiII
 BstMCI BsrFI Msp17I BsmFI

 BlnI BfaI XmaIII EcoRII Bme18I Eco47I Bst2UI
 ErhI EcoT14I BsiEI BstNI PpuMI Psp5II BstNI BstH2I
 AluI AvrII EaeI EclXI BsaOI SinI DraII EcoRII Bsp143II
gagttctgccgtcagctcccctaggcggccgtaggccagggtcctctgggtcgcgtccaggccggggcgctggag base pairs
ctcaagacggcagtcgaggggatccgccggcatccggtcccaggagacccagcgcaggtccggccccgcgacctc 13501 to 13575
 Eco130I CfrI Eco52I BstOI HgiEI HgaI HaeII
 StyI MaeI EagI BstMCI Bst2UI AvaII BstOI
 BssT1I BstZI Bsh1285I MvaI EcoO109I MvaI

BpmI BsrFI
 BssAI
 HinfI NlaIII AgeI Cfr10I
aaagttgtaaaagtgaatcagcccgccgaacatgagccgcgacaggaaccggtaggcgaactccaccgaggtctc base pairs
tttcaacattttcacttagtcgggcggcttgtactcggcgctgtccttggccatccgcttgaggtggctccagag 13576 to 13650
 TfiI Hsp92II PinAI
GsuI BsaWI
 Bse118I

 EcoRII MboII AvaII
BsaI BstOI BbsI Bme18I TaqI
Alw26I MvaI BpuAI AluI BbvI TthHB8I MaeII BsmFI
cccctgggtcttcacgaagctgtcgtcgcgcagcacagcctcgaaggtccgaaacgtcccgtcgaacccaaacac base pairs
ggggacccagaagtgcttcgacagcagcgcgtcgtgtcggagcttccaggctttgcagggcagcttgggtttgtg 13651 to 13725
Eco31I Bbv16II Bst71I TaqI SinI TthHB8I
BsmAI Bst2UI BpiI HgiEI
 BstNI Eco47I

 EcoO65I Bst2UI BsiWI BsaAI
 BstEII BstOI PspLI MaeII
 BssHII Tsp45I EcoRII MaeII Csp6I MaeI
catctttcggaggcgcgcggtcaccgcgacctggctgttgaggacgtacgtgatgtcgttccgggccacgactag base pairs
gtagaaagcctccgcgcgccagtggcgctggaccgacaactcctgcatgcactacagcaaggcccggtgctgatc 13726 to 13800
 BsePI Eco91I BstNI SplI RsaI BfaI
 BstPI MaeIII Pfl23II
 PspEI HphI MvaI SunI AfaI

 Bsp1286I PmaCI BstOI Eco47I
AluI ApaLI Bbv12I BsaAI BmyI BstNI HgiEI
 Alw44I Alw21I MaeII SduI EcoRII Bme18I PleI HphI
ctgttgcttgctgtgcacctcacagcgcacgtgccccgcgtcctggtcctgactctgggagtagttggtgatgcg base pairs
gacaacgaacgacacgtggagtgtcgcgtgcacggggcgcaggaccaggactgagaccctcatcaaccactacgc 13801 to 13875
 VneI BmyI Eco72I HgaI SinI SfaNI
 SduI BsiHKAI PmlI Bsp1286I MvaI AvaII
 AspHI BbrPI Bst2UI HinfI

 EaeI Bsp143I NcoI Bsp19I
 Bse1I MboI DpnI StyI DsaI
 BsrSI DpnII AclWI Eco130I Hsp92II BbvI TthHB8I
actggcgttggccgtgatccacttttccatggtcagcgtgggttgctgcgtgagccgtcgatactcgtcaaactc base pairs
tgaccgcaaccggcactaggtgaaaaggtaccagtcgcacccaacgacgcactcggcagctatgagcagtttgag 13876 to 13950
 BseNI NdeII AlwI ErhI BstDSI Bst71I TaqI
 BsrI Sau3AI BssT1I NlaIII
 CfrI Kzo9I EcoT14I XcmI

 Bbv12I MaeIII
 AspHI HinfI
 MslI SduI BsiHKAI Bsp68I HphI
tttgaccgacacaaacgtgagcacggggagggtaaacacaacaaactccccctcgcgagtcacctttaggtaggc base pairs
aaactggctgtgtttgcactcgtgcccctcccatttgtgttgtttgagggggagcgctcagtggaaatccatccg 13951 to 14025
 MaeII Bsp1286I NruI PleI
 BmyI Tsp45I
 Alw21I

 BalI AfaI
 CfrI Hsp92II
 AluI MscI NlaIII BsmFI HgaI EaeI
gtggagcttggccatgtacgcgctgacctccttgtgggacgagaacagccgcgtccaccccggaaggttggccgg base pairs
cacctcgaaccggtacatgcgcgactggaggaacaccctgctcttgtcggcgcaggtggggccttccaaccggcc 14026 to 14100
 EaeI Csp6I CfrI
 MluNI RsaI


 AvaII Bsp1286I HphI
 Bme18I NspI BmyI BseRI AfaI
 HphI BsmFI DrdI NlaIII Bbv12I Csp6I
gttggtgatgtaactttccgggacgacaaagcggtccacaaactgcatgtgctcctcggtgatgggaaggccgta base pairs
caaccactacattgaaaggccctgctgtttcgccaggtgtttgacgtacacgaggagccactacccttccggcat 14101 to 14175
 MaeIII SinI Hsp92II Alw21I RsaI
 HgiEI SduI BsiHKAI
 Eco47I AspHI

 Hsp92II AspHI
 RcaI SduI BsiHKAI Tru1I NspBII
 GsuI MslI NlaIII BsiI Bbv12I Tru9I PvuII
ctccagcaccttcatgaggttcccgaactcgtgctccacacatcgcttgttgttaatgaaaatggcccagctgtg base pairs
gaggtcgtggaagtactccaagggcttgagcacgaggtgtgtagcgaacaacaattacttttaccgggtcgacac 14176 to 14250
 BpmI BspHI BssSI Alw21I MseI AluI
 EcoNI Bsp1286I MspA1I
 BmyI

 BsaAI Bbv12I
 AfaI AfaI AspHI
 Csp6I Csp6I SduI Alw21I
cgagaggcgcgtgtactcgcgtagggtgcggttgcagatgaggtacgtgagcacgttttcgctctgccggacgga base pairs
gctctccgcgcacatgagcgcatcccacgccaacgtctactccatgcactcgtgcaaaagcgagacggcctgcct 14251 to 14325
 RsaI RsaI Bsp1286I
 MaeII BmyI BsiHKAI
 MaeII

 BstBI Bpu14I
 LspI NspV BpmI Bse118I
 SfaNI TthHB8I HinfI GsuI BssAI
gcatcgcagtttttggtgttcgaaggtggactccagcgaggccgtctgggtcggcgaccccacgcacaccagcac base pairs
cgtagcgtcaaaaaccacaagcttccacctgaggtcgctccggcagacccagccgctggggtgcgtgtggtcgtg 14326 to 14400
 SfuI TaqI PleI BsrFI
 Csp45I Cfr1
 Bsp119I

 EaeI EclXI BsaOI BstMCI
 Eco52I AfaI Bse1I AfaI Tru1I BsiEI
 CfrI Bsh1285I Csp6I BsrSI Csp6I Tru9I BstF5I BstDSI
cggccgcaggcggcccgcgtactggggggtgtggtacagggcgttaatcatccaccagcaatacaccacggtcgt base pairs
gccggcgtccgccgggcgcatgaccccccacaccatgtcccgcaattagtaggtggtcgttatgtggtgccagca 14401 to 14475
 EagI BstMCI RsaI BseNI RsaI MseI FokI DsaI MslI
 0I XmaIII BsrI Bsh1285I
 BstZI BsiEI BsaOI

 MvaI FriOI BsrFI Bst71I
 BshNI BstOI BmyI Bst71I BssAI NaeI BstH2I
 Eco64I EcoRII SduI TthHB8I BcgI HphI Bse118I Bsp143II
gagtaggtgccgccccaggagcccggcctcgtcgatgacgataatgttgctgcgggtgaaagccggcagcgcccc base pairs
ctcatccacggcggggtcctcgggccggagcagctactgctattacaacgacgcccactttcggccgtcgcgggg 14476 to 14550
 BanI BstNI Bsp1286I TaqI BbvI MroNI Cfr10I
 AccB1I Bst2UI Eco24I NgoMI BbvI
 BanII NgoAIV HaeII

 MvaI SduI Bst2UI
 BstOI BshNI EcoRII
 EcoRII Eco64I BstNI MaeI HphI
gtgtgtgaccgaggccaggcgcgtgagggcaccctggcccagccccaaagtctgctctagggcggtgagggcgtg base pairs
cacacactggctccggtccgcgcactcccgtgggaccgggtcggggtttcagacgagatcccgccactcccgcac 14551 to 14625
 Tsp45I BstNI BanI BmyI MvaI BfaI
 MaeIII Bst2UI AccB1I BstOI
 Bsp1286I

 BpiI MvaI PspOMI Eco24I MboI DpnI
 Bbv16II BstOI DraII XcmI TaqI Bsp143I Bse1I
 HgaI EcoRII Bsp120I FriOI TthHB8I Kzo9I BsrSI
gaactcgtttcgcgcgtcttcgcccccgtgcgccgccagggcccgcttggtgatgtcgaggatcacctcccagta base pairs
cttgagcaaagcgcgcagaagcgggggcacgcggcggtcccgggcgaaccactacagctcctagtggagggtcat 14626 to 14700
 BpuAI BstNI EcoO109I BanII DpnII AclWI BseNI
 BbsI Bst2UI Bsp1286I ApaI NdeII AlwI BsrI
 MboII BglI SduI BmyI HphI Sau3AI HphI

 Csp6I NspBII BspMI MscI Bst2UI
 BsaI SfcI PstI BbsI EaeI EcoRII
 MaeII Alw26I BbvI BpuAI BbvI BalI Csp6I BbvI
gtacgtcaggtctcgccgctgcaggtcttccagcgaggcggggctgctggccagggtgtacgggtgctgccccag base pairs
catgcagtccagagcggcgacgtccagaaggtcgctccgccccgacgaccggtcccacatgcccacgacggggtc 14701 to 14775
 Eco31I Bst71I Bbv16II Bst71I BstOI AfaI Bst71I
 AfaI BsmAI BstSFI BpiI CfrI BstNI RsaI
 RsaI MspA1I MboII MluNI MvaI

MspA1I MvaI HincII BsoBI
AluI BstOI AccI DdeI Eco88I
PvuII BstNI HinfI BsiI SalI HinfI Ama87I
ctgggcctggacgtgattcccgcgaaacccgaactcgtgaaagatggtgttgatgggtcgactcagaaacgcccc base pairs
gacccggacctgcactaagggcgctttgggcttgagcactttctaccacaactacccagctgagtctttgcgggg 14776 to 14850
 EcoRII TfiI BssSI TthHB8I PleI BcoI
NspBII Bst2UI TaqI BstDEI AvaI
 MaeII HindII

 BspLU11I AccB1I NarI HaeII EcoRII Bme18I EcoNI
 MseI Csp6I NspI KasI BbiII EheI MaeIII SinI DraII
 AluI MaeII NlaIII HinfI Eco64I BsaHI BbeI DrdI MvaI EcoO109I
cgagagcttaacgtacatgttctgcgccgcgattcgcgtggcgcccgtgaccacgcagtccaggacctcgttgag base pairs
gctctcgaattgcatgtacaagacgcggcgctaagcgcaccgcgggcactggtgcgtcaggtcctggagcaactc 14851 to 14925
 Tru9I RsaI Hsp92II TfiI BanI Msp17I Bsp143II BstNI PpuMI Psp5II
 Tru1I AfaI BshNI Hsp92I Tsp45I BstOI HgiEI Eco47I
 AflIII Hin1I AcyI BstH2I Bst2UI AvaII

 AfaI BsaWI DpnII BamHI DpnI Bse118I
 BsaAI MroI Bsp13I Sau3AI AlwI HphI
 BsgI Csp6I BspEI BsiMI MboI XhoII BsrFI
ggtctgcacgcacgtactctttccggatccggcgttgccggtgatgagatacgccgcgaacggaaactcccggag base pairs
ccagacgtgcgtgcatgagaaaggcctaggccgcaacggccactactctatgcggcgcttgcctttgagggcctc 14926 to 15000
 MaeII AccIII NdeII MflI Kzo9I Cfr10I
 RsaI Kpn2I BstYI Bsp143I BssAI
 BseAI BstX2I BstI AclWI

 BsrFI BsaOI PpuMI Eco47I Msp17I
 BstD102I Bsh1285I AvaII ErhI EcoT14I TspRI BshNI Tsp45I MaeII
 AccBSI BsiEI Bme18I Psp5II BssT1I SfcI Eco64I BspMI Hsp92I
cggcaggccggtcgggacctccaaggccgccacgtcccggaaccactgcaggcgcggcacctgcgtgacgtcgag base pairs
gccgtccggccagccctggaggttccggcggtgcagggccttggtgacgtccgcgccgtggacgcactgcagctc 15001 to 15075
 BsrBI Cfr10I SinI DraII Eco130I BsmFI BstSFI BanI MslI Hin1I AcyI
 BssAI BstMCI HgiEI BsmFI MaeII PstI AccB1I MaeIII BsaHI
 Bse118I BcgI EcoO109I StyI BbiII TthHB8I

AatII Bsp1286I BanII FokI Eco47I TspEI
 AluI AluI BmyI FriOI BstF5I HgiEI MaeII Tru9I
 TaqI BbvI Ecl136II SacI Alw21I SinI NlaIII Sse9I DraI
ctgctgctgcgagagctctcggatgcgtgcgatgattggttggaccccgtgcatggacgtaaaatttaaaaacgc base pairs
gacgacgacgctctcgagagcctacgcacgctactaaccaacctggggcacgtacctgcattttaaatttttgcg 15076 to 15150
 BbvI Bst71I SduI Bbv12I SstI Bme18I Hsp92II Tsp509I
 Bst71I EcoICRI Eco24I BsiHKAI AvaII AcsI MseI
 AspHI Psp124BI SfaNI ApoI Tru1I

 Eco88I Bst71I Eco47I MvaI SduI Msp17I BsmFI
 PspAI PspALI HgiEI BstNI BshNI BbiII AatII
 BsmFI Ama87I BbvI SinI EcoRII BanI Hin1I BsaHI
ctcgtccctgaaccgcacggcgggtctggccccgggctgctgtgggggcggacctggtgcccggacgtcccgcga base pairs
gagcagggacttggcgtgccgcccagaccggggcccgacgacacccccgcctggaccacgggcctgcagggcgct 15151 to 15225
 BcoI XmaI Bme18I Bst2UI Bsp1286I AcyI
 Cfr9I SmaI AvaII BstOI AccB1I Hsp92I
 AvaI BsoBI SexAI Eco64I BmyI MaeII

 BanII BsaHI StyI DsaI CfrI AccB1I NarI MroNI Cfr10I SfaNI
 FriOI BbiII ErhI BstDSI NlaIII KasI BbiII EheI NgoAIV BbeI
 Eco24I Hin1I Eco130I BglI BssHII Eco64I BsaHI NgoMI HaeII NaeI
gccctccccgccggacgccgccatggccgcacagcgcgcgcgggcgccggcgatgcggacgcggggcggcgacgc base pairs
cgggaggggcggcctgcggcggtaccggcgtgtcgcgcgcgcccgcggccgctacgcctgcgccccgccgctgcg 15226 to 15300
 SduI Msp17I BssT1I EaeI BsePI BanI Msp17I SgrAI Bse118I HgaI
 Bsp1286I Hsp92I NcoI Bsp19I BshNI Hsp92I BsrFI BstH2I
 BmyI AcyI HgaI EcoT14I Hsp92II Hin1I AcyI BssAI Bsp143II

 BsoBI Eco88I
 BstH2I Eco88I PspAI PspALI
HgaI Bsp143II Ama87I HphI HphI Ama87I BsmFI HinfI
ggcgctatgcgcccccgaggacggctgggtgaaggttcaccccacccccgggacgatgttgttccgcgagattct base pairs
ccgcgatacgcgggggctcctgccgacccacttccaagtggggtgggggccctgctacaacaaggcgctctaaga 15301 to 15375
 HaeII BcoI BcoI XmaI TfiI
 AvaI Cfr9I SmaI
 AvaI BsoBI

 BsoBI RsaI AvaII
 Eco88I AfaI BsrGI Bme18I
 Ama87I Csp6I SspBI MaeII BsaWI
cctcgggcagatggggtacaccgagggtcagggggtgtacaacgtcgtccggtccagcgaggccgccacccgaca base pairs
ggagcccgtctaccccatgtggctcccagtcccccacatgttgcagcaggccaggtcgctccggcggtgggctgt 15376 to 15450
 BcoI RsaI Bsp1407I SinI
 AvaI Csp6I HgiEI
 BseRI AfaI Eco47I

 SfcI PstI Sau3AI EcoO109I BstNI BpmI AccB1I
 NspBII NdeII RsaI PpuMI Psp5II MvaI Eco64I BsrSI
 PvuII Bst71I Kzo9I BseRI MaeII SinI AvaII BstOI BseRI Hin1I
gctgcaggcggcgatcttccacgcgctcctcaacgccacgacgtaccgggacctggaggaggactggcgccgcca base pairs
cgacgtccgccgctagaaggtgcgcgaggagttgcggtgctgcatggccctggacctcctcctgaccgcggcggt 15451 to 15525
 AluI BbvI DpnII MboII Csp6I Bme18I BsmFI Bst2UI BshNI
 MspA1I MboI DpnI AfaI HgiEI Eco47I EcoNI KasI BbiII
 BstSFI Bsp143I DraII EcoRII GsuI BanI Msp17I

 Hsp92I EheI Eco72I PflMI NspBII SacII BbvI Asp718I BsaWI Eco88I
 BsrI BstH2I BbrPI BstDSI SstII BglI Eco64I Csp6I BcoI XmaI EcoRV
 BsaHI Bsp143II AccB7I DsaI KspI BpmI AlwNI BshNI AfaI PspAI PspALI
cgtggtggcccgcggcctccagccgcagcggctggttcgcaggtaccggaacgcccgggagggcgatatcgccgg base pairs
gcaccaccgggcgccggaggtcggcgtcgccgaccaagcgtccatggccttgcgggccctcccgctatagcggcc 15526 to 15600
BseNI AcyI MaeII PmaCI Van91I Sfr303I MspA1I Acc65I BspMI Cfr9I Eco32I
 Bse1I BbeI BsaAI Esp1396I Cfr42I NspBII BanI RsaI Ama87I BsoBI
 NarI HaeII PmlI DraIII MspA1I GsuI Bst71I AccB1I KpnI AvaI SmaI

 TaqI BsaAI Esp1396I
 CfrI BstD102I Eco72I BstDEI Bst71I PflMI
 BglI AccBSI AflIII BbrPI SfaNI HgaI BstDSI
ggtggccgagcgggtgttcgacacgtggcgatgcacgctcaggacgacgctgctggactttgcccacggggtggt base pairs
ccaccggctcgcccacaagctgtgcaccgctacgtgcgagtcctgctgcgacgacctgaaacgggtgccccacca 15601 to 15675
 EaeI BsrBI MaeII DdeI BbvI DsaI AccB7I
 TthHB8I PmaCI DraIII
 PmlI Van91I

 CspI BsrI
 Bme18I AluI BseNI
AccI CpoI AvaII TthHB8I MaeII
agactgctttgcgccgggcggcccaagcggaccgaccagcttccccaaatatatcgactggctgacgtgtctggg base pairs
tctgacgaaacgcggcccgccgggttcgcctggctggtcgaaggggtttatatagctgaccgactgcacagaccc 15676 to 15750
 SinI Eco47I TaqI BsrSI
 HgiEI Bse1I
 RsrII AflIII

 AlwNI Bst2UI
 Acc16I Bst71I BstNI
 FspI HgaI HgaI Bsp143II DdeI BbvI
gctggttcccatattgcgcaagacgcgcgagggggaggcgacgcagcgcctgggggcgtttctcaggcagcacac base pairs
cgaccaagggtataacgcgttctgcgcgctccccctccgctgcgtcgcggacccccgcaaagagtccgtcgtgtg 15751 to 15825
 AviII BbvI BstH2I BstDEI Bst71I
 EcoRII BstOI
 HaeII MvaI

 BbvI MscI Bsh1285I KspI SacII BsrFI BcoI XmaI DpnII MflI AclWI
 NspBII MluNI BsaOI NspBII BssHII Bse118I AvaI BstYI Sau3AI
 BbvI PvuII CfrI BsiEI BstDSI Cfr42I NgoMI Ama87I BsoBI NdeII DpnI
gctgccccggcagctggccacggtcgccggggccgcggagcgcgccggcccggggcttctggatctggccgtcgc base pairs
cgacggggccgtcgaccggtgccagcggccccggcgcctcgcgcggccgggccccgaagacctagaccggcagcg 15826 to 15900
 Bst71I AluI EaeI DsaI DsaI SstII MroNI Cfr10I Eco88I BstX2I XhoII
 MspA1I BalI BstMCI MspA1I BsePI NgoAIV PspAI PspALI Bsp143I
 Bst71I BstDSI Sfr303I BssAI NaeI Cfr9I SmaI MboI Kzo9I

 Alw44I Bbv12I
AlwI HinfI Hsp92II BstMCI SduI BsiHKAI
 CfrI PleI MslI NlaIII BsiEI ApaLI Alw21I
gttcgactccacgcgcatggcggaatacgaccgcgtgcacatctactacaaccatcgccggggggagtggctggt base pairs
caagctgaggtgcgcgtaccgccttatgctggcgcacgtgtagatgatgttggtagcggcccccctcaccgacca 15901 to 15975
 TthHB8I BstXI Bsh1285I AspHI
 EaeI BsaOI Bsp1286I
 TaqI VneI BmyI

 MvaI Eco47I Bsh1285I
 AtsI MspA1I BstOI BmyI HgiEI Bse118I BstNI
 Tth111I BbvI EcoRII SduI SinI BsrFI BsaOI Bst2UI
gcgcgacccggtcagcgggcagcgcggcgagtgcctggtgctgtgcccccccctgtggaccggcgaccgcctggt base pairs
cgcgctgggccagtcgcccgtcgcgccgctcacggaccacgacacggggggggacacctggccgctggcggacca 15976 to 16050
 AspI NspBII Bst71I BstNI Bsp1286I Bme18I BsiEI BstOI
 Bst2UI AvaII Cfr10I EcoRII
 BssAI BstMCI DrdI

 MvaI BpiI BcoI DpnII DpnI
 TthHB8I AlwNI Bsp1286I Sau3AI
 BbsI TfiI NspBII Ama87I NdeII
cttcgattcgcccgttcagcggctgtgccccgagatcgtcgcgtgccacgccctccgggaacacgcgcacatctg base pairs
gaagctaagcgggcaagtcgccgacacggggctctagcagcgcacggtgcgggaggcccttgtgcgcgtgtagac 16051 to 16125
 BpuAI HinfI MspA1I SduI AvaI Bsp143I
 TaqI MboII BmyI BsoBI Kzo9I
 Bbv16II Eco88I MboI

 AccB1I
 Acc16I KasI
 FspI HgaI Eco64I
ccgtctgcgcaacaccgcgtccgtcaaggtgctgttggggcgcaagagcgacagcgagcgcggggtggctggcgc base pairs
ggcagacgcgttgtggcgcaggcagttccacgacaaccccgcgttctcgctgtcgctcgcgccccaccgaccgcg 16126 to 16200
 AviII BanI
 BshNI
 Hin1I

 NarI HaeII MroNI Cfr10I EclXI BsaOI Esp3I
 BbiII EheI BstH2I BsmBI NgoAIV EaeI Eco52I Alw26I
 BsaHI BbeI Bsp143II BstF5I Alw26I BsrFI BglI XmaIII HgaI BsiI
cgcgcgggtcgtcaataaggcgctgggggaggatgacgagacgaaggccggctcggccgcctcgcgtctcgtgcg base pairs
gcgcgcccagcagttattccgcgaccccctcctactgctctgcttccggccgagccggcggagcgcagagcacgc 16201 to 16275
 Msp17I Bsp143II HaeII FokI BsmAI NgoMI SfiI EagI BstMCI BsmBI
 Hsp92I Esp3I Bse118I BstZI BsiEI BsmAI
 AcyI BstH2I BssAI NaeI CfrI Bsh1285I BssSI

 NspI BsaAI
 Hsp92II BbrPI AfaI
 MslI NlaIII PaeI MaeII BglI Csp6I
gctcatcatcaacatgaagggcatgcgccacgtgggcgacatcaacgacacggtacgcgcctacttggacgaggc base pairs
cgagtagtagttgtacttcccgtacgcggtgcacccgctgtagttgctgtgccatgcgcggatgaacctgctccg 16276 to 16350
 Hsp92II NlaIII PmaCI RsaI
 BbuI Eco72I
 SphI PmlI

 SduI Sau3AI HincII Bst2UI BssAI CfrI
 BshNI DpnII Kzo9I AccI BstNI BshNI Cfr10I
 Eco64I NdeII TaqI SalI XcmI Eco64I EaeI XmaIII
gggggggcacctgatcgacacccccgccgtcgaccacaccctccctgggttcggcaagggcggcaccggccgcgg base pairs
cccccccgtggactagctgtgggggcggcagctggtgtgggagggacccaagccgttcccgccgtggccggcgcc 16351 to 16425
 BanI BmyI Bsp143I TthHB8I EcoRII BanI Bse118I
 AccB1I MboI DpnI TaqI BstOI AccB1I BstZI
 Bsp1286I TthHB8I HindII MvaI BsrFI EagI

 Bsh1285I KspI EcoRII PpuMI Psp5II Eco88I BstMCI EcoRII XmaIII DsaI HindII
Eco52I NspBII SacII SinI DraII BcoI XmaI Bsh1285I BstOI CfrI BsiEI BsaOI
 DsaI Sfr303I Bst2UI AvaII PspAI PspALI BbvI BstNI EaeI Eco52I HincII
gtcgcgcccccaggacccgggggcgcgaccgcagcagcttcgccaggcgtttcagacggccgtggtcaacaacat base pairs
cagcgcgggggtcctgggcccccgcgctggcgtcgtcgaagcggtccgcaaagtctgccggcaccagttgttgta 16426 to 16500
EclXI BstMCI Cfr42I MvaI EcoO109I Cfr9I SmaI Bst71I MvaI BstZI BstDSI
BsiEI BsaOI SstII BstOI HgiEI Ama87I BsoBI BsaOI BcgI EagI Bsh1285I
 BstDSI MspA1I BstNI Bme18I Eco47I AvaI BsiEI AluI Bst2UI EclXI BstMCI

 NspI
 Hsp92II BsmBI
 PaeI GsuI Alw26I
caacggcatgctggagggctatatcaataatctctttggaaccatagaacgcctgcgagagacgaacgcgggtct base pairs
gttgccgtacgacctcccgatatagttattagagaaaccttggtatcttgcggacgctctctgcttgcgcccaga 16501 to 16575
 NlaIII BsmAI
 BbuI BpmI Esp3I
 SphI

 SfcI Bst71I BsePI MvaI BpmI
 NspBII PstI BstMCI Bst71I BstOI BstH2I BsrBI BsePI
 PvuII BcgI BsiEI AluI AscI EcoRII Bsp143II BssHII
ggcgacccagctgcaggcgcgcgaccgcgagctgcggcgcgcccaggcgggggcgctggagcgggagcagcgcgc base pairs
ccgctgggtcgacgtccgcgcgctggcgctcgacgccgcgcgggtccgcccccgcgacctcgccctcgtcgcgcg 16576 to 16650
 AluI BbvI Bsh1285I BbvI BstNI HaeII AccBSI BbvI
 MspA1I BssHII BsaOI BssHII Bst2UI GsuI Bst71I
 BstSFI BsePI BstD102I

 CspI CfrI BsiEI MboI Bsp143I
 Bme18I EaeI Eco52I BstX2I DpnI
 CpoI AvaII XmaIII BsaOI BstYI MflI AlwI
ggcggaccgggcggccgggggaggcgcgggccgcccggcggaggcggatcttctccgggccgactacgacattat base pairs
ccgcctggcccgccggccccctccgcgcccggcgggccgcctccgcctagaagaggcccggctgatgctgtaata 16651 to 16725
 SinI Eco47I EclXI DpnII XhoII MboII
 HgiEI BstZI BstMCI NdeII Kzo9I
 RsrII EagI Bsh1285I Sau3AI AclWI

 BbiII AatII NcoI Bsp19I BsaAI MslI MscI Bse1I Pfl23II
 Hin1I AcyI StyI DsaI MaeII SunI EaeI BseNI FokI SunI
TthHB8I BsaHI Eco130I Hsp92II Pfl23II AfaI BalI Csp6I AfaI SplI RsaI
cgacgtcagcaagtccatggacgacgacacgtacgtggccaacagtttccagcaccagtacatccccgcgtacgg base pairs
gctgcagtcgttcaggtacctgctgctgtgcatgcaccggttgtcaaaggtcgtggtcatgtaggggcgcatgcc 16726 to 16800
TaqI MaeII ErhI BstDSI AflIII BsiWI MaeII BsrSI BstF5I Csp6I
 Msp17I BssT1I NlaIII SplI Csp6I CfrI RsaI PspLI
 Hsp92I EcoT14I PspLI RsaI MluNI BsrI BsiWI

 EcoRII PpuMI Psp5II Eco88I BstH2I BmyI BsiI
AfaI Bst2UI AvaII XhoI PaeR7I Bsp1286I TfiI
 BstOI HgiEI Eco47I AvaI Bsp143II Alw21I BbvI HinfI
ccaggacctcgagcgcctgtcgcgcctctgggagcacgagctggtgcgctgcttcaagattctgcgccaccgcaa base pairs
ggtcctggagctcgcggacagcgcggagaccctcgtgctcgaccacgcgacgaagttctaagacgcggtggcgtt 16801 to 16875
 BstNI Bme18I Sfr274I TthHB8I SduI BsiHKAI Bst71I
EaeI MvaI EcoO109I BcoI TaqI HaeII AspHI BssSI
CfrI SinI DraII Ama87I BsoBI Bbv12I AluI

 MvaI NdeII RsaI Bsp143I Bsh1285I
 BstOI TaqI Bsp143I MaeI MboI DpnI PvuI Csp6I
 EcoRII MaeII Sau3AI AfaI DpnII BspCI BsaOI TthHB8I
caagcagggccaggaaacgtcgatctcgtactctagcggggcgatcgcctccttcgtggccccgtatttcgagta base pairs
gttcgtcccggtcctttgcagctagagcatgagatcgccccgctagcggaggaagcaccggggcataaagctcat 16876 to 16950
 BstNI TthHB8I Csp6I NdeII BsiEI TaqI RsaI
 Bst2UI DpnII DpnI BfaI Sau3AI Ple19I AfaI
 MboI Kzo9I Kzo9I SgfI BstMCI

MaeII BsoBI BsePI HphI BlnI FokI
 Eco88I BstD102I Bse118I ErhI EcoT14I
 Ama87I AccBSI BssAI AvrII MaeI BseRI
cgtgcttcgcgccccccgagcgggcgcgctcatcaccggctccgatgtcatcctaggggaggaggagttatggga base pairs
gcacgaagcgcggggggctcgcccgcgcgagtagtggccgaggctacagtaggatcccctcctcctcaataccct 16951 to 17025
 BcoI BsrBI BsrFI Eco130I EcoNI
 AvaI BssHII Cfr10I StyI BstF5I
 BsaAI BssT1I BfaI

 Hsp92I
 Tru1I PstI RsaI Msp17I RsaI
 Tru9I SfcI MaeII PshAI BsaHI MaeII
ggcggtctttaagaaaacccgcctgcagacgtacctgacagacgtcgcggccctgttcgtggcggacgtacagca base pairs
ccgccagaaattcttttgggcggacgtctgcatggactgtctgcagcgccgggacaagcaccgcctgcatgtcgt 17026 to 17100
 MseI BstSFI Csp6I Hin1I AcyI Csp6I
 AfaI BbiII AatII AfaI
 MaeII

 NspBII SinI AvaII
 DsaI Sfr303I HgiEI
 HgaI KspI SacII EcoO1
cgcggctctgccccggcccccctccccaacccccgccgatttccgggcgagcgcgtccccgcggggcgggtcccg base pairs
gcgccgagacggggccggggggaggggttgggggcggctaaaggcccgctcgcgcaggggcgccccgcccagggc 17101 to 17175
 BstDSI Cfr42I DraII
 BsmFI SstII PpuMI
 MspA1I Bme18I

 Bme18I Bme18I AvaII Bsp143I Ama87I XmaI Eco47I BsaHI
 BsmFI BsmFI SinI Eco47I DpnI Bsh1285I BsoBI HgiEI BstOI BbiII
 09I HgiEI HgiEI HgiEI Sau3AI BsiEI PspAI PspALI SinI BstNI Hin1I
gtcccggacccggacccgatcccggtcgcccgggagaacgccgaggggtgcgccggaccagggctggggcgtcga base pairs
cagggcctgggcctgggctagggccagcgggccctcttgcggctccccacgcggcctggtcccgaccccgcagct 17176 to 17250
 SinI Eco47I Bme18I MboI AlwI BsaOI Eco88I Bme18I MvaI Msp17I
 Eco47I SinI AvaII DpnII Kzo9I BstMCI AvaI SmaI AvaII Bst2UI Hsp92I
 Psp5II AvaII Eco47I NdeII AclWI BcoI Cfr9I EcoRII AcyI TthHB8I

 XmaIII Eco47I
HgaI BstF5I CfrI BsiEI EcoNI HgiEI
TaqI EaeI EaeI Eco52I BstF5I SinI
acgcagggatggccgaccccacgcccgccgatgagggaacggccgccgccatcctcaaacaggccatcgccgggg base pairs
tgcgtccctaccggctggggtgcgggcggctactcccttgccggcggcggtaggagtttgtccggtagcggcccc 17251 to 17325
 CfrI BstZI BstMCI FokI Bme18I
 FokI EagI Bsh1285I AvaII
 EclXI BsaOI

 Bsp143I Bst2UI BbvI MvaI
 BcgI MboI DpnI BstOI HaeII AviII BstOI
 PshAI TthHB8I DpnII AclWI AlwNI FspI NlaIII EcoRII
accgcagtctggtcgaggtggcggaggggatcagcaaccaggcgctgctgcgcatggcctgcgaggtgcgccagg base pairs
tggcgtcagaccagctccaccgcctcccctagtcgttggtccgcgacgacgcgtaccggacgctccacgcggtcc 17326 to 17400
 TaqI NdeII AlwI BstNI Bsp143II Hsp92II BstNI
 Sau3AI EcoRII BstH2I Acc16I Bst2UI
 BsmFI Kzo9I MvaI Bst71I

 Bsp143I Bsh1285I MspA1I BbiII Tsp45I BstOI
 MboI DpnI PvuI NspBII SacII Hin1I AcyI BstNI
 DpnII BspCI BsaOI KspI PshAI HincII MaeII HphI
tcagcgatcgccagccgcggtttaccgcgaccagcgtcctgcgcgttgacgtcacccccagggggcggttgcggt base pairs
agtcgctagcggtcggcgccaaatggcgctggtcgcaggacgcgcaactgcagtgggggtcccccgccaacgcca 17401 to 17475
 NdeII BsiEI BstDSI SstII HgaI HindII BsaHI EcoRII
 Sau3AI Ple19I DsaI Cfr42I Msp17I MaeIII MvaI
 Kzo9I SgfI BstMCI Sfr303I Hsp92I AatII Bst2UI

 PspLI AfaI Hsp92I AfeI BbvI AvaII
 SplI Csp6I BbiII Tru1I HaeII SinI BsmFI
 AflIII SunI Hin1I HgaI Tru9I Bsp143II Eco47I
tcgttctggacgggagttccgacgacgcgtacgtggcgtcggaggattactttaagcgctgcggggaccagccga base pairs
agcaagacctgccctcaaggctgctgcgcatgcaccgcagcctcctaatgaaattcgcgacgcccctggtcggct 17476 to 17550
 MluI BsiWI MaeII BsaHI MseI Aor51HI Bst71I
 HgaI RsaI Msp17I Eco47III Bme18I
 Pfl23II BsaAI AcyI BstH2I HgiEI

 Eco47I BbrPI AspHI EcoRII CfrI
 BstMCI HgiEI Eco72I Bsp1286I BstOI Bsp1286I
MaeII BsiEI EaeI SinI MaeII Alw44I Bbv12I Bst2UI
cgtatcgcggttttgcggtcgtcgtcctcacggccaacgaggaccacgtgcacagcctggccgtgccccccctcg base pairs
gcatagcgccaaaacgccagcagcaggagtgccggttgctcctggtgcacgtgtcggaccggcacgggggggagc 17551 to 17625
 Bsh1285I CfrI Bme18I PmaCI SduI Alw21I MvaI BmyI
 BsaOI AvaII BsaAI ApaLI BsiHKAI EaeI
 PmlI VneI BmyI BstNI SduI

 Bse118I HgiEI EcoICRI Eco24I BsiHKAI
 BssAI SinI TaqI SduI Bbv12I Alw21I
 BbvI BsgI BstDSI BsmFI Ecl136II Psp124BI Csp6I
ttctgctgcaccggctctccttgtttcgccccacggacctccgggacttcgagctcgtctgcctgctgatgtacc base pairs
aagacgacgtggccgagaggaacaaagcggggtgcctggaggccctgaagctcgagcagacggacgactacatgg 17626 to 17700
 Bst71I DsaI Eco47I TthHB8I AspHI SacI SstI EcoRII
 BsrFI Bme18I AluI BmyI FriOI RsaI
 Cfr10I AvaII Bsp1286I BanII AfaI

 BstOI
BstNI
 Bst2UI BsmFI
tggagaactgtccccggagccacgccacgccctcgctgttcgtcaaggtgtcggcgtggttgggggtcgtggccc base pairs
acctcttgacaggggcctcggtgcggtgcgggagcgacaagcagttccacagccgcaccaacccccagcaccggg 17701 to 17775
 GsuI
 MvaI
 BpmI

 Esp3I MspA1I MboI Bsp143I BsrI
 BsmAI MspA1I AluI BstX2I BstI Bse1I
 AflIII Alw26I HgaI BbvI BseRI BbvI BstYI TspRI BsrSI AlwI
gccacgcgtctcccttcgagcgcgtccgctgccttctcctccgcagctgccactggatcctgaacacgctaatgt base pairs
cggtgcgcagagggaagctcgcgcaggcgacggaagaggaggcgtcgacggtgacctaggacttgtgcgattaca 17776 to 17850
 MluI HgaI TthHB8I NspBII PvuII DpnII BamHI BseNI MslI
 BsmBI Bst71I NspBII NdeII MflI Kzo9I AclWI
 TaqI Bst71I Sau3AI XhoII DpnI

 Bse1I MluNI
 TthHB8I BfaI BseNI Hsp92II CfrI
 NlaIII Asp700I AluI TspRI RsaI BspMI
gcatggcgggcgtgaagcccttcgacgacgagctagtcctgccccactggtacatggcccactacctgctggcca base pairs
cgtaccgcccgcacttcgggaagctgctgctcgatcaggacggggtgaccatgtaccgggtgatggacgaccggt 17851 to 17925
 Hsp92II XmnI MaeI BsrSI NlaIII EaeI
 TaqI DrdI Csp6I MscI
 BsrI AfaI BalI

 Bsp1286I BanII BcoI AvaI
 EcoICRI Eco24I BsiHKAI XmaI
 Ecl136II SacI Alw21I Eco88I
acaatccgccccccgtcctctcggccctgttttgcgccaccccgcagagctctgcgttgcagttgcccgggcccg base pairs
tgttaggcggggggcaggagagccgggacaaaacgcggtggggcgtctcgagacgcaacgtcaacgggcccgggc 17926 to 18000
 AluI BmyI FriOI Ama87I PspALI
 SduI Bbv12I SstI Cfr9I SrfI
 AspHI Psp124BI PspAI BsoBI

 SmaI Eco24I XmaIII NgoAIV BsaOI Hsp92I BbvI EcoRI StyI HgiEI
 PspOMI BanII CfrI MroNI Bse118I Msp17I Hsp92II ApoI EcoT14I
 Bsp120I ApaI EaeI Eco52I BsiEI NaeI AcyI Bst71I Tsp509I Bme18I
tcccccgcacggactgtgtggcctataacccggccggcgtcatgggaagctgctggaattccaaggacctgcgtt base pairs
agggggcgtgcctgacacaccggatattgggccggccgcagtacccttcgacgaccttaaggttcctggacgcaa 18001 to 18075
 BmyI BsmFI BstZI NgoMI Bsh1285I BsaHI AluI AcsI ErhI SinI
 Bsp1286I EagI BssAI Cfr10I Hin1I HgaI Sse9I Eco130I PpuMI
 SduI FriOI EclXI BsrFI BstMCI BbiII NlaIII TspEI BssT1I

 Eco47I FriOI Eco47I
 AvaII BmyI HgiEI AtsI
 BspMI SduI SinI AspI
cggctctggtgtattggtggctttcggggagccccaaacgacggacctcgtcgcttttctatcggttttgctaac base pairs
gccgagaccacataaccaccgaaagcccctcggggtttgctgcctggagcagcgaaaagatagccaaaacgattg 18076 to 18150
 DraII Bsp1286I Bme18I
 Psp5II Eco24I AvaII
 EcoO109I BanII Tth111I

 BsaWI BsoBI
 MroI Bsp13I Eco88I
 BspEI BsiMI MaeII MaeII Ama87I BstF5I
tccggaaaataaacgtgttttttatggaacgttccccacctgtcgtgtcatctctcgggggatggtggtgggcct base pairs
aggccttttatttgcacaaaaaataccttgcaaggggtggacagcacagtagagagccccctaccaccacccgga 18151 to 18225
 AccIII AflIII Psp1406I BcoI FokI
 Kpn2I AvaI
 BseAI

 Bsp1286I
 ApaLI Bbv12I
 Alw44I Alw21I NlaIII
gtgtgtgtgtcttgtgcaccgaaggaggaaagtgggggggtggtggtgctggtggtggaaagacatgatagaggg base pairs
cacacacacagaacacgtggcttcctcctttcacccccccaccaccacgaccaccacctttctgtactatctccc 18226 to 18300
 VneI BmyI Hsp92II
 SduI BsiHKAI
 AspHI


 Bse118I Bse1I HgaI
 MboII BssAI BsrSI BssHII
aacaaagaaatagaagaaaaccacaaccggcgcgtgccagtaaatacggacgcgcgcacacgcggggggtaagtt base pairs
ttgtttctttatcttcttttggtgttggccgcgcacggtcatttatgcctgcgcgcgtgtgcgccccccattcaa 18301 to 18375
 BsrFI BseNI BsePI
 Cfr10I BsrI


 Bbv12I EcoO109I ApaI Tsp509I
 AspHI PspOMI BmyI TspEI Asp700I AfaI
 SduI BsiHKAI Bsp1286I Sse9I HinfI TthHB8I Csp6I
ggagcacggggccccggtttattgaccaaattcagggaaacagaaaccgaatcttttcatcgaaagggtacacaa base pairs
cctcgtgccccggggccaaataactggtttaagtccctttgtctttggcttagaaaagtagctttcccatgtgtt 18376 to 18450
 Bsp1286I SduI BanII AcsI TfiI TaqI RsaI
 BmyI Bsp120I Eco24I ApoI XmnI
 Alw21I DraII FriOI

 HinfI MboI Bsp143I
 Bse1I BstX2I DpnI
 AluI BsrSI BstYI MflI
agctcccgccctcgccccacacgccttccagaacccccgtaaacaccagttgaatctcgcgcaggatctcgcgca base pairs
tcgagggcgggagcggggtgtgcggaaggtcttgggggcatttgtggtcaacttagagcgcgtcctagagcgcgt 18451 to 18525
 BseNI DpnII XhoII
 BsrI NdeII Kzo9I
 TfiI Sau3AI AclWI

 Bbv12I StyI DsaI SstII AfaI Sau3AI BstDSI BsaHI
 AspHI ErhI BstDSI Cfr42I DpnII XhoII AclWI AcyI
AlwI HphI BstDSI SduI BsiHKAI NspBII Csp6I MboI Bsp143I Msp17I
ggtgatgggcgcagtccacgggggggagcaccaagggccgcgggtacagatccacggggacgccgaccgactccc base pairs
ccactacccgcgtcaggtgccccccctcgtggttcccggcgcccatgtctaggtgcccctgcggctggctgaggg 18526 to 18600
 BspMI DsaI Bsp1286I EcoT14I Sfr303I BstYI MflI DsaI BbiII
 BmyI Eco130I MspA1I RsaI BstX2I DpnI AlwI Hsp92I
 Alw21I BssT1I KspI SacII NdeII Kzo9I Hin1I BsmFI

 BstMCI PspAI PspALI GsuI AccB1I NarI HaeII MspA1I
 Bsh1285I AvaI BsmFI HgaI BsmBI BseNI KasI BbiII EheI DsaI
 HgaI PleI Eco88I AflIII Alw26I BsrI HgaI Eco64I BsaHI BbeI NspBII
cgcccccgggacatacgcgcacgacgcgtctccagtattgctccgcgtccagcagggcgcctccgcggaaggccg base pairs
gcgggggccctgtatgcgcgtgctgcgcagaggtcataacgaggcgcaggtcgtcccgcggaggcgccttccggc 18601 to 18675
 BsiEI Ama87I XmaI MluI BsmAI BsrSI BanI Msp17I Bsp143II
 BsaOI BcoI BsoBI Esp3I Bse1I BshNI Hsp92I BstDSI
 HinfI Cfr9I SmaI BpmI Hin1I AcyI BstH2I

 MvaI BseNI ScaI Hsp92I
 Cfr42I BstOI BpmI Bse1I Msp17I
 EcoRII Csp6I BsrI HgaI BsaHI EcoNI
tttggggcagggggtcgtcggcctcgcctgggggggtcagaacgctccagtactccgcgtccagacgcctcccga base pairs
aaaccccgtcccccagcagccggagcggacccccccagtcttgcgaggtcatgaggcgcaggtctgcggagggct 18676 to 18750
 SstII BstNI GsuI RsaI Hin1I HgaI
 Sfr303I Bst2UI BsrSI AfaI BbiII
KspI SacII Acc113I Eco255I AcyI

 BstOI BsaHI BbiII HgaI Eco88I NgoAIV
 BstF5I DrdI BbiII Hin1I AcyI Cfr9I NgoMI
 EcoRII Bst2UI Hin1I NlaIII BsaHI AvaI SmaI BseRI MroNI
aggcatccaggacaaagcggtcacaggcgtcctccatgacgccccgggccgcgcacacggcctcctccggcgggc base pairs
tccgtaggtcctgtttcgccagtgtccgcaggaggtactgcggggcccggcgcgtgtgccggaggaggccgcccg 18751 to 18825
 SfaNI MvaI Tsp45I Msp17I Hsp92II Ama87I PspALI BssAI
 BstNI MaeIII Hsp92I Msp17I BcoI XmaI BsrFI
 FokI AcyI HgaI Hsp92I PspAI BsoBI Bse118I

 CfrI MroNI Bse118I HinfI BsmBI XmaIII BstDSI Sfr303I BsiWI
 EaeI EclXI BsrFI BstMCI DdeI CfrI BsiEI DsaI SstII Pfl23II
 Cfr10I BssAI Cfr10I FseI Alw26I HgaI EaeI Eco52I BglI Cfr42I SunI
cggcggccggccgccggaggattcgtctcagcgcgtcgcgcataacctcggccgccgcggcgtacgcggccccgc base pairs
gccgccggccggcggcctcctaagcagagtcgcgcagcgcgtattggagccggcggcgccgcatgcgccggggcg 18826 to 18900
 NaeI XmaIII NgoAIV BsaOI BsmAI BstZI BstMCI MspA1I PspLI
 BstZI Eco52I BsiEI NaeI BstDEI EagI Bsh1285I KspI SacII RsaI
 EagI NgoMI Bsh1285I TfiI Esp3I EclXI BsaOI NspBII SplI Csp6I

 BstDSI Cfr42I SbfI MvaI BpmI
 NspBII Sse8387I BstOI
 DsaI SstII SfcI EcoRII Tsp45I
ggagaggaaatccctgcaggaagtcggtgtcatcgcgggagttccagaaccacgccccggtctggctccaggtga base pairs
cctctcctttagggacgtccttcagccacagtagcgccctcaaggtcttggtgcggggccagaccgaggtccact 18901 to 18975
AfaI Sfr303I BstSFI BstNI
 MspA1I PstI Bst2UI
 KspI SacII GsuI

 Hsp92I BshNI Hsp92I BbeI BstOI
HphI Msp17I BanI Msp17I EcoRII MvaI AfaI
 AccI AcyI Eco64I BsaHI HaeII Bst2UI EaeI Csp6I
cgacgtgggtgtagacgccctctggcgccagggagggggcgaggcgcgggcgtatgccgttggccgaaagtacgg base pairs
gctgcacccacatctgcgggagaccgcggtccctcccccgctccgcgcccgcatacggcaaccggctttcatgcc 18976 to 19050
MaeIII Hin1I HgaI Hin1I AcyI BstH2I CfrI RsaI
 MaeII BbiII KasI BbiII EheI BstNI
 BsaHI AccB1I NarI Bsp143II

BsePI BsaHI XhoI PaeR7I EcoO109I ApaI AcyI Bst2UI Kzo9I Msp17I Ama87I BsoBI
 BbiII Ama87I TthHB8I Bsp1286I Msp17I BstNI Sau3AI Hin1I HgaI Eco88I
 Hin1I Sfr274I TaqI DraII FriOI BbiII HgaI NdeII AclWI AcyI BcoI XmaI
cgcgcacggacgcctcgagggcccggcgggcgtcctggatcgcgccgtgcgcggcgtccgcgtccccggggtcca base pairs
gcgcgtgcctgcggagctcccgggccgcccgcaggacctagcgcggcacgcgccgcaggcgcaggggccccaggt 19051 to 19125
 Msp17I BcoI BsoBI PspOMI BanII Hsp92I MvaI MboI AlwI BsaHI PspAI
BssHII Hsp92I Eco88I BglI SduI Eco24I BsaHI BstOI Bsp143I BbiII HgaI AvaI
 AcyI HgaI AvaI Bsp120I BmyI Hin1I EcoRII DpnII DpnI Hsp92I Cfr9I

 HgiEI BshNI MboI
 SinI Eco64I CfrI DpnII
 Bme18I BbvI HphI EaeI TthHB8I
cgttgaacagcccccagaacgcagccccggtgccgccgcagaccgcaaacttcaccgagctggccgtctgctcga base pairs
gcaacttgtcgggggtcttgcgtcggggccacggcggcgtctggcgtttgaagtggctcgaccggcagacgagct 19126 to 19200
BsmFI AvaII Bst71I AluI TaqI
 SmaI Eco47I BanI NdeII
PspALI MaeII AccB1I Sau3AI

 Kzo9I BbvI BsaHI AccB1I
 DpnI NspBII BbiII BanI
 BstSFI EaeI NlaIII PvuII Hin1I AflIII Eco64I
tctgcaggcagacggcggccatgaccccgccgagcagctgccggagcgcggggcaggcgtcgcacgcgtccggca base pairs
agacgtccgtctgccgccggtactggggcggctcgtcgacggcctcgcgccccgtccgcagcgtgcgcaggccgt 19201 to 19275
 Bsp143I CfrI Hsp92II AluI Msp17I MluI HgaI
 SfcI MspA1I Hsp92I BshNI
 PstI Bst71I AcyI HgaI

 BstH2I Eco88I Bsp120I Eco24I Bst2UI BstZI EclXI HgaI MvaI
 BstNI HaeII PspAI PspALI BmyI ApaI Bsp1286I CfrI Eco52I BstOI
 MvaI GsuI Ama87I SmaI Bsp1286I BstOI Eco24I EagI BsiEI EcoRII
ccaggcgctccagcacggcccgggcccagggctccgagggggcggccgccaccagcgcgtccagcctttccaggc base pairs
ggtccgcgaggtcgtgccgggcccgggtcccgaggctcccccgccggcggtggtcgcgcaggtcggaaaggtccg 19276 to 19350
EcoRII Bsp143II BcoI XmaI PspOMI BanII SduI BanII NotI Bsh1285I BstNI
 Bst2UI BpmI Cfr9I SrfI EcoRII BstNI BmyI EaeI XmaIII BsaOI Bst2UI
 BstOI AvaI BsoBI SduI FriOI MvaI FriOI CciNI BstMCI

 Eco88I AvaI BstOI Esp1396I GsuI
 PspAI PspALI EcoNI EcoRII AccB7I BstOI
 Ama87I BbvI Ama87I EaeI MvaI PflMI BstNI BpmI
ccgcccgcccccgggcttccggcagcccggcctccccgaggcccgcgagggcggccaggagctgggcctggagcc base pairs
ggcgggcgggggcccgaaggccgtcgggccggaggggctccgggcgctcccgccggtcctcgacccggacctcgg 19351 to 19425
 BcoI XmaI Bst71I BcoI CfrI AlwNI EcoRII
 Cfr9I SmaI Eco88I BstNI AluI Bst2UI
 AvaI BsoBI BsoBI Bst2UI Van91I MvaI

 FriOI XmaIII
Bsp1286I Bse118I CfrI BsiEI CfrI
 BanII BssAI EaeI Eco52I TthHB8I BstF5I BstDSI
cggagaaacaaaaccgcgccgtccagaccggcccgacggccgccggggggtcgagtagttggatggtggtggccg base pairs
gcctctttgttttggcgcggcaggtctggccgggctgccggcggccccccagctcatcaacctaccaccaccggc 19426 to 19500
SduI BsrFI BstZI BstMCI TaqI FokI EaeI
BmyI Cfr10I EagI Bsh1285I DsaI
Eco24I EclXI BsaOI

 AccB1I XmaIII NgoAIV BsaOI DsaI XmaIII
 BanI BstMCI CfrI MroNI Bse118I BglI EagI NgoMI
 BsiEI EaeI Eco52I BsiEI NaeI BstZI BssAI
tggggtgccaccgcgcgaccgcttcccgaaaggcgggcaggaggcggccggccgcctccgaggccacggccggcc base pairs
accccacggtggcgcgctggcgaagggctttccgcccgtcctccgccggccggcggaggctccggtgccggccgg 19501 to 19575
 Eco64I Bsh1285I BstZI NgoMI Bsh1285I EaeI Eco52I
 BshNI BsaOI EagI BssAI Cfr10I FseI CfrI MroNI
 EclXI BsrFI BstMCI BstDSI EclXI

 BsrFI BstMCI BstDSI Cfr42I Eco64I Hsp92I HaeII NspBII EcoRII DraII MvaI DsaI
 Bsh1285I Hsp92II SstII BstOI AccB1I AcyI BstDSI Cfr42I MvaI BstOI EaeI
 BsiEI NaeI NspBII SacII MvaI BshNI BsaHI BstH2I SstII BstOI BstNI NlaIII
atgcccgcgggggcaggacgaccctggcgcccaccgcgggccaggcccccaggcacgcggcatgggtggccgcgg base pairs
tacgggcgcccccgtcctgctgggaccgcgggtggcgcccggtccgggggtccgtgcgccgtacccaccggcgcc 19576 to 19650
 Bse118I NlaIII KspI BstNI BanI Hin1I NarI BbeI MspA1I BstNI EcoRII Hsp92II
 Cfr10I FseI MspA1I EcoRII KasI Msp17I Bsp143II KspI SacII EcoO109I CfrI
 NgoAIV BsaOI DsaI Sfr303I Bst2UI BbiII EheI DsaI Sfr303I Bst2UI Bst2UI BstDSI

 SstII KasI Hsp92I HaeII BstNI HinfI XmaIII NgoAIV BsaOI
 NspBII Eco64I BbiII Bsp143II Bst2UI CfrI MroNI Bse118I
 KspI SacII Msp17I EheI EcoRII MaeIII EaeI Eco52I BsiEI NaeI
cgccccgcaccaggtcacgcgccgactcggcggcggcggcggccggcacggtaaacgtgggccagcccggaaatc base pairs
gcggggcgtggtccagtgcgcggctgagccgccgccgccgccggccgtgccatttgcacccggtcgggcctttag 19651 to 19725
 Sfr303I BshNI BsaHI BstH2I Tsp45I BstZI NgoMI Bsh1285I
 MspA1I BanI Hin1I NarI BbeI BstOI BglI EagI BssAI Cfr10I MaeII
 Cfr42I AccB1I AcyI SexAI MvaI PleI EclXI BsrFI BstMCI

 SduI BanII Cfr9I SmaI DraII BanII BbsI Bst2UI
 EcoO109I ApaI Eco47I AvaI BsoBI SduI FriOI BpuAI BstOI
 Bsp120I FriOI HgiEI Ama87I Bsp120I Eco24I MboII EcoRII
ccagcacggcaaagtattggacgggccctccccggacctcaaacccgggccccagaaaagcgaagacgggggcca base pairs
ggtcgtgccgtttcataacctgcccgggaggggcctggagtttgggcccggggtcttttcgcttctgcccccggt 19726 to 19800
 PspOMI BmyI SinI BcoI XmaI EcoO109I ApaI BstNI
 DraII Eco24I AvaII PspAI PspALI Bsp1286I Bbv16II
 Bsp1286I Bme18I Eco88I PspOMI BmyI BpiI MvaI

 BanII Eco47I
 BmyI HgiEI Ksp632I BstH2I
 SduI SinI DsaI TspRI MboII Bsp143II
gggctccgggggcggcgtggaccgtggtatgccactgccggaagagggcgaccagcgccggggcggagaacccgt base pairs
cccgaggcccccgccgcacctggcaccatacggtgacggccttctcccgctggtcgcggccccgcctcttgggca 19801 to 19875
 Bsp1286I Bme18I Eam1104I HaeII
 Eco24I AvaII EarI
 FriOI BstDSI

 BsrFI HaeII KspI
 BssAI NaeI BbvI NspBII
 SgrAI Bse118I BbvI Tsp45I NspBII BstDSI
cgccggcgctcacgaagtagtcgtagccgcgcggcagcagcacccgcgccgtgacccgctgcgggtgtccgcggg base pairs
gcggccgcgagtgcttcatcagcatcggcgcgccgtcgtcgtgggcgcggcactgggcgacgcccacaggcgccc 19876 to 19950
 MroNI Cfr10I Bst71I MaeIII MspA1I DsaI SstII
 NgoMI Bsp143II Bst71I MspA1I
 NgoAIV BstH2I Sfr3

 SacII EcoRII Bst2UI Eco64I BbiII Bsp143II MvaI
 EcoNI BstOI HgiEI KasI Hsp92I HaeII Bst2UI BstOI
 Cfr42I TthHB8I BstNI Bme18I AccB1I NarI BbeI BstOI EcoRII
gccgcaggccgacctcgcacacctcgaccaggtccgcgaaggcgccctccttcctggtcggcggaaacgccaggg base pairs
cggcgtccggctggagcgtgtggagctggtccaggcgcttccgcgggaggaaggaccagccgcctttgcggtccc 19951 to 20025
 TaqI Tth111I SinI BanI Msp17I EheI BstNI BstNI
 SexAI AtsI AvaII BshNI BsaHI BstH2I MvaI Bst2UI
 03I AspI MvaI Eco47I Hin1I AcyI EcoRII

 Eco47I BsrI
 HgiEI HphI BseNI
 BssHII SinI Tsp45I HgaI TspRI
tggtgtattcgcgcgcaaaacgcgcggtcctcgtcgtgatggtgacggcgagcgaggcggaggacgcgcactggg base pairs
accacataagcgcgcgttttgcgcgccaggagcagcactaccactgccgctcgctccgcctcctgcgcgtgaccc 20026 to 20100
 BsePI Bme18I MaeIII BsrSI
 AvaII Bse1I


 BstOI Bbv12I
 EcoRII BssHII AspHI BssHII
 Bsp68I EaeI MvaI BsePI BssHII SduI BsiHKAI
ggctgtcgcgaatggcggccaggcgcgcccacgccaaccgcgcgccggggtgctcggcgacgcgcgcggacaggg base pairs
ccgacagcgcttaccgccggtccgcgcgggtgcggttggcgcgcggccccacgagccgctgcgcgcgcctgtccc 20101 to 20175
 NruI CfrI AscI BsePI Bsp1286I HgaI
 BstNI BmyI BsePI
 Bst2UI Alw21I

 AccI Msp17I TthHB8I StyI BstNI EagI MroNI BsrFI Bsh1285I
 SalI Hin1I AcyI TaqI ErhI EcoRII BstZI BsrBI BstD102I
 NspBII HincII MaeII AatII BssT1I BstOI BseRI EclXI NgoMI BsiEI
ccagcgggtcgacgtcgaccttggcctccacgtccaggagggcggcgcgaggagcggccggcgggccccacgacg base pairs
ggtcgcccagctgcagctggaaccggaggtgcaggtcctcccgccgcgctcctcgccggccgcccggggtgctgc 20176 to 20250
 MspA1I HindII BsaHI AccI Eco130I Bst2UI EaeI Eco52I NgoAIV BstMCI
 TthHB8I Hsp92I HincII EcoT14I CfrI AccBSI Bse118I
 TaqI BbiII SalI HindII MaeII MvaI XmaIII BssAI Cfr10I

 Bsp1286I Msp17I TthHB8I EcoO109I BstNI EcoO109I Eco24I
 Bsp120I BmyI ApaI AcyI PpuMI Psp5II MvaI BbvI BsmFI Eam1104I BmyI
 PspOMI Eco24I BbiII TaqI SinI AvaII BstOI NspBII MboII EarI FriOI
ccctttcgaccctcacgaccagacccgtctgcgggtcccagcccaggcgcagcgggacgaagagggcccaccggc base pairs
gggaaagctgggagtgctggtctgggcagacgcccagggtcgggtccgcgtcgccctgcttctcccgggtggccg 20251 to 20325
 DraII BanII BsaHI Bme18I EcoRII MspA1I Bsp120I SduI
BsaOI SduI FriOI Hsp92I HgiEI Eco47I Bst71I PspOMI DraII
NaeI EcoO109I Hin1I HgaI DraII BsmFI Bst2UI Ksp632I Bsp1286I

 Bse118I BstNI EcoO109I BstNI BmyI
 BssAI BstH2I MvaI BstH2I PpuMI Psp5II MvaI
 BsrFI Bsp143II BpmI Bsp143II SinI AvaII BstOI
ccgtctggcgctccagggccgccagaacgcacgcatacagcgcccgccacagggtcgggtcccccaggggctcca base pairs
ggcagaccgcgaggtcccggcggtcttgcgtgcgtatgtcgcgggcggtgtcccagcccagggggtccccgaggt 20326 to 20400
 BanII HaeII Bst2UI HaeII Bme18I EcoRII SduI
 ApaI BglI EcoRII GsuI HgiEI Eco47I Bsp1286I
 Cfr10I BstOI DraII BsmFI Bst2UI

 BpmI CfrI BsiEI EagI Eco52I BcoI XmaI BbiII Alw26I
 FriOI BstZI BstMCI CfrI EclXI BsaOI AvaI SmaI MaeII BsmAI
Eco24I MspA1I EclXI BsaOI EaeI XmaIII BglI Eco88I Hin1I AcyI
gcggggaggcggccggggccgtcgcggcgcgggcggccgcgacggccccgggggccgagacgtcgggggagccgt base pairs
cgcccctccgccggccccggcagcgccgcgcccgccggcgctgccggggcccccggctctgcagccccctcggca 20401 to 20475
 BanII EaeI Eco52I BglI BstZI Bsh1285I PspAI PspALI BsaHI AatII
 NspBII XmaIII CciNI BstMCI Ama87I BsoBI Msp17I BsmBI
 GsuI EagI Bsh1285I NotI BsiEI SfiI Cfr9I Hsp92I Esp3I

 SbfI KspI SacII
 Sse8387I BsePI NspBII Hin1I
 SfcI BspMI BssHII BstDSI Cfr42I
agaagtcctgcaggtcggacgaaccaacggacacctccgcgaagcgcgcgcgcgcctcccccgcggcgtcgcgac base pairs
tcttcaggacgtccagcctgcttggttgcctgtggaggcgcttcgcgcgcgcgcggagggggcgccgcagcgctg 20476 to 20550
 BstSFI BsePI BssHII DsaI SstII BbiII
 PstI MspA1I Msp17I
 Sfr303I Hsp92I

AcyI
 HgaI
BsaHI BbvI
agaccagatacagcagggcgtggaggcagtcgcgcgtgcgcgggggcagccataccgcgtatagggtaatggcgc base pairs
tctggtctatgtcgtcccgcacctccgtcagcgcgcacgcgcccccgtcggtatggcgcatatcccattaccgcg 20551 to 20625
 NruI Bst71I
 Bsp68I


 BbiII HgaI AvaI
BstH2I Hin1I Ama87I PspALI
Bsp143II BseRI SfaNI NlaIII BsaHI BcoI XmaI TthHB8I
tgacgctctcctccacccaaacgatgccgggggcttccatgccacgacgcccgggggttgccgtgtatcgaacga base pairs
actgcgagaggaggtgggtttgctacggcccccgaaggtacggtgctgcgggcccccaacggcacatagcttgct 20626 to 20700
HaeII HgaI Hsp92II AcyI PspAI BsoBI TaqI
 Msp17I Cfr9I SmaI
 Hsp92I Eco88I

 BstOI Eco147I
 Hsp92II Pme55I
 HphI SfaNI BstNI AatI AluI
gcgcggccccagacttatagggtgctaaagttcaccgccccctgcatcatgggccaggcctcggtgggaagctcc base pairs
cgcgccggggtctgaatatcccacgatttcaagtggcgggggacgtagtacccggtccggagccacccttcgagg 20701 to 20775
 NlaIII MvaI
 EcoRII StuI
 Bst2UI SseBI

 Eco88I AccB1I NarI HaeII BstH2I
 XhoI PaeR7I KasI BbiII EheI AviII Bsp143II
 Sfr274I TspRI Eco64I BsaHI BbeI FspI BbvI
gacagagccgcctcgagaatgatgtcagtgttgggctgggcgccggaggcgtgcgtgcgcaagcagcgcccccac base pairs
ctgtctcggcggagctcttactacagtcacaacccgacccgcggcctccgcacgcacgcgttcgtcgcgggggtg 20776 to 20850
 Ama87I TaqI BanI Msp17I Bsp143II Bst71I
 BcoI BsoBI BshNI Hsp92I Acc16I HaeII
 AvaI TthHB8I Hin1I AcyI BstH2I

 AfaI
 Bst71I Csp6I MspA1I
 BssHII AluI BssHII BbvI PvuII
gcgggcgcgcgcagcttgaagcgcgcgcccgcaaactcccgcttatgggccatcagcagcgcgtacagctgtctg base pairs
cgcccgcgcgcgtcgaacttcgcgcgcgggcgtttgagggcgaatacccggtagtcgtcgcgcatgtcgacagac 20851 to 20925
 BsePI BbvI BsePI Bst71I AluI
 RsaI
 NspBII

 BsaHI
 HaeII TaqI BbiII Bst71I Tru1I
 HgaI AlwNI TthHB8I Hin1I AluI HphI Tru9I
tgcgtccggcaggcgctgtggtcgatgcggtgggcgtccagcagctccacgatggctcgcttggtgaggttttta base pairs
acgcaggccgtccgcgacaccagctacgccacccgcaggtcgtcgaggtgctaccgagcgaaccactccaaaaat 20926 to 21000
 Bsp143II SfaNI Msp17I BbvI MseI
 BstH2I Hsp92I
 AcyI HgaI

 Eco88I Bbv12I MscI NspBII Bsp143I
 PspAI PspALI AspHI CfrI AluI BsgI MboI DpnI
 Ama87I MaeII SduI BsiHKAI MspA1I DpnII
acgcgccccgccccgggaaacgtctgcgtgctcttggccagctgcaccccgaacagttcgccccagatgatcttg base pairs
tgcgcggggcggggccctttgcagacgcacgagaaccggtcgacgtggggcttgtcaagcggggtctactagaac 21001 to 21075
 BcoI XmaI Bsp1286I BalI Bst71I NdeII
 Cfr9I SmaI BmyI EaeI PvuII Sau3AI
 AvaI BsoBI Alw21I MluNI BbvI Kzo9I

 Bbv12I BsmBI Eco47I BsrBI Hsp92I
 AspHI Alw26I HgiEI DdeI Msp17I HgaI
 SduI BsiHKAI SinI BbvI AccBSI BsaHI
aacagcgacagcgcgtgctccgtctcgctcacggacccgcgcggggggcagccgctcagggcgtcggccacgcgc base pairs
ttgtcgctgtcgcgcacgaggcagagcgagtgcctgggcgcgccccccgtcggcgagtcccgcagccggtgcgcg 21076 to 21150
 Bsp1286I Bme18I Bst71I Hin1I EaeI
 BmyI BsmAI AvaII BstDEI BbiII CfrI
 Alw21I Esp3I BstD102I AcyI

 TthHB8I NspV BbvI
 Tru1I MaeII Bse1I Bsp119I MspA1I
 Tru9I HgaI Tsp45I TspRI BsrSI Csp45I NspBII NspI
ttaaccgcgtcctccgacagcaaggggccgtcggtcacgttacagtggcccagttcgaacaccagctgcatgtag base pairs
aattggcgcaggaggctgtcgttccccggcagccagtgcaatgtcaccgggtcaagcttgtggtcgacgtacatc 21151 to 21225
 MseI MaeIII BseNI BstBI AluI Bst71I
 MaeIII BsrI LspI Bpu14I NlaIII
 SfuI TaqI PvuII Hsp92II

 AvaI
 BstMCI Bst71I MaeII HinfI
 BsiEI AluI GsuI Ama87I EaeI TthHB8I
cggtcgtagtgggggttcagcagctccagcacgtcctcggggctaaaggttcgccccgaccccccggccatcgag base pairs
gccagcatcacccccaagtcgtcgaggtcgtgcaggagccccgatttccaagcggggctggggggccggtagctc 21226 to 21300
 Bsh1285I BbvI BpmI BcoI CfrI TaqI
 BsaOI Eco88I
 BsoBI

 StyI DsaI Bst71I
 SfcI Eco130I Hsp92II
 BsmFI PstI EaeI NcoI Bsp19I AluI MaeII
tcccactgcaggcacgcggccatggtgctgcacagacggaacagctcccagacgggggcgacgtttagggtgggg base pairs
agggtgacgtccgtgcgccggtaccacgacgtgtctgccttgtcgagggtctgcccccgctgcaaatcccacccc 21301 to 21375
 PleI TspRI CfrI EcoT14I BbvI
 BstSFI ErhI BstDSI BsgI
 BssT1I NlaIII

 Bsp143I BbiII BstDSI
 BpmI MboI DpnI Hin1I HgaI
 AluI AluI DpnII BstF5I AcyI
tgtagggccacaagctccagctctccggcggcgttgatcgtggggatgacgcccgtggcgtagtggtcgtaaagc base pairs
acatcccggtgttcgaggtcgagaggccgccgcaactagcacccctactgcgggcaccgcatcaccagcatttcg 21376 to 21450
 GsuI NdeII FokI BsaHI
 Sau3AI Msp17I DsaI
 Kzo9I Hsp92I

 Bst71I StyI DsaI HgaI BpiI Eco147I BstNI
 BstH2I Eco130I Hsp92II Bbv16II BpmI EcoRII
 MboII Bsp143II EaeI NcoI Bsp19I MboII StuI GsuI SexAI MvaI
cgccggaagatggcgctgctatgggcggccatggggacgcgaagacaggcctccagcagcaccaggtagatgaac base pairs
gcggccttctaccgcgacgatacccgccggtacccctgcgcttctgtccggaggtcgtcgtggtccatctacttg 21451 to 21525
 HaeII CfrI EcoT14I BsmFI BpuAI SseBI BbvI Bst2UI
 BbvI ErhI BstDSI BbsI AatI Bst71I
 BssT1I NlaIII Pme55I BstOI

 XmaIII EcoRII XmaIII NgoAIV BsaOI Hsp92I
 CfrI BsiEI BstNI CfrI MroNI Bse118I Msp17I
 EaeI Eco52I BstOI NlaIII EaeI Eco52I BsiEI NaeI BsaHI
cgcgtgcggccgaccaggctgttgaggccgcgcatgagcgcgaccacctcggccggcgcgacgtccggccggagg base pairs
gcgcacgccggctggtccgacaactccggcgcgtactcgcgctggtggagccggccgcgctgcaggccggcctcc 21526 to 21600
 BstZI BstMCI BglI Hsp92II BstZI NgoMI Bsh1285I MaeII
 EagI Bsh1285I MvaI EagI BssAI Cfr10I Hin1I AcyI
 EclXI BsaOI Bst2UI EclXI BsrFI BstMCI BbiII

 CfrI BsiEI RsaI EcoRII EcoO109I BstOI
 EaeI EclXI Csp6I BsmBI Bst2UI BsmFI PpuMI Psp5II
 EagI BstMCI TthHB8I BseRI Alw26I BstNI MaeII SinI AvaII
tacttttcgacgaaaaggcccacctcctccgtctcggcggcctgggccgacagggacgtgtcggggtcctggcag base pairs
atgaaaagctgcttttccgggtggaggaggcagagccgccggacccggctgtccctgcacagccccaggaccgtc 21601 to 21675
 BstZI Bsh1285I TaqI BsmAI BstOI AflIII Bme18I BstNI
 XmaIII BsaOI Esp3I MvaI HgiEI Eco47I
AatII Eco52I AfaI DraII EcoRII

 AluI NdeII Kzo9I Bsp120I BmyI Eco81I Bsu36I BsoBI
 BbvI DpnII XhoII NspBII SduI ApaI BstDEI Eco88I
 Bst2UI Bst71I Sau3AI AclWI EcoO109I BanII AocI MslI Ama87I
cgcagctcccgcagatcccgctgggccctcagggcatcaaaatgtatcccccgcaaaaacagacaaaagttcctc base pairs
gcgtcgagggcgtctagggcgacccgggagtcccgtagttttacatagggggcgtttttgtctgttttcaaggag 21676 to 21750
 MvaI BstYI MflI AlwI DraII FriOI Bse21I BcoI
 Bst71I BstX2I DpnI MspA1I Bsp1286I CvnI SfaNI AvaI
 BbvI MboI Bsp143I PspOMI Eco24I DdeI BglI

 BsaHI PmaCI BbuI Tru1I
 BbiII BsaAI NlaIII NspI Tru9I
 Hin1I MaeII MslI SphI NlaIII BstF5I
ggggtcagcgcggcgtcgtggccccagaaccgcacgtgcatgcagttgagggtcagaagcatgtggaggatgtta base pairs
ccccagtcgcgccgcagcaccggggtcttggcgtgcacgtacgtcaactcccagtcttcgtacacctcctacaat 21751 to 21825
 Msp17I Eco72I PaeI Hsp92II FokI
 Hsp92I PmlI Hsp92II MseI
 AcyI HgaI BbrPI NspI

 Bsp1286I ApoI
 ApaLI Bbv12I AfaI TspEI
 Alw44I Alw21I Csp6I Sse9I SfcI SfaNI
agactgtccgcgaggcacgccagcgtgcacctctcgaagtagtgcttgtaccggaatttgctgtagatgcgcgac base pairs
tctgacaggcgctccgtgcggtcgcacgtggagagcttcatcacgaacatggccttaaacgacatctacgcgctg 21826 to 21900
 VneI BmyI TthHB8I RsaI AcsI BstSFI
 SduI BsiHKAI BsaWI
 AspHI TaqI Tsp509I

 BstH2I Cfr10I
 HgaI Bsp143II EcoRII
 BglI AflIII BbvI BssAI Tsp45I
ccccgcgcctgcgccgcgtcggcgtgcgacgcgtcgcagcgccctttgaaccggcggcacaacaggttcgtcacc base pairs
ggggcgcggacgcggcgcagccgcacgctgcgcagcgtcgcgggaaacttggccgccgtgttgtccaagcagtgg 21901 to 21975
 HgaI MluI Bst71I BsrFI MaeIII
 HaeII Bse118I


BstOI NgoAIV TspRI BsaAI FriOI
 NgoMI CfrI BstH2I Eco72I NlaIII BmyI
 HphI MroNI Cfr10I NspBII Bsp143II PmaCI BbvI SduI
tgggaaaactgtgccggccactgcccgctggcgctcaccacgtggttgagcagcatgggcgtaaagacgggctcc base pairs
acccttttgacacggccggtgacgggcgaccgcgagtggtgcaccaactcgtcgtacccgcatttctgcccgagg 21976 to 22050
 MvaI BssAI EaeI MspA1I HaeII MaeII BbrPI Bst71I Bsp1286I
BstNI BsrFI NaeI HphI DraIII Hsp92II Eco24I
Bst2UI Bse118I PmlI BanII

 Eco47I MboI Kzo9I Van91I
 BsePI HgiEI DpnII AluI RsaI PflMI
 BssHII SinI NlaIII Bsp143I HinfI Csp6I AlwNI
gagcgcgccccggacccgtccatgtagatcagcagctcccccttgcggagagtccgtacccgccccagcgactgg base pairs
ctcgcgcggggcctgggcaggtacatctagtcgtcgagggggaacgcctctcaggcatgggcggggtcgctgacc 22051 to 22125
 Bme18I Hsp92II DpnI PleI AccB7I
 AvaII NdeII BbvI AfaI Esp1396I
 Sau3AI Bst71I

 BsrI NlaIII Bsp143I
 Csp6I Eam1105I NspI MboI
 BseNI AhdI NlaIII MaeII NlaIII DpnII
tacacggacaccatgtccggcccgtagttcatgggtttcacgtaggcgaacatgctgtcaaagtgcggcggatcg base pairs
atgtgcctgtggtacaggccgggcatcaagtacccaaagtgcatccgcttgtacgacagtttcacgccgcctagc 22126 to 22200
 Bse1I EclHKI Hsp92II BsaAI Hsp92II NdeII
 RsaI AspEI Sau3AI
BsrSI AfaI Hsp92II Kzo9I

 AluI BshNI RsaI KpnI MaeIII
DpnI DdeI BstMCI Asp718I Bse118I Tsp45I
 TthHB8I Tsp45I BsiEI Eco64I AfaI NlaIII
aagctaaggcccaccgtcacgaccgttgtgtagatgaccacccggtaccggccccatgtggtcacgtcgccgggc base pairs
ttcgattccgggtggcagtgctggcaacacatctactggtgggccatggccggggtacaccagtgcagcggcccg 22201 to 22275
 TaqI BstDEI MaeIII Bsh1285I BanI Csp6I Cfr10I MaeII
 AlwI BsaOI Acc65I BssAI Hsp92II
 AclWI AccB1I BsrFI

 CspI NgoAIV BstOI
 Bme18I NgoMI EcoRII
 HphI BbvI CpoI AvaII MroNI Cfr10I MvaI
ggggtgagcgagtggagcagcagcacgcggtccgtaaactgccggcagaacctggcaacgacctccgcgaaggag base pairs
ccccactcgctcacctcgtcgtcgtgcgccaggcatttgacggccgtcttggaccgttgctggaggcgcttcctc 22276 to 22350
 Bst71I SinI Eco47I BssAI NaeI BstNI
 HgiEI BsrFI AlwNI
 RsrII Bse118I Bst2UI

 SalI HindII NgoAIV EcoRII AscI BsaHI
 Eco31I NgoMI CfrI Bst2UI BpmI BbiII
 Alw26I HincII MboII MaeII MroNI Cfr10I MvaI AluI MboII Hin1I
accgtcgacgagaagatgcagacgttatctccgccggccaggcgcgcctccagctccccgaagaaggtggcgtcc base pairs
tggcagctgctcttctacgtctgcaatagaggcggccggtccgcgcggaggtcgaggggcttcttccaccgcagg 22351 to 22425
 BsmAI AccI SfaNI BssAI EaeI BstOI GsuI Msp17I
 BsaI TaqI BsrFI NaeI BglI BsePI Hsp92I
 TthHB8I Bse118I BstNI BssHII AcyI

 BsaHI AccB1I NarI HaeII BsrFI SbfI EcoO109I
 BbiII KasI BbiII EheI BssAI NaeI Sse8387I Eco47I
 Hin1I Eco64I BsaHI BbeI NgoMI SfcI SinI DraII BcoI
gggggggcgtccggggggggcgccccgcccgccggcccccggcggcgcagggccgcctgcaggacctcgggcccc base pairs
cccccccgcaggccccccccgcggggcgggcggccgggggccgccgcgtcccggcggacgtcctggagcccgggg 22426 to 22500
 Msp17I BanI Msp17I Bsp143II Cfr10I BstSFI PpuMI Psp5II
 Hsp92I BshNI Hsp92I MroNI NgoAIV PstI HgiEI Ama87I
HgaI AcyI HgaI Hin1I AcyI BstH2I Bse118I Bme18I AvaII

Eco88I SduI BanII AfaI PmaCI
 Bsp120I Eco24I BstOI BsePI Csp6I BsaAI
 PspOMI BmyI EcoRII AscI NlaIII MaeII MaeII
aggcgcgggagaaacagacaacggcgcgccgaaaatccgggcatggcgtactccccgatgaccacgtgaacgttc base pairs
tccgcgccctctttgtctgttgccgcgcggcttttaggcccgtaccgcatgaggggctactggtgcacttgcaag 22501 to 22575
 BsoBI Bsp1286I BstNI BssHII Hsp92II Eco72I
 EcoO109I ApaI MvaI RsaI PmlI Psp14
AvaI DraII FriOI Bst2UI BbrPI

 XcmI BsaHI StyI DsaI MslI Bsp143I
 BsgI NspBII BbiII ErhI BstDSI DpnII
 BglI BbvI PvuII Hin1I HgaI EcoT14I NdeII
ttttcgccccggaggctgcacagaaagtccaccagctgcgcgttggcggtggcgtccatggcgatgatccgcggg base pairs
aaaagcggggcctccgacgtgtctttcaggtggtcgacgcgcaaccgccaccgcaggtaccgctactaggcgccc 22576 to 22650
 Bst71I AluI Bst71I Msp17I BssT1I Hsp92II Kzo9I
06I MspA1I Hsp92I NcoI Bsp19I MboI
 BbvI AcyI Eco130I NlaIII Sau3AI

 MspA1I Bsp1286I FspI BbvI SalI HindII MspA1I
 DpnI NspBII SduI PmlI AviII SfaNI AccI HgaI AlwNI AfaI
 DsaI KspI SacII BsaAI Acc16I MluI HgaI PvuII SfaNI Csp6I
cacgtgcgcagcaggcgcagcatcaacgcgtcgacgcggcccagctgctgcatcgtcggcgagtacagttggccc base pairs
gtgcacgcgtcgtccgcgtcgtagttgcgcagctgcgccgggtcgacgacgtagcagccgctcatgtcaaccggg 22651 to 22725
 AlwI SstII BmyI PmaCI Bst71I TthHB8I AluI Bst71I RsaI
 BstDSI Sfr303I MaeII BbrPI Bst71I TaqI NspBII
 AclWI Cfr42I Eco72I BbvI AflIII HincII BbvI

 TaqI AtsI Bst2UI BmyI BsiHKAI BmyI Bbv16II
 TthHB8I AspI BstNI Bsp1286I SduI ApaI MboII
 MaeII HindII Tth111I DrdI MaeII Bsp120I BanII Asp7
aacgtcgacatgacttcgtccaggacgagcacgtcgtagttgttcaacaggttcgggcccacgcgatgaagactt base pairs
ttgcagctgtactgaagcaggtcctgctcgtgcagcatcaacaagttgtccaagcccgggtgcgctacttctgaa 22726 to 22800
 SalI NlaIII EcoRII SduI Alw21I PspOMI FriOI XmnI
 AccI Hsp92II BstOI AspHI Bsp1286I BpuAI
 HincII MvaI Bbv12I Eco24I BbsI

 BspHI Tsp509I AspI
 BsmBI BstMCI Hsp92II HphI
 00I BspMI Alw26I BsiEI NlaIII BstF5I Tsp45I
tccacctgcacgatgagacggtggaaggggcggtcgttcatgatgtaattggtggatgagaagtaggtgacgaag base pairs
aggtggacgtgctactctgccaccttccccgccagcaagtactacattaaccacctactcttcatccactgcttc 22801 to 22875
 BpiI BsgI BsmAI Bsh1285I Sse9I FokI MaeIII
 Esp3I BsaOI TspEI Tth111I
 RcaI AtsI

 Bst2UI
 BstDEI BstNI BstDEI BsmBI
 HinfI HgaI AlwNI Alw26I
tcgggcaaccctgactcagcgaaccgcgtcgccagggtctgagtaaaactccgacgacaggagacgaccagcaca base pairs
agcccgttgggactgagtcgcttggcgcagcggtcccagactcattttgaggctgctgtcctctgctggtcgtgt 22876 to 22950
 DdeI EcoRII DdeI BsmAI
 PleI BstOI Esp3I
 MvaI

 MroI Bsp13I Kzo9I MboI DpnI BsoBI FriOI
 BspEI BsiMI Bsp143I DpnII AclWI Eco88I BmyI BsePI
 MslI BsiI BsaWI DpnII AclWI NspBII Bsp143I Ama87I SduI BssHII
ctcgtgtccggagagtggatcgcttcccccaaccagcggatcagcgcggtagtttttcccgagcccattggcgcg base pairs
gagcacaggcctctcacctagcgaagggggttggtcgcctagtcgcgccatcaaaaagggctcgggtaaccgcgc 22951 to 23025
 BssSI BseAI Sau3AI MspA1I Kzo9I BcoI Bsp1286I
 AccIII NdeII AlwI NdeII AlwI AvaI Eco24I
 Kpn2I MboI DpnI Sau3AI BanII

 AvaII AvaII DpnII
 Bme18I BstH2I HphI TthHB8I
 MaeIII BglI Bsp143II Tsp45I SinI BbvI AccBSI
cggaccacagttacgcaccgggccgtcggggcgctcgcgtccgggaaggtgacgggtccgtgttgctgccgctcg base pairs
gcctggtgtcaatgcgtggcccggcagccccgcgagcgcaggcccttccactgcccaggcacaacgacggcgagc 23026 to 23100
 SinI HaeII HgaI MaeIII Bme18I Bst71I
 HgiEI HgiEI TaqI
 Eco47I Eco47I NdeII

 Sau3AI Bsh1285I Ama87I BsoBI
 Kzo9I Ple19I HgiEI PspAI PspALI FokI
 BstD102I BsaOI AvaII Eco88I EaeI SfaNI
atcgttgttttcgggtggacccggggaacccactcggccaaatcccccccgtaaagcatccgcgccagcgataca base pairs
tagcaacaaaagcccacctgggccccttgggtgagccggtttagggggggcatttcgtaggcgcggtcgctatgt 23101 to 23175
 BsrBI DpnI BsiEI SinI BcoI AvaI CfrI BstF5I
 MboI BspCI BstMCI Eco47I XmaI
 Bsp143I PvuI Bme18I Cfr9I SmaI

 AfaI BsaHI PspOMI Eco24I BstOI MboI MflI
 MaeII BbiII Bsp120I FriOI BstNI NdeII BstI
 TthHB8I RsaI BstF5I Hin1I HgaI SduI BanII BstYI Bsp143I
ctcgacgtgtactgctcgcactcgtcatccccgatgggacgccgggcccccaggggatcccccgaggccgcgccg base pairs
gagctgcacatgacgagcgtgagcagtaggggctaccctgcggcccgggggtcccctagggggctccggcgcggc 23176 to 23250
 TaqI Csp6I FokI Msp17I EcoO109I ApaI MvaI BstX2I XhoII
 AflIII Hsp92I DraII BmyI EcoRII DpnII BamHI
 AcyI BsmFI Bsp1286I Bst2UI Sau3AI Kzo9I

AclWI Eco64I Hsp92I HaeII BbiII Ama87I XmaI BsmBI
 AlwI BsoBI Hin1I NarI BbeI Hsp92I Eco88I Alw26I
DpnI AvaI BshNI BsaHI BstH2I MaeII PspAI PspALI BsgI MaeII EaeI
ggcgccgacgtcgcgcccggggcgcgggcggcgtggtgggtctggtgtgtgcaggtggcgacgttcatcgtctcg base pairs
ccgcggctgcagcgcgggccccgcgcccgccgcaccacccagaccacacacgtccaccgctgcaagtagcagagc 23251 to 23325
 BcoI BanI Msp17I Bsp143II BsaHI Cfr9I SmaI BspMI CfrI
 Ama87I KasI BbiII EheI Msp17I AatII AvaI BsmAI
 Eco88I AccB1I AcyI Hin1I AcyI BcoI BsoBI Esp3I

 FriOI BstOI
 BmyI BstNI
 XcmI SduI EcoRII BsmFI
gccatctgcgtcgtggggctcctggtgctggcctctgtgttccgggacaggtttccctgcctttacgcccccgcg base pairs
cggtagacgcagcaccccgaggaccacgaccggagacacaaggccctgtccaaagggacggaaatgcgggggcgc 23326 to 23400
 HgaI Bsp1286I
 Eco24I MvaI
 BanII Bst2UI

 TaqI
 BsiEI
 BstDSI BsaOI BsmFI
acctcttatgcgaaggcgaacgccacggtcgaggtgcgcgggggtgtagccgtccccctccggttggacacgcag base pairs
tggagaatacgcttccgcttgcggtgccagctccacgcgcccccacatcggcagggggaggccaacctgtgcgtc 23401 to 23475
 DsaI TthHB8I BsaWI
 Bsh1285I
 BstMCI

 BalI Pfl23II TspEI XmaIII Csp6I MspA1I EcoO65I
 CfrI SplI Csp6I MaeII CfrI BsiEI RsaI KspI SacII Tsp45I
 AlwNI MscI PspLI AfaI AccI EaeI Eco52I AfaI Sfr303I Eco91I
agcctgctggccacgtacgcaattacgtctacgctgttgctggcggcggccgtgtacgccgcggtgggcgcggtg base pairs
tcggacgaccggtgcatgcgttaatgcagatgcgacaacgaccgccgccggcacatgcggcgccacccgcgccac 23476 to 23550
 EaeI MaeII SunI Tsp509I BstZI BstMCI DsaI SstII BstEII
 MluNI BsiWI Sse9I EagI Bsh1285I NspBII BstPI
 BsaAI RsaI EclXI BsaOI BstDSI Cfr42I PspEI

 MvaI
 SfaNI BstOI
 HphI BssHII BstF5I EcoRII BglI SfaNI
acctcgcgctacgagcgcgcgctggatgcggcccgtcgcctggcggcggcccgtatggcgatgccacacgccacg base pairs
tggagcgcgatgctcgcgcgcgacctacgccgggcagcggaccgccgccgggcataccgctacggtgtgcggtgc 23551 to 23625
 BsePI FokI BstNI
 MaeIII Bst2UI


 Bsp143I
 MboI DpnI NspBII
 MaeII DpnII BbvI SfaNI PvuII
ctaatcgccggaaacgtctgcgcgtggctgttgcagatcacagtcctgctgctggcccaccgcatcagccagctg base pairs
gattagcggcctttgcagacgcgcaccgacaacgtctagtgtcaggacgacgaccgggtggcgtagtcggtcgac 23626 to 23700
 NdeII Bst71I AluI
 Sau3AI MspA1I
 Kzo9I

 MvaI HgiEI
 BstOI PpuMI
 MaeII BsgI BsiI EcoRII SinI AvaII
gcccaccttatctacgtcctgcactttgcgtgcctcgtgtatctcgcggcccatttttgcaccaggggggtcctg base pairs
cgggtggaatagatgcaggacgtgaaacgcacggagcacatagagcgccgggtaaaaacgtggtccccccaggac 23701 to 23775
 BssSI BstNI Bme18I
 Bst2UI DraII
 EcoO109I

Eco47I Csp6I AccB1I NarI HaeII CspI BsaWI
Psp5II MaeII BspMI KasI BbiII EheI Bme18I
 AccBSI AfaI HgaI Eco64I BsaHI BbeI CpoI AvaII
agcgggacgtacctgcgtcaggttcacggcctgattgacccggcgccgacgcaccatcgtatcgtcggtccggtg base pairs
tcgccctgcatggacgcagtccaagtgccggactaactgggccgcggctgcgtggtagcatagcagccaggccac 23776 to 23850
 DdeI BstD102I EcoNI BanI Msp17I Bsp143II SinI Eco47I
 BstDEI BsmFI BshNI Hsp92I HgaI HgiEI
 BsrBI RsaI Hin1I AcyI BstH2I RsrII

 Bse1I BmyI Bsp1286I EagI BstMCI MspA1I Eco31I
 BsrSI SduI ApaLI Bbv12I CfrI Bsh1285I Sfr303I
 Eco64I Alw44I Alw21I XmaIII BsaOI KspI Alw26I
cgggcagtaatgacaaacgccttattactgggcaccctcctgtgcacggccgccgccgcggtctcgttgaacacg base pairs
gcccgtcattactgtttgcggaataatgacccgtgggaggacacgtgccggcggcggcgccagagcaacttgtgc 23851 to 23925
 BanI BshNI VneI BmyI EaeI EclXI BstDSI SstII
 BseNI Bsp1286I SduI BsiHKAI BsiEI DsaI Cfr42I
 BsrI AccB1I AspHI BstZI Eco52I NspBII SacII

 Bsp143I BstMCI BsoBI NspI
 BsmAI Kzo9I PvuI Eco88I Hsp92II
 MboI BsiEI Ama87I PaeI HgaI
atcgccgccctgaacttcaacttttccgccccgagcatgctcatctgcctgacgacgctgttcgccctgcttgtc base pairs
tagcggcgggacttgaagttgaaaaggcggggctcgtacgagtagacggactgctgcgacaagcgggacgaacag 23926 to 24000
 BsaI Sau3AI Ple19I BcoI NlaIII
 DpnII DpnI Bsh1285I AvaI BbuI
 NdeII BspCI BsaOI SphI

 SduI BanII
 DraIII EcoO109I ApaI BsoBI
 TthHB8I Tsp45I MaeII Bsp120I FriOI Eco88I
gtgtcgctgttgttggtggtcgagggggtgctgtgtcactacgtgcgcgtgttggtgggcccccacctcggggcc base pairs
cacagcgacaacaaccaccagctcccccacgacacagtgatgcacgcgcacaaccacccgggggtggagccccgg 24001 to 24075
 TaqI MaeIII BsaAI PspOMI BmyI Ama87I
 DraII Eco24I AvaI
 Bsp1286I BcoI

 MvaI Bbv12I PinAI MslI
 Bse118I BstOI AspHI AgeI Bse118I
 BssAI SfaNI BstNI SduI BsiHKAI BsrFI MaeII BbvI
atcgccgccaccggcatcgtcggcctggcctgcgagcactaccacaccggtggttactacgtggtggagcagcag base pairs
tagcggcggtggccgtagcagccggaccggacgctcgtgatggtgtggccaccaatgatgcaccacctcgtcgtc 24076 to 24150
 BsrFI EcoRII Bsp1286I BsaWI MaeIII BsaAI Bst71I
 Cfr10I Bst2UI BmyI SgrAI Cfr10I
 Alw21I BssAI BstXI

 TspRI SduI ApaI Bst2UI NcoI Bsp19I
 EaeI EcoO109I BanII BstNI BstH2I StyI DsaI Hsp92II
 Bsp120I FriOI PleI HgaI Bsp143II Eco130I CfrI
tggccgggggcccagacgggagtccgcgtcgccctggcgctcgtcgccgcctttgccctcgccatggccgtgctt base pairs
accggcccccgggtctgccctcaggcgcagcgggaccgcgagcagcggcggaaacgggagcggtaccggcacgaa 24151 to 24225
 PspOMI Eco24I EcoRII HaeII ErhI BstDSI MslI
 CfrI DraII BmyI PshAI BstOI BssT1I EaeI
 Bsp1286I HinfI MvaI EcoT14I NlaIII

 ApaLI Bbv12I Cfr10I Tsp509I Hsp92II
 Bsp1286I BsrFI TspEI Acc16I SphI BshNI
 Alw44I Alw21I SgrAI Sse9I FspI PaeI Eco64I
cggtgcacgcgcgcctacctgtatcaccggcgacaccacactaaatttttcgtgcgcatgcgcgacacccggcac base pairs
gccacgtgcgcgcggatggacatagtggccgctgtggtgtgatttaaaaagcacgcgtacgcgctgtgggccgtg 24226 to 24300
 SduI BsiHKAI BssAI AcsI AviII NspI BanI
 VneI BmyI BssHII Bse118I ApoI NlaIII AccB1I
 AspHI BsePI HphI BbuI

 TaqI HgaI RsaI Cfr9I
 BstH2I SplI Csp6I BcoI
 BglI Bsp143II PspLI AfaI NlaIII MaeI Ama87I
cgcgcccattcggcgcttcgacgcgtacgcagctccatgcgcggttctaggcgtggcgggccgcccggagacccg base pairs
gcgcgggtaagccgcgaagctgcgcatgcgtcgaggtacgcgccaagatccgcaccgcccggcgggcctctgggc 24301 to 24375
 HaeII MluI BsiWI AluI Hsp92II BfaI Alw26I
 TthHB8I Pfl23II Bst71I PspAI
 AflIII SunI BbvI Eco88I

 XmaI Bsp143I BssAI Bsh1285I EcoO1
 BsmAI PspALI MboI DpnI BsaWI BstMCI PpuMI
 BsoBI BsmFI DpnII TthHB8I BsiEI TfiI SinI
ggctacgcggaaaccccctacgcgagcgtgtcccaccacgccgagatcgaccggtatggggattccgacggggac base pairs
ccgatgcgcctttgggggatgcgctcgcacagggtggtgcggctctagctggccatacccctaaggctgcccctg 24376 to 24450
 Eco31I NdeII TaqI BsrFI BsaOI Bme18I
 AvaI SmaI Sau3AI PinAI Cfr10I HgiEI
 BsaI Kzo9I AgeI Bse118I HinfI DraII

 09I NdeII Bsp1286I BanII BcoI Ama87I PspALI
 Psp5II Kzo9I EcoICRI Eco24I BsiHKAI PspAI BsoBI BmyI
 AvaII MboI BsiI Ecl136II SacI Alw21I BsoBI AvaI SduI
ccgatctacgacgaagtggcccccgaccacgaggccgagctctacgcccgagtgcaacgccccgggcctgtgccc base pairs
ggctagatgctgcttcaccgggggctggtgctccggctcgagatgcgggctcacgttgcggggcccggacacggg 24451 to 24525
 DpnII Bsp143I BssSI AluI BmyI FriOI Ama87I BcoI XmaI Bsp1286I
 Eco47I DpnI SduI Bbv12I SstI AvaI Cfr9I SmaI
 BsmFI Sau3AI AspHI Psp124BI Eco88I Eco88I

 Msp17I BmyI EcoT14I BstDSI Cfr42I Cfr10I
 BsaHI Bsp1286I StyI HgiEI NspBII SacII Bsp1407I
 Hsp92I SduI BstDSI Eco130I Eco47I SstII Bse118I AfaI
gacgccgagcccatttacgacaccgtggaggggtatgcgccaaggtccgcgggggagccggtgtacagcaccgtt base pairs
ctgcggctcgggtaaatgctgtggcacctccccatacgcggttccaggcgccccctcggccacatgtcgtggcaa 24526 to 24600
 AcyI FriOI DsaI ErhI Bme18I MspA1I BsrFI Csp6I
 Hin1I HgaI BanII BssT1I AvaII KspI BssAI BsrGI
 BbiII Eco24I SinI DsaI Sfr303I SspBI RsaI


 Tru1I
 Tru9I MaeII HphI BssHII
cggcgatggtagccgtttcgttcgttttaataaaccgacgttgtgcgtttcaccatacttcggcgcgcgtgtgtg base pairs
gccgctaccatcggcaaagcaagcaaaattatttggctgcaacacgcaaagtggtatgaagccgcgcgcacacac 24601 to 24675
 MseI BsePI


 EaeI BseRI
tgtgtttttttttttgtggtgtttattttccccccaccccttccttttctttcggccaccacccccctcctcccc base pairs
acacaaaaaaaaaaacaccacaaataaaaggggggtggggaaggaaaagaaagccggtggtggggggaggagggg 24676 to 24750
 CfrI


 AfaI
 RsaI
cgtactatacaacaaaaaataccacacatacgaccaaatacggacaatcatttctgtctttattcgctatcagag base pairs
gcatgatatgttgttttttatggtgtgtatgctggtttatgcctgttagtaaagacagaaataagcgatagtctc 24751 to 24825
 Csp6I


 EcoO109I Eco47I
 PpuMI Psp5II HgiEI
 MaeII SinI AvaII Tsp45I HgaI SinI
agtgggggcgtgagcgtggcaggagggcgggccacgtcggggtcccgccgtctggtgtgacgcgatggggggtcc base pairs
tcacccccgcactcgcaccgtcctcccgcccggtgcagccccagggcggcagaccacactgcgctaccccccagg 24826 to 24900
 Bme18I MaeIII Bme18I
 HgiEI Eco47I AvaII
 DraII BsmFI

 BsrFI PspOMI BmyI ApaI AccB1I AcyI BcoI Eco88I BstEII HphI BmyI
 SfaNI RsaI BseNI DraII BanII Hin1I NarI HaeII BbeI SmaI PspEI SduI
 BssHII Csp6I Bsp120I Eco24I Eco64I Hsp92I Bsp143II BsoBI EcoO65I Bsp1286I
gatgcgcgccggtactggggccccggcgcccgggtgaccacgcgcacgtcggggggcacgtagaagttaccctct base pairs
ctacgcgcggccatgaccccggggccgcgggcccactggtgcgcgtgcagccccccgtgcatcttcaatgggaga 24901 to 24975
 BsePI Cfr10I BsrSI EcoO109I BanI Msp17I Ama87I Cfr9I PspALI Tsp45I MaeII
 BssAI AfaI BsrI SduI FriOI BshNI BsaHI BstH2I AvaI Eco91I MaeIII MaeIII
 Bse118I Bse1I Bsp1286I KasI BbiII EheI PspAI XmaI BstPI MaeII BsaAI

 Ksp632I AhdI MaeII Sse9I
 Eam1104I PleI BbiII AatII BsmBI
 HinfI Eam1105I Hin1I AcyI Tsp509I Alw26I HphI
tcttcggactcgatgtccacgacgtcaaattcgtgggcggtcagcgagacgacctccccgccgtcggtggtgatg base pairs
agaagcctgagctacaggtgctgcagtttaagcacccgccagtcgctctgctggaggggcggcagccaccactac 24976 to 25050
 EarI TthHB8I Msp17I TspEI BsmAI
 TaqI AspEI Hsp92I ApoI Esp3I
 MboII EclHKI BsaHI AcsI

 AspEI
 EclHKI Esp1396I
 AhdI BbvI AccB7I TthHB8I
acgttgtgtcggcagcagcagggccgcgccccggagaacgcgaggcccataacttggcgagcgtatcgtcgaagg base pairs
tgcaacacagccgtcgtcgtcccggcgcggggcctcttgcgctccgggtattgaaccgctcgcatagcagcttcc 25051 to 25125
 MaeII Bst71I PflMI TaqI
 Eam1105I Van91I


 MvaI NdeII Kzo9I NdeII DpnII
 BstOI BsmFI Sau3AI AclWI DpnII DpnI MboI
 BstNI BstF5I BstYI MflI AlwI HgaI HphI Ksp22I NdeII
ccaggcggctgtttcgccggatgtcccggtagatccccggctcgacgcggacgggggtgatgatcagggcgatcg base pairs
ggtccgccgacaaagcggcctacagggccatctaggggccgagctgcgcctgcccccactactagtcccgctagc 25126 to 25200
EcoRII FokI DpnII XhoII TthHB8I FbaI Sau3AI Bsp143I
 Bst2UI BstX2I DpnI TaqI BclI Bsp143I Kzo9I
 MboI Bsp143I MboI Kzo9I Sau3AI

 DpnI BstMCI Bst2UI NdeII TthHB8I BscI AlwI SfaNI AvaII SduI BanII Bst2UI NspBII
 BspCI BsaOI MvaI Eco47I DpnI Bsp106I AclWI EcoT14I PspOMI BmyI EcoRII Bsp143II
 BsiEI EcoRII Bme18I MboI BanIII TaqI Eco130I SinI Bsp120I Eco24I MvaI BstH2I
gaacggcctggtccgggaggatcgatgccttggcgggtccgggggccccgccaggcccggcgggcgctccgcggc base pairs
cttgccggaccaggccctcctagctacggaaccgcccaggcccccggggcggtccgggccgcccgcgaggcgccg 25201 to 25275
 PvuI BstNI HgiEI Sau3AI BspDI Bsu15I ErhI Bme18I DraII FriOI BstOI BstDSI
 Bsh1285I SinI DpnII Kzo9I Bsa29I ClaI StyI HgiEI EcoO109I ApaI BglI DsaI
 Ple19I BstOI AvaII Bsp143I BspXI BseCI BssT1I Eco47I Bsp1286I BstNI HaeII

 KspI SacII BsaOI Bst2UI EcoRII DraIII
 EagI EclXI Bsh1285I BpmI BshNI MaeIII HphI
 BstZI XmaIII EcoRII GsuI BseRI Eco64I MaeII BstNI
cgtcctccaggcggaacgtcacgccctcctccgcgcccgcgcggtgcctgccgaggaacgtcaccaggtgcggtt base pairs
gcaggaggtccgccttgcagtgcgggaggaggcgcgggcgcgccacggacggctccttgcagtggtccacgccaa 25276 to 25350
 CfrI SstII Eco52I BstOI Tsp45I BanI Tsp45I Bst2UI
 MspA1I Cfr42I BstMCI MvaI MaeIII AccB1I SexAI MvaI
 EaeI Sfr303I BsiEI BstNI MaeII BstOI

 NdeII XhoII
 DpnII BglII
 TthHB8I BsgI Sau3AI DpnI
gcagggggcagtcgggaaagtggctgtcgaggacgtatccctgcaccaagatctgtttgaagttcgggtggcggg base pairs
cgtcccccgtcagccctttcaccgacagctcctgcatagggacgtggttctagacaaacttcaagcccaccgccc 25351 to 25425
 TaqI MaeII BstYI MflI
 BstX2I Kzo9I
 MboI Bsp143I

 FriOI Bsp1286I BanII BstNI BpmI
 BmyI EcoICRI Eco24I SstI Bst2UI
 MboII SduI AluI Ecl136II SacI Alw21I MvaI XcmI
ggttggcgaagatgggctcgcggcgaaccagctccccggagctccaggccacgggagagatggtgcgacgctcaa base pairs
ccaaccgcttctacccgagcgccgcttggtcgaggggcctcgaggtccggtgccctctctaccacgctgcgagtt 25426 to 25500
 Bsp1286I AluI BmyI FriOI BsiHKAI DsaI HgaI
 Eco24I SduI Bbv12I EcoRII GsuI
 BanII AspHI Psp124BI BstOI BstDSI

 BsaHI NdeII AclWI
 BbiII Tru1I BstH2I Bsp143I
 Hin1I HgaI Alw26I Tru9I Bsp143II AlwI
ggtcggggacgccaaacagaagcacctccgagacaacgccgctatttaactccaccagcgcccgatccggggcgg base pairs
ccagcccctgcggtttgtcttcgtggaggctctgttgcggcgataaattgaggtggtcgcgggctaggccccgcc 25501 to 25575
 Msp17I BsmAI MseI HaeII Sau3AI
 Hsp92I DpnII DpnI
 AcyI BsmFI MboI Kzo9I

 BsrFI BsmFI
 BssAI NaeI TfiI Bse1I HphI BstH2I Bsp12
 SfaNI SgrAI Bse118I TthHB8I BsrSI Tsp45I Bsp143II
agcatcgccttttttcgccggcggcgcgggaatcgagccagtcccggtcttgggtgacgagcgcctcctccgggc base pairs
tcgtagcggaaaaaagcggccgccgcgcccttagctcggtcagggccagaacccactgctcgcggaggaggcccg 25576 to 25650
 MroNI Cfr10I HinfI BseNI MaeIII HaeII BseRI
 NgoMI TaqI BsrI PspOMI
 NgoAIV

 BmyI Eco88I HgiEI
 0I BanII PspAI PspALI Bme18I FokI
 SduI ApaI Ama87I SmaI BstF5I BstF5I
ccggaacgcgcccgggcgcgaagtagcgcacgccggggttggggatggaccggatgaacgcccggaacgcctccg base pairs
ggccttgcgcgggcccgcgcttcatcgcgtgcggccccaacccctacctggcctacttgcgggccttgcggaggc 25651 to 25725
 Bsp1286I BcoI XmaI SinI Eco47I
 FriOI Cfr9I SrfI FokI BsaWI
 Eco24I AvaI BsoBI AvaII

 Bsp143I Bsh1285I HgiEI Eco47I RsaI KspI SacII Hin1I NarI BstH2I DsaI Sfr3
 MboI DpnI PvuI Bme18I SplI SunI NspBII BglI AccB1I AcyI SinI Eco47I
 DpnII BspCI BsaOI EcoO109I Pfl23II BstDSI Cfr42I BshNI BsaHI BbeI AvaII KspI
gcgatcgccgcgccatcaggtcctcgtacgcggaggccgcgggggcgccggggtccgcggggtcgaacgcgtact base pairs
cgctagcggcgcggtagtccaggagcatgcgcctccggcgcccccgcggccccaggcgccccagcttgcgcatga 25726 to 25800
 NdeII BsiEI SinI AvaII BsiWI DsaI SstII BanI Msp17I Bsp143II BstDSI
 Sau3AI Ple19I PpuMI Psp5II Csp6I MspA1I Eco64I BbiII EheI Bme18I NspBII
 Kzo9I SgfI BstMCI DraII PspLI AfaI Sfr303I KasI Hsp92I HaeII HgiEI MspA1I

 03I MluI MseI MvaI MvaI EcoO109I
SstII AflIII Tru1I BstOI BstOI PpuMI Psp5II
 TthHB8I Tru9I EcoRII EcoRII SinI AvaII
tggctcggcacttaacctcgtagaaggccaggggggtctggggggcgggggccaggtagccgtgagggtccctgg base pairs
accgagccgtgaattggagcatcttccggtccccccagaccccccgcccccggtccatcggcactcccagggacc 25801 to 25875
 Cfr42I Csp6I BstNI BstNI Bme18I BstNI
 SacII RsaI Bst2UI Bst2UI HgiEI Eco47I
 TaqI AfaI DraII EcoRII

 BstOI BmyI FokI MvaI BsaHI PpuMI Psp5II VneI BmyI
 MvaI BsiI BstNI BbiII MslI BglI SinI DraII MluI Bsp1286I
 Bst2UI MslI EcoRII Hin1I BsmFI NlaIII EaeI NlaIII EcoO109I SduI
ggcacacgaggatgtccagggacgcccccaccatgcccgtgtggccgtccatgaggaccccgcacgcgtgcacgt base pairs
ccgtgtgctcctacaggtccctgcgggggtggtacgggcacaccggcaggtactcctggggcgtgcgcacgtgca 25876 to 25950
 BsmFI BstF5I Msp17I HgaI Hsp92II Hsp92II AvaII Alw44I
 SduI BssSI BstOI Hsp92I CfrI Bme18I AflIII AspHI
 Bsp1286I Bst2UI AcyI HgiEI Eco47I ApaLI Bbv12I

 HgiEI Eco47I XmaI HphI Msp17I Msp17I BbiII DrdI
 EcoNI DraII BcoI Eco88I BstH2I BsaHI Hsp92I Hin1I HgaI
 Alw21I SinI AvaII Cfr9I PspALI Bsp143II AcyI BsaHI HincII BsaHI
tctcctcggcgaggtccccgggttggtgaaagacgaagcgcccggcgtcggcgtcgtcgttgacgcccgcgtccg base pairs
agaggagccgctccaggggcccaaccactttctgcttcgcgggccgcagccgcagcagcaactgcgggcgcaggc 25951 to 26025
MaeII BseRI Bme18I Ama87I BsoBI HaeII Hsp92I BbiII HindII AcyI HgaI
 BsiHKAI PpuMI Psp5II AvaI SmaI Hin1I HgaI AcyI Msp17I
 EcoO109I PspAI BsmFI BbiII Hin1I HgaI Hsp92I


 BspMI HphI HphI
cgcggcccacgcagtagcgaaacagcaggtttcgggccgtcggctcgttcacccgcccgaacatcaccgccgacg base pairs
gcgccgggtgcgtcatcgctttgtcgtccaaagcccggcagccgagcaagtgggcgggcttgtagtggcggctgc 26026 to 26100


 Eam1105I Hsp92I BsrI Eco47I
 BsrSI AspEI AcyI BseNI HgiEI
 AhdI BsrI BsaHI HphI TspRI SinI BssHII
actgggcgtccagccgcaggctggcgttgtgggtgagccactgggacgagaagcacggaccctgcgcgccccacc base pairs
tgacccgcaggtcggcgtccgaccgcaacacccactcggtgaccctgctcttcgtgcctgggacgcgcggggtgg 26101 to 26175
 EclHKI BbiII XcmI BsrSI Bme18I BsePI
 BseNI Hin1I HgaI Bse1I AvaII
 Bse1I Msp17I BsmFI

 SduI ApaI MvaI
 BstMCI EcoO109I BanII BstOI
 BbvI BsiEI EcoO109I Bsp120I FriOI EcoRII HgaI
gcagcgtggaggcggtcgtcaggccccgccgaagcagggcccagagctggcagtcggcctggttttgcgtcgccg base pairs
cgtcgcacctccgccagcagtccggggcggcttcgtcccgggtctcgaccgtcagccggaccaaaacgcagcggc 26176 to 26250
 Bst71I Bsh1285I PspOMI Eco24I BstNI
 BsaOI DraII DraII BmyI AluI Bst2UI
 Bsp1286I

 BshNI
 BanI
 HgaI Hin1I
cctcgtaaaatcccataagcgggcggggggcgacggcttcggcggcggacgggggggcgcggcgcgtcaggcgcc base pairs
ggagcattttagggtattcgcccgccccccgctgccgaagccgccgcctgcccccccgcgccgcgcagtccgcgg 26251 to 26325
 Eco64I
 KasI
 AccB1I

 Hsp92I BbeI BshNI NgoAIV EagI Bsh1285I DsaI KspI SacII MroNI NgoAIV EagI Bsh1285I
Msp17I Bsp143II NgoMI BstZI Eco52I BmyI BstDSI Cfr42I Eco47I BsrFI BstZI BsiEI
 AcyI Eco64I BssAI EaeI XmaIII SduI Eco24I Sfr303I AvaII Hsp92II CfrI Eco52I
agaggtgccggccgagcccgcggtccaccatgccggccgcctccagcgacacgacgagggagcacagatagtcca base pairs
tctccacggccggctcgggcgccaggtggtacggccggcggaggtcgctgtgctgctccctcgtgtctatcaggt 26326 to 26400
 BsaHI BstH2I MroNI Cfr10I NaeI BstMCI BanII SstII Bme18I NgoMI EaeI XmaIII
BbiII EheI BanI BsrFI CfrI EclXI BsaOI FriOI MspA1I HgiEI BssAI Cfr10I NaeI
 NarI HaeII AccB1I Bse118I BsiEI Bsp1286I NspBII SinI NlaIII Bse118I EclXI

 Bbv12I BstOI FriOI EcoO109I EaeI BsrBI Hsp92I TaqI
 BpmI Alw21I MvaI Eco24I DraII BanII BalI Msp17I Bst71I HaeII Psp14
 SduI BsiHKAI SduI BanII Bsp1286I CfrI BstD102I BbvI BspMI TthHB8I
ggcgagcccacaggggcccgatggccagaggggagcggacgccgcgcagcaggccgcgcaggtggcgctcgaacg base pairs
ccgctcgggtgtccccgggctaccggtctcccctcgcctgcggcgcgtcgtccggcgcgtccaccgcgagcttgc 26401 to 26475
 GsuI Bsp1286I Bst2UI Bsp120I BmyI ApaI AccBSI BsaHI Bsp143II
 BstMCI BmyI EcoRII BmyI PspOMI Eco24I MscI Hin1I AcyI BstH2I
 BsaOI AspHI BstNI Bsp1286I SduI FriOI MluNI BbiII HgaI MaeII

 Bsp143I Msp17I Eco47I Eco47I
 06I Esp1396I MboI DpnI Hsp92I HgiEI HgiEI
 AccB7I TspRI DpnII AclWI AcyI SinI SinI
tttccgccaagatatgggggggcagtgcgttggggatcgccgacgccgaccacatcgggtcggggtccgggggac base pairs
aaaggcggttctatacccccccgtcacgcaacccctagcggctgcggctggtgtagcccagccccaggccccctg 26476 to 26550
 PflMI NdeII AlwI BsaHI Bme18I Bme18I
 Van91I Sau3AI Hin1I HgaI AvaII AvaII
 Kzo9I BbiII

 PstI
 BbvI
 SfcI TthHB8I
cggggctgcagtccgggtcgatggcgtgtgcgccccccggcgagaggggaatgtcgggggttggcgggccggatg base pairs
gccccgacgtcaggcccagctaccgcacacgcggggggccgctctccccttacagcccccaaccgcccggcctac 26551 to 26625
 BstSFI TaqI
 Bst71I
BsmFI

 AatI EcoNI Eco88I PspOMI BmyI PmaCI
 Pme55I PspAI PspALI Bsp1286I BsaAI
BstF5I DdeI BsmFI Ama87I Bsp120I Eco24I MaeII
aggcctcagagagggccggggacgcgggccgggccttttcgcccggggccccgccgtcgggttgcccacgtgggg base pairs
tccggagtctctcccggcccctgcgcccggcccggaaaagcgggccccggggcggcagcccaacgggtgcacccc 26626 to 26700
FokI SseBI HgaI BcoI XmaI EcoO109I ApaI Eco72I
 StuI BstDEI Cfr9I SmaI DraII BanII PmlI
 Eco147I AvaI BsoBI SduI FriOI BbrPI

 FriOI Eco88I PspOMI BmyI Tsp45I SduI ApaI
 BmyI PspAI PspALI Bsp1286I MaeII EcoO109I BanII
 SduI Ama87I Bsp120I Eco24I MaeIII Bsp120I FriOI
ggctctggggccaatgggaacccggggcccccggtgacgtggggcggggtggggcggggcggggcccaaagacgg base pairs
ccgagaccccggttacccttgggccccgggggccactgcaccccgccccaccccgccccgccccgggtttctgcc 26701 to 26775
 Bsp1286I BcoI XmaI EcoO109I ApaI PspOMI Eco24I
 Eco24I Cfr9I SmaI DraII BanII DraII BmyI
 BanII AvaI BsoBI SduI FriOI HphI Bsp1286I

 BsaOI NdeII XhoII Eco47I Sfr303I
 BstYI Bsp143I HgiEI NspBII Bsp143II
 BsiEI BstX2I Kzo9I BsmFI SinI DsaI SstII
tcgccagatctaggctgttgggtcggggccgcttcgggggactatcggggtcgcgggcggggtccgcggggcgct base pairs
agcggtctagatccgacaacccagccccggcgaagccccctgatagccccagcgcccgccccaggcgccccgcga 26776 to 26850
 Bsh1285I Sau3AI DpnI Bme18I KspI SacII
 BstMCI MboI BglII BfaI AvaII MspA1I HaeII
 DpnII MflI MaeI BstDSI Cfr42I

 BstH2I Hin1I NarI BbeI EagI BsiEI Ksp632I Ama87I TthHB8I Psp124BI
 AccB1I EheI BstZI EclXI Eam1104I SapI SduI BmyI SacI
 Eco64I BbiII AcyI EaeI XmaIII BsaOI BbvI MboII EarI Eco88I Bsp1286I FriOI
tggcgccgggtgttgcggcggccgccatttttacgagcagccgaagagctcgagggcggaagggatcctcacgac base pairs
accgcggcccacaacgccgccggcggtaaaaatgctcgtcggcttctcgagctcccgccttccctaggagtgctg 26851 to 26925
 KasI Hsp92I HaeII CfrI Eco52I Bst71I Ecl136II BcoI BsoBI Bbv12I
 BshNI BsaHI BstH2I CciNI BstMCI AluI Sfr274I AvaI AspHI TaqI
 BanI Msp17I Bsp143II NotI Bsh1285I EcoICRI XhoI PaeR7I Eco24I

 DpnII BamHI DpnI EaeI Eco52I MaeIII DdeI AvaI
 SstI NdeII XhoII BsePI XmaIII BglI Eco31I BbvI BcoI
 BstYI Bsp143I BssHII EclXI BsaOI Alw26I NspBII AluI
agagagtggcgcgcggccgggttggcgtgacagaggcgggagaccagcaccagcagcggcctcagctcgggcggc base pairs
tctctcaccgcgcgccggcccaaccgcactgtctccgccctctggtcgtggtcgtcgccggagtcgagcccgccg 26926 to 27000
BanII BstX2I BstI AlwI BstZI BstMCI BsmAI MspA1I Ama87I
 BsiHKAI Sau3AI Kzo9I CfrI BsiEI Tsp45I BsaI Bst71I Eco88I
Alw21I MboI MflI AclWI EagI Bsh1285I BstDEI BsoBI


 Tsp509I
 BbvI Sse9I TspRI BssHII HinfI
agcgacaccgacgacaggacggccttgtgcgtgcgctggtaatttatacactgctccgtgaacgcgcgccgaatc base pairs
tcgctgtggctgctgtcctgccggaacacgcacgcgaccattaaatatgtgacgaggcacttgcgcgcggcttag 27001 to 27075
 Bst71I TspEI BsePI TfiI


 AccB1I NarI HaeII MvaI AccB7I EagI XmaIII
 KasI BbiII EheI FokI BstOI Esp1396I CfrI BsmAI
 Eco64I BsaHI BbeI SfaNI EcoRII DsaI EaeI BsaI
ttgggattgcgaaggtggcgccggatgccctccggcacgtcatacgccaggccgtgggtgttggtctcggccgag base pairs
aaccctaacgcttccaccgcggcctacgggaggccgtgcagtatgcggtccggcacccacaaccagagccggctc 27076 to 27150
 BanI Msp17I Bsp143II BstNI PflMI BstZI EclXI
 BshNI Hsp92I BstF5I MaeII Bst2UI Van91I Alw26I
 Hin1I AcyI BstH2I BstDSI Eco31I

 HincII
 BstMCI DsaI
 Bsh1285I BsgI BbvI BseRI AluI
ttgacaaagagggcggggtgcagaacgcagcgataggcgaggagggccacggcaaagtccggcgagagctggttg base pairs
aactgtttctcccgccccacgtcttgcgtcgctatccgctcctcccggtgccgtttcaggccgctctcgaccaac 27151 to 27225
 BsaOI Bst71I BstDSI
Eco52I
BsiEI HindII

 ScaI BsrI PspAI SmaI Tsp45I BsaHI BstNI BcoI AfaI
 Tru1I RsaI BseNI Eco88I HgaI BbiII EcoRII AvaI BsaAI
 Tru9I Acc113I Ama87I BsoBI MaeIII Hin1I HgaI MvaI MaeII AluI
ttaaagtactggtagcccgggacgcgggtcacggggacgcccaggctcggggccacgtacacgctaaccagcagc base pairs
aatttcatgaccatcgggccctgcgcccagtgcccctgcgggtccgagccccggtgcatgtgcgattggtcgtcg 27226 to 27300
 MseI Csp6I BsrSI Cfr9I BsmFI Msp17I BstOI Eco88I Csp6I
 AfaI Bse1I AvaI PspALI Hsp92I Bst2UI BsoBI RsaI
 Eco255I BcoI XmaI PshAI AcyI BsmFI Ama87I

 MvaI Bsp143I BstMCI MaeII
Bst71I BbvI EcoRII MboI DpnI BsaOI BbiII AatII Tru1I
 GsuI AlwNI BstNI DpnII TthHB8I Hin1I AcyI Eco57I Tru9I
tccagcagcgtctgccccagggcgtagagatcgaccgccagcccgacgtcgtgcttcagggggcggttgttaaac base pairs
aggtcgtcgcagacggggtcccgcatctctagctggcggtcgggctgcagcacgaagtcccccgccaacaatttg 27301 to 27375
BbvI BpmI HgaI BstOI NdeII TaqI Msp17I MseI
 Bst71I Bst2UI Sau3AI BsiEI Hsp92I
 Kzo9I Bsh1285I BsaHI

 Bsp1286I FriOI Csp6I
 BstD102I AfaI EcoICRI BsaWI BanII BmyI
 AccBSI Csp6I Ecl136II Psp124BI RsaI SduI MslI
tcggcccgctcgttgttgaggtactttaccgagagctccggtggctggttgtacccgtgccccaccagagtgtga base pairs
agccgggcgagcaacaactccatgaaatggctctcgaggccaccgaccaacatgggcacggggtggtctcacact 27376 to 27450
 BsrBI RsaI AluI BmyI SacI SstI AfaI Bsp1286I
 SduI Bbv12I Alw21I
 AspHI Eco24I BsiHKAI

 Eco88I Ama87I BsmFI Eam1105I BstNI
 BstDSI FokI PspAI PspALI Eco88I PleI SinI Eco47I
 EaeI SfaNI Ama87I HinfI AvaI TaqI AspEI AvaII MvaI
aagttggccgtggtcagggcggcgggcatcccaaacccccggggggactcgaggtccggctcctggaggcaaaac base pairs
ttcaaccggcaccagtcccgccgcccgtagggtttgggggcccccctgagctccaggccgaggacctccgttttg 27451 to 27525
 CfrI BstF5I BcoI XmaI Sfr274I TthHB8I Bme18I BstOI
 DsaI Cfr9I SmaI XhoI PaeR7I EclHKI EcoRII
 AvaI BsoBI BcoI BsoBI AhdI HgiEI Bst2UI

 BseNI Eco88I PspEI EcoRII BstOI CfrI MroNI Bse118I
GsuI Ama87I BsoBI BstPI MaeIII CfrI SfiI XmaIII NgoAIV
 BsrI PspAI SmaI Eco91I BstNI MvaI EcoRII BglI EclXI BsrFI
tggccccgggatatcgtggagttggagttcagggtcaccaggctaaagtcggccaggacggccggccggagcgac base pairs
accggggccctatagcacctcaacctcaagtcccagtggtccgatttcagccggtcctgccggccggcctcgctg 27526 to 27600
 BsrSI Cfr9I Eco32I BstEII BstOI EaeI MvaI EaeI Eco52I Cfr10I
BpmI BcoI XmaI EcoRV EcoO65I HphI BstNI BstZI BssAI BsiEI
 Bse1I AvaI PspALI Tsp45I Bst2UI Bst2UI EagI NgoMI Bsh1285I

 AhdI NdeII DpnI Ple19I SfaNI MvaI Bsp143I EcoRII
 BsaOI AspEI Bsp143I BstMCI BssSI BstOI MboI DpnI BstOI
 BstMCI Eam1105I BspCI BsaOI BsiI EcoRII DpnII AclWI MvaI
accgcgtccgatcgcagcatcacgaggacgttggcgcacttgatgtccaggtggctgatcccgcacctggtgttc base pairs
tggcgcaggctagcgtcgtagtgctcctgcaaccgcgtgaactacaggtccaccgactagggcgtggaccacaag 27601 to 27675
 FseI DpnII Kzo9I PvuI Bst71I BstNI NdeII AlwI BstNI
 NaeI HgaI MboI BsiEI BbvI MaeII Bst2UI Sau3AI SexAI DraIII
 EclHKI Sau3AI Bsh1285I Kzo9I Bst2UI

 MvaI
 BsePI BstOI
 BstDSI EcoRII TspRI Bsp68I BssHII
aggaacaccacggcgcgcgccaggtctgtgaagcagtggtggagggccgtcgcgacggagggggtggtcgcgcgc base pairs
tccttgtggtgccgcgcgcggtccagacacttcgtcaccacctcccggcagcgctgcctcccccaccagcgcgcg 27676 to 27750
 DsaI BssHII BstNI NruI BsePI
 Bst2UI


 BsaHI AluI CfrI AvaII Pfl23II Bsp143I BbvI
 BbiII PvuII AfaI Bme18I SplI Csp6I MboI DpnI BstH2I PstI
 Hin1I HgaI EaeI Csp6I BcgI NlaIII SunI DpnII Bsp143II
agggacgccagctggccgatgtacttgccgaggtccatgtcgtacgcggggaacacgatctggcgctgctgcagc base pairs
tccctgcggtcgaccggctacatgaacggctccaggtacagcatgcgccccttgtgctagaccgcgacgacgtcg 27751 to 27825
 Msp17I NspBII RsaI SinI Hsp92II RsaI NdeII HaeII Bst71I
 Hsp92I MspA1I HgiEI PspLI AfaI Sau3AI SfcI
 AcyI BsmFI Eco47I BsiWI Kzo9I BstSFI

 AvaI KspI SacII EagI Eco52I EarI
 Eco88I BstD102I NspBII BstF5I CfrI EclXI BsaOI
BbvI Ama87I AccBSI BstDSI Cfr42I EaeI XmaIII MboII BsiI
gagaacccgagcggggtgataaagccgcggatgtcgtgggtgcggccgccgcgaagagcgcactcccccacgagc base pairs
ctcttgggctcgccccactatttcggcgcctacagcacccacgccggcggcgcttctcgcgtgagggggtgctcg 27826 to 27900
 BcoI BsrBI DsaI SstII BstZI Bsh1285I Eam1104I BssSI
Bst71I HphI MspA1I FokI CciNI BstMCI Ksp632I
 BsoBI Sfr303I NotI BsiEI SapI

 Bsp1286I BanII DsaI BpiI
 EcoICRI Eco24I BsiHKAI Bbv16II HinfI
 Bsp68I Ecl136II SacI Alw21I BstF5I AluI Bsp68I
agggtcgcgacgagctccacggcaaaccactctttttcccggatggtcttcacggcgagcttgtgttcgcgaatc base pairs
tcccagcgctgctcgaggtgccgtttggtgagaaaaagggcctaccagaagtgccgctcgaacacaagcgcttag 27901 to 27975
 NruI AluI BmyI FriOI BstDSI FokI BpuAI NruI
 SduI Bbv12I SstI BbsI TfiI
 AspHI Psp124BI MboII

 BsoBI FriOI EcoO109I PspAI BanII DpnII MflI AclWI
 AfaI Eco88I BmyI Bst71I SduI Eco24I ApaI BstX2I Kzo9I
 BcgI Csp6I Ama87I SduI AluI Bsp120I BcoI AvaI BsoBI NdeII XhoII
aactgcacctcgccgtacccccccgagcccccgaagctgcgggccccggggatctccagggtcgtgtagcggagg base pairs
ttgacgtggagcggcatgggggggctcgggggcttcgacgcccggggcccctagaggtcccagcacatcgcctcc 27976 to 28050
 BsgI RsaI BcoI Bsp1286I BbvI Ama87I Cfr9I XmaI SmaI Sau3AI
 AvaI Eco24I PspOMI Bsp1286I FriOI BstYI Bsp143I
 BanII DraII BmyI Eco88I PspALI MboI DpnI

 GsuI Zsp2I SfaNI
 BstNI BpmI FokI Mph1103I BssHII
 EcoRII HindII Ppu10I EcoT22I BstF5I BsmFI BstF5I
gcggggttgacggcgaatacggggatgcatagcttgtggatgcgcgcgagggacaggatgtgcgaggggggcgac base pairs
cgccccaactgccgcttatgcccctacgtatcgaacacctacgcgcgctccctgtcctacacgctccccccgctg 28051 to 28125
AlwI MvaI BstF5I AluI FokI FokI
 BstOI HincII SfaNI BsePI
 Bst2UI NsiI

 SinI BspMI BshNI Hsp92I DdeI CfrI Eco88I
 NlaIII BsmBI AvaII BanI Msp17I HaeII EaeI Ama87I
 EaeI Alw26I FspI Eco64I BsaHI BbeI AluI EcoO109I
gggggcgaggtcatggccgtctcggacctgcgcaggggcgggcgccttagcttggccgcagggccgggggcctcg base pairs
cccccgctccagtaccggcagagcctggacgcgtccccgcccgcggaatcgaaccggcgtcccggcccccggagc 28126 to 28200
 CfrI BsmAI HgiEI Acc16I Hin1I NarI Bsp143II DraII BsoBI
 Hsp92II Esp3I Eco47I KasI BbiII EheI BstDEI BcoI
 Bme18I AviII AccB1I AcyI BstH2I AvaI

 BsrBI
 BstD102I BsmBI BstD102I
 BsmFI AccBSI Alw26I BsmFI BsmFI AccBSI AluI
ggggacgagcggcgacgagacgagcggctcactcgccatcgggacagtcccgcgcgaagccgctcccggaagctg base pairs
cccctgctcgccgctgctctgctcgccgagtgagcggtagccctgtcagggcgcgcttcggcgagggccttcgac 28201 to 28275
 BsrBI BsmAI AccBSI BsrBI
 Esp3I
 BstD102I

Sau3AI EcoO109I PspAI BsmFI AccIII FriOI BsmAI AccB1I AcyI HaeII AvaI
NdeII AlwI PpuMI Psp5II AvaI SmaI BmyI BseAI Alw26I Hin1I NarI Bsp143II BsmBI
 Kzo9I SinI AvaII Cfr9I PspALI Kpn2I BanII KasI Esp3I BsaHI BstH2I Alw26I
gatcggcggcgggacccggggcgggctccggagacggcgccgtctcggggggaggggccgcttgggcgtccggac base pairs
ctagccgccgccctgggccccgcccgaggcctctgccgcggcagagcccccctccccggcgaacccgcaggcctg 28276 to 28350
DpnII AclWI Bme18I BcoI Eco88I Bsp1286I Eco24I Eco64I Msp17I EheI Ama87I BsmAI
MboI DpnI HgiEI Eco47I XmaI SduI MroI Bsp13I BanI BshNI Hsp92I BbeI Eco88I
Bsp143I DraII Ama87I BsoBI BspEI BsaWI BsiMI BsmBI BbiII DrdI BcoI BsoBI

Esp3I BsaHI BseAI BbiII DdeI MvaI AatI Bsp1286I BssT1I
 Hsp92I Kpn2I Hin1I HgaI BstOI SseBI VneI BmyI Eco130I
 Msp17I MroI BsiMI BsaHI HgaI BstNI Eco147I HgaI SduI BsiHKAI
gcccggcggctgagggagtgtatgtaggacgcgagccaggccttgaaggagcgtcggtgtgcaccttgggggctg base pairs
cgggccgccgactccctcacatacatcctgcgctcggtccggaacttcctcgcagccacacgtggaacccccgac 28351 to 28425
 BbiII AccIII HgaI AcyI EcoRII StuI Alw44I Alw21I EcoT14I
 BglI AcyI BsaWI Msp17I BstDEI Bst2UI ApaLI Bbv12I StyI
 Hin1I BspEI Bsp13I Hsp92I Pme55I AspHI ErhI

 BbvI DrdI MvaI
 NspBII MaeI FokI BstOI
 PvuII NlaIII BspMI HinfI BstF5I EcoRII BsmFI
atgtcagctgccacatgactagcaggtcgctgtcgcccggactcatccatccgtccgccaggtcgccgtcccccc base pairs
tacagtcgacggtgtactgatcgtccagcgacagcgggcctgagtaggtaggcaggcggtccagcggcagggggg 28426 to 28500
 AluI Hsp92II PleI BstNI
 MspA1I BfaI Bst2UI
 Bst71I

 BsmBI KspI SacII MboII BstOI Eco47I Bsp143I Bsh1285I BstNI
 Alw26I DsaI SstII TaqI AluI EcoRII Bme18I MboI DpnI PvuI EcoRII
 AflIII BstDSI Cfr42I EarI BbvI BseRI SinI DpnII PshAI BstMCI MvaI
acagagacgcgttcgccgcggcctcttcgagctgctcctcctggtccgcaagacgatcgtccgccgcgtccaggc base pairs
tgtctctgcgcaagcggcgccggagaagctcgacgaggaggaccaggcgttctgctagcaggcggcgcaggtccg 28501 to 28575
 MluI HgaI NspBII TthHB8I Bst71I MvaI HgiEI NdeII BspCI BsaOI Bst2UI
 BsmAI MspA1I Eam1104I BstNI AvaII Sau3AI Ple19I HgaI
 Esp3I Sfr303I Ksp632I Bst2UI Kzo9I BsiEI BcgI BstOI

 BstH2I Bsp1720I Kzo9I AlwI AccB1I MboII
 Bsp143II NdeII TaqI Asp718I AfaI Ksp632I
 Bpu1102I DpnII TthHB8I BshNI KpnI MaeII Eam11
gctcgctaagcgcgggatcgaggtaccgtcggtgtgcggttagaaaatcacgtcgcgccgcttgctcttccacgc base pairs
cgagcgattcgcgccctagctccatggcagccacacgccaatcttttagtgcagcgcggcgaacgagaaggtgcg 28576 to 28650
 DdeI BstDEI Bsp143I BanI Csp6I SapI
 HaeII BlpI MboI DpnI Eco64I RsaI EarI
 CelII Sau3AI AclWI Acc65I

 TspEI
 Tsp509I BstDEI Tsp509I
04I Tru9I SfaNI BssHII BsmFI Sse9I
gaattttaacacaggtcgctcgctgtcgcatcatctctaagcgcgcgcgggactttagccgcgcctccaattcca base pairs
cttaaaattgtgtccagcgagcgacagcgtagtagagattcgcgcgcgccctgaaatcggcgcggaggttaaggt 28651 to 28725
 Sse9I DdeI TspEI
 AcsI MseI BsePI
 ApoI Tru1I

 EcoT14I BshNI Hsp92I BbeI ErhI MaeI NdeII DpnI RsaI
 StyI CfrI BanI Msp17I Bsp143II EcoT14I Sau3AI AlwI BstSFI
 Eco130I BglI Hin1I AcyI AvrII BlnI DpnII MflI AclWI AluI
agtgggccgccttggcggccataaaggcgccaacaaacctaggatcttgtgtactcacgccctcccggtgtagct base pairs
tcacccggcggaaccgccggtatttccgcggttgtttggatcctagaacacatgagtgcgggagggccacatcga 28726 to 28800
 ErhI EaeI Eco64I BsaHI BstH2I BssT1I BstX2I Kzo9I SfcI
 BssT1I KasI BbiII EheI Eco130I BstYI Bsp143I Csp6I
 BglI AccB1I NarI HaeII StyI BfaI MboI XhoII AfaI

 Bst71I AvaII BsrGI BsmAI
 Bme18I Csp6I CfrI
 PstI SinI BsmFI AfaI HgaI Esp3I
gcagggtctggtccctgtacacctcggcccggaggtgcgtctcggccaaacgtcggcgcagggccgcgtggctgg base pairs
cgtcccagaccagggacatgtggagccgggcctccacgcagagccggtttgcagccgcgtcccggcgcaccgacc 28801 to 28875
 AlwNI SspBI EaeI MaeII
 BbvI HgiEI Bsp1407I Alw26I
 Eco47I RsaI BsmBI

BsaHI BsmBI MaeII HinfI
BbiII BsmAI BbiII AatII
Hin1I Alw26I BssHII Hin1I AcyI DdeI
cgtctcggctcatctcgccgcccccgcgcgcgcccgacgtcggactccttcgccccgacccccctgacctcagcc base pairs
gcagagccgagtagagcggcgggggcgcgcgcgggctgcagcctgaggaagcggggctggggggactggagtcgg 28876 to 28950
Msp17I Esp3I BsePI Msp17I PleI BstDEI
Hsp92I Hsp92I
AcyI HgaI BsaHI

 BalI AluI Van91I BbiII BsgI AfaI MvaI HaeII MvaI
 MscI PvuII MspA1I BsaHI Bst71I BstNI EcoRII Bst2UI
 EaeI AlwNI NspBII Hsp92I Csp6I BstOI Bsp143II
gcccccgcctcgcccgcgatgtttggccagcagctggcgtccgacgtgcagcagtacctggagcgcctggagaaa base pairs
cgggggcggagcgggcgctacaaaccggtcgtcgaccgcaggctgcacgtcgtcatggacctcgcggacctcttt 28951 to 29025
 CfrI AccB7I Bst71I AcyI BbvI EcoRII BpmI BstOI
 MluNI PflMI BbvI Msp17I MaeII AlwNI GsuI BstNI GsuI
 Esp1396I Hin1I HgaI RsaI Bst2UI BstH2I BpmI

 BsaHI TaqI EclHKI Hsp92I
 BbiII AccI AhdI Msp17I Bst71I
 Hin1I TthHB8I AspEI BsaHI HgaI SfaNI HgaI
cagaggcaacagaaggtgggcgtcgacgaggcgtcggcgggcctgacgctcggcggcgatgcgctgcgcgtccct base pairs
gtctccgttgtcttccacccgcagctgctccgcagccgcccggactgcgagccgccgctacgcgacgcgcaggga 29026 to 29100
 Msp17I HgaI Eam1105I HgaI BbvI BsmFI
 Hsp92I HincII Hin1I AcyI
 AcyI SalI HindII BbiII

 BsaHI Esp1396I Eco47I XmaI Msp17I BsmFI
 BbiII BstH2I DsaI PshAI Ama87I BsoBI BbiII HgaI
 Hin1I Bsp143II AccB7I HgiEI PspAI PspALI AcyI
tttttggattttgccaccgcgacgcccaagcgccaccagaccgtggtcccgggcgtcgggacgctccacgactgc base pairs
aaaaacctaaaacggtggcgctgcgggttcgcggtggtctggcaccagggcccgcagccctgcgaggtgctgacg 29101 to 29175
 Msp17I HaeII BstDSI SinI BcoI AvaI Hin1I HgaI
 Hsp92I PflMI Bme18I Cfr9I BsmFI Hsp92I
 AcyI HgaI Van91I AvaII Eco88I SmaI BsaHI

 BmyI SapI EaeI Eco52I MvaI BshNI NgoAIV
 Bsp1286I BsrBI EarI CfrI Bsh1285I Tru1I BstOI MroNI Bse118I
 BbvI SduI BsiHKAI Eam1104I XmaIII BsaOI Tru9I EcoRII BanI BsrFI BstDEI
tgcgagcactcgccgctcttctcggccgtcgcgcggcggttgctgtttaatagcctggtgccggcgcaactcagg base pairs
acgctcgtgagcggcgagaagagccggcagcgcgccgccaacgacaaattatcggaccacggccgcgttgagtcc 29176 to 29250
 Bst71I Alw21I AccBSI MboII EclXI MseI BstNI AccB1I Cfr10I
 AspHI BstD102I BstZI BsiEI Bst2UI BssAI NaeI
 Bbv12I Ksp632I EagI BstMCI Eco64I NgoMI DdeI

 BstOI BsoBI
 EcoRII Eco88I BsePI
 Tsp45I EaeI AluI GsuI MvaI Ama87I AscI
gggcgtgactttgggggcgaccacacggccaagctggagttcctggcccccgagctggtgcgggcggtggcgcgc base pairs
cccgcactgaaacccccgctggtgtgccggttcgacctcaaggaccgggggctcgaccacgcccgccaccgcgcg 29251 to 29325
 MaeIII CfrI BpmI BcoI AluI BssHII
 BstNI AvaI
 Bst2UI

 BsaHI
 BbiII BmyI
 BssHII Hin1I SduI SfcI HgaI AflIII
ctgcggtttcgggagtgcgcgccggaggacgccgtgccccaacgcaacgcctactacagcgtcctgaacacgttt base pairs
gacgccaaagccctcacgcgcggcctcctgcggcacggggttgcgttgcggatgatgtcgcaggacttgtgcaaa 29326 to 29400
 BsePI Msp17I Bsp1286I BstSFI MaeII
 Hsp92I
 AcyI HgaI


 AlwNI BstD102I Bse1I
 EcoO109I BsgI AccBSI DraIII BsmFI BsrSI
caggccctgcaccgctccgaagcctttcggcagttggttcacttcgtgcgggacttcgcccagttgttgaaaacc base pairs
gtccgggacgtggcgaggcttcggaaagccgtcaaccaagtgaagcacgccctgaagcgggtcaacaacttttgg 29401 to 29475
 DraII BsrBI Asp700I BseNI
 XmnI BsrI


 EcoO109I ApaI EcoT14I MluNI
 Bsp120I FriOI StyI CfrI
 MaeI Alw26I Alw26I SduI BanII Eco130I MaeII BstDSI
tcgttccgggcctctagtctcgcggagactacgggccccccgaagaaacgggccaaggtggacgtggccacccac base pairs
agcaaggcccggagatcagagcgcctctgatgcccggggggcttctttgcccggttccacctgcaccggtgggtg 29476 to 29550
 BfaI BsmAI BsmAI Bsp1286I MboII ErhI XcmI EaeI DsaI
 PspOMI BmyI BssT1I MscI
 DraII Eco24I BalI

 BsiWI BanI StyI AluI AspHI FriOI Eam1104I
 PspLI Eco64I BssT1I Bsp1286I BanII Ksp632I
 MaeII Csp6I ErhI Ecl136II Eco24I SstI EarI EaeI
gggcagacgtacggcaccttggagctcttccagaaaatgatactaatgcacgcgacctactttctggccgccgtg base pairs
cccgtctgcatgccgtggaacctcgagaaggtcttttactatgattacgtgcgctggatgaaagaccggcggcac 29551 to 29625
 SplI RsaI AccB1I EcoICRI Psp124BI SapI CfrI
 Pfl23II BshNI EcoT14I BmyI SacI BsiHKAI
 SunI AfaI Eco130I SduI Bbv12I Alw21I MboII

 BbvI Bme18I MaeII DpnII MflI AclWI XmaIII
 Eco88I Eco47I HindII TthHB8I Sau3AI AlwI CfrI
 Ama87I AvaII BspMI BsiI BstYI Bsp143I EaeI
ctgctcggggaccacgcggagcaggtcaacacgttcctgcggctcgtgttcgagatccccctgtttagcgacacg base pairs
gacgagcccctggtgcgcctcgtccagttgtgcaaggacgccgagcacaagctctagggggacaaatcgctgtgc 29626 to 29700
 BcoI Bst71I HincII BssSI NdeII XhoII BstZI
 AvaI SinI BsmFI AflIII TaqI MboI Kzo9I EagI
 BsoBI HgiEI BstX2I DpnI EclXI

 BsaOI Bst2UI Hin1I AcyI BbeI SexAI Eco64I
 MslI BstNI BshNI BsaHI HaeII EcoRII
 Eco52I BssHII MaeI EcoRII Eco64I Hsp92I BstDSI BstOI
gccgtgcggcacttccgccagcgcgccaccgtgtttctagtccccaggcgccacggaaagacctggtttttggtg base pairs
cggcacgccgtgaaggcggtcgcgcggtggcacaaagatcaggggtccgcggtgcctttctggaccaaaaaccac 29701 to 29775
 Bsh1285I BsePI BfaI BsmFI BanI Msp17I Bsp143II Bst2UI
 BsiEI BstOI AccB1I NarI BstH2I BstNI
 BstMCI MvaI KasI BbiII EheI DsaI MvaI

 Bsp143I
 BanI BmyI MboI DpnI BstMCI
 SduI HgaI DpnII AclWI BstF5I BsiEI
cccctcatcgcgctgtcgctcgcgtccttccgggggatcaagataggctacacggcccacatccgcaaggcgacc base pairs
ggggagtagcgcgacagcgagcgcaggaaggccccctagttctatccgatgtgccgggtgtaggcgttccgctgg 29776 to 29850
 Bsp1286I NdeII AlwI FokI Bsh1285I
 BshNI Sau3AI BsaOI
 AccB1I Kzo9I

 BmyI Bsp143I BbiII Eco88I SinI Tth111I
 FriOI MboI DpnI Hsp92I PspAI PspALI AspI
 SduI DpnII TthHB8I HgaI Ama87I SmaI AvaII
gagcccgtgtttgatgagatcgacgcctgcctgcggggctggtttggctcgtcccgggtggaccacgtcaagggg base pairs
ctcgggcacaaactactctagctgcggacggacgccccgaccaaaccgagcagggcccacctggtgcagttcccc 29851 to 29925
 BanII NdeII TaqI BsaHI BglI BcoI XmaI Bme18I AtsI
 Bsp1286I Sau3AI Msp17I Cfr9I BsmFI Eco47I
 Eco24I Kzo9I Hin1I AcyI AvaI BsoBI HgiEI MaeII

 MboI MslI Ple19I
 DpnII DpnI BstMCI BpmI
 BbvI Bsp143I Bsh1285I MaeII
gaaaccatctcgttctcgttcccggacggctcgcgcagcacgatcgtgtttgcctccagccacaacacgaacgta base pairs
ctttggtagagcaagagcaagggcctgccgagcgcgtcgtgctagcacaaacggaggtcggtgttgtgcttgcat 29926 to 30000
 Bst71I Kzo9I PvuI GsuI
 NdeII BspCI BsaOI
 Sau3AI BsiEI BcgI

 KspI SacII BsoBI Sau3AI Bsh1285I
 AfaI NspBII Eco64I BshNI Eco88I Kzo9I BsiEI
 Csp6I BstDSI Cfr42I Eco64I Ama87I Bsp143I BstMCI
agtacgccttcctcccgcggtgcctgtttccccggtgccgccctccccgagatcgaccgacagacaaacacagcc base pairs
tcatgcggaaggagggcgccacggacaaaggggccacggcgggaggggctctagctggctgtctgtttgtgtcgg 30001 to 30075
 RsaI DsaI SstII BshNI BanI BcoI NdeII TaqI
 MspA1I BanI AccB1I AvaI MboI DpnI BsaOI
 Sfr303I AccB1I DpnII TthHB8I

 NcoI Bsp19I
 StyI DsaI
 HgaI BsmFI BbvI Eco130I Hsp92II
agacgcgagtgtgggacgacacgcccgcagccccccccccgccatggcgggggggaagccttactgtttatttgt base pairs
tctgcgctcacaccctgctgtgcgggcgtcggggggggggcggtaccgcccccccttcggaatgacaaataaaca 30076 to 30150
 Bst71I ErhI BstDSI
 BssT1I NlaIII
 EcoT14I

 BalI BstMCI SstII Bst71I
 MscI BstDSI Sfr303I
 EaeI DsaI BsiEI MspA1I BbvI AccI
aatcggacgatgaggctctggccacggcccgcgcgaccgcggggcagctcgttgcaaacaggcggctggtatacg base pairs
ttagcctgctactccgagaccggtgccgggcgcgctggcgccccgtcgagcaacgtttgtccgccgaccatatgc 30151 to 30225
 CfrI Bsh1285I Cfr42I Bst1107I
 MluNI DsaI NspBII AluI
 BstDSI BsaOI KspI SacII

 AccB1I NarI HaeII
 KasI BbiII EheI HaeII HgaI
 Eco64I BsaHI BbeI BstH2I BstF5I BglI
atgacagaacgcagaggcgccacccggcgctggtcgggcggatgacgctttccgcgccgtcccggcccacgacga base pairs
tactgtcttgcgtctccgcggtgggccgcgaccagcccgcctactgcgaaaggcgcggcagggccgggtgctgct 30226 to 30300
 BanI Msp17I Bsp143II FokI BsmFI
 BshNI Hsp92I Bsp143II
 Hin1I AcyI BstH2I

 BshNI BmyI
 BssSI DsaI BanI Bsp1286I
 EcoNI BspMI SfaNI EcoNI HgaI Eco64I
cctcgtgcaggtgggccgtgatgcgcgggcggcgggtcgcctgccgcaggataaccgcgtccacggggtgcccga base pairs
ggagcacgtccacccggcactacgcgcccgccgcccagcggacggcgtcctattggcgcaggtgccccacgggct 30301 to 30375
 BsiI BstDSI SduI
 BsgI DraIII
 AccB1I

 EarI BstOI Eco47I
 BseRI EcoRII HgiEI NspI
 MboII HgaI EaeI MvaI SinI NlaIII
agaggagctgacacaggctcgcgtccccccggacggccagggtgcgctgggccatattggaccacatgcacgggg base pairs
tctcctcgactgtgtccgagcgcaggggggcctgccggtcccacgcgacccggtataacctggtgtacgtgcccc 30376 to 30450
 Eam1104I BsmFI CfrI Bme18I Hsp92II
 Ksp632I BstNI AvaII
 AluI Bst2UI

 Eco147I BspDI BscI
 StuI BsePI TthHB8I Bsu15I
 HgaI BsmFI BstDSI AscI HinfI BspXI ClaI BglI
cgacgcagggacaggcctccgccacggcgggggcgcgccacagcgcgttggcggaatcgatgtgggccgtcgggg base pairs
gctgcgtccctgtccggaggcggtgccgcccccgcgcggtgtcgcgcaaccgccttagctacacccggcagcccc 30451 to 30525
 Pme55I DsaI BssHII TfiI Bsa29I
 AatI BanIII TaqI
 SseBI Bsp106I BseCI

 AccB1I NarI HaeII Cfr9I PspALI MvaI BmyI Ama87I
 KasI BbiII EheI BcoI XmaI EcoRII FokI SduI ApaI
 Eco64I BsaHI BbeI PspAI BsoBI BstNI BbvI BstF5I Bsp120I BanII
cgcaggcgccgcctcctcccggggggtcggtaatcctggatagcagccatcctaaatggcgggcccggctgcccg base pairs
gcgtccgcggcggaggagggccccccagccattaggacctatcgtcggtaggatttaccgcccgggccgacgggc 30526 to 30600
 BanI Msp17I Bsp143II AvaI SmaI BstOI Bst71I PspOMI FriOI PspAI
 BshNI Hsp92I Ama87I BseRI Bst2UI Bsp1286I Cfr9I
 Hin1I AcyI BstH2I Eco88I Eco24I BcoI

 XmaI BstOI StyI DsaI Hsp92II EagI Eco52I BshNI MvaI
AvaI Bst71I EclHKI ErhI BstDSI CfrI EclXI BsaOI BstOI
Eco88I SmaI EcoRII Bst2UI NcoI Bsp19I EaeI XmaIII Eco64I BstNI
ggggacagagcgaccccaggtcatcatccatggcccagcagtatatgcggccgccggggaggtgccaccaggccc base pairs
cccctgtctcgctggggtccagtagtaggtaccgggtcgtcatatacgccggcggcccctccacggtggtccggg 30601 to 30675
 BsoBI AhdI MvaI Eco130I FokI BstZI Bsh1285I EcoRII DraII
 PspALI BstNI AspEI EcoT14I NlaIII CciNI BstMCI BanI Bst2UI
 BbvI BsmFI Eam1105I BssT1I BstF5I NotI BsiEI AccB1I EcoO1

 AvaII Bst2UI Acc65I SexAI MvaI AccB1I AcyI
 Bme18I MvaI BanI Csp6I BstNI KasI Hsp92I
 SinI BstNI SduI HinfI BstDSI AccB1I KpnI Eco64I BbiII
ccggacccagggcacagcacgccccggattcgggggccgtgtccgtgggtaccaggtaggcgccgtcgagctcgt base pairs
ggcctgggtcccgtgtcgtgcggggcctaagcccccggcacaggcacccatggtccatccgcggcagctcgagca 30676 to 30750
 Eco47I Bsp1286I TfiI DsaI Eco64I AfaI Bst2UI Hin1I NarI
 HgiEI BstOI Asp718I EcoRII BanI Msp17I
 09I EcoRII BmyI BshNI RsaI BstOI BshNI BsaHI

 TthHB8I AspHI SacI SstI DsaI FriOI BstOI
 HaeII AluI BmyI FriOI BsiI Bsp1286I Bst71I
EheI TaqI Bsp1286I BanII BssSI BmyI AluI BstNI
gggccacgggctcgtccgcgagctgttcggcggcggggtcgggggtttcctccgggggggaggcagcttccaggt base pairs
cccggtgcccgagcaggcgctcgacaagccgccgccccagcccccaaaggaggccccccctccgtcgaaggtcca 30751 to 30825
 BbeI Ecl136II Eco24I BsiHKAI Eco24I BbvI MvaI
Bsp143II EcoICRI Psp124BI BstDSI BanII EcoRII
 BstH2I SduI Bbv12I Alw21I SduI AluI Bst2UI

 Bsp1286I Bst71I
EaeI ApaLI Bbv12I BbvI Eco47I
 MaeI Alw44I Alw21I SinI MaeIII BbvI
ggccgaaggctagggtgcacagcagcggggtccgggggtgcgttacgctgcggaggtggacggtggcgcagtagc base pairs
ccggcttccgatcccacgtgtcgtcgccccaggcccccacgcaatgcgacgcctccacctgccaccgcgtcatcg 30826 to 30900
 BfaI VneI BmyI NspBII Bme18I Bst71I
 CfrI SduI BsiHKAI HgiEI
 AspHI MspA1I AvaII

 SfuI TaqI HaeII BssT1I
 BstH2I MseI Asp700I Bsp119I Bsp143II
 Bsp143II MboII MaeII TthHB8I BbvI ErhI HgaI
ggcgctcgcggttaaagaagaaaatggcaaagaacgtgttcgaaggcaggcgcagcgccttgggccgcgtcaggt base pairs
ccgcgagcgccaatttcttcttttaccgtttcttgcacaagcttccgtccgcgtcgcggaacccggcgcagtcca 30901 to 30975
 HaeII Tru9I AflIII Csp45I Bst71I StyI Csp6I
 Tru1I XmnI BstBI Bpu14I Eco130I
 LspI NspV BstH2I EcoT14I

 MboI Bsp143I BcoI Eco47I BseAI Bst2UI
RsaI BstX2I Kzo9I BmyI BsoBI HgiEI MroI BsiMI
 BstYI MflI MboII SduI AvaI SinI AccIII EcoRII NspBII
acaggaagatctcgcagaaaagggcacgctcggggtcggggtccggaagggccacctggcacagcggctcggtga base pairs
tgtccttctagagcgtcttttcccgtgcgagccccagccccaggccttcccggtggaccgtgtcgccgagccact 30976 to 31050
 DpnII BglII Bsp1286I Bme18I BsaWI BstNI MspA1I
AfaI NdeII XhoII Ama87I AvaII Kpn2I BstOI
 Sau3AI DpnI Eco88I BspEI Bsp13I MvaI

 AvaII MspCI BpiI
 Bme18I BshNI Bst98I BsrBI Bbv16II
HphI Eco64I BspTI MseI AccBSI HgaI MboII
ggaccgtgaggcaccgaaaaatcttaagccgctcgtccccccgaacgacgcgccacacgaagacagagttggcga base pairs
cctggcactccgtggctttttagaattcggcgagcaggggggcttgctgcgcggtgtgcttctgtctcaaccgct 31051 to 31125
 SinI BanI AflII Tru1I BsmFI BpuAI
 HgiEI AccB1I Vha464I BstD102I BbsI
 Eco47I BfrI Tru9I

 Ama87I Eco24I ApaI AccB1I NarI HaeII EcoO65I
 SfaNI AtsI EcoO109I Cfr9I XmaI SmaI KasI BbiII EheI BstEII
 BssHII Tth111I Bsp120I BcoI AvaI BsoBI HgaI Eco64I BsaHI BbeI BstPI
tgcgcgcgacgaggtcggcttcgggccccgggtcgggggcgcgcgcgtcggggggggcgccccggtgacccggcg base pairs
acgcgcgctgctccagccgaagcccggggcccagcccccgcgcgcgcagccccccccgcggggccactgggccgc 31126 to 31200
 BsePI AspI PspOMI Bsp1286I BanII BsePI BanI Msp17I Bsp143II
 DraII BmyI Eco88I PspALI BshNI Hsp92I Eco91I
 SduI PspAI FriOI BssHII Hin1I AcyI BstH2I

 NspBII Ama87I BsoBI BstNI Msp17I BstNI Msp17I SduI BstOI
 HphI Sfr303I PspAI PspALI Bst2UI AcyI Bst2UI AcyI BmyI BstNI
 BstDSI Cfr42I Eco88I EcoRII Hin1I HgaI Hin1I BsmFI MspA1I XcmI
gggccgcggctcccggggggcctggcgtcgcctggggacgccagagtgcccgctgtgccaggttggtggtgggga base pairs
cccggcgccgagggccccccggaccgcagcggacccctgcggtctcacgggcgacacggtccaaccaccacccct 31201 to 31275
 MaeIII KspI SacII AvaI EcoO109I BbiII EcoRII BbiII HgaI EcoRII BstXI
 Tsp45I MspA1I BcoI XmaI DraII MvaI BsaHI MvaI Hsp92I NspBII Bst2UI
PspEI DsaI SstII Cfr9I SmaI BstOI Hsp92I BstOI BsaHI Bsp1286I MvaI

 Eco47I HgaI
 HgiEI BsmAI BsrBI
 SinI BsmFI BsmBI HphI
agggaccggagacgcaccaaaagcagaggggccagcgcgtgtatgagttggggggggggtgggtgagcggtggaa base pairs
tccctggcctctgcgtggttttcgtctccccggtcgcgcacatactcaaccccccccccacccactcgccacctt 31276 to 31350
 Bme18I Alw26I AccBSI
 AvaII Esp3I BstD1
 BsaWI

 EcoO109I
 MspA1I PpuMI Psp5II
 AflIII NspBII SinI AvaII BsaWI PleI
caaaagcacgcgtcagcggacaaggccgggtcccgtagccgccccgcgacagaaccggagtccgacggcacgcgc base pairs
gttttcgtgcgcagtcgcctgttccggcccagggcatcggcggggcgctgtcttggcctcaggctgccgtgcgcg 31351 to 31425
 MluI HgaI Bme18I HinfI
02I HgiEI Eco47I
 DraII BsmFI

 DsaI SstII Tru1I
 AtsI RsaI MspA1I MseI
 Tth111I DdeI Csp6I NspBII Tru9I MaeI
gacggggtctgcgaggctgaggtacgccgcggtgttaatggtaaacgcaaagcctcccggaaagaccactagccc base pairs
ctgccccagacgctccgactccatgcggcgccacaattaccatttgcgtttcggagggcctttctggtgatcggg 31426 to 31500
 AspI BstDEI BstDSI Cfr42I BfaI
 AfaI KspI SacII
 Sfr303I

 EcoT14I MluI
 StyI AfaI
 Eco130I Csp6I AluI AlwNI
gcagaggcggcgattgaacccaaggcagaggtacgcgtagctctctcccggaaggtattgctcgcagaccctgtg base pairs
cgtctccgccgctaacttgggttccgtctccatgcgcatcgagagagggccttccataacgagcgtctgggacac 31501 to 31575
 ErhI RsaI
 BssT1I AflIII


 MaeII MaeIII
 BbiII Tsp45I
 TspRI BbvI NlaIII HgaI Hin1I AcyI
tggggcagtggaggggctgccctccatgaagcgacatttactctgctcgcgtccattgacgtcaccgtcaatcac base pairs
accccgtcacctccccgacgggaggtacttcgctgtaaatgagacgagcgcaggtaactgcagtggcagttagtg 31576 to 31650
 Bst71I Hsp92II Msp17I DrdI
 Hsp92I HphI
 BsaHI AatII

 BsaHI
 NspBII BbiII
 HphI HphI BbvI Alw26I SfcI AflIII Hin1I
cactgcgattggacggttggtgaggcgcagcgtgtctccgctggtgctgtagtagtcaaacgcgtagtgggcgtc base pairs
gtgacgctaacctgccaaccactccgcgtcgcacagaggcgaccacgacatcatcagtttgcgcatcacccgcag 31651 to 31725
 TspRI Bst71I BsmAI BstSFI MluI Msp17I
 MspA1I Hsp92I
 AcyI

 EcoRII
 BstMCI MvaI
 HinfI BstF5I DdeI BsmFI BsiEI BstOI
ggagtcggcgaagcgggcggggatgtcgtcgctgagagggacgagccgccgccgccgcccccgaccgccctggcc base pairs
cctcagccgcttcgcccgcccctacagcagcgactctccctgctcggcggcggcggcgggggctggcgggaccgg 31726 to 31800
 PleI FokI BstDEI Bsh1285I EaeI
 BsaOI Bst2UI
 HgaI BstNI

CfrI BstOI SunI Eco47I Eco88I BssHII
 CfrI SplI RsaI HgiEI Sfr274I TaqI
 SfaNI BglI BstNI Pfl23II AflIII SinI BsmFI AvaI BsePI
gcccagatgcgccagcacggccagggcgtacgcggtgtgaaagaacgcgtcgggggcggtcccctcgagggcgcg base pairs
cgggtctacgcggtcgtgccggtcccgcatgcgccacactttcttgcgcagcccccgccaggggagctcccgcgc 31801 to 31875
 EaeI MvaI BsiWI MluI HgaI Bme18I BcoI BsoBI
 EcoRII PspLI AfaI AvaII Ama87I TthHB8I
 Bst2UI Csp6I XhoI PaeR7I

 MvaI BmyI MaeIII
 BstOI Bsp1286I HaeII HgaI
 EcoRII BpmI Alw21I Bsp143II HphI MaeI
catcaggttctccaggagcacggggaagcgccgcgtcacctcccctagccactcgctctggtgggggccaaagtc base pairs
gtagtccaagaggtcctcgtgccccttcgcggcgcagtggaggggatcggtgagcgagaccacccccggtttcag 31876 to 31950
 SfaNI BstNI SduI BsiHKAI Tsp45I BfaI
 Bst2UI AspHI BstH2I
 GsuI Bbv12I

 EcoT14I MvaI EcoO109I
 HaeII StyI BstOI SduI Eco24I
 AlwNI MboII Eco130I EcoRII PspOMI FriOI
gtagcgcaggcgctggaagatgcgcgggccgccttggagcgcggcccggatagagtggcccagggcccgcagaca base pairs
catcgcgtccgcgaccttctacgcgcccggcggaacctcgcgccgggcctatctcaccgggtcccgggcgtctgt 31951 to 32025
 Bsp143II SfaNI ErhI BstNI DraII BanII
 BstH2I BssT1I Bst2UI Bsp1286I
 Bsp120I BmyI

ApaI Kzo9I BsePI CfrI BsiEI PstI
 Bsp143I FokI EaeI Eco52I BbvI
 MboI DpnI SfaNI SfiI XmaIII SfcI BbvI
cgcgatctggatgcgcgcgacgaaggccacctcggccgcgatgtcaaagggctgcagcacggggcgcgggtggcg base pairs
gcgctagacctacgcgcgctgcttccggtggagccggcgctacagtttcccgacgtcgtgccccgcgcccaccgc 32026 to 32100
 Sau3AI BstF5I BglI EclXI BsaOI BstSFI
 DpnII BssHII BstZI BstMCI Bst71I
 NdeII BcgI EagI Bsh1285I Bst71I

 EcoO109I XhoI BsoBI KspI SacII
 PpuMI Psp5II Eco88I Bst71I BglI NspBII BsmFI
 SinI AvaII BcoI TthHB8I HgaI Bsp143II BstDSI Cfr42I MspA1I
caggggtccctcgagcgcgggaaagcgacgcagcagcgccgtctgggccgcgggggacagctggtgggggcgcac base pairs
gtccccagggagctcgcgccctttcgctgcgtcgtcgcggcagacccggcgccccctgtcgaccacccccgcgtg 32101 to 32175
 Bme18I Ama87I PaeR7I BbvI HaeII DsaI SstII PvuII
 HgiEI Eco47I AvaI TaqI BstH2I MspA1I AluI
 DraII Sfr274I BsmFI Sfr303I NspBII

 SseBI MluNI KspI SacII
 AatI EaeI NspBII
 HgaI Pme55I BstDSI AluI BstDSI Cfr42I HgaI
gacgcgctcggcggcacaggcctccgtcagggccgtggccagctcggaggacagccgcggggggcgggcgcgtcg base pairs
ctgcgcgagccgccgtgtccggaggcagtcccggcaccggtcgagcctcctgtcggcgccccccgcccgcgcagc 32176 to 32250
 StuI EcoNI DsaI MscI DsaI SstII
 Eco147I CfrI MspA1I
 BalI Sfr303I

 ApoI HaeII SinI BsmFI Eco47I
 EcoRI BsmBI AfeI BbvI AvaII HgiEI
 Sse9I Alw26I Aor51HI Bst71I SinI
cccgccccacgccaccgaattctcgtaggagacgacgacgaagcgctgcttggtcccgtagtgatggcgcaggac base pairs
gggcggggtgcggtggcttaagagcatcctctgctgctgcttcgcgacgaaccagggcatcactaccgcgtcctg 32251 to 32325
 AcsI BsmAI Eco47III Bme18I Bme18I
 TspEI Esp3I Bsp143II Eco47I AvaII
 Tsp509I BstH2I HgiEI

 BglI Bsh1285I Bse118I KspI SacII
 Bse1I BsiEI NgoMI CfrI NspBII SinI
 DsaI BstXI BsrSI Bse118I MroNI Cfr10I BstDSI Cfr42I
cacggagatggagcgacggctccacagccagtcgggccggtcgccgccggccagagcttcccacccgcggtccag base pairs
gtgcctctacctcgctgccgaggtgtcggtcagcccggccagcggcggccggtctcgaagggtgggcgccaggtc 32326 to 32400
 MslI BseNI Cfr10I BssAI EaeI DsaI SstII
 BsrI BsrFI BsaOI BsrFI NaeI MspA1I Bme18I
BstDSI BssAI BstMCI NgoAIV AluI Sfr303I

 TaqI Bsp143I Bsh1285I AvaII BmyI BsiHKAI BsaHI
 TthHB8I MboI DpnI PvuI Bme18I BssSI Bsp1286I BbiII AatII MspA1I
 Eco47I DpnII BspCI BsaOI Eco47I MslI HphI MaeII Hin1I AcyI NspBII
ccactcgaccagcgatcgcggcttggcggtccccggcacgagggtgagcacgtcgttgaggacgtcctcgcccgc base pairs
ggtgagctggtcgctagcgccgaaccgccaggggccgtgctcccactcgtgcagcaactcctgcaggagcgggcg 32401 to 32475
 HgiEI NdeII BsiEI SinI BsmFI BsiI SduI Alw21I Msp17I BstDSI
 Sau3AI Ple19I HgiEI AspHI Hsp92I DsaI
 AvaII Kzo9I SgfI BstMCI Bbv12I MaeII

KspI SacII XmaI PspOMI BmyI BglI MvaI
 BcoI AvaI Bsp120I Eco24I HaeII BstOI
 Ama87I BsoBI DraII BanII Bsp143II EcoRII BpmI
ggcccgggggcccccccggctggcaaagcgccccccgccgggcggctccaggcccgccagcaccgcctccgcgtc base pairs
ccgggcccccgggggggccgaccgtttcgcggggggcggcccgccgaggtccgggcggtcgtggcggaggcgcag 32476 to 32550
 Cfr42I Eco88I EcoO109I ApaI BstNI
 SstII PspAI PspALI Bsp1286I BstH2I Bst2UI
Sfr303I Cfr9I SmaI SduI FriOI GsuI

 Bst2UI BanII MvaI Bme18I MvaI BshNI BsaHI BstH2I Ama87I AvaI
 BstNI BmyI MspA1I BstOI Eco47I Eco64I BbiII EheI BstNI Bsp1286I
HgaI HgaI SduI NspBII EcoRII SinI BstNI KasI Hsp92I HaeII BstOI BmyI
cgacgcgcccagggctcccccgctgacggcctggtggaccagggcgccctggcggagccccgaggcgacgccgga base pairs
gctgcgcgggtcccgagggggcgactgccggaccacctggtcccgcgggaccgcctcggggctccgctgcggcct 32551 to 32625
 EcoRII Bsp1286I BstNI AvaII Bst2UI Hin1I AcyI EcoRII SduI Eco88I
 BstOI Eco24I Bst2UI EcoRII BanI Msp17I Bsp143II MvaI BcoI
 MvaI FriOI HgiEI BstOI AccB1I NarI BbeI Bst2UI Eco24I

 Hin1I HgaI CspI BbiII AcyI HgiEI
 BsoBI AcyI Bme18I Hin1I HphI SinI
 FriOI Hsp92I BssHII CpoI AvaII Tsp45I MaeII BsgI NspBII Eco47I
ggccgcgtgcttggggcgcgcgcggaccgggtggcggcgggtgacgtcctgcacggcccgctggaccagcgcgag base pairs
ccggcgcacgaaccccgcgcgcgcctggcccaccgccgcccactgcaggacgtgccgggcgacctggtcgcgctc 32626 to 32700
 BbiII BsePI SinI Eco47I MaeIII BsaHI MspA1I
 BanII BsaHI HgiEI Msp17I AatII Bme18I
 Msp17I RsrII Hsp92I AvaII

MboI Bsp143I KspI SacII Eco88I
BstX2I DpnI NspBII EaeI PspAI PspALI Bst71I
BstYI MflI AlwI AflIII BstDSI Cfr42I Ama87I AluI
gatctcctcgttctcttgcgtgatggacacgtcctccgcggtggccgtgtcgcctcccggggccgtgagctgctc base pairs
ctagaggagcaagagaacgcactacctgtgcaggaggcgccaccggcacagcggagggccccggcactcgacgag 32701 to 32775
DpnII XhoII BseRI MaeII DsaI SstII BcoI XmaI BbvI
NdeII Kzo9I MspA1I CfrI Cfr9I SmaI
Sau3AI AclWI Sfr303I AvaI BsoBI

 XmaIII NgoAIV BsaOI Ama87I Eco31I AcyI
 BshNI CfrI MroNI Bse118I SfiI BsoBI Msp17I HgaI
 BseRI Eco64I EaeI Eco52I BsiEI NaeI Eco88I Hin1I EcoRII
ctccggggagatgggggggtctggggtgccgacaacggccggcccggccccgcccgagaccgaggacgcctgggg base pairs
gaggcccctctacccccccagaccccacggctgttgccggccgggccggggcgggctctggctcctgcggacccc 32776 to 32850
 BanI BstZI NgoMI Bsh1285I BcoI BsmAI Hsp92I
 AccB1I EagI BssAI Cfr10I FseI AvaI BsaI BsaHI
 EclXI BsrFI BstMCI BglI Alw26I BbiII BstNI

MvaI BstOI KspI
 BshNI EcoRII NspBII
 Eco64I BstF5I BsmFI BstDSI
agtgggggtgccgctttcccccatccccagggacaggtgggccgccgcctccgtcgcggcggcgggagccgcggc base pairs
tcacccccacggcgaaagggggtaggggtccctgtccacccggcggcggaggcagcgccgccgccctcggcgccg 32851 to 32925
BstOI BanI FokI MvaI DsaI SstII
Bst2UI AccB1I BstNI MspA1I
 Bst2UI Sfr3

 SacII XmaIII
 BssHII CfrI BsiEI
 Cfr42I MaeII NlaIII EaeI Eco52I
ccccagccgcgcgacgtagcgacaaaagtggcgacagaggcgcatgaggcgcgcgccgtcggccgcgtatcgcgt base pairs
ggggtcggcgcgctgcatcgctgttttcaccgctgtctccgcgtactccgcgcgcggcagccggcgcatagcgca 32926 to 33000
 Hsp92II BstZI BstMCI
 BsePI EagI Bsh1285I
 03I EclXI BsaOI

 DrdI BmyI FriOI MaeIII Alw21I BmyI DraII Eco24I Bst2UI
 AluI AspHI SacI BsiHKAI Bbv12I SduI Bsp120I FriOI BstOI
 BsmFI Bsp1286I BanII SduI BsiHKAI BstDSI SduI BanII MvaI
gtttggcgggacgagctcgtcgtaactgaacaggagcacgcgggcacaggtcgcccacgggccccacgccaggcg base pairs
caaaccgccctgctcgagcagcattgacttgtcctcgtgcgcccgtgtccagcgggtgcccggggtgcggtccgc 33001 to 33075
 Ecl136II Psp124BI Bsp1286I DsaI EcoO109I ApaI
 EcoICRI Eco24I SstI AspHI Bsp1286I PspOMI BmyI EcoRII
 SduI Bbv12I Alw21I BmyI Bsp1286I BstNI

 BstH2I RsaI EcoT14I AcyI BsiEI
 Bsp143II Csp6I AfaI StyI Msp17I Bse118I BsmBI
 BbvI BsiEI AfaI Csp6I Eco130I BsaHI BssAI BstMCI Alw26I
cagcgccgcgaccgtgtacgggtcgtacacgccttgggcgtcgcacgcgaccggcagggagacgaacagcccgcc base pairs
gtcgcggcgctggcacatgcccagcatgtgcggaacccgcagcgtgcgctggccgtccctctgcttgtcgggcgg 33076 to 33150
 Bst71I Bsh1285I RsaI ErhI Hin1I HgaI BsrFI BsaOI BsmAI
 HaeII BstMCI BssT1I Hsp92I Cfr10I Esp3I
 BsaOI BbiII Bsh1285I

 Eco47I MboI DpnI BssAI
 HgiEI DpnII AclWI FokI
 BsmFI SinI BstF5I MaeI Bsp143I SfaNI
cgcgctggggacgcgcggcaggaggtccgggtgcgccgggatgacgggggctaggatcgcccccaccgcatccgc base pairs
gcgcgacccctgcgcgccgtcctccaggcccacgcggccctactgcccccgatcctagcgggggtggcgtaggcg 33151 to 33225
 HgaI Bme18I FokI BfaI Kzo9I BstF5I
 AvaII NdeII AlwI MroNI
 Sau3AI NgoMI

 NaeI Cfr10I PspAI BsoBI ApaI
 NgoAIV BsrFI BsaOI BcoI XmaI Eco24I TthHB8I
 BsrFI BsaAI BstDSI BsgI BsiEI Bsp120I AvaI BmyI PspALI
cggcacgtaggcggcaaacgccgaacgccacggggtgcagtcgccggtcgcgtgggcccgggtctgggtttcgac base pairs
gccgtgcatccgccgtttgcggcttgcggtgccccacgtcagcggccagcgcacccgggcccagacccaaagctg 33226 to 33300
 Bse118I DsaI DraIII BssAI BstMCI Ama87I Bsp1286I SmaI
 Cfr10I Bse118I PspOMI Eco88I FriOI TaqI
 MaeII Bsh1285I Cfr9I SduI BanII

 EagI Eco52I EagI Eco52I PpuMI Psp5II MvaI
 CfrI EclXI BsaOI CfrI EclXI BsaOI SinI AvaII BstOI
 EaeI XmaIII EaeI XmaIII BsiI NspBII EcoO109I EcoRII
ccggaagttcgcggccgccccaccgtcggggcggccgcgcacgagggcggacagcgggacccccgccgccgccag base pairs
ggccttcaagcgccggcggggtggcagccccgccggcgcgtgctcccgcctgtcgccctgggggcggcggcggtc 33301 to 33375
 BstZI Bsh1285I BstZI Bsh1285I MspA1I DraII BstNI
 CciNI BstMCI CciNI BstMCI BssSI Bme18I BsmFI Bst2UI
 NotI BsiEI NotI BsiEI HgiEI Eco47I

 BbvI Bme18I Psp5II Eco88I NdeII
 TfiI Eco88I EcoO109I BcoI BsoBI MboI
 XcmI GsuI MaeII Ama87I HgiEI Ama87I XmaI DpnII
gcactcgctggagatgatgacgtgaatcagcgaggcggggctgctcgggtcccgggtgagatcgtattggacctc base pairs
cgtgagcgacctctactactgcacttagtcgctccgccccgacgagcccagggcccactctagcataacctggag 33376 to 33450
 BpmI HinfI BcoI Bst71I AvaII Cfr9I BsmFI HphI
 AvaI SinI DraII PspAI PspALI Sau3AI
 BsoBI PpuMI Eco47I AvaI SmaI Bsp143I

 SinI CfrI MroNI Bse118I SduI Eco64I
 DpnI Eco47I EaeI Eco52I BsiEI BsaOI BanII
Kzo9I AvaII NlaIII XmaIII BsrFI BglI Bsp1286I BshNI
gttggcaaagtgcgcgttcatggcccggccggcggtgcgagcccttcccggtgccggaaggggcgtgggtggggg base pairs
caaccgtttcacgcgcaagtaccgggccggccgccacgctcgggaagggccacggccttccccgcacccaccccc 33451 to 33525
 Bme18I Hsp92II EclXI NgoAIV BstMCI FriOI AccB1I
 HgiEI BstZI NgoMI Bsh1285I BmyI BanI
 EagI BssAI Cfr10I NaeI Eco24I

 AvaI EcoO109I BstDSI Sfr303I PmaCI Bst2UI
 BcoI PspOMI Eco24I KspI SacII BbrPI BstNI
 HgaI Bsp120I BmyI DsaI SstII BsaAI EcoRII HinfI
gtgcgtgtgcgcgtcctcggggcccgcgggcgcacgtgcgcttatacgctgtgtgtttcgtctgtccccagggaa base pairs
cacgcacacgcgcaggagccccgggcgcccgcgtgcacgcgaatatgcgacacacaaagcagacaggggtccctt 33526 to 33600
 Ama87I SduI BanII MspA1I Eco72I BsmFI TfiI
 Eco88I Bsp1286I ApaI Cfr42I PmlI BstOI
 BsoBI DraII FriOI NspBII MaeII MvaI

 MvaI HincII Bme18I
 BstOI Tru1I AccI SfaNI
 EcoRII Tru9I BspMI SalI BstF5I Eco47I
tccggggccaggactttaacctgcttttcgtcgacgaggccaactttattcgcccggatgcggtccagacgatta base pairs
aggccccggtcctgaaattggacgaaaagcagctgctccggttgaaataagcgggcctacgccaggtctgctaat 33601 to 33675
 BstNI MseI TthHB8I FokI AvaII
 Bst2UI TaqI SinI
 HindII HgiEI

 Bsp143I Esp3I HindII
 MboI DpnI BsmAI TaqI AluI
 DpnII MboII SalI AccI BsiI
tgggctttctcaatcaggccaactgcaagatcatcttcgtctcgtcgaccaacaccgggaaggccagcacgagct base pairs
acccgaaagagttagtccggttgacgttctagtagaagcagagcagctggttgtggcccttccggtcgtgctcga 33676 to 33750
 NdeII Alw26I BssSI
 Sau3AI BsmBI HincII
 Kzo9I TthHB8I

 RsaI KspI SacII EcoO65I DsaI
 BsrGI NspBII Bst71I BstEII NspI
 SspBI BstDSI Cfr42I AluI MaeII Tsp45I NlaIII
ttttgtacaacctccgcggggccgccgacgagctgctcaacgtggtcacctatatatgcgacgaccacatgccgc base pairs
aaaacatgttggaggcgccccggcggctgctcgacgagttgcaccagtggatatatacgctgctggtgtacggcg 33751 to 33825
 Bsp1407I DsaI SstII BbvI Eco91I Hsp92II
 Csp6I MspA1I BstPI MaeIII BstDSI
 AfaI Sfr303I PspEI HphI NspBII

 KspI Tsp45I
 MspA1I MaeIII
 Sfr303I HgaI BstDSI
gggtggtgacgcacaccaacgccacggcctgttcctgctatatcctgaacaaacccgtgtttatcacgatggacg base pairs
cccaccactgcgtgtggttgcggtgccggacaaggacgatataggacttgtttgggcacaaatagtgctacctgc 33826 to 33900
 SstII HphI DsaI
 Cfr42I
 SacII

AccB1I NarI HaeII EagI BstMCI Sau3AI MboI DpnI
KasI BbiII EheI EaeI Eco52I NdeII HinfI DpnII
Eco64I BsaHI BbeI CfrI Bsh1285I Bsp143I PleI NlaIII Bsp143I
gcgccgttcgccggacggccgatctgtttctgcccgactccttcatgcaggagatcatcggggggcaggcccgcg base pairs
cgcggcaagcggcctgccggctagacaaagacgggctgaggaagtacgtcctctagtagccccccgtccgggcgc 33901 to 33975
BanI Msp17I Bsp143II EclXI DpnII DpnI Hsp92II Kzo9I
BshNI Hsp92I BstZI BsiEI MboI NdeII
 Hin1I AcyI BstH2I XmaIII BsaOI Kzo9I Sau3AI

 Alw26I Bse118I
 Bse118I BssAI BstMCI BstD102I AfaI
 BssAI Eco31I Bsh1285I AccBSI Csp6I
agaccggcgacgaccggcccgtcctaacaaagtcggcgggggagcggtttctgctgtaccgcccctccaccacca base pairs
tctggccgctgctggccgggcaggattgtttcagccgccccctcgccaaagacgacatggcggggaggtggtggt 33976 to 34050
 BsrFI BsaI BsiEI DrdI BsrBI RsaI
 Cfr10I BsrFI BsaOI
 BsmAI Cfr10I

 BsoBI AflIII BsaAI
 Eco88I RsaI RsaI Bme18I
 NspBII Ama87I Csp6I Csp6I SinI EaeI BssHII
ccaacagcggcctgatggcccccgagctgtacgtgtacgtggacccggcgttcacggccaacacgcgcgcctccg base pairs
ggttgtcgccggactaccgggggctcgacatgcacatgcacctgggccgcaagtgccggttgtgcgcgcggaggc 34051 to 34125
 MspA1I BcoI AluI MaeII MaeII Eco47I CfrI BsePI
 AvaI AfaI AfaI HgiEI
 BsaAI AvaII

 BssAI BstMCI BshNI RsaI Bst2UI AspHI
BshNI Cfr10I Bsh1285I Asp718I BstNI SduI BsiHKAI
Eco64I SfaNI Eco64I AfaI MboII GsuI Bbv12I BssHII
gcaccggcatcgcggtcgtcgggaggtaccgcgacgatttcattatcttcgccctggagcactttttcctccgcg base pairs
cgtggccgtagcgccagcagccctccatggcgctgctaaagtaatagaagcgggacctcgtgaaaaaggaggcgc 34126 to 34200
BanI Bse118I BsiEI BanI Csp6I EcoRII BpmI Alw21I BsePI
AccB1I BcgI Acc65I KpnI BstOI Bsp1286I
 BsrFI BsaOI AccB1I MvaI BmyI

 Bsp143I KspI SacII VneI BmyI BstOI Esp1396I Bst71I
 MboI DpnI BstDSI Cfr42I BbvI Bsp1286I EcoRII AlwNI HaeII
 DpnII AclWI NspBII NspBII HgaI SduI BsiHKAI MvaI Van91I BbvI
cgctcacgggatcggcccccgcggacatcgcccgctgcgtcgtgcacagcctcgcccaggtgctggcgctgcacc base pairs
gcgagtgccctagccgggggcgcctgtagcgggcgacgcagcacgtgtcggagcgggtccacgaccgcgacgtgg 34201 to 34275
 NdeII AlwI MspA1I MspA1I Alw44I Alw21I BstNI PflMI Bsp143II
 Sau3AI DsaI SstII Bst71I AspHI EcoNI AccB7I BstH2I
 Kzo9I Sfr303I ApaLI Bbv12I Bst2UI BsgI

 Eco88I BstMCI BstOI CfrI Eco52I EaeI BalI
Ama87I BsoBI Bsh1285I Bst71I EaeI EclXI BstMCI
 PspAI PspALI BbvI TthHB8I EcoRII HinfI XmaIII DsaI MluNI
ccggggcgtttcgcagcgttcgcgtggcggtcgagggcaacagcagccaggactcggccgtggccatcgccacac base pairs
ggccccgcaaagcgtcgcaagcgcaccgccagctcccgttgtcgtcggtcctgagccggcaccggtagcggtgtg 34276 to 34350
 Cfr9I SmaI Bst71I BsiEI BbvI MvaI PleI BstDSI CfrI AflIII
 BcoI XmaI TaqI BstNI BstZI Bsh1285I MscI
 AvaI BsaOI Bst2UI EagI BsiEI BsaOI

 BsaAI BstOI Eco88I PspOMI Eco88I FriOI
MslI PmaCI BstF5I PspAI PspALI BcoI SduI BanII
 Eco72I SfaNI EcoRII Bst2UI Ama87I Bsp120I AvaI Eco24I
acgtgcataccgagatgcaccgcatcctggcctcggcgggggccaacggcccggggcccgagctcctcttctatc base pairs
tgcacgtatggctctacgtggcgtaggaccggagccgcccccggttgccgggccccgggctcgaggagaagatag 34351 to 34425
 BbrPI SfaNI MvaI BcoI XmaI EcoO109I BmyI Ecl136II
 MaeII BstNI Cfr9I SmaI DraII BsoBI ApaI
 PmlI FokI AvaI BsoBI Ama87I Bsp1286I

 EcoICRI SacI BsiHKAI TspRI BsaHI BbsI TthHB8I
 AspHI Psp124BI Ksp632I AfaI BbiII Bbv16II Bsp119I
 AluI BmyI FriOI BseRI Csp6I Hin1I BpuAI LspI
actgcgagccgcccggcggcgcggtattgtaccccttctttctgctcaacaaacagaagacgcccgccttcgaat base pairs
tgacgctcggcgggccgccgcgccataacatggggaagaaagacgagttgtttgtcttctgcgggcggaagctta 34426 to 34500
 Eco24I SstI EarI RsaI Msp17I BpiI Csp45I
 Bsp1286I BanII Eam1104I Hsp92I HgaI BstBI
 SduI Bbv12I Alw21I MboII AcyI MboII SfuI

 BsaHI BbiII HgaI BsmFI Bsp1286I BanII BsmAI BstSFI
 BbiII Hin1I AcyI Bst2UI AspHI FriOI Alw26I MaeIII
 NspV Hin1I NlaIII BsaHI MvaI SduI Bbv12I Alw21I Tsp45I
actttatcaaaaagttcaactccgggggcgtcatggcgtcccaggagctcgtctccgtgacggtgcgcctgcaga base pairs
tgaaatagtttttcaagttgaggcccccgcagtaccgcagggtcctcgagcagaggcactgccacgcggacgtct 34501 to 34575
 Msp17I Hsp92II EcoRII AluI BmyI SacI SstI Esp3I
 Bpu14I Hsp92I Msp17I BstNI Ecl136II Eco24I BsiHKAI SfcI
 TaqI AcyI HgaI Hsp92I BstOI EcoICRI Psp124BI BsmBI PstI

 BsiEI MaeII
 AtsI BstMCI Bst71I BsmBI BbiII AatII
 Tth111I BsaOI AluI TthHB8I Alw26I Hin1I AcyI
ccgacccggtcgagtatctgtccgagcagctcaacaacctcatcgaaaccgtctctcccaacaccgacgtccgca base pairs
ggctgggccagctcatagacaggctcgtcgagttgttggagtagctttggcagagagggttgtggctgcaggcgt 34576 to 34650
 AspI TaqI BbvI TaqI BsmAI Msp17I
 TthHB8I Esp3I Hsp92I
 Bsh1285I BsaHI

 Hsp92II BseAI BstDSI Cfr42I MscI Bst2UI
 AfaI BsaWI BshNI MspA1I Hsp92II NlaIII BstNI
 RsaI AccIII Eco64I KspI SacII DrdI EaeI BalI BstXI
tgtactccggaaaacgcaacggtgccgcggacgacctcatggtcgcggtcatcatggccatttacctggcggccc base pairs
acatgaggccttttgcgttgccacggcgcctgctggagtaccagcgccagtagtaccggtaaatggaccgccggg 34651 to 34725
 NlaIII Kpn2I BanI NspBII NlaIII CfrI EcoRII
 Csp6I MroI Bsp13I AccB1I Sfr303I Hsp92II BstOI
 NspI BspEI BsiMI DsaI SstII MluNI MvaI

 BstYI Bsp143I Bsp143I
 BstMCI MboI MflI AclWI MboI DpnI
 BsiEI BstX2I XhoII DpnII MaeII HinfI
cgaccgggatccccccggccttttttccgatcacgcgcacgtcttgagtctttcttgccgtttcttttgtttctc base pairs
gctggccctaggggggccggaaaaaaggctagtgcgcgtgcagaactcagaaagaacggcaaagaaaacaaagag 34726 to 34800
 Bsh1285I Sau3AI DpnI NdeII PleI
 BsaOI NdeII BstI AlwI Sau3AI
 DpnII BamHI Kzo9I Kzo9I


 TspRI
tttctttccccccctctctccgcaataaacgccttcccggaactgtgtttccccccctacaacagtgttgtccgt base pairs
aaagaaagggggggagagaggcgttatttgcggaagggccttgacacaaagggggggatgttgtcacaacaggca 34801 to 34875


 BstMCI
 BsiEI
tggttgggtggttggggtgcgggggtgggcgggggaagcaagaaaacggtcggcgaacacaacatcgggaaaacg base pairs
accaacccaccaaccccacgcccccacccgcccccttcgttcttttgccagccgcttgtgttgtagcccttttgc 34876 to 34950
 Bsh1285I
 BsaOI


 PmaCI BpiI TaqI
 BsaAI Bbv16II Tru1I
 HinfI MaeII HgaI HinfI Tru9I XcmI
gattcccgcacgtgcgtcttcccagattcgacacacacaccccccttctccttaaataaacacaaaccacacgct base pairs
ctaagggcgtgcacgcagaagggtctaagctgtgtgtgtggggggaagaggaatttatttgtgtttggtgtgcga 34951 to 35025
 TfiI Eco72I BpuAI TthHB8I MseI
 PmlI BbsI TfiI
 BbrPI MboII

 HaeII KspI SacII AvaII
 Tru1I AfeI NspBII SinI DraII
 Tru9I Aor51HI MaeII BstDSI Cfr42I EcoO1
cgttggttggttaatgccagcgctttatttacgtcttgttttttttgcgtttcctccgcgggtcccttcccaaca base pairs
gcaaccaaccaattacggtcgcgaaataaatgcagaacaaaaaaaacgcaaaggaggcgcccagggaagggttgt 35026 to 35100
 MseI Eco47III DsaI SstII PpuMI Ec
 Bsp143II MspA1I Bme18I Psp5II
 BstH2I Sfr303I HgiEI BsmFI

 BstDEI AccB1I NarI
 Bse21I KasI BbiII EheI
09I Eco81I NlaIII Eco64I BsaHI
cgcctgcccccgcctcaggggtagcggataaccggggccatgtcgccggattgcacaacggcggcgccgtcgaac base pairs
gcggacgggggcggagtccccatcgcctattggccccggtacagcggcctaacgtgttgccgccgcggcagcttg 35101 to 35175
o47I DdeI Bsu36I Hsp92II BanI Msp17I
 CvnI BshNI Hsp92I
 AocI Hin1I AcyI

HaeII Csp6I MvaI MvaI Eco88I BglI
 TaqI BstOI Bst2UI Ama87I BstOI
 BbeI MaeII EcoRII BstNI BstF5I BsmFI EcoRII
gtacacacccgaaccgccggggccagggccaggatgtccccgagttggcccgcgtgcgccagccaggcgaccagc base pairs
catgtgtgggcttggcggccccggtcccggtcctacaggggctcaaccgggcgcacgcggtcggtccgctggtcg 35176 to 35250
Bsp143II AfaI BstNI BstOI FokI BsoBI BstNI
 TthHB8I Bst2UI BcoI Bst2UI
BstH2I RsaI EcoRII AvaI MvaI


 BstH2I AluI
 Bsp143II BbvI SfaNI NlaIII BstF5I
gcctcgtaaagcggcagcctgcgttcgccgtcctgcatcagcatgggggcttcggggtggatgagctgggcggct base pairs
cggagcatttcgccgtcggacgcaagcggcaggacgtagtcgtacccccgaagccccacctactcgacccgccga 35251 to 35325
 HaeII Bst71I MslI Hsp92II FokI


 MvaI DdeI BlpI
 BstSFI BstOI HaeII Bsp1720I
 Tsp45I HgaI SfcI MaeII EcoRII Bsp143II BbvI
tctcgcgtgacgctctgcatctgcaggagcgcgttcacgtatccgtcctgggcgctcagcgcgagcagccggggg base pairs
agagcgcactgcgagacgtagacgtcctcgcgcaagtgcataggcaggacccgcgagtcgcgctcgtcggccccc 35326 to 35400
 MaeIII SfaNI BsaAI BstNI Bpu1102I Bst71I
 PstI Bst2UI BstH2I
 CelII BstDEI

 AluI
 BstMCI
 BstF5I BstF5I AccI NlaIII BsiEI BbvI
atgagcgtgaggatgagggtggttccttcggttatggagtagaccatgttgaggacgagcgaccgcagctcggtg base pairs
tactcgcactcctactcccaccaaggaagccaatacctcatctggtacaactcctgctcgctggcgtcgagccac 35401 to 35475
 FokI FokI Hsp92II Bsh1285I
 BsaOI Bst71I


 MaeII EaeI BsmBI SduI FriOI
 BbiII AatII Bsp120I Eco24I
 Hin1I AcyI BsiI Alw26I DraII BanII MaeII
tttacggaggcgagttgctggacgtcggccacgagcgagagacgggccccgttgtaatacagcacgttgaggtcg base pairs
aaatgcctccgctcaacgacctgcagccggtgctcgctctctgcccggggcaacattatgtcgtgcaactccagc 35476 to 35550
 Msp17I BssSI BsmAI EcoO109I
 Hsp92I PspOMI BmyI ApaI
 BsaHI CfrI Esp3I Bsp1286I

 Bsp1286I BanII BcoI AvaI Hin1I HgaI DraII BstF5I AvaI
 EcoICRI Eco24I BsiHKAI BsoBI Hsp92I HgiEI BsmFI Ama87I PspALI
 Ecl136II SacI Alw21I Eco88I Msp17I Bme18I Eco47I PspAI BsoBI
gggagctccccgggcgtccgggggtcggggttgaggtcccggatgccccgggcgaccagccgcgcgactatctcg base pairs
ccctcgaggggcccgcaggcccccagccccaactccagggcctacggggcccgctggtcggcgcgctgatagagc 35551 to 35625
 AluI BmyI FriOI Ama87I XmaI BbiII SinI AvaII FokI Cfr9I SmaI
 SduI Bbv12I SstI Cfr9I SmaI BsaHI EcoO109I SfaNI Eco88I
 AspHI Psp124BI PspAI PspALI AcyI PpuMI Psp5II BcoI XmaI

 MvaI HgiEI BsaWI AvaII BstOI
 BstOI SinI AvaII Bme18I PleI GsuI
 EcoRII AccBSI BsmFI BbvI SinI HinfI MvaI MaeII BcgI
cgggccaggggcgttgggagcgggaccggaaaccgcagcgtgaggtccagcgactccaggcgcacgtccgtcgcc base pairs
gcccggtccccgcaaccctcgccctggcctttggcgtcgcactccaggtcgctgaggtccgcgtgcaggcagcgg 35626 to 35700
 BstNI BsrBI Eco47I Bst71I EcoRII BpmI EcoRII
 Bst2UI Bme18I HgiEI BstNI
 BstD102I Eco47I Bst2UI

Bst2UI BbsI MboI Bsp143I HincII
BstNI BpuAI BstX2I XhoII AccI
 TthHB8I BsmFI BstYI BamHI AlwI SalI
tggccctcgaagacgggcgggacgaggctgacgggatccccgttgcagaggtcgacgggggaggtgttgcggaga base pairs
accgggagcttctgcccgccctgctccgactgccctaggggcaacgtctccagctgccccctccacaacgcctct 35701 to 35775
 TaqI MboII DpnII MflI DpnI TthHB8I
BstOI Bbv16II NdeII BstI AclWI TaqI
MvaI BpiI Sau3AI Kzo9I HindII

 MroNI Cfr10I FriOI BstOI Eco47I
 BshNI BsrFI BmyI BstNI Bme18I MslI
 Eco64I NgoAIV SduI EcoRII HgiEI HinfI BbvI
ttgacggtgccggcgtgcgtgagccccaggtccacggggcaggcgacgattcgcgtgggcagcacccgcgtgatt base pairs
aactgccacggccgcacgcactcggggtccaggtgccccgtccgctgctaagcgcacccgtcgtgggcgcactaa 35776 to 35850
 BanI NgoMI Bsp1286I SinI DsaI TfiI Bst71I
 AccB1I Bse118I Eco24I MvaI AvaII
 BssAI NaeI BanII Bst2UI BstDSI

 KspI SacII BsaWI BsmAI
 NspBII HaeII RsaI MroI Bsp13I HinfI
 BstDSI Cfr42I Csp6I MaeII BsmFI BspEI BsiMI Esp3I
accgcggggaagcgcctgcggtacgccagcaacaaccccaacgtgtcgggactaactcctccggagacgaacgat base pairs
tggcgccccttcgcggacgccatgcggtcgttgttggggttgcacagccctgattgaggaggcctctgcttgcta 35851 to 35925
 DsaI SstII Bsp143II AflIII AccIII Alw26I
 MspA1I BstH2I AfaI Kpn2I BseRI TfiI
 Sfr303I BseAI BsmBI

 AluI
 BstH2I SfcI
 MaeII Bsp143II BstF5I TthHB8I NspBII
tcgtgcgccacgtccgcgagcgccagctggcggcggatggtcggcagaaagaccactcgaccctcgcaccgctgc base pairs
agcacgcggtgcaggcgctcgcggtcgaccgccgcctaccagccgtctttctggtgagctgggagcgtggcgacg 35926 to 36000
 HaeII MspA1I FokI TaqI MspA1I
 PvuII BstSFI
 NspBII

BbvI HaeII MspA1I BsoBI Bsp143I BstBI Bpu14I MboI Kzo9I
 PstI BstH2I Sfr303I Eco88I MboI DpnI LspI NspV BclI Bsp143I
 BbvI DsaI KspI SfaNI Ama87I DpnII AclWI TthHB8I FbaI Sau3AI
agcgccgcggcatcggggcgcgagatacccgaggggatcgcgatgtctgcttcgaaacaatccgtgatcatggcg base pairs
tcgcggcgccgtagccccgcgctctatgggctcccctagcgctacagacgaagctttgttaggcactagtaccgc 36001 to 36075
 Bst71I NspBII SacII BcoI NdeII AlwI SfuI TaqI DpnII DpnI
 Bsp143II SstII AvaI Sau3AI NruI Csp45I NdeII Eco64I
Bst71I BstDSI Cfr42I Kzo9I Bsp68I Bsp119I Ksp22I BanI

 NlaIII BbiII Bsp143II
 AccB1I NarI HaeII
 Hsp92II BsaHI Alw26I BsmFI
ccgggccgcgagacaccggaacgcgggggtgcgggagggccggaaagcgcaacgcaaccgggacgatgatgaaac base pairs
ggcccggcgctctgtggccttgcgcccccacgccctcccggcctttcgcgttgcgttggccctgctactactttg 36076 to 36150
 KasI Msp17I EheI BsmAI
 Hin1I AcyI BstH2I
 BshNI Hsp92I BbeI BsaWI

 SduI BsaOI Bsp1720I BanII
 BshNI Bsh1285I CelII BmyI Bst71I
 Eco64I BsiEI Bpu1102I FriOI
agagatggggggcaccgaccgtgtgggagagggggcggggcagggctcagcagcacgcacggggaggtctgtcgt base pairs
tctctaccccccgtggctggcacaccctctcccccgccccgtcccgagtcgtcgtgcgtgcccctccagacagca 36151 to 36225
 BanI BmyI DdeI SduI BbvI
 AccB1I BstMCI BlpI Bsp1286I
 Bsp1286I BstDEI Eco24I

 FriOI BstOI BcoI BsoBI Eco24I BsiHKAI AvaII TfiI
 Acc16I BmyI BstNI HinfI EcoICRI SduI Psp124BI Bme18I Eco47I
 FspI SduI EcoRII TfiI BsmFI Ecl136II BmyI SacI SstI HgiEI BsmFI
gcgcaggagccccaggtgagaatcagtcccccggagctcgggtctgggttttattgggacctgccctcggaatcg base pairs
cgcgtcctcggggtccactcttagtcagggggcctcgagcccagacccaaaataaccctggacgggagccttagc 36226 to 36300
 AviII Bsp1286I HphI AluI AvaI Bbv12I BanII PpuMI Psp5II
 Eco24I MvaI Ama87I Bsp1286I Alw21I EcoO109I HinfI
 BanII Bst2UI Eco88I AspHI FriOI SinI DraII BspMI

 MvaI
 Bse1I BstOI
 BsrSI EcoRII BsmFI MaeII
cggctcccagtccaagcccccctggggggggcggggacagggggtgtgtgtgggtaaaagcaacgtcggaaaatc base pairs
gccgagggtcaggttcggggggaccccccccgcccctgtcccccacacacacccattttcgttgcagccttttag 36301 to 36375
 BseNI BstNI
 BsrI Bst2UI


 Tsp509I
 AluI MboII Sse9I
aaacccaatgccccaaacaggaaaaaaaaagacgggcgggtggagggaaagctggggaagaagaagccaatttta base pairs
tttgggttacggggtttgtcctttttttttctgcccgcccacctccctttcgaccccttcttcttcggttaaaat 36376 to 36450
 TspEI


 BsaHI
 EcoO109I BbiII BsaI
 Alw26I Hin1I Alw26I
cagagacaggccctttagcggggaggcgtcgtagatgagatactgcgtaaagtgggtctctcgcgcgtgggcctc base pairs
gtctctgtccgggaaatcgcccctccgcagcatctactctatgacgcatttcacccagagagcgcgcacccggag 36451 to 36525
 BsmAI Msp17I Eco31I
 DraII Hsp92I BsmAI
 AcyI HgaI

 Bst71I Bsp143I
 BstH2I MboI Kzo9I
 Bsp143II DpnII DpnI EcoNI
cccatcgcgggcgctgcgtagcagggcggggtcgctggcgcaggtgatcgggtaggcttcctgaaacaggccgca base pairs
gggtagcgcccgcgacgcatcgtcccgccccagcgaccgcgtccactagcccatccgaaggactttgtccggcgt 36526 to 36600
 HaeII NdeII HphI
 BbvI Sau3AI
 BspMI

 MboII AluI AspHI FriOI Eco64I Tru9I EaeI
 BbsI Ecl136II Psp124BI BanI HaeII BbvI
 BpuAI BsiI Bsp1286I BanII BshNI MseI MaeII NspBII BstDSI
cgggtcttccacgagctcgcggcaccccggcgggcgcttaaactgcacgtcgctggcagcggtggccgtggatac base pairs
gcccagaaggtgctcgagcgccgtggggccgcccgcgaatttgacgtgcagcgaccgtcgccaccggcacctatg 36601 to 36675
 Bbv16II SduI Bbv12I Alw21I Bsp143II BsgI MspA1I DsaI
 BpiI BssSI BmyI SacI BsiHKAI BstH2I Bst71I
 EcoICRI Eco24I SstI AccB1I Tru1I CfrI

 Bsp143I HgaI GsuI XmaIII BstMCI SstII
 MboI DpnI BsmBI Bst2UI CfrI BsiEI BsaOI Cfr42I
 DpnII AclWI Alw26I BstOI EaeI EaeI Eco52I MspA1I
cgccgatcccgtttccacgatgagacgctccaggcagcgatgtttggccgtgatgtcggccgcggtgaagaactt base pairs
gcggctagggcaaaggtgctactctgcgaggtccgtcgctacaaaccggcactacagccggcgccacttcttgaa 36676 to 36750
 NdeII AlwI BsmAI BstNI BbvI CfrI BstZI BstDSI KspI
 Sau3AI Esp3I MvaI Bst71I EagI Bsh1285I Sfr3
 Kzo9I EcoRII BpmI EclXI DsaI NspBII

 RsaI EcoO109I SplI RsaI
 BstDEI DraII Bsp1407I PpuMI Psp5II SunI
 MboII AlwNI EcoO109I HphI SspBI SinI AvaII PspLI
gaagcaggggctgaggacgggcgaggccccgttgaggtgataggccccgttgtacagcaggtccccgtacgagaa base pairs
cttcgtccccgactcctgcccgctccggggcaactccactatccggggcaacatgtcgtccaggggcatgctctt 36751 to 36825
SacII DdeI DraII EcoO109I Csp6I Bme18I BsmFI BsiWI
03I BsrGI HgiEI Eco47I Csp6I
HphI AfaI DraII BspMI Pfl23II

 Hin1I HgaI Van91I EaeI
 NspBII BbiII BstDSI CfrI BstDSI AfaI
 AfaI BbvI BsaHI AccB7I DsaI EaeI Csp6I NlaIII
ccgctgcgacgcccacgggttggccgtggccgcgaagggccgcgccgggtcgctctggccgtggtcgtacatgag base pairs
ggcgacgctgcgggtgcccaaccggcaccggcgcttcccggcgcggcccagcgagaccggcaccagcatgtactc 36826 to 36900
 MspA1I Hsp92I PflMI BstDSI CfrI RsaI Hsp92II
 Bst71I AcyI Esp1396I DsaI
 Msp17I DsaI EaeI CfrI

 MaeII BsmFI NgoAIV EagI BsiEI BstDSI SstII NgoMI
 BbiII AatII AfaI NgoMI BstZI Eco52I DsaI Sfr303I BsrFI
 Hin1I AcyI BsmFI RsaI MroNI Cfr10I Bsh1285I BsmFI SacII NgoAIV
ggctatgacgtccccctccttgtcccccgcgtacacgccgccggccgcgcgtccccgcgggttgcagggccggcg base pairs
ccgatactgcagggggaggaacagggggcgcatgtgcggcggccggcgcgcaggggcgcccaacgtcccggccgc 36901 to 36975
 Msp17I Csp6I BssAI EaeI EclXI HgaI NspBII MroNI
 Hsp92I BsrFI CfrI NaeI BsaOI MspA1I BssAI
 BsaHI Bse118I XmaIII BstMCI KspI Cfr42I Bse118I

 MluNI DsaI Eco47I BsrFI
 EaeI DsaI BstXI FokI HgiEI BssAI
 BstDSI BstDSI SfaNI SinI NlaIII
aaagtagttgatgtccgtggccacgggggtggcgatgaactcacacacggcatcctgcccgtggtccatgccggc base pairs
tttcatcaactacaggcaccggtgcccccaccgctacttgagtgtgtgccgtaggacgggcaccaggtacggccg 36976 to 37050
Cfr10I DsaI MscI MslI BstF5I Bme18I MroNI
NaeI CfrI BstDSI NgoMI
 BalI AvaII Hsp92II

 AscI BstDSI Cfr42I BstOI Bst71I NgoAIV BsrFI
 Bse118I MspA1I BanI EcoRII NgoMI FseI BstMCI
 NaeI BsePI Sfr303I AccB1I MroNI Cfr10I BsiEI
gcgccgcggcacctgggcgcagccaaagaccgggaggggctgggccggccccagccggtttcccgccacgaccgc base pairs
cgcggcgccgtggacccgcgtcggtttctggccctccccgacccggccggggtcggccaaagggcggtgctggcg 37051 to 37125
 NgoAIV DsaI SstII BshNI MvaI BssAI NaeI Cfr10I Bsh1285I
 Cfr10I NspBII SacII BstNI BbvI BsrFI BssAI BsaOI
 BssHII KspI Eco64I Bst2UI Bse118I Bse118I

 Acc16I EagI Eco52I BfaI PspOMI Eco24I MspA1I Cfr42I BsiEI
 BcgI AfaI CfrI EclXI BsaOI BbvI Bsp120I FriOI NspBII SstII Eco52I
 FspI Csp6I EaeI XmaIII MaeI NspBII DraII BmyI BstDSI EagI EclXI BstMCI
gttgcgcaggtacacggcggccgcgttgtctagcagcgggggggccccgcggccgaggtaaaagttttgggggag base pairs
caacgcgtccatgtgccgccggcgcaacagatcgtcgcccccccggggcgccggctccattttcaaaaccccctc 37126 to 37200
 RsaI BstZI Bsh1285I MspA1I SduI BanII BstZI Sfr303I Bsh1285I
 AviII BspMI CciNI BstMCI Bst71I Bsp1286I DsaI CfrI XmaIII BsaOI
 NotI BsiEI EcoO109I ApaI EaeI KspI SacII

 Bsp1286I MvaI HgiEI
 MaeIII BshNI BstDSI BstOI Eco47I
 NlaIII Eco64I DsaI BglI EcoRII Bme18I
gttgcccatgtccgtaacggggttgcggacggtgcccgtggccgcgacggcggtgtagcccacacccaggtccac base pairs
caacgggtacaggcattgccccaacgcctgccacgggcaccggcgctgccgccacatcgggtgtgggtccaggtg 37201 to 37275
 Hsp92II BanI SduI CfrI BstNI AvaII
 AccB1I EaeI Bst2UI MaeII
 BmyI SinI

 MvaI GsuI
 BstOI
 HphI HincII EcoRII AluI
gtttccgcgcggctgggtgagcgtgaagttgacccccccgcccgtttcgtggcgggccacctggagctggcccag base pairs
caaaggcgcgccgacccactcgcacttcaactgggggggcgggcaaagcaccgcccggtggacctcgaccgggtc 37276 to 37350
 HindII BstNI
 Bst2UI
 BpmI

 Eco47I
 AfaI HgaI HgiEI
 Csp6I BssHII MaeII Tsp45I SinI MslI HphI
aaagtacgcctccgacgcgcgctcggaaaacagcacgttctcggtcacgaagcggtcctgccgcacgacggtgaa base pairs
tttcatgcggaggctgcgcgcgagccttttgtcgtgcaagagccagtgcttcgccaggacggcgtgctgccactt 37351 to 37425
 RsaI BsePI MaeIII Bme18I
 AvaII


 Eco88I FriOI
 PspAI PspALI BmyI AfaI
 Ama87I AluI HphI BstDSI SduI Csp6I
cccgaacccggggtggaggcccgtcttgagctggtgatacagggccacggggctcatcttgaagtaccccgccat base pairs
gggcttgggccccacctccgggcagaactcgaccactatgtcccggtgccccgagtagaacttcatggggcggta 37426 to 37500
 BcoI XmaI DsaI Bsp1286I RsaI
 Cfr9I SmaI Eco24I
 AvaI BsoBI BanII

 BsgI Van91I
 BstDSI
 NlaIII BbvI AccB7I BstF5I
gagcgcgtaggtcagcgcgttctcccccgccgcgctctcgcgggcgtgctgcaccacgggctggcggatggagga base pairs
ctcgcgcatccagtcgcgcaagagggggcggcgcgagagcgcccgcacgacgtggtgcccgaccgcctacctcct 37501 to 37575
 Hsp92II Bst71I PflMI FokI
 DsaI Esp1396I


 MvaI Eco47I MvaI MaeII AatII Bst2UI
 BstOI HgiEI BstNI BbiII BsmFI BstOI
 EcoRII BglI SinI BsmFI Hin1I AcyI BstNI
gaagtagttggcccccagggccggggggaccagggggacgtcgcgcgccaggtcgcgcagggccggggggaagtt base pairs
cttcatcaaccgggggtcccggcccccctggtccccctgcagcgcgcggtccagcgcgtcccggccccccttcaa 37576 to 37650
 BseRI BstNI Bme18I Bst2UI BsaHI EcoRII
 Bst2UI AvaII BstOI Hsp92I BsePI MvaI
 SfiI EcoRII Msp17I BssHII

 BalI PmaCI BshNI Hsp92I
 MscI PmlI KasI BbiII EheI
 EaeI MaeII Eco64I BsaHI BbeI MaeII
gggcgcgttggccacgtggtcggcgcccgcaaacagcgcgtggacgggcaggacgtagaagtattcgccattttg base pairs
cccgcgcaaccggtgcaccagccgcgggcgtttgtcgcgcacctgcccgtcctgcatcttcataagcggtaaaac 37651 to 37725
 CfrI Eco72I BanI Msp17I Bsp143II
 MluNI BbrPI Hin1I NarI HaeII
 BsaAI AccB1I AcyI BstH2I

 AvaII Bst2UI BssAI NaeI Bst71I Acc16I
 Bme18I MvaI Esp1396I BbvI BsrFI BsgI TthHB8I SfaNI
 BstF5I EcoRII AlwNI NlaIII SgrAI Bse118I Bsp143II MboII NlaIII
gatggtgtggtccaggtgctggggggccatgagcagcacgccggcgtgcagcgccccgtcgaagatgcgcatgtt base pairs
ctaccacaccaggtccacgaccccccggtactcgtcgtgcggccgcacgtcgcggggcagcttctacgcgtacaa 37726 to 37800
 FokI SinI BstNI PflMI Hsp92II MroNI Cfr10I HaeII FspI Hsp92II
 HgiEI BstOI Van91I Bst71I NgoAIV BbvI TaqI AviII
 Eco47I AccB7I NgoMI BstH2I NspI

 HincII AccB1I NarI HaeII BshNI BsaHI BstH2I KspI SacII BsiHKAI
EaeI AccI KasI BbiII EheI Eco64I BbiII EheI DsaI Cfr42I BmyI Bst71I
 SalI HgaI Eco64I BsaHI BbeI BanI Msp17I Bsp143II Sfr303I Bbv12I HaeII
ggccgtcgacgcggtgttggcgcccgcgtcgggcgccgcggagcacagcagcgccgtcgtgcgctcggccatgtt base pairs
ccggcagctgcgccacaaccgcgggcgcagcccgcggcgcctcgtgtcgtcgcggcagcacgcgagccggtacaa 37801 to 37875
 TthHB8I BanI Msp17I Bsp143II Hin1I AcyI BstDSI SstII AspHI BbvI
CfrI TaqI BshNI Hsp92I HgaI KasI Hsp92I HaeII NspBII SduI Alw21I BstH2I
 HindII Hin1I AcyI BstH2I AccB1I NarI BbeI MspA1I Bsp1286I Bsp143II

EaeI PstI Bsp1286I TaqI
 CfrI BstSFI Bsp120I BanII HindII NspI
 NlaIII AlwNI BbvI NlaIII SduI ApaI HincII NlaIII
gtgcgccagcacctgcagcgtgagcatggcgggcccgtcgacgacgacgcgcccgttgtggaacatgcgcttgac base pairs
cacgcggtcgtggacgtcgcactcgtaccgcccgggcagctgctgctgcgcgggcaacaccttgtacgcgaactg 37876 to 37950
BcgI Hsp92II SfcI Bst71I Hsp92II Eco24I TthHB8I Hsp92II
 BspMI PspOMI FriOI AccI HgaI
 BmyI SalI

 BalI MspA1I Bsp143I Eco88I
 MscI SfaNI MboI DpnI AvaI
 EaeI BstXI BstF5I Tsp45I DpnII AclWI SduI BseRI
cgtgttggccaccagattggcgggatgcagcgggtgggcggggtcggtcacgggatcgctcgggcactcctcacc base pairs
gcacaaccggtggtctaaccgccctacgtcgcccacccgccccagccagtgccctagcgagcccgtgaggagtgg 37951 to 38025
 CfrI FokI Bst71I MaeIII NdeII AlwI BsoBI
 MluNI NspBII Sau3AI BcoI Bsp1286I
 BbvI Kzo9I Ama87I BmyI

 Bsp143I AvaII BstMCI HgiEI KspI SacII
 MboI DpnI BsmFI SfcI Bst71I AfaI Bsh1285I DsaI SstII BbvI
 DpnII SinI NlaIII PstI Csp6I TthHB8I Eco47I NspBII BsgI
gggggcgatctccgggaccaccatgttctgcagcgtggcgtacacgcggtcgaagcggacccccgcggtgcagca base pairs
cccccgctagaggccctggtggtacaagacgtcgcaccgcatgtgcgccagcttcgcctgggggcgccacgtcgt 38026 to 38100
HphI NdeII Bme18I Hsp92II BbvI RsaI BsiEI SinI BstDSI Cfr42I
 Sau3AI HgiEI BstSFI TaqI Bme18I MspA1I Bst71I
 Kzo9I Eco47I BsaOI AvaII Sfr303I

 NgoAIV BshNI Eco47I EaeI Eco52I
 BstH2I NgoMI Eco64I HgiEI Bse1I EclXI BsrFI
 Bsp143II MroNI Cfr10I BsaAI SinI BsrSI EagI NgoMI
gcgcccccgcgagaaggccggcaccagcacgtaatagtagattttgtggtggacggtccagtcggccggccggtg base pairs
cgcgggggcgctcttccggccgtggtcgtgcattatcatctaaaacaccacctgccaggtcagccggccggccac 38101 to 38175
 HaeII BssAI NaeI MaeII Bme18I BseNI XmaIII
 BsrFI BanI AvaII BsrI CfrI BssAI
 Bse118I AccB1I DrdI BstZI MroNI

Bse118I FseI EagI BsrFI BstMCI BsaHI CfrI Bsh1285I MvaI BssAI Cfr10I Eco52I
 BstMCI BglI EclXI BsiEI BbiII BstZI BsiEI BglI BsgI BsrFI CfrI EclXI BsaOI
 Bsh1285I BstZI Bse118I Hin1I AcyI XmaIII SfiI BstOI MroNI Bse118I BsiEI
cggccggtcgtcggcggcgtcggccgcgcgggcctgggtgttgtgcagcagccggccgtcgttgcggttaaagtc base pairs
gccggccagcagccgccgcagccggcgcgcccggacccacaacacgtcgtcggccggcagcaacgccaatttcag 38176 to 38250
NgoAIV BsaOI CfrI BssAI Bsh1285I EaeI EclXI BsaOI Bst2UI NgoMI BstZI NaeI BstMCI
 BsiEI SfiI XmaIII Cfr10I Msp17I HgaI Eco52I EcoRII BbvI NgoAIV EagI Bsh1285I
Cfr10I NaeI EaeI Eco52I BsaOI Hsp92I EagI BstMCI BstNI Bst71I EaeI XmaIII Tru9I

 Tru1I EclXI MaeII FriOI SduI
 EaeI Eco52I BmyI BssSI
 MseI EagI Bsh1285I AccI SduI BsiI HgaI Alw26I
ggccgtcgccacgttgcacgccgccgcgtagacgggctcgtgcccccccgcgtcaatccggcagtctcggtggcg base pairs
ccggcagcggtgcaacgtgcggcggcgcatctgcccgagcacgggggggcgcagttaggccgtcagagccaccgc 38251 to 38325
 XmaIII BsaOI Bsp1286I BsmAI
 BstZI BstMCI Eco24I BmyI
 CfrI BsiEI BanII Bsp1286I

Eco47I
HgiEI BstOI HaeII
SinI BstNI BsmFI BsiI BbvI Bsp143II SfaNI
gtccagggccgcgtgtcgcataaggccgtcgcagtcccacacgaggggcggcagcagcgccgggtcgcgcatcag base pairs
caggtcccggcgcacagcgtattccggcagcgtcagggtgtgctccccgccgtcgtcgcggcccagcgcgtagtc 38326 to 38400
Bme18I MvaI BssSI Bst71I
AvaII Bst2UI BstH2I
 EcoRII

 BmyI Esp1396I
 TfiI SduI ApaI PflMI EcoRII
 HphI AluI DdeI AluI Bsp120I BanII AccB7I BstNI
gtgattcagctcggcctgagcctgcccgcccagctccgggcccggcagggtaaagtcgtccaccagctgggccag base pairs
cactaagtcgagccggactcggacgggcgggtcgaggcccgggccgtcccatttcagcaggtggtcgacccggtc 38401 to 38475
 HinfI BstDEI PspOMI FriOI PvuII MspA1I
 Bsp1286I AluI NspBII
 Eco24I Van91I BstOI

Bst2UI Bst2UI DraII EaeI Eco88I BsoBI
 TthHB8I BstNI HgiEI BsmFI Ama87I Eco88I
 EcoO109I SexAI SinI AvaII CfrI NlaIII BsoBI Ama87I Tsp45I
ggcctcgacgtgggccaccaggtcccgatacacggccatgcactcctcggggaggtcgcccccgaggtaggtcac base pairs
ccggagctgcacccggtggtccagggctatgtgccggtacgtgaggagcccctccagcgggggctccatccagtg 38476 to 38550
 DraII EcoRII Bme18I Eco47I Hsp92II BseRI BcoI MaeIII
 MvaI MaeII BstOI EcoO109I MslI BcoI EcoNI AvaI
 TaqI MvaI PpuMI Psp5II AvaI

 AlwNI HphI
 AfaI Eco31I Bse1I Van91I DdeI
 Csp6I Alw26I SfaNI BsrSI AluI SfaNI
gatgtacgagaccagcgagtagtcgttcacgaacgccgcgcatcgcgtgttgttccagtagctggtgatgcactg base pairs
ctacatgctctggtcgctcatcagcaagtgcttgcggcgcgtagcgcacaacaaggtcatcgaccactacgtgac 38551 to 38625
 RsaI BsmAI BseNI Esp1396I
 BsaI BsrI PflMI BstDEI
 AccB7I

HinfI MvaI PmlI Bsp1286I
 PleI BstOI Eco72I Bbv12I HinfI BspMI
 MaeIII BsiI EcoRII AflIII BbrPI Alw21I BbvI
agtcacgagccgcgccagggcgcagaacacgtgctcgttgccgtgaatcgcggcttgcagcaggtaaaacaccgc base pairs
tcagtgctcggcgcggtcccgcgtcttgtgcacgagcaacggcacttagcgccgaacgtcgtccattttgtggcg 38626 to 38700
TspRI BssSI BstNI MaeII SduI BsiHKAI Bst71I
 Bst2UI BsaAI AspHI TfiI
 Tsp45I PmaCI BmyI

 HgiEI KspI SacII NgoAIV KasI Msp17I Bsp143II
 Bst71I TaqI NspBII NgoMI Eco64I Hsp92I Eco130I
 AluI SinI TthHB8I BstDSI Cfr42I MroNI Cfr10I BbiII EheI StyI
cgggtagctgcggtcctcgaacgccccgcggacggcggctatggtagccggcgccatggcgtggcggccaacgcc base pairs
gcccatcgacgccaggagcttgcggggcgcctgccgccgataccatcggccgcggtaccgcaccgccggttgcgg 38701 to 38775
 BbvI Eco47I DsaI SstII BssAI NaeI AccB1I AcyI ErhI
 Bme18I MspA1I BsrFI BanI Hin1I NarI BstH2I
 AvaII Sfr303I Bse118I BshNI BsaHI HaeII BssT1I

EcoT14I Esp1396I SduI Bbv12I Alw21I MvaI Ama87I BsoBI Hsp92I EcoRII Bst2UI
NcoI NlaIII EaeI EcoICRI FriOI BsiHKAI BpmI Cfr9I SmaI BsaHI BsaWI BstOI EcoRII
 BstDSI PflMI AluI AspHI SacI SstI Bst2UI BcoI XmaI Msp17I HgaI HaeII MvaI
gagctccaggccccgggcgtcacgaaacgccaccggacacagcgccaggggcaggttgccgttgaccacgcgcca base pairs
ctcgaggtccggggcccgcagtgctttgcggtggcctgtgtcgcggtccccgtccaacggcaactggtgcgcggt 38776 to 38850
BbeI Bsp19I Van91I BmyI Psp124BI BstNI GsuI PspAI PspALI Tsp45I BstH2I HincII
 DsaI AccB7I CfrI Bsp1286I BanII BstOI DraII Eco88I BbiII MaeIII BstNI HindII
 Hsp92II Ecl136II Eco24I EcoRII EcoO109I AvaI Hin1I AcyI Bsp143II BspMI

BstNI BstOI Bsp143I HgiEI BssAI CfrI BsiEI BbiII AatII
 BstNI MboI AlwI CspI Bse118I XmaIII BsmFI TaqI MaeII
XcmI EcoRII NdeII AclWI RsrII Cfr10I Eco52I AluI Hin1I AcyI
ggtggcctggatcgcccccggaccggccggggggacttcgccgccgggaagctcgacgtcggccacgcccgcgaa base pairs
ccaccggacctagcgggggcctggccggcccccctgaagcggcggcccttcgagctgcagccggtgcgggcgctt 38851 to 38925
 Bst2UI DpnII DpnI Bme18I BsrFI EagI BstMCI TthHB8I BsaHI
 MvaI MvaI Sau3AI CpoI AvaII EaeI EclXI BsaOI Msp17I EaeI
 BstOI Bst2UI Kzo9I SinI Eco47I BstZI Bsh1285I Hsp92I CfrI

 BstOI KspI SacII
 TaqI BbvI EcoRII BglI NlaIII NspBII
 MboII AluI GsuI Bst2UI BbvI BstDSI Cfr42I
gaagtcgaacgcggggtgcagctccagagccaggttggcgttgtcgggctgcatgaactgctccgcggtcatctg base pairs
cttcagcttgcgccccacgtcgaggtctcggtccaaccgcaacagcccgacgtacttgacgaggcgccagtagac 38926 to 39000
 TthHB8I BsgI BpmI MvaI Bst71I DsaI SstII
 Bst71I BstNI Hsp92II MspA1I
 XcmI Sfr303I

 AvaII EagI BstDSI Bst71I
 Bme18I XmaIII BstMCI HaeII BstNI Hsp92II
 BsaWI EaeI Eco52I BsaOI BbvI Bst2UI BbvI NlaIII
gcactcggcgacccaccggacccggccgtgggcgaggcgctgccgccaggcgttcagaaaacgctgctgcatgtc base pairs
cgtgagccgctgggtggcctgggccggcacccgctccgcgacggcggtccgcaagtcttttgcgacgacgtacag 39001 to 39075
 SinI BstZI BsiEI Bsp143II MvaI Bst71I
 HgiEI CfrI Bsh1285I BstH2I BstOI NspI
 Eco47I EclXI DsaI EcoRII

 NgoAIV EagI FseI MaeII SunI SplI RsaI
 NgoMI BstZI Eco52I PspLI AfaI SunI AfaI AspI
 MroNI Cfr10I BsiEI SplI Csp6I BsiWI TfiI TthHB8I
cgcgccggggccggccggggccgcgacgtacgccccgtacggattcgcggcctcgacggggtcgtggttcacgcc base pairs
gcgcggccccggccggccccggcgctgcatgcggggcatgcctaagcgccggagctgccccagcaccaagtgcgg 39076 to 39150
 BssAI EaeI EclXI BsaOI Pfl23II Csp6I TaqI Tth111I
 BsrFI CfrI NaeI BstMCI BsiWI PspLI HinfI AtsI
 Bse118I XmaIII Bsh1285I RsaI Pfl23II

 XmaIII DrdI HgaI AspEI AvaII BsaOI NcoI Bsp19I
 CfrI BsiEI TthHB8I Hsp92II EclHKI BsmFI StyI DsaI
 EaeI Eco52I TaqI NlaIII BstF5I SinI Eco47I Eco130I Hsp92II
cccgacggccgcgtcgatgttcatgagcgaaggatgacacacggtcccgaccgcgttctccatggacagccgcag base pairs
gggctgccggcgcagctacaagtactcgcttcctactgtgtgccagggctggcgcaagaggtacctgtcggcgtc 39151 to 39225
 BstZI BstMCI BspHI FokI AhdI HgiEI BstMCI ErhI BstDSI
 EagI Bsh1285I RcaI Eam1105I BsiEI BssT1I NlaIII
 EclXI BsaOI Bme18I Bsh1285I EcoT14I

 BstOI AvaII BbvI FriOI
 AlwNI Bme18I NspBII BmyI CfrI BshNI
 SexAI MvaI PvuII SduI EaeI Eco64I
aacctggtggtcctttccccaaaaaaacagctgccggggagggaacgcgcggggctccgggtggccgggggcggg base pairs
ttggaccaccaggaaaggggtttttttgtcgacggcccctcccttgcgcgccccgaggcccaccggcccccgccc 39226 to 39300
 EcoRII SinI AluI Bsp1286I BanI
 BstNI HgiEI MspA1I Eco24I AccB1I
 Bst2UI Eco47I Bst71I BanII

 SduI BstOI HgiEI BsmFI HaeII StyI Bsp19I Bst2UI
 SexAI MvaI DraII AfeI Eco130I DsaI NlaIII BstOI
 EcoRII PpuMI Psp5II Aor51HI ErhI BstDSI EcoRII PshAI
caccaggtccccggcgtgcgcggcgaagcgctccatggccgggttgaacagccccaggggcaggacgaacgtcag base pairs
gtggtccaggggccgcacgcgccgcttcgcgaggtaccggcccaacttgtcggggtccccgtcctgcttgcagtc 39301 to 39375
 BmyI Bst2UI EcoO109I Eco47III BssT1I CfrI BstNI MaeII
 BstNI Bme18I Eco47I Bsp143II EcoT14I Hsp92II BglI
 Bsp1286I SinI AvaII BstH2I NcoI EaeI MvaI

Eco47I EcoT14I KasI Msp17I EheI BstNI BmyI BsmBI Bst2UI BstOI
HgiEI StyI DsaI NlaIII BbiII Bsp143II SduI Alw26I EcoO109I MvaI
SinI ErhI BstDSI Hsp92II BsaHI BbeI BstOI MaeII SfaNI EcoRII GsuI BstNI BpmI
gtccatggcgcccaccagggggtagggcacgttggtggcggcgtagatgcgtctctccagggcctccaggaagac base pairs
caggtaccgcgggtggtcccccatcccgtgcaaccaccgccgcatctacgcagagaggtcccggaggtccttctg 39376 to 39450
Bme18I NcoI Eco64I Hin1I NarI HaeII Bst2UI HgaI BstNI BpmI Bst2UI
AvaII BssT1I BanI BshNI Hsp92I EcoRII Bsp1286I BsmAI BstOI EcoRII MboII
 Eco130I Bsp19I AccB1I AcyI BstH2I MvaI MslI Esp3I MvaI DraII GsuI

BpuAI BalI MboI Bsp143I TaqI
 BbsI MscI BstX2I DpnI BsePI BstH2I
 DrdI EaeI BstYI MflI AlwI Bsp143II BstF5I
cagcctgtcgcctatggccaccagatccgcgcgcacgcgcgttgtctggggggcgctttcgagttcatccagcgt base pairs
gtcggacagcggataccggtggtctaggcgcgcgtgcgcgcaacagaccccccgcgaaagctcaagtaggtcgca 39451 to 39525
 Bbv16II CfrI DpnII XhoII BssHII HaeII FokI
 MluNI NdeII Kzo9I TthHB8I
 BpiI Sau3AI AclWI

 Esp3I Eco88I BspMI MvaI
 BsmAI XhoI PaeR7I Esp1396I BstOI
 Alw26I Sfr274I BseRI AccB7I MaeII EcoRII
ctcccggttcgcctcgagttgctcctcctgcatatccagcaggtggcggcccacgtcgtccaggctccgcacggc base pairs
gagggccaagcggagctcaacgaggaggacgtataggtcgtccaccgccgggtgcagcaggtccgaggcgtgccg 39526 to 39600
 HgaI Ama87I TaqI PflMI BstNI
 BsmBI BcoI BsoBI Van91I Bst2UI
 AvaI TthHB8I

 MaeIII PspEI BssAI MaeII
 BstH2I BstPI MaeIII
 HphI Bsp143II Eco91I BsrFI
cttgcccatcaccagcgccgtgacgaggttggccccgttcaagaccatctcgccgtaggtcaccggcacgtcggc base pairs
gaacgggtagtggtcgcggcactgctccaaccggggcaagttctggtagagcggcatccagtggccgtgcagccg 39601 to 39675
 HaeII BstEII Bse118I
 Tsp45I EcoO65I HphI
 Tsp45I Cfr10I

 PspEI HphI
 PstI BstH2I BstPI MaeIII AccI
 Eco57I SfcI Bsp143II Eco91I SalI
ctcggtgtcctccaccttcaggaaggactgcaggaggcgctgtttgatggcggcggtggtgaccagcaccccgtc base pairs
gagccacaggaggtggaagtccttcctgacgtcctccgcgacaaactaccgccgccaccactggtcgtggggcag 39676 to 39750
 BstSFI HaeII BstEII TthHB8I
 EcoO65I TaqI
 Tsp45I

HincII BsiEI BstZI CfrI Eco47I
 BsrFI BsaOI XmaIII BmyI Bst71I HgiEI
 BssAI BstMCI EagI HgaI SduI DsaI DrdI HgaI SinI
gaccggccgcccgcgcgtgtcggcgtgcgtcaggcggggcacggccacggacggctgcgtcgccgtggtcaggtc base pairs
ctggccggcgggcgcgcacagccgcacgcagtccgccccgtgccggtgcctgccgacgcagcggcaccagtccag 39751 to 39825
 Cfr10I CfrI Bsp1286I BbvI BstDSI Bme18I
 Bse118I EaeI Eco52I EaeI DsaI AvaII
HindII Bsh1285I EclXI BstDSI

 BstOI Eco147I Bst2UI
 EcoRII StuI TaqI BstNI
 BsiI MvaI TthHB8I EcoNI MboII BsiI AluI
cacgagccaggcctcgatggcctcgcggcgatggcccgccttgcccaggaagaagctcgtgtcgcaaaagctccg base pairs
gtgctcggtccggagctaccggagcgccgctaccgggcggaacgggtccttcttcgagcacagcgttttcgaggc 39826 to 39900
 BssSI Pme55I SfiI EcoRII AluI BssSI
 BstNI AatI BglI BstOI
 Bst2UI SseBI MvaI

 MvaI Eco88I EcoRII MvaI KasI Hsp92I HaeII EcoRV
 BstOI PspAI PspALI BstOI AccB1I NarI BbeI Eco32I
 Eco57I EcoRII Ama87I SmaI MvaI EcoRII BanI BbiII EheI TaqI NlaIII
cttcagctcggcgaccagggtcgcccgggcaaccctggtcgccaggcgcccgttgtcgagatatcgttgcatggg base pairs
gaagtcgagccgctggtcccagcgggcccgttgggaccagcggtccgcgggcaacagctctatagcaacgtaccc 39901 to 39975
 AluI BstNI BcoI XmaI BstNI BstNI BshNI BsaHI BstH2I Hsp92II
 Bst2UI Cfr9I SrfI BstOI Bst2UI Hin1I AcyI TthHB8I
 PshAI AvaI BsoBI Bst2UI Eco64I Msp17I Bsp143II

 MvaI AccB1I NarI HaeII PmlI SfaNI EclXI BsaOI
 BstOI KasI BbiII EheI Eco72I BbvI CfrI BsiEI
 EcoRII Eco64I BsaHI BbeI MaeII BsgI EaeI Eco52I
caacagcagggccaggggaggcgccttctccaacagcacgtgcagcatctggtcggccgtgccgcgctcaaacgc base pairs
gttgtcgtcccggtcccctccgcggaagaggttgtcgtgcacgtcgtagaccagccggcacggcgcgagtttgcg 39976 to 40050
 BstNI BanI Msp17I Bsp143II BbrPI AlwNI BstZI BstMCI
 Bst2UI BshNI Hsp92I BsaAI Bst71I EagI Bsh1285I
 Hin1I AcyI BstH2I PmaCI XmaIII

 MvaI MvaI BbvI
 BstOI BstOI Bst71I BssHII NspBII
 EcoRII EcoRII BssHII AluI BstF5I PvuII SfaNI
ccccaggacggcctggacgttgcgcgcgagctgctggatggcgcgcagctggcgatgcaggctaatgcccgtccc base pairs
ggggtcctgccggacctgcaacgcgcgctcgacgacctaccgcgcgtcgaccgctacgtccgattacgggcaggg 40051 to 40125
 BstNI BstNI BsePI BbvI FokI AluI
 Bst2UI Bst2UI BsePI MspA1I
 MaeII Bst71I

 Bst2UI CfrI BstOI SduI ApaI
 BstNI BglI EcoRII EcoO109I BanII
BsmFI EcoO109I BsrDI BcgI MluNI MvaI Bsp120I FriOI
gtccagggcctcccccgtgagcagggcaatggcctcggtggccaggctgaaggcggcgttcagggcccggcggtc base pairs
caggtcccggagggggcactcgtcccgttaccggagccaccggtccgacttccgccgcaagtcccgggccgccag 40126 to 40200
 EcoRII DraII SfiI BalI Eco57I PspOMI Eco24I
 BstOI EaeI BstNI DraII BmyI TthHB8I
 MvaI MscI Bst2UI Bsp1286I

 BstMCI Tsp509I
 BsaOI Sse9I TthHB8I
 Bsh1285I NlaIII BbvI
gatgaccttcgtcatgtaattatgcacgggctgctcgacggggtgcgggccgtcgcgggcgatgaggggctggtg base pairs
ctactggaagcagtacattaatacgtgcccgacgagctgccccacgcccggcagcgcccgctactccccgaccac 40201 to 40275
 TaqI PshAI Hsp92II Bst71I
 TspEI TaqI
 BsiEI

Eco47I Bsp1286I
HgiEI ApaLI Bbv12I DsaI
SinI TthHB8I BsgI NlaIII AluI Alw44I Alw21I
gacctcgaactgcacacgcccttcgttcatgtaagccagctccgggaacttggtgcacacgcacgccacggacag base pairs
ctggagcttgacgtgtgcgggaagcaagtacattcggtcgaggcccttgaaccacgtgtgcgtgcggtgcctgtc 40276 to 40350
Bme18I Hsp92II VneI BmyI BstDSI
AvaII SduI BsiHKAI
 TaqI AspHI

 Bsp1286I BanII BpmI EcoO109I BsaHI
 EcoICRI Eco24I BsiHKAI PpuMI Psp5II BbiII
 Ecl136II SacI Alw21I SinI AvaII Hin1I MaeII
gccgagctccagaaagcgcacgagcgacagggtgttgcagtaggaccccagcagggcgtcaaactctacgtcata base pairs
cggctcgaggtctttcgcgtgctcgctgtcccacaacgtcatcctggggtcgtcccgcagtttgagatgcagtat 40351 to 40425
 AluI BmyI FriOI GsuI Bme18I Msp17I
 SduI Bbv12I SstI BssSI HgiEI Eco47I Hsp92I
 AspHI Psp124BI BsiI DraII AcyI HgaI

 BsaHI NdeII DpnI
 BbiII TaqI Bsp143I
 HinfI Hin1I HgaI MboI BspCI
caggctgttttcgtcggagcgcacggcggcgaaaaaatcaaagagtctgcggtgggacgccacctcgatcgtact base pairs
gtccgacaaaagcagcctcgcgtgccgccgcttttttagtttctcagacgccaccctgcggtggagctagcatga 40426 to 40500
 PleI Msp17I TthHB8I BsiEI
 Hsp92I DpnII Kzo9I
 AcyI BsmFI Sau3AI

 Bsh1285I FokI Cfr10I BstDSI Cfr42I BsaWI BstPI Ama87I PspALI BstD102I
 PvuI AfaI BsrFI BstF5I Sfr303I AgeI Cfr10I MaeIII XmaI CfrI
 BsaOI DdeI Bse118I EaeI NspBII Csp6I BsrFI EcoO65I Cfr9I SmaI AccBSI
caggatggagccggtgggcaggatggccgcggcgtaccggtaacccggggggtcgcgggcaggagcggccattgg base pairs
gtcctacctcggccacccgtcctaccggcgccgcatggccattgggccccccagcgcccgtcctcgccggtaacc 40501 to 40575
 Csp6I BstDEI CfrI MspA1I RsaI PinAI Eco91I BcoI AvaI EaeI
 BstMCI BstF5I FokI KspI SacII BssAI BstEII PspAI BsoBI BsrBI
Ple19I RsaI BssAI DsaI SstII AfaI Bse118I PspEI Eco88I

 EcoT14I BcoI BsoBI Eco24I BsiHKAI
 StyI EcoICRI SduI Psp124BI
 Eco130I HinfI Ecl136II BmyI SacI SstI HphI
gttccttgggggattcgcaggctccatcaagccgagctcgggaaggccaagcccctcccgcacaacgcctcaccg base pairs
caaggaaccccctaagcgtccgaggtagttcggctcgagcccttccggttcggggagggcgtgttgcggagtggc 40576 to 40650
 ErhI TfiI AluI AvaI Bbv12I BanII
 BssT1I Ama87I Bsp1286I Alw21I
 Eco88I AspHI FriOI

NgoMI EcoT14I
MroNI Cfr10I StyI
 NgoAIV HgaI BstDSI Eco130I
ccggcggacgcgactaacaacccacgggccgccaaaaccccaaggggcaacccgaccaacaacaggcgaggggag base pairs
ggccgcctgcgctgattgttgggtgcccggcggttttggggttccccgttgggctggttgttgtccgctcccctc 40651 to 40725
SgrAI Bse118I DsaI ErhI
BssAI NaeI BssT1I
BsrFI

 BsoBI AvaII EaeI XmaIII
 Eco88I BssAI BstZI
 MaeII Ama87I Eco47I CfrI
gaaaggcgtaaagggggcgttgggaggcaaaaagaaagaaaacacccagacgtaggcccgaggaccggccggcgt base pairs
ctttccgcatttcccccgcaaccctccgtttttctttcttttgtgggtctgcatccgggctcctggccggccgca 40726 to 40800
 BcoI SinI BsrFI EagI
 AvaI Bme18I Cfr10I
 HgiEI Bse118I

 BsiEI Msp17I BcoI SduI BsiHKAI
 Eco52I NaeI BbiII Ama87I AspHI SduI Bst71I
 EclXI Bsh1285I AcyI BsoBI BmyI TspRI AluI
cctctgtccccgagcacccactgtgcccaacaggcacgggggcgagctgcccctgccttatatacccccccgcca base pairs
ggagacaggggctcgtgggtgacacgggttgtccgtgcccccgctcgacggggacggaatatatgggggggcggt 40801 to 40875
 NgoAIV Hin1I HgaI BsmFI Bbv12I Bsp1286I BbvI
 MroNI BstMCI BsaHI Eco88I Alw21I BmyI
 NgoMI BsaOI Hsp92I AvaI Bsp1286I BglI

 MvaI HgiEI
 BshNI BstOI Eco47I
 Eco64I EcoRII Bme18I MaeI
cacccccgttagaacgcgacgggtgccttcaagatggccctggtccaaaagcgtgctagaaaaaagttggtaaag base pairs
gtgggggcaatcttgcgctgcccacggaagttctaccgggaccaggttttcgcacgatcttttttcaaccatttc 40876 to 40950
 BanI BstNI AvaII BfaI
 AccB1I Bst2UI
 SinI

 KasI BbiII EheI Bse118I EaeI NaeI BstMCI
 Hsp92II Hin1I AcyI NgoAIV BbeI XmaIII BglI
 MslI NlaIII AccB1I NarI BsrFI BstH2I EclXI BsaOI
gcggcaaagcagtccgccgccgccacccacatggcggcgccggccgcgcaggcgattcccagagaacgggcgcgg base pairs
cgccgtttcgtcaggcggcggcggtgggtgtaccgccgcggccggcgcgtccgctaagggtctcttgcccgcgcc 40951 to 41025
 BstXI Eco64I BsaHI BssAI Bsp143II EagI Bsh1285I
 BanI Msp17I MroNI Cfr10I BstZI BsiEI HinfI
 BshNI Hsp92I NgoMI HaeII CfrI Eco52I TfiI

 MboI Bsp143I AluI Bsp143I AspEI Esp3I
 BstX2I XhoII AlwNI MboI DpnI EclHKI BsmBI
 BstYI BamHI AlwI BbvI MspA1I DpnII HphI BsmFI Alw26I
aggggatccgtgcggggcagcagctggctggcggtgatccaatggaaaagcccgtcgggactgaacgtctcatgg base pairs
tcccctaggcacgccccgtcgtcgaccgaccgccactaggttaccttttcgggcagccctgacttgcagagtacc 41026 to 41100
 DpnII MflI DpnI Bst71I NdeII AclWI AhdI BsmAI
 NdeII BstI AclWI PvuII Sau3AI Eam1105I NlaIII
 Sau3AI Kzo9I NspBII Kzo9I AlwI MaeII Hsp92II

 CfrI XmaIII EcoRII MboI Kzo9I
 BstZI Eco52I BstOI BclI Bsp143I
 CciNI BsiEI BstNI FbaI Sau3AI XcmI
gcggccgccaccagggcgcacagggccgcgccgcccatgatcacgcacaacccccaaaacacgggtggcgacaac base pairs
cgccggcggtggtcccgcgtgtcccggcgcggcgggtactagtgcgtgttgggggttttgtgcccaccgctgttg 41101 to 41175
 EaeI NotI BstMCI Bst2UI DpnII NlaIII
 EagI Bsh1285I BglI NdeII Hsp92II
 EclXI BsaOI MvaI Ksp22I DpnI

 Bsp143I AfaI PmaCI
 MboI DpnI Csp6I HaeII BsaAI
 DpnII AclWI MaeII BseRI MaeII MaeIII
ggcaggcgatcccgtttgatgttcacgtacaggaggagcgcccgtgccagccacgtgacatagtaggcgaggacg base pairs
ccgtccgctagggcaaactacaagtgcatgtcctcctcgcgggcacggtcggtgcactgtatcatccgctcctgc 41176 to 41250
 NdeII AlwI BsaAI Bsp143II Eco72I
 Sau3AI RsaI BstH2I PmlI BbrPI
 Kzo9I Tsp45I

 Hsp92II Eco64I Msp17I Bsp143II AvaII DsaI CfrI
 BssAI NspI BanI BbiII EheI SinI NspI MspA1I SacII NspBII
 NlaIII NaeI KasI Hsp92I HaeII Bme18I NlaIII EaeI Cfr42I GsuI
gcggctataatacatgccggcgccaccgcccgtccggtccacccgtaatacatgcccgcggccaccagctccagc base pairs
cgccgatattatgtacggccgcggtggcgggcaggccaggtgggcattatgtacgggcgccggtggtcgaggtcg 41251 to 41325
 MroNI NgoAIV BshNI BsaHI BstH2I Hsp92II KspI AluI BpmI
 NgoMI Bse118I Hin1I NarI BbeI HgiEI BstDSI Sfr303I MspA1I
 BsrFI Cfr10I AccB1I AcyI BsaWI Eco47I NspBII SstII

 Eco47I
 HgiEI BstOI Bse118I
 SinI BstNI HphI BstXI BssAI
ggcttgaggaccaggaacgaccaagcaaacatcaccacccgcttggaaaagaccggctgggtgtggggcggaaga base pairs
ccgaactcctggtccttgctggttcgtttgtagtggtgggcgaaccttttctggccgacccacaccccgccttct 41326 to 41400
 Bme18I MvaI BsrFI
 AvaII Bst2UI Cfr10I
 EcoRII

 BpiI SunI AfaI
 Bbv16II Pfl23II BsrBI
MboII MaeII SplI RsaI TthHB8I
cgcgagtaggccgaactgacaaaaaaatcagacgtgccgtacgaggacagcgaaaactgttcatcgagcggcagt base pairs
gcgctcatccggcttgactgtttttttagtctgcacggcatgctcctgtcgcttttgacaagtagctcgccgtca 41401 to 41475
 BpuAI PspLI TaqI AccBSI
 BbsI BsiWI BstD1
 HgaI Csp6I

 MboI Kzo9I
 NruI Bsp143I
 AccI AluI DpnII DpnI BstF5I MslI
tctccgtcctccccgccacacgcggcctcgtctaccagctcgcgatccaacaaaggaacatcatcccgcattgtc base pairs
agaggcaggaggggcggtgtgcgccggagcagatggtcgagcgctaggttgtttccttgtagtagggcgtaacag 41476 to 41550
 Bsp68I AclWI FokI
 02I NdeII AlwI
 Sau3AI

 NgoAIV PspAI BsoBI ApaI
 NgoMI BglI Ama87I Bsp1286I
 NlaIII MroNI Cfr10I BbvI BcgI Bsp120I AvaI BmyI
atggtcggtgcggggagccggcgaggcagcaaaaccgaaagtagtgctggcggcgcgggcccgggtccggaccca base pairs
taccagccacgcccctcggccgctccgtcgttttggctttcatcacgaccgccgcgcccgggcccaggcctgggt 41551 to 41625
 Hsp92II BssAI NaeI Bst71I PspOMI Eco88I FriOI
 BsrFI BcoI XmaI Eco24I
 Bse118I Cfr9I SduI BanII

 HgiEI MroI BsiMI HindIII Eco88I
 SmaI Eco47I Bsp13I AluI PspAI
PspALI BspEI BseAI AvaII FokI Ama87I
agcttcagggatggggggcggaggccaaaatcaaacaagcaccgcgcgggttctacacacaacccccacccgggt base pairs
tcgaagtccctaccccccgcctccggttttagtttgttcgtggcgcgcccaagatgtgtgttgggggtgggccca 41626 to 41700
 SinI AccIII SinI Eco47I BcoI XmaI
 Bme18I BsaWI HgiEI Eco57I Cfr9I
 AvaII Kpn2I Bme18I BstF5I AvaI BsoBI

 KspI SacII Bst2UI XmaIII Bsp120I FriOI
PspALI NspBII BstF5I BstNI MaeII CfrI BsiEI PspOMI
 BstDSI Cfr42I EcoRII Tsp45I BsmFI EaeI Eco52I SduI BanII
agtatccgcggatgcgagtgcctggcgaagtcacgtcccagcaggatataaacctcggccgttgggcccggaacc base pairs
tcataggcgcctacgctcacggaccgcttcagtgcagggtcgtcctatatttggagccggcaacccgggccttgg 41701 to 41775
 DsaI SstII SfaNI MaeIII BstZI BstMCI BmyI ApaI
SmaI MspA1I FokI BstOI EagI Bsh1285I Eco24I
 Sfr303I MvaI EclXI BsaOI Bsp1286I

 Tsp509I BsaHI XcmI
 TspEI BbiII Esp1396I FokI FokI
 Sse9I Hin1I AccB7I BstF5I Bsp68I MslI HgaI
cccgaaattcacacccacgccctgacgcccaaatcatgggtggatgtggttcgcgagccgcacatccgtgcgtcc base pairs
gggctttaagtgtgggtgcgggactgcgggtttagtacccacctacaccaagcgctcggcgtgtaggcacgcagg 41776 to 41850
 AcsI Msp17I PflMI Hsp92II NruI BstF5I
 ApoI Hsp92I Van91I
 AcyI HgaI NlaIII

 KspI SacII
 NspBII
 BstDSI Cfr42I TspRI
gccctcccccgcgggctgatgacgtggcggttagtcagtgggaaggcagggggaaagatgggttgggggaggaaa base pairs
cgggagggggcgcccgactactgcaccgccaatcagtcacccttccgtccccctttctacccaaccccctccttt 41851 to 41925
 DsaI SstII MaeII
 MspA1I
 Sfr303I

 NgoAIV EagI FseI Tsp45I
 BsaMI Tru1I NgoMI BstZI Eco52I
 MboII MaeII BsmI Tru9I MroNI Cfr10I BsiEI
cgaagaaaacacccagagggccacgtcgggaatgcgcccggagttgtccttaaaaggccggccgtgcgtgacgga base pairs
gcttcttttgtgggtctcccggtgcagcccttacgcgggcctcaacaggaattttccggccggcacgcactgcct 41926 to 42000
 Mva1269I MseI BssAI EaeI EclXI BsaOI
 BsrFI CfrI NaeI BstMCI
 Bse118I XmaIII Bsh1285I

 BsaHI MboI DpnI
 BbiII NdeII AlwI
 MaeIII Hin1I DpnII AclWI EaeI
agccgtcgtttgcccaagcaccgacgccgcgatccacagtggggggagttcctccgtccggccacaaccctacgc base pairs
tcggcagcaaacgggttcgtggctgcggcgctaggtgtcacccccctcaaggaggcaggccggtgttgggatgcg 42001 to 42075
 Msp17I Sau3AI TspRI CfrI
 Hsp92I Bsp143I
 AcyI HgaI Kzo9I

 HgiEI Eco47I
 Bme18I BsmFI
 BstDSI DraII
gcgggcggcacgcgcgagagcaacccacgggtcccgttcgcgccaccgccagcccttgctcccaccaccctcctc base pairs
cgcccgccgtgcgcgctctcgttgggtgcccagggcaagcgcggtggcggtcgggaacgagggtggtgggaggag 42076 to 42150
 DsaI SinI AvaII
 PpuMI Psp5II
 EcoO109I

 NgoAIV Eco130I DsaI Ecl136II SduI
 NgoMI CfrI BssT1I Hsp92II BlpI
BseRI BstDSI MroNI Cfr10I EcoT14I EcoICRI BstDEI
ccaccaccccactattcccccccccaagtccgccccgtggctcgccggccatggagctcagctatgccaccaccc base pairs
ggtggtggggtgataagggggggggttcaggcggggcaccgagcggccggtacctcgagtcgatacggtggtggg 42151 to 42225
 DsaI BssAI EaeI StyI Bsp19I Bpu1102I
 BsrFI NaeI NcoI NlaIII DdeI Bsp172
 Bse118I ErhI BstDSI AluI CelII

 Bbv12I Alw21I Eco47I
Bsp1286I BanII MaeII MaeIII HgiEI
 Eco24I BsiHKAI MaeII SinI
tgcaccaccgggacgttgtgttttacgtcacggcagacagaaaccgcgcctactttgtgtgcggggggtccgttt base pairs
acgtggtggccctgcaacacaaaatgcagtgccgtctgtctttggcgcggatgaaacacacgccccccaggcaaa 42226 to 42300
 BmyI Psp124BI BsgI Tsp45I Bme18I
 0I SacI SstI BsmFI AvaII
AspHI FriOI AluI

 BsoBI Bst2UI
 Eco88I BstDEI Tsp509I BstNI AvaII DraII
 Ama87I DdeI Sse9I XcmI SinI BsmFI
attccgtagggcggcctcgggattctcagccgggggaaattgccaagtttggcctggtggtccgggggacaggcc base pairs
taaggcatcccgccggagccctaagagtcggccccctttaacggttcaaaccggaccaccaggccccctgtccgg 42301 to 42375
 BcoI HinfI TspEI EcoRII Bme18I EcoO109I
 AvaI TfiI BstOI Eco47I
 MvaI HgiEI

 PspLI SunI EcoICRI Eco24I BsiHKAI
 Hsp92II SplI SnaBI AfaI AspHI SacI SstI
 XcmI NlaIII MaeII BstSNI Ecl136II Psp124BI MaeII BstDSI
ccaaagaccgcatggtcgccaactacgtacgaagcgagctccgccagcgcggcctgcgggacgtgcggcccgtgg base pairs
ggtttctggcgtaccagcggttgatgcatgcttcgctcgaggcggtcgcgccggacgccctgcacgccgggcacc 42376 to 42450
 DrdI Eco105I Csp6I SduI Bbv12I Alw21I BsmFI DsaI
 BsaAI BsiWI AluI BmyI FriOI BglI
 Pfl23II RsaI Bsp1286I BanII

 MvaI Bsp1286I BanII Alw26I
 BstOI EcoICRI Eco24I BsiHKAI
 EcoRII MaeII Ecl136II SacI Alw21I Esp3I
gggaggacgaggtgttcctggacagcgtgtgtctgctaaacccgaacgtgagctccgagcgagacgtgattaata base pairs
ccctcctgctccacaaggacctgtcgcacacagacgatttgggcttgcactcgaggctcgctctgcactaattat 42451 to 42525
 BstNI AluI BmyI FriOI MaeII AsnI
 Bst2UI SduI Bbv12I SstI BsmBI
 AspHI Psp124BI BsmAI

 PshBI BstNI CfrI Eco88I EcoO65I
VspI MaeII Mva1269I PspAI PspALI
 MseI EcoRII MvaI BsgI BbvI Ama87I Eco91I
ccaacgacgttgaagtgctggacgaatgcctggccgaatactgcacctcgctgcgaaccagcccgggggtgctgg base pairs
ggttgctgcaacttcacgacctgcttacggaccggcttatgacgtggagcgacgcttggtcgggcccccacgacc 42526 to 42600
 Tru1I BsmI Bst2UI Bst71I BcoI XmaI BstEII
 AseI BsaMI EaeI Cfr9I SmaI BstPI
Tru9I BstOI AvaI BsoBI PspEI

 AtsI Bbv12I MboI BspCI BsaOI
Tsp45I Tth111I AluI AspHI DpnII Kzo9I BstMCI
 BssHII Alw26I TthHB8I SduI BsiHKAI BsiEI HincII
tgaccggggtgcgcgtgcgcgcgcgagacagggtcatcgagctatttgagcacccggcgatcgtcaacatttcct base pairs
actggccccacgcgcacgcgcgcgctctgtcccagtagctcgataaactcgtgggccgctagcagttgtaaagga 42601 to 42675
 BsePI BsmAI TaqI Bsp1286I Bsp143I Bsh1285I
 MaeIII AspI BmyI NdeII DpnI PvuI
 HphI Alw21I Sau3AI Ple19I HindII

 SnaBI MvaI Bst2UI BsoBI AspHI
 AfaI BsaAI BstOI BstOI Eco88I Bsp1286I
 Csp6I MaeII EcoRII EcoRII Ama87I SduI
cgcgcttcgcgtacaccccctccccctacgtattcgccctggcccaggcgcacctcccccggctcccgagctcgc base pairs
gcgcgaagcgcatgtgggggagggggatgcataagcgggaccgggtccgcgtggagggggccgagggctcgagcg 42676 to 42750
 RsaI Eco105I BstNI BstNI BcoI Ecl136II
 BstSNI Bst2UI MvaI AvaI AluI
 EcoNI EcoICRI

 Eco24I BsiHKAI EcoRII AccBSI Esp1396I Msp17I BssAI
 BanII BpmI FriOI Bst2UI BsaMI AccB7I MvaI BsaHI Bse118I
 Bbv12I Alw21I BmyI BstNI BsrBI BsmI EcoRII BstNI BbiII BsrFI
tggagcccctggtgagcggcctgtttgacggcattcccgccccgcgccagcccctggacgcccgcgaccggcgca base pairs
acctcggggaccactcgccggacaaactgccgtaagggcggggcgcggtcggggacctgcgggcgctggccgcgt 42751 to 42825
 SacI SstI Bsp1286I BstOI Mva1269I AlwNI BstOI Hsp92I Bsh1285I
BmyI FriOI GsuI Eco24I MvaI BstD102I PflMI Bst2UI AcyI Cfr1
 Psp124BI SduI BanII HphI Van91I Hin1I HgaI BsiEI

BstMCI DpnII Kzo9I SduI Eco47I
 FokI NdeII DpnI BshNI CfrI HgiEI
 BsaOI BcgI Ksp22I BanI BstXI SinI
cggatgtcgtgatcacgggcacccgcgcccccagaccgatggccgggaccggggccgggggcgcgggggccaagc base pairs
gcctacagcactagtgcccgtgggcgcgggggtctggctaccggccctggccccggcccccgcgcccccggttcg 42826 to 42900
 BstF5I MboI Eco64I BmyI EaeI Bme18I
0I FbaI Sau3AI AccB1I AvaII
 BclI Bsp143I Bsp1286I BsmFI

 BstMCI BsoBI Esp3I
 BsiEI Eco88I BsmAI EarI
SfiI TthHB8I Ama87I HgaI Eam1104I
gggccaccgtcagcgagttcgtgcaagtgaagcacatcgaccgtgttgtgtccccgagcgtctcttccgcccccc base pairs
cccggtggcagtcgctcaagcacgttcacttcgtgtagctggcacaacacaggggctcgcagagaaggcgggggg 42901 to 42975
BglI TaqI BcoI Alw26I
 Bsh1285I AvaI BsmBI MboII
 BsaOI BsmFI Ksp632I

 Eco88I SduI EcoRII BpmI SduI BanII
 BstH2I HinfI PspAI PspALI BanII GsuI EcoO109I ApaI BstD1
 Bsp143II HgaI PleI Ama87I BglI FriOI BstOI Bsp120I FriOI AccBSI
cgccgagcgcccccgacgcgagtctgccgcccccggggctccaggaggccgccccgccgggccccccgctcaggg base pairs
gcggctcgcgggggctgcgctcagacggcgggggccccgaggtcctccggcggggcggcccggggggcgagtccc 42976 to 43050
 HaeII PshAI BcoI XmaI Bsp1286I MvaI PspOMI BmyI DdeI
 Cfr9I SmaI BmyI BstNI DraII Eco24I BsrBI
 AvaI BsoBI Eco24I Bst2UI Bsp1286I BstDEI

 BsrFI Bsh1285I BpmI Eco24I
 02I BssAI NaeI BsaOI GsuI Bsp1286I
 SgrAI Bse118I Bsp143II SduI HinfI HgaI
agctgtggtgggtgttctacgccggcgaccgggcgctggaggagccccacgccgagtcgggattgacgcgcgagg base pairs
tcgacaccacccacaagatgcggccgctggcccgcgacctcctcggggtgcggctcagccctaactgcgcgctcc 43051 to 43125
 AluI MroNI Cfr10I HaeII BseRI BanII PleI
 NgoMI BsiEI BstH2I BmyI
 NgoAIV BstMCI FriOI

 AvaII FriOI NspBII
 HgiEI BmyI DsaI SstII
 SinI NlaIII AluI SduI BstDSI Cfr42I
aggtccgcgccgtgcatgggttccgggagcaggcgtggaagctgtttgggtcggtgggggctccgcgggcgtttc base pairs
tccaggcgcggcacgtacccaaggccctcgtccgcaccttcgacaaacccagccacccccgaggcgcccgcaaag 43126 to 43200
 Bme18I Hsp92II Bsp1286I Sfr303I
 BseRI Eco24I KspI SacII
 Eco47I BanII MspA1I

Ama87I Eco24I
 Eco88I Bsp1286I BstD1
 SfiI DdeI SduI AluI AccI BstF5I AccBSI
tcggggccgcgctggccctgagcccgacccaaaagctcgccgtctactactatctcatccaccgggagcggcgca base pairs
agccccggcgcgaccgggactcgggctgggttttcgagcggcagatgatgatagagtaggtggccctcgccgcgt 43201 to 43275
 AvaI BglI BstDEI BanII FokI BsrBI
 BsoBI BmyI
BcoI FriOI

 NspI Csp6I BstDSI
 02I BstMCI FokI HaeII AfaI
 BsmFI BsiI BsiEI AfaI Bsp143II Csp6I
tgtcccccttccccgcgctcgtgcggctcgtcggtcggtacatccagcgccacggcctgtacgttcccgcgcccg base pairs
acagggggaaggggcgcgagcacgccgagcagccagccatgtaggtcgcggtgccggacatgcaagggcgcgggc 43276 to 43350
 NlaIII BssSI Bsh1285I BstF5I RsaI
 Hsp92II BsaOI BstH2I MaeII
 RsaI DsaI

 XmaIII SinI AvaII CfrI
 CfrI Hsp92II CfrI BsiEI Bme18I EaeI
 MaeII SfaNI HgaI EaeI Eco52I HgiEI BsmFI
acgaaccgacgttggccgatgccatgaacgggctgttccgcgacgcgctggcggccgggaccgtggccgagcagc base pairs
tgcttggctgcaaccggctacggtacttgcccgacaaggcgctgcgcgaccgccggccctggcaccggctcgtcg 43351 to 43425
 EaeI NlaIII BstZI BstMCI BstDSI AluI
 EagI Bsh1285I DsaI
 EclXI BsaOI Eco47I

 TaqI BssT1I Cfr10I
BbvI Hsp92II ErhI BsrFI BstH2I
 BseRI BseRI MaeII HgaI Bsp143II
tcctcatgttcgacctcctcccgcccaaggacgtgccggtggggagcgacgcgcgggccgacagcgccgccctgc base pairs
aggagtacaagctggaggagggcgggttcctgcacggccacccctcgctgcgcgcccggctgtcgcggcgggacg 43426 to 43500
 NlaIII Eco130I BssAI HaeII
Bst71I TthHB8I StyI Bse118I
 EcoT14I

 Eco88I Bme18I Ama87I Bsp1286I Csp6I
 PspAI PspALI BsmBI AvaI BmyI NlaIII
 BbvI HinfI Ama87I SinI Alw26I SduI Alw21I AfaI
tgcgctttgtggactcgcaacgcctgaccccgggggggtccgtctcgcccgagcacgtcatgtacctcggcgcgt base pairs
acgcgaaacacctgagcgttgcggactggggcccccccaggcagagcgggctcgtgcagtacatggagccgcgca 43501 to 43575
 Bst71I PleI BcoI XmaI HgiEI BsmAI Eco88I MaeII Hsp92II
 Cfr9I SmaI AvaII Esp3I BsoBI Bbv12I RsaI
 AvaI BsoBI Eco47I BcoI AspHI BsiHKAI

 MvaI BssAI EaeI Hin1I EcoRII CfrI CfrI SacII BbiII
 BstOI AfaI Bse118I DsaI BsaHI MvaI DsaI KspI BsePI MaeIII
 EcoRII Csp6I BsrFI NaeI BbiII BstNI BstDSI Sfr303I BglI Hin1I
tcctgggcgtgttgtacgccggccacggacgcctggccgcggccacgcataccgcgcgcctgacgggcgtgacgt base pairs
aggacccgcacaacatgcggccggtgcctgcggaccggcgccggtgcgtatggcgcgcggactgcccgcactgca 43576 to 43650
 BstNI RsaI NgoAIV BstDSI AcyI Bst2UI MspA1I BssHII Tsp45I
 Bst2UI MroNI Cfr10I Hsp92I BstOI NspBII SstII Msp17I
 NgoMI CfrI Msp17I HgaI EaeI EaeI Cfr42I Hsp92I

 AatII MvaI BstDSI MaeII SalI HincII AhdI BstF5I Sfr303I BsrFI
BsaHI Bst2UI AvaII BbiII BsmFI HindII Eam1105I NspBII MroNI Cfr10I
 AcyI BsmFI Eco47I Hin1I AcyI TaqI BstMCI AspEI KspI SacII Bse118I
ccctggtcctgaccgtgggggacgtcgaccggatgtccgcgtttgaccgcgggccggcgggggcggctggccgca base pairs
gggaccaggactggcaccccctgcagctggcctacaggcgcaaactggcgcccggccgcccccgccgaccggcgt 43651 to 43725
MaeII BstOI HgiEI Msp17I AatII BsiEI EclHKI DsaI SstII NgoMI EaeI
 EcoRII Bme18I Hsp92I AccI BsaWI BsaOI BstDSI Cfr42I NgoAIV CfrI
 BstNI SinI DsaI BsaHI TthHB8I Bsh1285I FokI MspA1I BssAI NaeI

 BshNI RsaI Bst2UI MvaI
 Asp718I BstNI BstOI Bse1I
 Eco64I AfaI MvaI BbvI EcoRII EaeI BsrSI
cgcgaaccgccgggtacctggacgcgctgcttaccgtttgcctggctcgcgcccagcacggccagtctgtgtgag base pairs
gcgcttggcggcccatggacctgcgcgacgaatggcaaacggaccgagcgcgggtcgtgccggtcagacacactc 43726 to 43800
 BanI Csp6I BstOI Bst71I BstNI CfrI BseNI
 Acc65I EcoRII Bst2UI BsrI
 AccB1I KpnI HgaI


 SfaNI
 Eco32I BsgI BstDSI BstF5I BsmFI
atatcccaataaagtgcagtcgttttctaacccacggatgccgttgtatgcctatacgggggactatgggggggg base pairs
tatagggttatttcacgtcagcaaaagattgggtgcctacggcaacatacggatatgccccctgatacccccccc 43801 to 43875
 EcoRV DsaI FokI


 HgaI
aaaggaaaggaaacaggaatggagaagggaaaggaacagaggcggtagcggacgcacggcggacacaataacaaa base pairs
tttcctttcctttgtccttacctcttccctttccttgtctccgccatcgcctgcgtgccgcctgtgttattgttt 43876 to 43950


 KspI SacII BsaHI HgaI
 NspBII BbiII MspA1I
 BstDSI Cfr42I HinfI Hin1I NspBII
cagaccgcggacacggagggagtcggttgggttgggcgtggacgccgctgcgtccacacacccgtttattcgcgt base pairs
gtctggcgcctgtgcctccctcagccaacccaacccgcacctgcggcgacgcaggtgtgtgggcaaataagcgca 43951 to 44025
 DsaI SstII PleI Msp17I BbvI
 MspA1I Hsp92I Bst71I
 Sfr303I AcyI HgaI

 Esp3I Eco47I BstDEI BstOI KspI SacII
 BsmAI MaeII HgiEI Bse21I FokI BstNI MspA1I
 HgaI MslI BsmFI SinI Eco81I BstF5I MvaI NspBII
ctccacaaaaatgggacgcacgttcggaccaccctaaggatgcccgccagggccgcggtaatcataacgaccccc base pairs
gaggtgtttttaccctgcgtgcaagcctggtgggattcctacgggcggtcccggcgccattagtattgctggggg 44026 to 44100
 Alw26I BstXI HgaI Bme18I DdeI Bsu36I EcoRII BstDSI Cfr42I
 BsmBI AvaII CvnI SfaNI DsaI SstII
 AocI Bst2UI Sfr303I

 Eco88I
 CfrI PspAI PspALI
 EaeI Ama87I BbvI HinfI
agcgcggacgcggccagaaacccgggggcgatggtggcgatgggcagcgtgtcaaaggccagcagatgaatcaca base pairs
tcgcgcctgcgccggtctttgggcccccgctaccaccgctacccgtcgcacagtttccggtcgtctacttagtgt 44101 to 44175
 HgaI BcoI XmaI Bst71I TfiI
 Cfr9I SmaI
 AvaI BsoBI

 MaeII Bsp1286I
 Eam1105I BshNI Bse118I
 BstDSI AhdI AflIII Eco64I BssAI
gttccgttggggaacaacaacagggccacggacggcacgtcgctggaaaacacgttcggggtgcccgccaccggc base pairs
caaggcaaccccttgttgttgtcccggtgcctgccgtgcagcgaccttttgtgcaagccccacgggcggtggccg 44176 to 44250
 DsaI EclHKI MaeII BanI SduI BsrFI
 AspEI AccB1I Cfr10I
 BmyI

 MvaI AlwNI XmaIII
 BstOI NspBII FokI MslI CfrI BsiEI
 EcoRII PvuII SfaNI BbvI NlaIII EaeI Eco52I
ccctgggccagctgctgttgggtggcatccgtgtccaccagcagcaccgacatgacctccccggccggggtgtag base pairs
gggacccggtcgacgacaacccaccgtaggcacaggtggtcgtcgtggctgtactggaggggccggccccacatc 44251 to 44325
 BstNI AluI Bst71I BstF5I Bst71I Hsp92II BstZI BstMCI
 Bst2UI MspA1I EagI Bsh1285I
 BbvI EclXI BsaOI


 Bse118I Acc16I
 BsiI BssAI FspI BglI
cgcagaaacacggcccccacgaggccgaggtcgcgccggttttcggtgcgcaccagccgcttcggctcaatctcc base pairs
gcgtctttgtgccgggggtgctccggctccagcgcggccaaaagccacgcgtggtcggcgaagccgagttagagg 44326 to 44400
 BssSI BsrFI AviII
 Cfr10I


 MaeII HincII
 BmyI BbiII AatII
 SduI BspMI HphI HphI NspBII Hin1I AcyI HindIII
cgcgcgtgcccttcgcaggtggcggtgagataggtgataaacagcgggcggcggacgtcaacgcccgtaagcttg base pairs
gcgcgcacgggaagcgtccaccgccactctatccactatttgtcgcccgccgcctgcagttgcgggcattcgaac 44401 to 44475
 Bsp1286I MspA1I Msp17I AluI
 Hsp92I
 BsaHI HindII

 Bsp143I NspBII SacII BstYI Sau3AI
 MboI DpnI MspA1I HphI BmyI NdeII
 DpnII AclWI Sfr303I Tsp45I MaeII HphI SduI BstX2I
tatccgatcccgcggggcaagggggtgtgggtgacgacgtagctggcgttgtgggtgatgggcacgaggatccgg base pairs
ataggctagggcgccccgttcccccacacccactgctgcatcgaccgcaacacccactacccgtgctcctaggcc 44476 to 44550
 NdeII BstDSI Cfr42I MaeIII AluI Bsp1286I MboI
 Sau3AI DsaI SstII BsiI BssSI
 Kzo9I AlwI KspI DpnII Bsp143I

 AclWI FriOI BsrI Bsp143I
BamHI DpnI BmyI BseNI MboI DpnI
 MflI Kzo9I Eco24I TspRI BsmFI DpnII AclWI
ggctccgcgttgtgcgacgggccgctacactggtgggtggcctccgggacgaaggcgcggatcagggcgttgtag base pairs
ccgaggcgcaacacgctgcccggcgatgtgaccacccaccggaggccctgcttccgcgcctagtcccgcaacatc 44551 to 44625
 BstI SduI BsrSI NdeII AlwI
 XhoII Bsp1286I Bse1I Sau3AI
 AlwI BanII Kzo9I

 KspI SacII BbiII AcyI MboI DpnI
 NspBII Hin1I BsaHI DpnII
 BstDSI Cfr42I NlaIII MaeII BstF5I Kzo9I
tgcgcccagcgcgtgagaacggaggccacgccgcgggtctgttgtgccatgacgtccgccgggatgtcggatcgg base pairs
acgcgggtcgcgcactcttgcctccggtgcggcgcccagacaacacggtactgcaggcggccctacagcctagcc 44626 to 44700
 DsaI SstII Hsp92II AatII FokI Bsp143I
 MspA1I Msp17I NdeII
 Sfr303I Hsp92I Sau3AI

 MluNI EcoT14I NlaIII BstNI Bsp143I Acc16I
AclWI Eco130I Bsp19I EcoRII BstF5I MboI DpnI BbvI
 CfrI StyI DsaI Hsp92II MvaI DpnII TthHB8I AluI NlaIII
gtggccatggccagcgcgtccaggatgaacccgccctcggcgagatcgaagcgcagggaagctgcgcatggggaa base pairs
caccggtaccggtcgcgcaggtcctacttgggcgggagccgctctagcttcgcgtcccttcgacgcgtacccctt 44701 to 44775
 MscI BssT1I CfrI BalI BstOI NdeII TaqI FspI Hsp92II
AlwI BalI NcoI EaeI MluNI Bst2UI Sau3AI AviII
 EaeI ErhI BstDSI MscI HgaI FokI Kzo9I Bst71I

 Eco47I Eco47I NheI Bsp143I
 HgiEI Ksp632I HgiEI BstOI MboI DpnI
 SinI MboII SinI BstNI MaeI DpnII
aagtggtccgggagccagaagaggtttttctggtggtcggtcctggctagcgcggcccggagatcggcgtgggtc base pairs
ttcaccaggccctcggtcttctccaaaaagaccaccagccaggaccgatcgcgccgggcctctagccgcacccag 44776 to 44850
 Bme18I Eam1104I Bme18I MvaI BfaI NdeII
 AvaII EarI AvaII Bst2UI Sau3AI
 EcoRII PstNHI Kzo9I

 KspI SacII BsaHI EaeI
 NspBII Hin1I AcyI AfaI DraII BbvI XmaIII
 BstDSI Cfr42I MaeII RsaI BstDSI EcoO109I NspBII CfrI
gccgcggcgacgtcggacgtacacagggccgtggttatgaggaggccccggcgggcgcgttcccgctgctcggcc base pairs
cggcgccgctgcagcctgcatgtgtcccggcaccaatactcctccggggccgcccgcgcaagggcgacgagccgg 44851 to 44925
 DsaI SstII BbiII MaeII DsaI BseRI MspA1I EagI
 MspA1I Msp17I AatII Bst71I
 Sfr303I Hsp92I Csp6I BstZI

 Eco52I BsePI MvaI AccB1I NarI HaeII XmaIII DsaI HaeII HgiEI
 BsaOI BstOI KasI BbiII EheI BstZI BsiEI Eco47III AvaII
 BsiEI BssHII BstNI Eco64I BsaHI BbeI CfrI Bsh1285I Aor51HI SinI
gagggcgcgcccgccaggaacggcgcccggaggacggccgtggcgtaaaacagcgctcggcggaccatcggggcg base pairs
ctcccgcgcgggcggtccttgccgcgggcctcctgccggcaccgcattttgtcgcgagccgcctggtagccccgc 44926 to 45000
 Bsh1285I EcoRII BanI Msp17I Bsp143II Eco52I BsaOI Bsp143II
 EclXI AscI Bst2UI BshNI Hsp92I EaeI EagI BstDSI AfeI Bme18I
 BstMCI Hin1I AcyI BstH2I EclXI BstMCI BstH2I Eco47I

 CfrI EclXI BsaOI BsaHI DpnII DpnI BsoBI
 EaeI XmaIII AfaI BbiII HgaI Bsp143I Eco88I
 BssHII NotI BstMCI Csp6I Hin1I TaqI Sau3AI Ama87I
gttagcgcgcggccgccgagaaactcggcgtacagggcgtcgatcaggcgggccgcgctcggggccaccgcgcca base pairs
caatcgcgcgccggcggctctttgagccgcatgtcccgcagctagtccgcccggcgcgagccccggtggcgcggt 45001 to 45075
 BsePI EagI Eco52I RsaI Msp17I NdeII BcoI
 BstZI BsiEI Hsp92I MboI Kzo9I AvaI
 CciNI Bsh1285I AcyI TthHB8I

 KspI SacII MvaI Eco88I
 NspBII NspBII BstOI XhoI
 BstDSI Cfr42I PvuII EcoRII AlwNI Sfr274I
taggccgcggggctgtccaacacgaacgccagctgatagcccagcgcgtgcgccaccaggctctgctctcgctcg base pairs
atccggcgccccgacaggttgtgcttgcggtcgactatcgggtcgcgcacgcggtggtccgagacgagagcgagc 45076 to 45150
 DsaI SstII AluI BstNI Ama87I
 MspA1I MspA1I Bst2UI BcoI
 Sfr303I AvaI

 DpnII Kzo9I SfaNI BsePI Eco47I
 PaeR7I Bsp143I Eco88I HgiEI
 NdeII AclWI Ama87I GsuI SinI AflIII
aggatcgcggccaccagatgcccgaggcgcgcctccagccgcaggcgggccgccgggtccaacacggacacgttc base pairs
tcctagcgccggtggtctacgggctccgcgcggaggtcggcgtccgcccggcggcccaggttgtgcctgtgcaag 45151 to 45225
 TaqI Sau3AI CfrI BcoI AscI BpmI Bme18I MaeII
 BsoBI MboI AlwI AvaI BssHII AvaII
 TthHB8I DpnI EaeI BsoBI

 EagI BsiEI Bst71I PspAI BsoBI BstOI BsrFI EaeI
 BstZI BstMCI NspBII Ama87I SmaI BstNI NgoMI BglI
 HinfI XmaIII BsaOI BbvI Eco88I EaeI MvaI NgoAIV CfrI
aggaacaccgagtcggccgcgcagcccgctgctccccgggcggccaggccggccagcacgcgcgagtgggccaaa base pairs
tccttgtggctcagccggcgcgtcgggcgacgaggggcccgccggtccggccggtcgtgcgcgctcacccggttt 45226 to 45300
 EaeI EclXI BbvI Bst71I AvaI CfrI MroNI Bse118I FseI
 CfrI Eco52I MspA1I BcoI XmaI EcoRII BssAI SfiI
 PleI Bsh1285I Cfr9I PspALI Bst2UI Cfr10I NaeI


 BspMI HinfI HgaI HincII
aagcccagcaggtcggagaggcgaatcgcgtcgtgggcgtgggccgcgttgacgaacgcaaaccccgacgaggcg base pairs
ttcgggtcgtccagcctctccgcttagcgcagcacccgcacccggcgcaactgcttgcgtttggggctgctccgc 45301 to 45375
 TfiI HindII


 AccB1I NarI HaeII Eco88I Eco24I SinI AvaII
 KasI BbiII EheI BsmFI PspAI SduI BanII EcoO109I
 BbvI Eco64I BsaHI BbeI HgaI HgaI Ama87I BmyI SmaI HgiEI
agcagccccgcgaggcgccagaacagggacggacgcgcgtccgtgccggagcccgggtcctcccccaaaaactcc base pairs
tcgtcggggcgctccgcggtcttgtccctgcctgcgcgcaggcacggcctcgggcccaggagggggtttttgagg 45376 to 45450
 Bst71I BanI Msp17I Bsp143II PshAI BcoI XmaI FriOI PpuMI Psp5II
 BshNI Hsp92I Cfr9I Bsp1286I Bme18I Eco47I
 Hin1I AcyI BstH2I AvaI BsoBI PspALI DraII

 Bbv12I Eco88I BsrFI NaeI BsaHI
 Bse1I AspHI BcoI BseRI Bse118I BbiII
 BsrSI SduI BsiHKAI MroNI Cfr10I Hin1I
gcataggcccgcgacatatactgggcgtagttcgtgctctcctcggggtagccggccacccgccggagggcgtcc base pairs
cgtatccgggcgctgtatatgacccgcatcaagcacgagaggagccccatcggccggtgggcggcctcccgcagg 45451 to 45525
 BseNI Bsp1286I BsoBI NgoAIV Msp17I
 BsrI BmyI Ama87I BssAI EaeI Hsp92I
 Alw21I AvaI NgoMI CfrI AcyI

 BsaHI EcoO109I ApaI MvaI MvaI BssAI EaeI EclXI
 BstH2I BbiII PspOMI Eco24I Bst2UI BstOI NgoAIV EagI BsiEI
 Bsp143II Hin1I Bsp120I FriOI BstOI HgaI BstNI BsrFI CfrI NaeI
agcgccgagccgttgtcggcgggcgtcggggcccccaggacaaagacgcgatacctggggccggccggaggcccg base pairs
tcgcggctcggcaacagccgcccgcagccccgggggtcctgtttctgcgctatggaccccggccggcctccgggc 45526 to 45600
 HaeII Msp17I DraII BmyI EcoRII EcoRII MroNI Cfr10I FseI
 Hsp92I SduI BanII Bst2UI Bse118I XmaIII
HgaI AcyI HgaI Bsp1286I BstNI NgoMI BstZI Eco52I

 BsaOI Cfr9I SduI BsiHKAI SstII BstMCI BsoBI Sau3AI
 BcoI Eco88I AspHI DsaI Sfr303I BsaOI Eco88I BshNI NdeII
 Bsh1285I PspALI Bbv12I MspA1I Bsh1285I Ama87I Eco64I DpnII
gggagcaccgcgggggcgttttcgtcggtcggatttccgacccgagcgagggtcttgtccgcaggcaccactatg base pairs
ccctcgtggcgcccccgcaaaagcagccagcctaaaggctgggctcgctcccagaacaggcgtccgtggtgatac 45601 to 45675
 Ama87I XmaI Bsp1286I NspBII SacII BcoI BanI MslI
 BstMCI AvaI SmaI Alw21I KspI BsiEI AvaI AccB1I MboI
 PspAI BsoBI BmyI BstDSI Cfr42I Bsp143I

 EagI BstMCI Bsp106I SfaNI SduI BanII
 BstZI Bsh1285I BanIII TaqI EcoRV Eco24I
 EaeI Eco52I BsmFI BspXI BseCI BssSI NlaIII BssHII BsiI
atctcggccggagggctgtcccgcatcgatatcacgagccccatgaagcccttcccgtatcgcgcgcgcacgagc base pairs
tagagccggcctcccgacagggcgtagctatagtgctcggggtacttcgggaagggcatagcgcgcgcgtgctcg 45676 to 45750
Kzo9I CfrI BsiEI TthHB8I ClaI BsiI BmyI Hsp92II BsePI BssSI
 DpnI EclXI BspDI BscI Eco32I FriOI XmnI
 XmaIII BsaOI Bsa29I Bsu15I Bsp1286I Asp700I

 Hsp92I BsaHI
 Msp17I BbiII DsaI TaqI
 Hin1I HgaI Hin1I BstDSI MaeII
gcggcgtcgcacccgaacgccagcccgcccgtcgtccagacgcccacgggccacgtcgaggccgacggggagagg base pairs
cgccgcagcgtgggcttgcggtcgggcgggcagcaggtctgcgggtgcccggtgcagctccggctgcccctctcc 45751 to 45825
 AcyI Msp17I TthHB8I
 BbiII Hsp92I
 BsaHI AcyI HgaI

 MaeII HinfI BstOI MvaI
 AfaI AfaI Eam1105I EcoRII CfrI BstOI SfaNI
Csp6I Csp6I AhdI PleI EcoO109I EaeI EcoRII MaeIII BstF5I BsgI
tacacgtaccgacccggagtccgtagcaggcccctggcggccagccaggtcacggatgcgttgtgcagatgcgcg base pairs
atgtgcatggctgggcctcaggcatcgtccggggaccgccggtcggtccagtgcctacgcaacacgtctacgcgc 45826 to 45900
 RsaI RsaI EclHKI DraII MvaI BstNI FokI SfaNI
 AflIII AspEI BstNI Bst2UI
 BsaAI Bst2UI Tsp45I

 MspA1I BcoI XmaI EagI Bsh1285I
 BstDEI SfaNI BsmFI SstII PspAI BsoBI HgaI Eco52I
 DdeI BstF5I BstDSI Cfr42I Eco88I EaeI EclXI
atgctcaggttcgtcgtcggatgcctcggtgtccccgcgggcggccccgggggcggcgcgttgcgtcggccgtcc base pairs
tacgagtccaagcagcagcctacggagccacaggggcgcccgccggggcccccgccgcgcaacgcagccggcagg 45901 to 45975
 SfaNI FokI DsaI Sfr303I Cfr9I SmaI BstZI BstMCI
 NspBII Ama87I PspALI CfrI BsiEI
 KspI SacII AvaI BglI XmaIII BsaOI

 BstDSI Cfr42I CspI CfrI EclXI BsaOI
 BshNI BstMCI BsmBI MspA1I Bme18I EaeI XmaIII MaeIII
 Eco64I BsiEI Alw26I Sfr303I CpoI AvaII EagI Bsh1285I
gggtgcctctcggtcgccccgtcgtctccccgcgggaacgtaagcccctcgcggtccggcgcggccgcgaatgtt base pairs
cccacggagagccagcggggcagcagaggggcgcccttgcattcggggagcgccaggccgcgccggcgcttacaa 45976 to 46050
 AccB1I Bsh1285I BsmAI NspBII MaeII SinI Eco47I NotI BstMCI
 BanI BsaOI Esp3I KspI SacII HgiEI BstZI Eco52I
 DsaI SstII RsrII CciNI BsiEI

 Bst2UI AvaI SinI BsmFI AccB1I NarI HaeII EcoO65I
 Ama87I BsoBI Eco47I KasI BbiII EheI BstEII
 BstNI Cfr9I SmaI AvaII Eco64I BsaHI BbeI BsmFI Eco91I
acccaggcccgggaccgcaacagcgcggaggcgccggggttgtgcgacagtcccttgagctgggtcacctcggcg base pairs
tgggtccgggccctggcgttgtcgcgcctccgcggccccaacacgctgtcagggaactcgacccagtggagccgc 46051 to 46125
 EcoRII BcoI XmaI HgiEI BanI Msp17I Bsp143II AluI Tsp45I
 BstOI Eco88I Bme18I BshNI Hsp92I BstPI MaeIII
 MvaI PspAI PspALI Hin1I AcyI BstH2I PspEI HphI

 EcoO109I ApaI Ecl136II SduI Eco24I BsiHKAI Bse118I FseI
 BsmFI Bsp1286I BcoI AluI AvaI Bbv12I BanII BssAI EaeI XmaIII BstMCI
 BsmFI Bsp120I FriOI Eco88I BcoI BsoBI Psp124BI MroNI Cfr10I Eco52I
gggggacgggacgtgggccccgcctcggggagctcgggcaggctcgcgttccgaggccggccgagcagataggtc base pairs
ccccctgccctgcacccggggcggagcccctcgagcccgtccgagcgcaaggctccggccggctcgtctatccag 46126 to 46200
 MaeII SduI BanII BsoBI Eco88I BmyI SacI SstI NgoAIV EagI BsiEI
 PspOMI BmyI Ama87I EcoICRI AspHI FriOI NgoMI BstZI EclXI BsaOI
 DraII Eco24I AvaI Ama87I Bsp1286I Alw21I BsrFI CfrI NaeI Bsh1285I

 BbvI PspAI PspALI AvaII Eco88I EagI BstDSI BstEII HphI HaeII
 NspBII Cfr9I SmaI EcoO109I AvaI CfrI Bsh1285I EcoO65I AfeI
 BstF5I PvuII BcoI XmaI PpuMI Eco47I BsmFI Eco52I BsaOI PspEI Eco47III
tttgggatgtaaagcagctgcccggggtcccgaggaaactcggccgtggtgaccaacacgaaacaaaagcgctcg base pairs
aaaccctacatttcgtcgacgggccccagggctcctttgagccggcaccactggttgtgctttgttttcgcgagc 46201 to 46275
 FokI AluI Ama87I BsoBI HgiEI Ama87I BstZI BsiEI Eco91I MaeIII BstH2I
 MspA1I Eco88I Bme18I Psp5II BsoBI XmaIII BstMCI Tsp45I Bsp143II
 Bst71I AvaI SinI DraII BcoI EaeI EclXI DsaI BstPI Aor51HI

 RsaI BmyI Eco88I BanI Hin1I NarI BbeI
 Csp6I SduI SfaNI PspAI PspALI Msp17I Bsp143II
 NlaIII BstF5I Ama87I Eco64I BbiII EheI
gcgtaccaccgaagcatgggcacggatgccgtagtcaggttgagttcgcccgggggcgccaagcgtccgcgctgg base pairs
cgcatggtggcttcgtacccgtgcctacggcatcagtccaactcaagcgggcccccgcggttcgcaggcgcgacc 46276 to 46350
 Hsp92II FokI BcoI XmaI KasI Hsp92I HaeII
 Bsp1286I Cfr9I SmaI BshNI BsaHI BstH2I
 AfaI AvaI BsoBI AccB1I AcyI HgaI

 BsaHI BsaHI Bse1I
 BbiII BbiII BseNI
 Hin1I Hin1I Csp6I AfaI
gggtcgctggcgtcgggggtgttgggcaaccacagacgcccggtgtttgtgtcgcgccagtacgtgcgggccaac base pairs
cccagcgaccgcagcccccacaacccgttggtgtctgcgggccacaaacacagcgcggtcatgcacgcccggttg 46351 to 46425
 Msp17I Msp17I BsrSI
 Hsp92I Hsp92I RsaI MaeII
 AcyI HgaI AcyI HgaI BsrI BsaAI

 Bse1I MvaI
 BseNI Hsp92II BstOI
 BstDSI TthHB8I Csp6I AfaI NlaIII EcoRII
cccagaccgtgcaaaaaccacgggtcgatttgctccgtccagtacgtgtcatggcccccggcaacgcccaccagg base pairs
gggtctggcacgtttttggtgcccagctaaacgaggcaggtcatgcacagtaccgggggccgttgcgggtggtcc 46426 to 46500
 DsaI TaqI BsrSI BsaAI BstNI
 RsaI MaeII Bst2UI
 BsrI AflIII SinI

 PpuMI Psp5II Eco130I NcoI DsaI BsmFI BsrI
 EcoO109I EcoO109I StyI FriOI Bsp19I BseNI
 Bme18I Eco47I Bsp120I ErhI EcoT14I NlaIII BsrSI
acccccatcaccacccacagaccggggcccatggtcgtcgtcccggctgccagtccgcagatgggggggggtgtc base pairs
tgggggtagtggtgggtgtctggccccgggtaccagcagcagggccgacggtcaggcgtctacccccccccacag 46501 to 46575
 HgiEI HphI PspOMI BmyI Eco24I ApaI BbvI XcmI
 DraII DraII Bsp1286I BanII Hsp92II Bse1I
 AvaII SduI BssT1I BstDSI Bst71I

 DsaI
 AfaI
 Csp6I EcoO109I
cgtacccacggcccaaagaggctccgcacctcggaggctatcggaggccctttgttgccgtaagcgcgggccaaa base pairs
gcatgggtgccgggtttctccgaggcgtggagcctccgatagcctccgggaaacaacggcattcgcgcccggttt 46576 to 46650
 RsaI DraII
 BstDSI


 BstF5I Bsp143I
 Esp1396I AfaI MboI DpnI BstD1
 AccB7I HphI Csp6I DpnII AclWI AccBSI
ggatggggtggggtgagggtaaaagcacaaagggagtaccagaccgaaaacaaggacggatcggcccgctccgtt base pairs
cctaccccaccccactcccattttcgtgtttccctcatggtctggcttttgttcctgcctagccgggcgaggcaa 46651 to 46725
 PflMI RsaI NdeII AlwI BsrBI
 Van91I Sau3AI
 FokI Kzo9I

 MvaI
 02I BshNI BstOI
 Eco64I EcoRII
tttcggtggggtgctgatacggtgccagccctggccccgaacccccgcgcttatggacacaccacacgacaacaa base pairs
aaagccaccccacgactatgccacggtcgggaccggggcttgggggcgcgaatacctgtgtggtgtgctgttgtt 46726 to 46800
 BanI BstNI
 AccB1I Bst2UI


 SseBI
 AatI
 BcgI Pme55I
tgccttttattctgttcttttattgccgtcatcgccgggaggccttccgttcgggcttccgtgtttgaactaaac base pairs
acggaaaataagacaagaaaataacggcagtagcggccctccggaaggcaagcccgaaggcacaaacttgatttg 46801 to 46875
 StuI
 Eco147I


 Bst2UI Eco47I
 BsePI BstNI HgiEI
 MaeII EcoRII SinI Tsp45I HgaI
tccccccacctcgcgggcaaacgtgcgcgccaggtcgcgtatctcggcgatggacccggcggttgtgacgcgggt base pairs
aggggggtggagcgcccgtttgcacgcgcggtccagcgcatagagccgctacctgggccgccaacactgcgccca 46876 to 46950
 BssHII Bme18I MaeIII
 BstOI AvaII
 MvaI

 Bsp143I FokI BsaHI Alw26I Bsp143I Bse8I MvaI
 MboI DpnI BbiII TaqI MboI DpnI EcoRII
 DpnII AclWI HphI Hin1I TthHB8I DpnII MamI BstNI
tgggatcatcccggcggtgaggcgcaacagggcgtctcgacacccgacgggcgactgatcgtaatccaggacaaa base pairs
accctagtagggccgccactccgcgttgtcccgcagagctgtgggctgcccgctgactagcattaggtcctgttt 46951 to 47025
 NdeII AlwI Msp17I BsmAI NdeII BsaBI BstOI
 Sau3AI BstF5I Hsp92I BsmBI Sau3AI Bsh1365I
 Kzo9I AcyI HgaI Esp3I Kzo9I BsrBRI Bst2UI

 EcoT22I EaeI MaeII MvaI
 Zsp2I BstMCI BbiII AatII BstOI AfaI
 Ppu10I SfaNI BsiEI Hin1I AcyI BstNI Csp6I BsmFI
tagatgcatcggaaggaggcggtcggccaagacgtccaagacccaggcaaaaatgtggtacaagtccccgttggg base pairs
atctacgtagccttcctccgccagccggttctgcaggttctgggtccgtttttacaccatgttcaggggcaaccc 47026 to 47100
 SfaNI Bsh1285I Msp17I EcoRII RsaI
 NsiI BsaOI Hsp92I Bst2UI
 Mph1103I CfrI BsaHI

 AvaI BsoBI
 BcoI Bst71I Eco31I Eco88I
 AluI BsoBI TthHB8I Alw26I Ama87I
ggccagcagctcgggaacgcggaacagggcaaacagcgtgtcctcgatgcggggcagagaccccgcgccgtcctc base pairs
ccggtcgtcgagcccttgcgccttgtcccgtttgtcgcacaggagctacgccccgtctctggggcgcggcaggag 47101 to 47175
 Ama87I TaqI SfaNI BsmAI BcoI
 Eco88I BsaI AvaI
 BbvI

 KspI SacII NgoAIV BsrI
 NspBII NgoMI BseNI
 BstDSI Cfr42I MroNI Cfr10I SfcI
ggggtcggggcgcggggtcgccgcggcgacccccgtcagccggccccagtcctcccgccacctcccgccgcgctg base pairs
ccccagccccgcgccccagcggcgccgctgggggcagtcggccggggtcaggagggcggtggagggcggcgcgac 47176 to 47250
 DsaI SstII PshAI BssAI NaeI BsrSI BstSFI
 MspA1I BsrFI Bse1I
 Sfr303I Bse118I

 PstI BshNI KpnI Bsp143I MscI BstNI
 BbvI Acc65I BspMI MboI DpnI NlaIII BstOI
 Eco64I RsaI DpnII AccI EaeI BalI
caggtaccgcaccgtgttggcgagtagatcgtagacacggcgaatggcggacagcatggccaggtcaagccgctc base pairs
gtccatggcgtggcacaaccgctcatctagcatctgtgccgcttaccgcctgtcgtaccggtccagttcggcgag 47251 to 47325
 Asp718I AfaI NdeII CfrI EcoRII
 Bst71I AccB1I Sau3AI Hsp92II MvaI
 BanI Csp6I Kzo9I MluNI Bst2UI

 BcoI AvaI SmaI AcyI MscI Bst2UI BsaWI MvaI HgiEI BstNI
BstD102I BsoBI BbiII CfrI BstOI BsaOI MroI Bsp13I BstOI Eco47I MvaI
AccBSI Eco88I Msp17I EaeI BstNI BstMCI BspEI BsiMI EcoRII Bme18I BstOI
gcccgggcgttggcgtctggccaggcggtcggcgtgttcggcctccggaaggacacccaggaccaggttcgtgcc base pairs
cgggcccgcaaccgcagaccggtccgccagccgcacaagccggaggccttcctgtgggtcctggtccaagcacgg 47326 to 47400
BsrBI Cfr9I SrfI BsaHI MluNI BsiEI AccIII BstNI AvaII Bst2UI
 Ama87I PspALI Hsp92I BalI MvaI Kpn2I EcoNI Bst2UI SexAI
 PspAI XmaI Hin1I HgaI EcoRII Bsh1285I BseAI SinI EcoRII

 MvaI Asp718I KpnI
 BstMCI BstOI Eco64I RsaI
 BsiEI NlaIII BglI BstNI NlaIII NlaIII BshNI
gggcgcggtcgggggcatgagggccacgaacgccaacacggcctggggggtcatgcttcccatgaggtaccgcgc base pairs
cccgcgccagcccccgtactcccggtgcttgcggttgtgccggaccccccagtacgaagggtactccatggcgcg 47401 to 47475
 Bsh1285I Hsp92II EcoRII Hsp92II Hsp92II Csp6I
 BsaOI Bst2UI BanI AccB1I
 Acc65I AfaI

 EclXI BsaOI BssAI Bsh1285I
 CfrI BsiEI BshNI Cfr10I BsaOI
 EaeI Eco52I Eco64I TthHB8I HphI Bsp12
ggccgggtagcacagcagggaggcgatagggtgccggtcgaaaacaagggtgagggccgggggcggggcttgcgg base pairs
ccggcccatcgtgtcgtccctccgctatcccacggccagcttttgttcccactcccggcccccgccccgaacgcc 47476 to 47550
 BstZI BstMCI BanI Bse118I BstMCI PspOM
 EagI Bsh1285I AccB1I BsiEI
 XmaIII BsrFI TaqI

 BmyI Hsp92I Bbv12I
 SduI ApaI Msp17I AspHI
 0I BanII BseRI BglI AcyI SduI BsiHKAI
gcccacagcctcccccccgatatgaggagccaaaacggcgtccgtcgccgcataaggcgtgctcattgttatctg base pairs
cgggtgtcggagggggggctatactcctcggttttgccgcaggcagcggcgtattccgcacgagtaacaatagac 47551 to 47625
 I FriOI Hin1I HgaI Bsp1286I
 Bsp1286I BbiII BmyI
 Eco24I BsaHI Alw21I

 EagI Bsh1285I NspBII SinI
 BstH2I BstZI BstMCI DsaI SstII HgiEI Bst71I
 Bsp143II BglI EclXI Eco32I KspI SacII Eco47I
ggcgctggtcattaccaccgccgcctccccggccgatatctcgccgcggtccagacggtgctgcgtgttgtagat base pairs
ccgcgaccagtaatggtggcggcggaggggccggctatagagcggcgccaggtctgccacgacgcacaacatcta 47626 to 47700
 HaeII EaeI Eco52I BstDSI Cfr42I BbvI
 CfrI BsiEI EcoRV Sfr303I AvaII
 XmaIII BsaOI MspA1I Bme18I

 EcoO109I BsrI BsoBI
 BsaI BseNI Eco88I AccI
 Alw26I AlwNI BsrSI Ama87I MaeII DrdI
gttcgtcagggtctcggaggcccccagcacctgccagtaagtcatcggctcggggacgtagacgatattgtcgcg base pairs
caagcagtcccagagcctccgggggtcgtggacggtcattcagtagccgagcccctgcatctgctataacagcgc 47701 to 47775
 Eco31I BspMI BcoI BsmFI
 BsmAI Bse1I AvaI
 DraII

 MvaI BbvI MboII DraIII AvaII
 BstOI NspBII BbsI DsaI Van91I
 EcoRII DraII PvuII BpuAI BstDSI SinI
cggccccagggcctccatcagctgcgcggaggtggtggtcttccccaccccgtggggtccgtctatataaacccg base pairs
gccggggtcccggaggtagtcgacgcgcctccaccaccagaaggggtggggcaccccaggcagatatatttgggc 47776 to 47850
 BstNI AluI Bbv16II AccB7I HgiEI
 Bst2UI MspA1I BpiI PflMI Bme18I
 EcoO109I Bst71I Esp1396I Eco47I

 AccIII BstYI Bsp143I AclWI Sfr303I AccIII Bsp13I BstNI MaeI
 Bst71I Bsp13I Sau3AI Kzo9I MspA1I Bsp1286I BseAI EcoO109I Msp17I
 BbvI AluI Kpn2I DpnII BamHI DpnI NspBII SduI MroI FriOI EcoRII BfaI
cagcagcgtgggcagctccggatccccgcgggctccggaggccccctggcgatggctaggacgggacgccgcgcg base pairs
gtcgtcgcacccgtcgaggcctaggggcgcccgaggcctccgggggaccgctaccgatcctgccctgcggcgcgc 47851 to 47925
 Bst71I BbvI BsaWI BstX2I MflI AlwI KspI SacII Kpn2I BanII BstOI Hin1I
 BspEI BsiMI MboI BstI BstDSI Cfr42I BmyI Eco24I DraII MvaI
 MroI BseAI NdeII XhoII DsaI SstII BspEI BsaWI BsiMI Bst2UI

 BsaHI CfrI BsiEI BstD102I NgoMI
 HgaI EclXI AccBSI BbvI MroNI Cfr10I
 AcyI BstZI BstMCI BsiI EaeI NgoAIV
gccgtcggtaggcccgctcgcacgagcagcctgaccgaacgcaggcgcgtgctgttggccggcgtgagaagccat base pairs
cggcagccatccgggcgagcgtgctcgtcggactggcttgcgtccgcgcacgacaaccggccgcactcttcggta 47926 to 48000
 BsmFI XmaIII BsaOI BssSI CfrI Bse118I
 BbiII EaeI Eco52I BsrBI Bst71I BssAI NaeI
 Hsp92I EagI Bsh1285I BsrFI

 BsoBI MvaI
 Eco88I BstOI
 Ama87I EcoRII BbvI
acccgcttctacaaggcgttcgcccgagaggtgcgggagttcaacgccaccaggatttgtggaacgctgctgacg base pairs
tgggcgaagatgttccgcaagcgggctctccacgccctcaagttgcggtggtcctaaacaccttgcgacgactgc 48001 to 48075
 BcoI BstNI Bst71I
 AvaI Bst2UI


 BstSFI Tru1I Eco47I
 BstD102I PstI HgaI HgiEI
 HgaI AccBSI BbvI TthHB8I Tsp45I Tru9I SinI BcoI
ctgatgagcgggtcgctgcagggtcgctcgctgttcgaggccacgcgcgtcaccttaatatgcgaagtggacctc base pairs
gactactcgcccagcgacgtcccagcgagcgacaagctccggtgcgcgcagtggaattatacgcttcacctggag 48076 to 48150
 BsrBI Bst71I TaqI MaeIII MseI Bme18I
 SfcI HphI AvaII
 Ama87I

 BstBI Bpu14I
Eco88I LspI NspV ApoI Psp1406I
 SfaNI TthHB8I AcsI Tsp509I HgaI
gggccgcgccgcccagactgcatctgcgtgttcgaattcgccaatgacaaaacgttgggaggtgtgtgcgtcatc base pairs
cccggcgcggcgggtctgacgtagacgcacaagcttaagcggttactgttttgcaaccctccacacacgcagtag 48151 to 48225
 BsoBI MslI SfuI TaqI TspEI MaeII EcoRII
AvaI Csp45I Sse9I
 Bsp119I EcoRI

 BstOI BpmI BspDI BscI HgiEI
 BstF5I NspI TthHB8I Bsu15I CfrI SinI DsaI
 Bst2UI NlaIII Bsa29I ClaI BsmFI NspBII Eco47I
ctggagctaaagacatgcaaatcgatttcttccggggacacggccagcaaacgcgaacagcggaccacgggcatg base pairs
gacctcgatttctgtacgtttagctaaagaaggcccctgtgccggtcgtttgcgcttgtcgcctggtgcccgtac 48226 to 48300
 MvaI Hsp92II BspXI MboII EaeI MspA1I BstDSI
 BstNI GsuI BanIII TaqI Bme18I MslI
 FokI AluI Bsp106I BseCI AvaII

 NspBII BstSFI DrdI
 PvuII SfcI AspI
NlaIII BbvI Eco57I PstI BsmFI AccI
aagcagctgcgccactccctgaagctgctgcagtcgctcgcgcctccgggggacaaggtcgtctacctgtgtcct base pairs
ttcgtcgacgcggtgagggacttcgacgacgtcagcgagcgcggaggccccctgttccagcagatggacacagga 48301 to 48375
Hsp92II Bst71I AluI Tth111I
 AluI BbvI AtsI
 MspA1I Bst71I

 BbvI Csp6I MboI Bsp143I
 Pfl23II BbvI BstX2I Kzo9I
 SplI Bst71I HgaI Tsp45I BsmFI BstYI MflI
attttggtgtttgtcgcgcagcgtacgctgcgcgtcagccgcgtgacccggctcgtcccgcaaaagatctccggc base pairs
taaaaccacaaacagcgcgtcgcatgcgacgcgcagtcggcgcactgggccgagcagggcgttttctagaggccg 48376 to 48450
 PspLI AfaI MaeIII DpnII BglII
 BsiWI Bst71I NdeII XhoII
 SunI RsaI Sau3AI DpnI

 MspA1I SstII Eco52I FokI
 HphI CfrI XmaIII BsaOI
 BstDSI EagI EclXI Bsh1285I MaeII DrdI
aacatcaccgcggccgtgcggatgctccaaagcctgtccacgtatgccgtgccgccggaaccgcagacccggcgg base pairs
ttgtagtggcgccggcacgcctacgaggtttcggacaggtgcatacggcacggcggccttggcgtctgggccgcc 48451 to 48525
 DsaI BstZI Cfr42I BstMCI BsaAI
 NspBII Sfr303I BsiEI SfaNI
 EaeI KspI SacII BstF5I

 BsaOI BshNI BsaHI BstH2I PmlI BbrPI
 Bsh1285I Hin1I AcyI BsiEI Eco72I
 Eco64I BbiII Bsp143II EcoO109I AflIII Tsp45I
tcgcggcgccgggtcgccgcgaccgccagaccgcaaaggcccccctccccgacacgtgacccggaaggcacggcg base pairs
agcgccgcggcccagcggcgctggcggtctggcgtttccggggggaggggctgtgcactgggccttccgtgccgc 48526 to 48600
 BsiEI KasI Hsp92I HaeII BstMCI DraII MaeII MaeIII
 BstMCI AccB1I NarI BbeI BsaOI BsaAI
 BanI Msp17I EheI Bsh1285I PmaCI

 MvaI BsaHI
 BstOI BbiII
 BstF5I EcoRII Hin1I BbvI
ggtcatccggccccaccagagagcgaccccccctccccaggggtcgtaggcgtcgctgcggagggtgggggtgtg base pairs
ccagtaggccggggtggtctctcgctgggggggaggggtccccagcatccgcagcgacgcctcccacccccacac 48601 to 48675
 FokI BstNI Msp17I Bst71I
 Bst2UI Hsp92I
 AcyI HgaI

 EaeI Eco47I
 BstH2I Bse118I HgiEI
 Eco57I Bsp143II BssAI SinI
cttcagaaaatcgcggcgcttttttgcgtgccggtggccgccaagagcagaccccggaccaaaaccgagtgaggt base pairs
gaagtcttttagcgccgcgaaaaaacgcacggccaccggcggttctcgtctggggcctggttttggctcactcca 48676 to 48750
 HaeII BsrFI Bme18I
 Cfr10I AvaII
 CfrI


 EarI
 Eam1104I EaeI
tctgtgtgttgtttttttttttttttttcctcgttttgttttctcttctttcccccccccctcccccgcttctgg base pairs
agacacacaacaaaaaaaaaaaaaaaaaggagcaaaacaaaagagaagaaaggggggggggagggggcgaagacc 48751 to 48825
 Ksp632I CfrI
 MboII


BalI BspMI MseI KspI SacII BsrFI
MscI FokI AflII Tru9I NspBII BssHII PleI BssAI NaeI
 SfaNI HphI MspCI BstDSI Cfr42I HinfI DrdI SgrAI Bse118I
ccaagcatcctcacctgcttaagcggaacccgcgggcgcgcggggactcatttgtcgccggcgacacccacccga base pairs
ggttcgtaggagtggacgaattcgccttgggcgcccgcgcgcccctgagtaaacagcggccgctgtgggtgggct 48826 to 48900
 BstF5I BspTI BfrI DsaI SstII BsmFI MroNI Cfr10I
MluNI Bst98I Tru1I MspA1I BsePI NgoMI
 Vha464I Sfr303I NgoAIV

 Bst2UI HincII PshAI
 BstNI AccI Bsh1285I
 EcoRII SalI BsiEI MspA1I MaeII
caacagcccctgggtgtcgaccgctgtcgcccccgtctgtcgcctctcccttttttccccccctcaaagaacgtg base pairs
gttgtcggggacccacagctggcgacagcgggggcagacagcggagagggaaaaaagggggggagtttcttgcac 48901 to 48975
 AlwNI TthHB8I BsaOI
 BstOI TaqI BstMCI
 MvaI HindII NspBII

 AccB1I NarI BssAI Bsp143II Tsp509I HgiEI Csp6I
 KasI BbiII EheI Bse118I EaeI MboII Bme18I AfaI
 Eco64I BsaHI BsrFI BstH2I TspEI TthHB8I NlaIII RsaI
gtgttgggcgccggccaattcttcccggagcgccgtcgtcgcccgcccgccgccctcgaacatggacccgtacta base pairs
cacaacccgcggccggttaagaagggcctcgcggcagcagcgggcgggcggcgggagcttgtacctgggcatgat 48976 to 49050
 BanI Msp17I MroNI Cfr10I NaeI HaeII TaqI SinI Eco47I
 BshNI Hsp92I NgoAIV BbeI Sse9I BstH2I Hsp92II
 Hin1I AcyI NgoMI HaeII CfrI Bsp143II AvaII

 BstOI AvaI
 BstH2I PleI GsuI BcoI
 TthHB8I MaeII Bsp143II HinfI MvaI AluI HphI
ccctttcgacgcgctggacgtttgggaacacaggcgcttcatcgtcgccgactccaggagcttcatcacccccga base pairs
gggaaagctgcgcgacctgcaaacccttgtgtccgcgaagtagcagcggctgaggtcctcgaagtagtgggggct 49051 to 49125
 TaqI HgaI HaeII EcoRII BpmI Ama87I
 BstNI Eco88I
 Bst2UI BsoBI

 Eco88I PspAI BsoBI
 PspAI PspALI Ama87I SmaI Esp3I BstD102I BstSFI
 Ama87I BsmFI BstF5I BstF5I XmaI BsmAI AccBSI TspRI
gttcccccgggacttctggatgttgcccgtgttcaacatcccccgggagacggcggcggagcgggcggcagtgct base pairs
caagggggccctgaagacctacaacgggcacaagttgtagggggccctctgccgccgcctcgcccgccgtcacga 49126 to 49200
 BcoI XmaI FokI FokI Eco88I BsmBI BsrBI SfcI
 Cfr9I SmaI BcoI AvaI Alw26I
 AvaI BsoBI Cfr9I PspALI

 Bst71I EaeI EagI XmaIII DsaI MspA1I BglI GsuI Bst2UI Bst71I HindII
 NspBII KspI EclXI BstDSI Sfr303I BstNI BpmI MvaI TthHB8I
 BstDSI CciNI NotI Bsh1285I KspI SfiI Bst2UI BglI BbvI HincII
gcaggcccagcgcaccgcggccgcggcggccctggagaacgccgccctccaggccgccgagctgcccgtcgacat base pairs
cgtccgggtcgcgtggcgccggcgccgccgggacctcttgcggcgggaggtccggcggctcgacgggcagctgta 49201 to 49275
 DsaI CfrI Cfr42I Eco52I NspBII SacII MvaI BstNI GsuI SalI
 BbvI MspA1I Sfr303I BsiEI BsaOI Cfr42I BstOI BstOI BpmI AccI
 PstI BstZI SstII SacII BstMCI SstII EcoRII EcoRII AluI TaqI

 Bsp143I Bsh1285I BsaHI BstOI BstH2I BsmAI
 HaeII MboI DpnI TaqI Bst71I BbiII BstNI GsuI GsuI Eco31I
 Bsp143II DpnII BspCI PvuI BspMI Hin1I EcoRII Bsp143II DsaI
cgagcgccggatacgcccgatcgagcagcaggtgcatcacatcgccgacgccctggaggcgctggagaccgcggc base pairs
gctcgcggcctatgcgggctagctcgtcgtccacgtagtgtagcggctgcgggacctccgcgacctctggcgccg 49276 to 49350
TthHB8I NdeII TthHB8I BsaOI SfaNI Msp17I Bst2UI HaeII Alw26I
TaqI BstH2I Sau3AI Ple19I BbvI Hsp92I MvaI BpmI BpmI BsaI
 Kzo9I BsiEI BstMCI AcyI HgaI BglI BstDSI

NspBII EaeI XmaIII BstMCI CfrI XmaIII BsaOI Ksp632I Hsp92I BstH2I BstH2I
 Cfr42I EagI Bsh1285I BstZI Cfr42I BstMCI BstF5I BbiII HgaI Bsp143II
 MspA1I BstZI Eco52I BsaOI EagI EclXI Bsh1285I FokI Msp17I Bsp143II BbvI
ggccgcggccgaagaggcggatgccgcgcgggacgccgaggcgaggggggagggcgctgcggacggggcagcgcc base pairs
ccggcgccggcttctccgcctacggcgcgccctgcggctccgctcccccctcccgcgacgcctgccccgtcgcgg 49351 to 49425
 KspI SacII NotI BstDSI MspA1I SstII Eco52I Eam1104I BsaHI HaeII Bst71I
 SstII CfrI EclXI DsaI EaeI Sfr303I BsiEI MboII SfaNI AcyI BbvI HaeII
 Sfr303I CciNI BsiEI NspBII KspI SacII BglI EarI Hin1I BsmFI Bst71I

 KspI SacII Bsp1286I DsaI Cfr42I Bsp143I SunI
 NspBII Bsp120I Eco24I MspA1I NdeII PspLI AfaI
 BcgI BstDSI Cfr42I SduI BanII KspI SacII Kzo9I BsiWI
gtcgcccaccgcgggccccgccgccgcggagatggaggttcagatcgtacgcaacgacccgccgctacgatacga base pairs
cagcgggtggcgcccggggcggcggcgcctctacctccaagtctagcatgcgttgctgggcggcgatgctatgct 49426 to 49500
 DsaI SstII EcoO109I ApaI Sfr303I DpnII SplI RsaI
 MspA1I PspOMI BmyI BstDSI SstII Sau3AI Pfl23II
 Sfr303I DraII FriOI NspBII MboI DpnI Csp6I

 BstX2I DpnI DraIII KspI SacII SstII
 DsaI MboI MflI AlwI RsaI NspBII BstDSI Cfr42I
 XcmI BstYI Bsp143I NlaIII BstDSI Cfr42I KspI HinfI
taccaacctccccgtggatctgctacacatggtgtacgcgggccgcggggccgcgggttcgtcgggagtcgtctt base pairs
atggttggaggggcacctagacgatgtgtaccacatgcgcccggcgccccggcgcccaagcagccctcagcagaa 49501 to 49575
 BstDSI Sau3AI AclWI Csp6I DsaI SstII NspBII SacII PleI
 DpnII XhoII Hsp92II MspA1I DsaI Sfr303I
 NdeII Kzo9I AfaI Sfr303I MspA1I

 BshNI RsaI BstOI Acc65I KpnI Kzo9I AclWI BstH2I
 Asp718I KpnI MvaI BshNI AfaI Bsp143I AlwI Bsp143II
 Eco64I AfaI Bst2UI AccB1I Sau3AI BstOI BbvI TthHB8I
tggtacctggtaccgcacgatccaggaacgcaccatcgcggacttccccctgaccacccgcagcgccgactttcg base pairs
accatggaccatggcgtgctaggtccttgcgtggtagcgcctgaagggggactggtgggcgtcgcggctgaaagc 49576 to 49650
 BanI Csp6I BstNI Asp718I DpnII EcoRII Bst71I TaqI
 Acc65I SexAI Eco64I RsaI NdeII BstNI MvaI HaeII
 AccB1I EcoRII BanI Csp6I MboI DpnI Bst2UI

 Esp3I Eco47I PstI XmaIII NgoAIV BsaOI
 BsmAI NspI NlaIII HgiEI BsmAI CfrI MroNI Bse118I
 NlaIII BspHI SinI Alw26I EaeI Eco52I BsiEI
agacgggcgcatgtccaagaccttcatgaccgcgctggtcctgtctctgcagtcgtgcggccggctgtacgtggg base pairs
tctgcccgcgtacaggttctggaagtactggcgcgaccaggacagagacgtcagcacgccggccgacatgcaccc 49651 to 49725
 Alw26I Hsp92II RcaI Bme18I SfcI BstZI NgoMI Bsh1285I
 BsmBI Hsp92II AvaII BstSFI EagI BssAI Cfr10I
 EclXI BsrFI BstMCI

 MaeII
 RsaI HaeII AfaI BssSI
 NaeI Bsp143II TthHB8I Csp6I HinfI
ccagcgccactattccgccttcgagtgcgccgtgctgtgtctgtatctgctgtaccgaaccacccacgagtcctc base pairs
ggtcgcggtgataaggcggaagctcacgcggcacgacacagacatagacgacatggcttggtgggtgctcaggag 49726 to 49800
 BsaAI TaqI RsaI BsiI
 Csp6I BstH2I PleI
 AfaI

 Bsp143I PvuI BsaBI DpnII DpnI SgfI SinI AvaII MvaI
 MboI DpnI BstMCI NruI MboI BsiEI BsaOI HgiEI BsmFI BstOI CfrI
 DpnII BspCI BsaOI Bse8I Sau3AI Ple19I PpuMI Psp5II EcoRII HgaI
ccccgatcgcgatcgcgctcccgttgcgttcggggacctgctggcccgcctgccgcgctacctggcgcgtctggc base pairs
ggggctagcgctagcgcgagggcaacgcaagcccctggacgaccgggcggacggcgcgatggaccgcgcagaccg 49801 to 49875
 NdeII BsiEI MamI Bsp68I Bsp143I PvuI Bme18I Eco47I BstNI EaeI
 Sau3AI Bsh1285I Bsh1365I Kzo9I Bsh1285I DraII BspMI Bst2UI
 Kzo9I Ple19I BsrBRI NdeII BspCI BstMCI EcoO109I

DsaI SstII Msp17I RsaI
 KspI SacII BstD102I Csp6I Bst71I
 NspBII AccBSI BsaHI AluI
cgcggtaatcggcgacgagagcggacgcccgcagtaccgctaccgcgacgacaagctgcccaaagcgcagttcgc base pairs
gcgccattagccgctgctctcgcctgcgggcgtcatggcgatggcgctgctgttcgacgggtttcgcgtcaagcg 49876 to 49950
 MspA1I BsrBI Hsp92I BbvI
BstDSI Cfr42I Hin1I AcyI AfaI
 Sfr303I BbiII HgaI

 XmaIII NgoAIV BsaOI CciNI Bsh1285I Bbv12I DraII Eco24I EaeI BalI MboI MslI
 CfrI MroNI Bse118I BstZI Eco52I AspHI Bsp120I Bsp1286I Bst2UI DpnII DpnI
 EaeI Eco52I BsiEI NaeI NotI BstMCI BmyI PspOMI BmyI BstNI MscI NdeII MaeII
ggcggccggcggccgctacgagcacggggccctggccacccacgtcgtgatcgccacgttggtgcgccacggggt base pairs
ccgccggccgccggcgatgctcgtgccccgggaccggtgggtgcagcactagcggtgcaaccacgcggtgcccca 49951 to 50025
 BstZI NgoMI Bsh1285I EagI BsiEI Bsp1286I EcoO109I ApaI BstOI DraIII Kzo9I
 EagI BssAI Cfr10I EaeI XmaIII BsaOI BsiHKAI SduI FriOI MvaI MluNI Sau3AI
 EclXI BsrFI BstMCI CfrI EclXI SduI Alw21I EcoRII BanII CfrI MaeII Bsp143I

 BsrFI Eco88I BsoBI
 BstDSI PspAI PspALI Eco88I
 BssAI Ama87I MaeII Ama87I Alw26I MslI MaeII
gctaccggcggccccgggcgacgttccccgagacaccagcacccgcgtgaaccccgacgacgtggcccaccgcga base pairs
cgatggccgccggggcccgctgcaaggggctctgtggtcgtgggcgcacttggggctgctgcaccgggtggcgct 50026 to 50100
 DraIII BcoI XmaI BcoI BsmAI
 DsaI Cfr10I Cfr9I SmaI AvaI
 Bse118I AvaI BsoBI

 Msp17I HincII EcoNI
 AtsI MaeII HindII EarI HgiEI BbvI
 Tth111I AatII EaeI Eam1104I SinI AlwNI
cgacgtcaaccgcgccgccgccgcgtttttggcacgcggccacaacctcttcctgtgggaggaccagacgctgct base pairs
gctgcagttggcgcggcggcggcgcaaaaaccgtgcgccggtgttggagaaggacaccctcctggtctgcgacga 50101 to 50175
 AspI Hsp92I CfrI Ksp632I Bme18I HgaI
 Hin1I BsaHI MboII AvaII Bst71I
 BbiII AcyI Eco47I

 Bst2UI AfaI CspI
 BstMCI BstNI NruI Csp6I Bme18I
 BsiEI XcmI EaeI BseRI MaeII CpoI
gcgggcgaccgccaacaccattacggccctggccgtgcttcggcggctcctcgcgaacggcaacgtgtacgcgga base pairs
cgcccgctggcggttgtggtaatgccgggaccggcacgaagccgccgaggagcgcttgccgttgcacatgcgcct 50176 to 50250
 Bsh1285I EcoRII CfrI Bsp68I AflIII SinI
 BsaOI BstOI RsaI HgiEI
 MvaI RsrII

 NspBII BbuI NdeII BcoI XmaI SmaI BsoBI BsaHI
 TthHB8I PstI Bst71I SphI Bsp143I Eco88I BglI Eco88I BbiII
 AvaII SfcI PvuII PaeI DpnII Ama87I BsoBI BsmFI Ama87I Hin1I
ccgcctcgacaaccgcctgcagctgggcatgctgatcccgggagccgtcccggcggaggccatcgctcggggggc base pairs
ggcggagctgttggcggacgtcgacccgtacgactagggccctcggcagggccgcctccggtagcgagccccccg 50251 to 50325
 Eco47I BstSFI BbvI NlaIII Sau3AI PspAI AclWI BcoI Msp17I
 TaqI AluI Hsp92II Kzo9I Cfr9I AlwI AvaI Hsp92I
 MspA1I NspI MboI DpnI AvaI PspALI AcyI

 Kpn2I HgaI Eco88I Hin1I NarI BstH2I MvaI BpmI HpaI
 MroI Bsp13I BsoBI AccB1I AcyI BstOI HaeII MseI AfaI
 AccIII HinfI PleI BshNI BsaHI BbeI EcoRII GsuI Tru9I Csp6I
gtccggattggactcgggcgccataaaaagcggcgacaacaacctggaggcgctgtgcgttaactatgtacttcc base pairs
caggcctaacctgagcccgcggtatttttcgccgctgttgttggacctccgcgacacgcaattgatacatgaagg 50326 to 50400
 BsaWI Ama87I BanI Msp17I Bsp143II BstNI Bsp143II HindII RsaI
 BseAI BcoI Eco64I BbiII EheI Bst2UI BstH2I Tru1I
 BspEI BsiMI AvaI KasI Hsp92I HaeII HincII

 Bsh1285I MvaI BbiII
 DrdI BsaOI Bse1I BstOI BsaHI
 NspBII BstDSI BstMCI BsrSI EaeI EcoRII Msp17I
gctgtatcaggcagaccccacggtcgagctgacccagttgtttccggggctggccgccctgtgcctggacgccca base pairs
cgacatagtccgtctggggtgccagctcgactgggtcaacaaaggccccgaccggcgggacacggacctgcgggt 50401 to 50475
 MspA1I DsaI TthHB8I BseNI CfrI BstNI Hsp92I
 BsiEI BsrI Bst2UI AcyI
 TaqI AluI Hin1I

 EcoRII BsrSI BsrI TthHB8I AccB1I NarI HaeII Bst2UI
 BstOI Msp17I SalI HincII KasI BbiII EheI BstNI HaeII
 BstNI EaeI TspRI Hsp92I TaqI Eco64I BsaHI BbeI BstOI BstH2I
ggcggggcggccactggcgtcgacgaggcgcgtggtggatatgtcgtcgggcgcccgccaggcggcgctcgtgcg base pairs
ccgccccgccggtgaccgcagctgctccgcgcaccacctatacagcagcccgcgggcggtccgccgcgagcacgc 50476 to 50550
 MvaI CfrI Hin1I BsaHI HgaI BanI Msp17I Bsp143II Bsp143II
 Bst2UI BbiII Bse1I HindII BshNI Hsp92I EcoRII MvaI
HgaI BseNI AcyI AccI Hin1I AcyI BstH2I BglI BsiI

 BpmI BmyI SacI BsiHKAI
 GsuI AspHI FriOI Tru1I
 HphI Ecl136II Psp124BI MslI Tru9I
cctcaccgcgctggagctcatcaaccgcacccgcacaaacaccacccctgtgggggagattattaacgcccacga base pairs
ggagtggcgcgacctcgagtagttggcgtgggcgtgtttgtggtggggacaccccctctaataattgcgggtgct 50551 to 50625
 AluI Bsp1286I BanII BstXI MseI
BssSI EcoICRI Eco24I SstI
 SduI Bbv12I Alw21I

 SfaNI BstOI BsaHI
 StyI EcoRII BbiII
 Eco130I EcoO109I BbvI BglI SfaNI Hin1I
tgccttggggatacaatacgaacaggggcctgggctgctcgcccagcaggcacgcatcggcttggcgtcaaacac base pairs
acggaacccctatgttatgcttgtccccggacccgacgagcgggtcgtccgtgcgtagccgaaccgcagtttgtg 50626 to 50700
 ErhI DraII MvaI Bst71I Msp17I
 BssT1I BstNI Hsp92I
 EcoT14I Bst2UI AcyI HgaI

 BsoBI
 AfaI Eco88I
 HinfI MaeII MaeII BbvI Csp6I Ama87I
caagcgattcgccacgttcaacgtgggcagcgactacgacctgttgtactttttgtgtctcgggttcattcccca base pairs
gttcgctaagcggtgcaagttgcacccgtcgctgatgctggacaacatgaaaaacacagagcccaagtaaggggt 50701 to 50775
 TfiI XcmI Bst71I RsaI BcoI BsmAI
 AvaI
 Alw26I

 Bse1I StyI MaeI Eco47I
 BseNI DsaI Eco130I HgiEI
 Csp6I AfaI AvrII EcoNI SinI EcoRII
gtacctgtccgtggcctagggaagggtgggggtggtggtggtggggtgtttttctgttgttgtttctggtccgcc base pairs
catggacaggcaccggatcccttcccacccccaccaccaccaccccacaaaaagacaacaacaaagaccaggcgg 50776 to 50850
 BsrSI BstDSI BssT1I Bme18I
 RsaI AlwNI ErhI EcoT14I AvaII
 BsrI BlnI BfaI

MvaI AccB1I NarI HaeII MaeII
BstOI KasI BbiII EheI BbiII AatII
 MaeIII Eco64I BsaHI BbeI BsmFI Hin1I AcyI
tggtcacaaaaggcacggcgccccgaaacgcgggctttagtcccggcccggacgtcggcggacacgcaacaacgg base pairs
accagtgttttccgtgccgcggggctttgcgcccgaaatcagggccgggcctgcagccgcctgtgcgttgttgcc 50851 to 50925
BstNI BanI Msp17I Bsp143II Msp17I
Bst2UI BshNI Hsp92I Hsp92I
 Tsp45I Hin1I AcyI BstH2I BsaHI

 SduI BanII
 EcoO109I ApaI
 Bsp120I FriOI SfaNI
cgggccccgtgggtgggtaagttggttcgggggcatcgctgtattcccttgcccgcttccacccccccccccctt base pairs
gcccggggcacccacccattcaaccaagcccccgtagcgacataagggaacgggcgaaggtggggggggggggaa 50926 to 51000
 PspOMI BmyI BstDSI
 DraII Eco24I
 Bsp1286I DsaI

 SduI BssT1I Hsp92II HgaI KasI BbiII EheI
 BshNI BmyI NcoI NlaIII BsaHI BstD102I Hin1I AcyI
 Eco64I ErhI EcoT14I BbiII AccBSI AccB1I NarI HaeII
cccgttttgtttgtttgtgcgggtgcccatggcgtcggcggaaatgcgcgagcggttggaggcgcctctgcccga base pairs
gggcaaaacaaacaaacacgcccacgggtaccgcagccgcctttacgcgctcgccaacctccgcggagacgggct 51001 to 51075
 BanI Eco130I DsaI Hin1I AcyI BsrBI Eco64I BsaHI BstH2I
 AccB1I StyI BstDSI Msp17I BanI Msp17I Bsp143II
 Bsp1286I Bsp19I Hsp92I BshNI Hsp92I BbeI

 Bsh1285I Bsp1286I EaeI PpuMI Psp5II Eco88I BstNI
 BsiEI BanI SduI BsaAI AfaI SinI AvaII PspAI BsmFI
 BsaOI AccB1I MaeII Csp6I NspBII EcoO109I Cfr9I PspALI
ccgggcggtgcccatctacgtggccgggtttttggccctgtacgacagcggggacccgggcgagctggccctgga base pairs
ggcccgccacgggtagatgcaccggcccaaaaaccgggacatgctgtcgcccctgggcccgctcgaccgggacct 51076 to 51150
 Eco64I MslI RsaI MspA1I DraII BcoI XmaI AluI
 BshNI BstXI Bme18I Ama87I BsoBI EcoRII
 BstMCI BmyI CfrI HgiEI Eco47I AvaI SmaI

 Bme18I BsaWI Bsp143I
BstOI Eco47I MroI Bsp13I MboI DpnI
 SinI BspEI BsiMI DpnII MaeII
cccagacacggtgcgtgcggccctgcctccggagaaccccctgccgatcaacgtagaccaccgcgctcggtgcga base pairs
gggtctgtgccacgcacgccgggacggaggcctcttgggggacggctagttgcatctggtggcgcgagccacgct 51151 to 51225
 MvaI AvaII AccIII NdeII AccI
 HgiEI Kpn2I Sau3AI
Bst2UI BseAI Kzo9I

 Bbv12I HindII BsoBI Bsp143I MspA1I
 AspHI DsaI Eco88I MboI DpnI BsgI
 SduI BsiHKAI Ama87I DpnII PvuII
ggtgggccgggtgctcgccgtggtcaacgaccctcgggggccgttttttgtggggctgatcgcgtgcgtgcagct base pairs
ccacccggcccacgagcggcaccagttgctgggagcccccggcaaaaaacaccccgactagcgcacgcacgtcga 51226 to 51300
 Bsp1286I HincII BcoI NdeII AluI
 BmyI BstDSI AvaI Sau3AI NspBII
 Alw21I Kzo9I BbvI

 BcoI BsoBI CfrI Eco52I BsaOI DsaI MspA1I Bme18I Cfr9I SmaI
 BpmI Ama87I TthHB8I EclXI Bsh1285I BstH2I Sfr303I AvaII Eco88I
 GsuI HgaI AvaI BstZI XmaIII BstMCI Bsp143II Cfr42I Ama87I BsoBI
ggagcgcgtcctcgagacggccgccagcgccgctatttttgagcgccgcggacccgcgctctcccgggaggagcg base pairs
cctcgcgcaggagctctgccggcggtcgcggcgataaaaactcgcggcgcctgggcgcgagagggccctcctcgc 51301 to 51375
 Bst71I Sfr274I EaeI BsmAI Esp3I HaeII HaeII KspI SinI BcoI AvaI BseRI
 XhoI PaeR7I Alw26I BsiEI BstH2I BstDSI SstII HgiEI PspAI PspALI
 Eco88I TaqI EagI BsmBI Bsp143II NspBII SacII Eco47I XmaI

 DpnII DpnI
 AfaI Ksp22I BsaI
 HgaI Csp6I NdeII HphI BspMI Alw26I BsmFI
tctgctgtacctgatcaccaactacctgccatcggtctcgctgtccacaaaacgccggggggacgaggttccgcc base pairs
agacgacatggactagtggttgatggacggtagccagagcgacaggtgttttgcggcccccctgctccaaggcgg 51376 to 51450
 RsaI MboI Kzo9I Eco31I
 FbaI Sau3AI BsmAI
 BclI Bsp143I

 Eco72I AccB1I NarI Eco130I Tsp45I
 BstMCI Acc16I BbrPI KasI BbiII EheI StyI HphI
 BsiEI FspI PmlI Eco64I BsaHI BstH2I EcoT14I
cgaccgcaccctgtttgcgcacgtggccctgtgcgccatcgggcggcgccttggaaccatcgtcacctacgacac base pairs
gctggcgtgggacaaacgcgtgcaccgggacacgcggtagcccgccgcggaaccttggtagcagtggatgctgtg 51451 to 51525
 Bsh1285I AviII PmaCI BanI Msp17I Bsp143II MaeIII
 BsaOI MaeII BshNI Hsp92I ErhI BssT1I
 BsaAI Hin1I AcyI HaeII BbeI

 MvaI HgiEI
 CfrI BstOI Eco47I
 MaeI EaeI EcoRII Bme18I HgaI HgaI
cagcctagacgcggccatcgctccgtttcgccacctggacccggcgacgcgcgagggggtgcgacgcgaggccgc base pairs
gtcggatctgcgccggtagcgaggcaaagcggtggacctgggccgctgcgcgctcccccacgctgcgctccggcg 51526 to 51600
 BfaI HgaI BstNI AvaII
 Bst2UI
 SinI

 Bsp1286I BanII CfrI MvaI SduI Eco24I
 EcoICRI Eco24I BsiHKAI Bsp120I BmyI BstH2I
 SfiI Ecl136II SacI Alw21I BstNI EcoO109I ApaI Bsp143II BbvI
cgaggccgagctcgcgctggccgggcgcacctgggcccccggcgtggaggcgctcacacacacgctgctctccac base pairs
gctccggctcgagcgcgaccggcccgcgtggacccgggggccgcacctccgcgagtgtgtgtgcgacgagaggtg 51601 to 51675
 BglI AluI BmyI FriOI EaeI BstOI DraII FriOI HaeII Bst71I
 SduI Bbv12I SstI EcoRII PspOMI BanII
 AspHI Psp124BI Bst2UI Bsp1286I

 MaeIII Bsp143I
 SfaNI GsuI BstD102I MboI DpnI
 HincII NlaIII BbvI NspBII EaeI AccBSI DpnII AclWI
cgccgtcaacaacatgatgctgcgtgaccgctggagccttgtggccgagcggcggcggcaggccgggatcgccgg base pairs
gcggcagttgttgtactacgacgcactggcgacctcggaacaccggctcgccgccgccgtccggccctagcggcc 51676 to 51750
 HindII Hsp92II MspA1I CfrI BsrBI NdeII AlwI
 Tsp45I BpmI Sau3AI
 Bst71I Kzo9I

 RsaI Tsp509I
 BsaAI TspEI
 AflIII Eco57I Sse9I DraI HinfI
acacacgtaccttcaggcgagcgaaaaatttaaaatatggggggcggagtctgcccctgcgccggagcgcgggta base pairs
tgtgtgcatggaagtccgctcgctttttaaattttataccccccgcctcagacggggacgcggcctcgcgcccat 51751 to 51825
 MaeII AcsI MseI PleI
 Csp6I ApoI Tru1I
 AfaI Tru9I

 Eco64I BbiII Bsp143II AvaI Eco64I BssT1I Hsp92II
 Bse118I Hin1I NarI BbeI Cfr9I SmaI Eco130I NlaIII
 BssAI KasI Hsp92I HaeII PspAI PspALI StyI DsaI BstF5I BspMI
taaaaccggcgccccgggtgccatggacacatcccccgccgcgagcgttcccgcgccgcaggtcgccgtccgtgc base pairs
attttggccgcggggcccacggtacctgtgtagggggcggcgctcgcaagggcgcggcgtccagcggcaggcacg 51826 to 51900
 BsrFI BshNI BsaHI BstH2I Eco88I BshNI NcoI Bsp19I
 Cfr10I AccB1I AcyI Ama87I BsoBI AccB1I EcoT14I FokI
 BanI Msp17I EheI BcoI XmaI BanI ErhI BstDSI

 MboII BssAI CfrI Bsh1285I BshNI
 BbsI BshNI Cfr10I Eco52I BanI
 HgaI HgaI BpuAI MboII Eco64I EaeI XmaIII BsaOI SfaNI
gcgtcaagtcgcgtcgtcgtcttcttcttcttcttttccggcaccggccgatatgaaccccgtttcggcatcggg base pairs
cgcagttcagcgcagcagcagaagaagaagaagaaaaggccgtggccggctatacttggggcaaagccgtagccc 51901 to 51975
 Bbv16II BanI Bse118I EclXI Eco64I
 BpiI AccB1I BstZI BsiEI KasI
 BsrFI EagI BstMCI AccB1I

 Hsp92I BbeI MboI Bsp143I
 Msp17I Bsp143II BstX2I XhoII MaeIII
 Hin1I AcyI BstYI BamHI AlwI AluI
cgccccggcccctccgccgcccggcgacgggagttatttgtggatccccgcctctcattacaatcagctcgtcac base pairs
gcggggccggggaggcggcgggccgctgccctcaataaacacctaggggcggagagtaatgttagtcgagcagtg 51976 to 52050
 BsaHI BstH2I DpnII MflI DpnI Tsp45I
 BbiII EheI NdeII BstI AclWI
 NarI HaeII Sau3AI Kzo9I

 NgoAIV EagI BsiEI BsaOI Cfr42I
 NgoMI BstZI Eco52I NspBII SacII
 HphI NspBII MroNI Cfr10I Bsh1285I KspI BsmFI
cgggcaatccgcgccccgccacccgccgctgaccgcgtgcggcctgccggccgcggggacggtggcctacggaca base pairs
gcccgttaggcgcggggcggtgggcggcgactggcgcacgccggacggccggcgcccctgccaccggatgcctgt 52051 to 52125
 MspA1I BssAI EaeI EclXI BstMCI SstII
 BsrFI CfrI NaeI DsaI MspA1I
 Bse118I XmaIII BstDSI Sfr303I

 AccB1I NarI BssAI Bsp143II BcoI XmaI SduI
 KasI BbiII EheI Bse118I NaeI AfaI AvaI SmaI EcoO1
 Eco64I BsaHI BsrFI BstH2I BseRI Csp6I Cfr9I Bsp12
ccccggcgccggcccgtccccgcactacccgcctcctcccgcccacccgtacccgggtatgctgttcgcgggccc base pairs
ggggccgcggccgggcaggggcgtgatgggcggaggagggcgggtgggcatgggcccatacgacaagcgcccggg 52126 to 52200
 BanI Msp17I MroNI Cfr10I RsaI Eco88I PspOM
 BshNI Hsp92I NgoAIV BbeI Ama87I PspALI DraII
 Hin1I AcyI NgoMI HaeII BsmFI PspAI BsoBI Bsp1286I

 BanII BsmFI MvaI MboI EcoRII
 09I ApaI EcoRII GsuI Bsp143I BstMCI MvaI
0I FriOI Bse1I Bst2UI Sau3AI BsiEI BstOI
cagtcccctggaggcccagatcgccgcgctggtgggggccatcgccgccgaccgccaggcgggtgggcttccggc base pairs
gtcaggggacctccgggtctagcggcgcgaccacccccggtagcggcggctggcggtccgcccacccgaaggccg 52201 to 52275
I BmyI BsrSI BstXI BpmI Kzo9I Bsh1285I BstXI
 Eco24I BsrI BstNI DpnII DpnI BsaOI Bst2UI
 BseNI BstOI NdeII BstNI

EagI Eco52I BstDSI NdeII MflI AclWI
CfrI EclXI BsaOI BsaI Sau3AI Kzo9I BstH2I DraIII RsaI
EaeI XmaIII Alw26I BstX2I BstI AlwI Bsp143II BsiI BbvI Csp6I
ggccgccggagaccacgggatccgggggtcggcgaagcgccgccgacacgaggtggagcagccggagtacgactg base pairs
ccggcggcctctggtgccctaggcccccagccgcttcgcggcggctgtgctccacctcgtcggcctcatgctgac 52276 to 52350
BstZI Bsh1285I Eco31I MboI XhoII HaeII BssSI Bst71I
CciNI BstMCI BsmAI DpnII BamHI DpnI AfaI
NotI BsiEI DsaI BstYI Bsp143I

 XmaIII Tsp45I HgiEI Eco88I BsoBI Bsh1285I
 CfrI BsiEI MaeIII RsrII BsmFI PspAI PspALI Eco88I BsiEI
 EaeI Eco52I CpoI AvaII Ama87I Ama87I SalI BstMCI
cggccgtgacgagccggaccgggacttcccgtattacccgggcgaggcccgccccgagccgcgcccggtcgactc base pairs
gccggcactgctcggcctggccctgaagggcataatgggcccgctccgggcggggctcggcgcgggccagctgag 52351 to 52425
 BstZI BstMCI SinI Eco47I BcoI XmaI BcoI TthHB8I
 EagI Bsh1285I Bme18I Cfr9I SmaI AvaI AccI BsaOI
 EclXI BsaOI CspI AvaI BsoBI TaqI HincII

 PleI Bst2UI SduI BanII BbiII AcyI
HindII EcoRII EcoO109I ApaI HaeII Hin1I HphI
 AscI BssHII Bsp120I FriOI XcmI Tsp45I MaeII BstSFI
ccggcgcgccgcgcgccaggcttccgggccccacgaaaccatcacggcgctggtgggggcggtgacgtccctgca base pairs
ggccgcgcggcgcgcggtccgaaggcccggggtgctttggtagtgccgcgaccacccccgccactgcagggacgt 52426 to 52500
 HinfI BsePI BstNI PspOMI BmyI Bsp143II MaeIII BsaHI BsmFI
 BssHII BstOI DraII Eco24I BstH2I Msp17I AatII
 BsePI MvaI Bsp1286I Hsp92I SfcI

 PstI BsrI NspI Cfr10I EcoO109I ApaI
 EcoNI BseNI Hsp92II AfaI BsrFI PspOMI Eco24I
 AlwNI NlaIII Csp6I SgrAI BglI SduI BanII
gcaggaactggcgcacatgcgcgcgcgtacccacgccccctacgggccgtatccgccggtggggccctaccacca base pairs
cgtccttgaccgcgtgtacgcgcgcgcatgggtgcgggggatgcccggcataggcggccaccccgggatggtggt 52501 to 52575
 BsrSI BssHII RsaI BssAI Bsp120I FriOI
 Bst71I Bse1I BsePI Bse118I Bsp1286I
 BbvI DraII BmyI

 EcoT14I
 Eco31I StyI
 Alw26I Eco130I
cccccacgcagacacggagacccccgcccaaccaccccgctaccccgccaaggccgtctatctgccgccgccgca base pairs
gggggtgcgtctgtgcctctgggggcgggttggtggggcgatggggcggttccggcagatagacggcggcggcgt 52576 to 52650
 BsmAI ErhI
 BsaI BssT1I


 Eco88I DraII Eco47I Eco91I Eco47I
 PspAI PspALI HgiEI Bse1I MaeIII HgiEI
 Ama87I EcoO109I SinI BsmFI BsrSI EcoO65I SinI
catcgcccccccggggcctcctctatccggggcggtccccccaccctcgtatcccccagttgcggttacccccgg base pairs
gtagcgggggggccccggaggagataggccccgccaggggggtgggagcatagggggtcaacgccaatgggggcc 52651 to 52725
 BcoI XmaI BseRI Bme18I BseNI PspEI Bme18I
 Cfr9I SmaI AvaII BsrI BstPI AvaII
 AvaI BsoBI BstEII

 EcoO109I
 BsrBI PpuMI Psp5II
 BsmFI SinI AvaII
tcccgctcccccgctacatcagccctcccccgcacacgcccacccccctccgccgccgccgggacccacgcctcc base pairs
agggcgagggggcgatgtagtcgggagggggcgtgtgcgggtggggggaggcggcggcggccctgggtgcggagg 52726 to 52800
 AccBSI Bme18I
 BstD102I HgiEI Eco47I
 DraII BsmFI

 BsoBI BshNI BsaHI BstH2I NgoAIV KasI Hsp92I HaeII MseI
 Eco88I Hin1I NarI HaeII BsrFI BanI Msp17I EheI BstDEI
 AluI Ama87I AccB1I AcyI BbeI NgoMI Eco64I BbiII Bsp143II
ccccgccgcgagcttaccccaacccgaggcgcccggcgcggaggccggcgccttagttaacgccagcagcgcggc base pairs
ggggcggcgctcgaatggggttgggctccgcgggccgcgcctccggccgcggaatcaattgcggtcgtcgcgccg 52801 to 52875
 BcoI Eco64I Hsp92I MroNI Cfr10I AccB1I AcyI DdeI Tru1I
 AvaI BanI Msp17I Bsp143II Bse118I Hin1I NarI BbeI Tru9I
 KasI BbiII EheI BssAI NaeI BshNI BsaHI BstH2I HpaI

 BbvI PmlI Eco88I NdeII EcoO109I
 HindII PmaCI PspAI PspALI Kzo9I PpuMI Psp5II
HincII BsaAI Ama87I SmaI Bsp143I Tsp45I SinI AvaII EcoRII
ccacgtgaacgtggacacggcccgggccgccgatctgtttgtgtcacagatgatggggtcccgctaactcgcctc base pairs
ggtgcacttgcacctgtgccgggcccggcggctagacaaacacagtgtctactaccccagggcgattgagcggag 52876 to 52950
 Eco72I BcoI XmaI DpnII DpnI MaeIII Bme18I
 Bst71I MaeII Cfr9I SrfI MboI HgiEI Eco47I
 MaeII BbrPI AvaI BsoBI Sau3AI DraII BsmFI

 MvaI BstX2I GsuI AccIII BsiMI
 BstOI MboI MflI BpmI Kpn2I AlwI Tru1I
 DpnII BamHI DpnI BsaWI Tru9I BsaWI AccI
caggatccggacttggggggggtgtgtgttttcatatattttaaataaacaaacaaccggacaaaagtataccca base pairs
gtcctaggcctgaaccccccccacacacaaaagtatataaaatttatttgtttgttggcctgttttcatatgggt 52951 to 53025
 BstNI NdeII BstI BspEI Bsp13I MseI Bst1107I
 Bst2UI Sau3AI XhoII MroI AclWI DraI
 BstYI Bsp143I Kzo9I BseAI


 Tsp5
 DraIII Sse9I
cttcgtgtgcttgtgtttttgtttgagaggggggggtggagtgggggggaaagtgggccgaatgacacaaaaatt base pairs
gaagcacacgaacacaaaaacaaactctccccccccacctcaccccccctttcacccggcttactgtgtttttaa 53026 to 53100
 TspEI


 BpiI
09I Bbv16II
 HphI MaeI MboII
aggtcggaggggtgaggggggggggctaggagccgaaccgatggcccccacacgcgacggaaggcccggaagact base pairs
tccagcctccccactccccccccccgatcctcggcttggctaccgggggtgtgcgctgccttccgggccttctga 53101 to 53175
 BfaI BpuAI
 BbsI


 BsrFI BsaOI
 BssAI Bsh1285I BsmBI
 DsaI AgeI Cfr10I Alw26I
accacggggagggggtgtggaaagcgaccggtcgcagggagacggggttggtttggggttggtttggggttggtt base pairs
tggtgcccctcccccacacctttcgctggccagcgtccctctgccccaaccaaaccccaaccaaaccccaaccaa 53176 to 53250
 PinAI BsiEI BsmAI
 BsaWI BstMCI Esp3I
 BstDSI Bse118I

 NspI EcoT14I
 NlaIII Tsp45I StyI
 BspLU11I MaeI Eco130I
ttcccgttagcacatgtctgcatttgtttttctagtcacacgcccccccccccccaaataaaaaccaaggcaaaa base pairs
aagggcaatcgtgtacagacgtaaacaaaaagatcagtgtgcgggggggggggggtttatttttggttccgtttt 53251 to 53325
 AflIII BfaI ErhI
 Hsp92II MaeIII BssT1I


 NlaIII AluI
caataccagaagtcatgtgtatttttgaacatcggtgtctttttatttatacacaagcccagctcccctcccctc base pairs
gttatggtcttcagtacacataaaaacttgtagccacagaaaaataaatatgtgttcgggtcgaggggaggggag 53326 to 53400
 Hsp92II


 Bsp1286I BanII Bbv16II RsaI MvaI
 AluI BmyI FriOI BpuAI Alw26I BstD102I BstOI Acc16I
 DdeI Ecl136II SacI Alw21I MboII AccBSI BstNI FspI
ccttagagctcgtcttcgtctccggcctcgtcctcgttgtggagcggagagtacctggctttgttgcgcttgcgc base pairs
ggaatctcgagcagaagcagaggccggagcaggagcaacacctcgcctctcatggaccgaaacaacgcgaacgcg 53401 to 53475
 BstDEI SduI Bbv12I SstI BpiI Esp3I BsrBI EcoRII AviII
 EcoICRI Eco24I BsiHKAI BsmBI Csp6I Bst2UI
 AspHI Psp124BI BbsI BsmAI AfaI

 BstEII Eco130I DdeI BstH2I Bsp1286I NcoI
 MslI EcoO65I StyI CelII HaeII SduI StyI
 NlaIII PspEI ErhI Bpu1102I Bsp143II Eco13
agaaccatgttggtgaccttggagctgagcagggcgctcgtgcccttctttctggccttgtgttccgtgcgctcc base pairs
tcttggtacaaccactggaacctcgactcgtcccgcgagcacgggaagaaagaccggaacacaaggcacgcgagg 53476 to 53550
 Hsp92II Tsp45I BssT1I Bsp1720I BcgI BmyI ErhI
 Eco91I HphI EcoT14I BstDEI BsiI BssT1I
 BstPI MaeIII AluI BlpI BssSI EcoT14I

 Bsp19I Bsp143I BcoI
 DsaI Hsp92II MboI DpnI AvaI
 0I CfrI DpnII AclWI EaeI SfiI
atggccgacaccaaagccatatatcggatcatttctcgggcctcggccaacttggcctcgtcaaacccgcccccc base pairs
taccggctgtggtttcggtatatagcctagtaaagagcccggagccggttgaaccggagcagtttgggcgggggg 53551 to 53625
 BstDSI NdeII AlwI BsoBI CfrI BglI
 EaeI Sau3AI Ama87I
 NlaIII Kzo9I Eco88I

 Eco88I EcoT14I
 PspAI PspALI StyI BsrBI
 Ama87I Eco130I HphI
tccgcgccttcctccccctccccgcccacgcccccggggtcggaagtcttgagttccttggtggtgagcggatac base pairs
aggcgcggaaggagggggaggggcgggtgcgggggccccagccttcagaactcaaggaaccaccactcgcctatg 53626 to 53700
 BcoI XmaI ErhI AccBSI
 Cfr9I SmaI BssT1I BstD1
 AvaI BsoBI

 MvaI NgoMI CfrI
 DraII BstOI NgoAIV
 EcoO109I NlaIII MaeII MboII EcoRII BssAI EaeI
agggccttcatgggattgcgttgcagttgcaggacgtagcggaaggcgaagaaggccgcgaccaggccggccagg base pairs
tcccggaagtaccctaacgcaacgtcaacgtcctgcatcgccttccgcttcttccggcgctggtccggccggtcc 53701 to 53775
 Hsp92II BstNI BsrFI
 02I Bst2UI Bse118I
 MroNI Cfr1

 BstOI Eco47I BsoBI EagI Eco52I
 FseI SinI BbvI DsaI BstH2I Eco88I CfrI EclXI Bsh1285I
 BstNI Bme18I Bsp143II Ama87I EaeI XmaIII BstMCI
accagcagccccacggcaagcgccccgaaggggttggacataaaggaggacacgcccgagacggccgacaccacg base pairs
tggtcgtcggggtgccgttcgcggggcttccccaacctgtatttcctcctgtgcgggctctgccggctgtggtgc 53776 to 53850
NaeI MvaI HgiEI BstDSI HaeII BcoI BstZI BsmBI BsaOI
 EcoRII AvaII AvaI Alw26I BsiEI
 0I Bst2UI Bst71I BsmAI Esp3I

 BssHII
 BstMCI TthHB8I
 NlaIII BsiEI BsmFI BstF5I MboII
ccccccactactcccatgactaccttgccgaccgcgcgccccaagtcccccatcccctcgaagaacgcgcacagc base pairs
ggggggtgatgagggtactgatggaacggctggcgcgcggggttcagggggtaggggagcttcttgcgcgtgtcg 53851 to 53925
 Hsp92II Bsh1285I FokI
 BsaOI TaqI
 BsePI

 BsaHI AtsI MspA1I
 BbiII Tth111I BsgI
 NlaIII Hin1I BstF5I TthHB8I BspMI PvuII
cccgcgaacatggcggcgttggcgtcggcgcggatgaccgtgtcgatgtcggcaaagcgcaggtcgtgcagctgg base pairs
gggcgcttgtaccgccgcaaccgcagccgcgcctactggcacagctacagccgtttcgcgtccagcacgtcgacc 53926 to 54000
 Hsp92II Msp17I FokI TaqI AluI
 Hsp92I AspI NspBII
 AcyI HgaI BbvI

 Bme18I Bsp143I MvaI
 BstH2I Eco47I MboI DpnI BstOI
 Bsp143II NspBII DpnII BsiI AccI EcoRII BpmI
ttgcggcgctggacctccgtgtagtccagcaggccgctgtccttgatctcgtggcgcgtgtagacctccaggggc base pairs
aacgccgcgacctggaggcacatcaggtcgtccggcgacaggaactagagcaccgcgcacatctggaggtccccg 54001 to 54075
 Bst71I HaeII AvaII MspA1I NdeII BssSI BstNI
 SinI Sau3AI Bst2UI
 HgiEI Kzo9I GsuI

 HgiEI BbiII AcyI
 Bsp1286I Bme18I BpmI HphI Hin1I HphI BstDEI
 BsiI Eco47I NlaIII TthHB8I BcgI Tsp45I MaeII HinfI
acaaactcgtggtcctccagcatggtgatgttcaggtcgatgaaggtgctgacggtggtgacgtcggcgcgactc base pairs
tgtttgagcaccaggaggtcgtaccactacaagtccagctacttccacgactgccaccactgcagccgcgctgag 54076 to 54150
 SduI SinI GsuI MslI TaqI MaeIII BsaHI DdeI
 BmyI BssSI Hsp92II Msp17I AatII
 AvaII Hsp92I

 AluI MluI AfaI Cfr10I
 RsaI RsaI BseRI AflIII BsrFI BstDSI
 PvuII HphI AflIII TthHB8I MaeII MboII SgrAI HaeII
agctggtgagagtacgcgtactcctcgaagtacacgtagcccccgccgaagatgaagtagcgccggtggcccacg base pairs
tcgaccactctcatgcgcatgaggagcttcatgtgcatcgggggcggcttctacttcatcgcggccaccgggtgc 54151 to 54225
 MspA1I Csp6I Csp6I TaqI Csp6I BssAI BstH2I
PleI AfaI AfaI RsaI Bse118I DsaI
 NspBII BsaAI Bsp143II

 Bsp1286I BcoI TaqI BbvI MboI Kzo9I EcoO1
 SduI BsiHKAI AvaI Bst71I NspBII DpnII DpnI PspOMI
 Alw44I Alw21I Eco88I HgaI HphI AluI PvuII TthHB8I MspA1I
gtgcacggctcgagcgcgtcgcgggtgaggcgcagctcgttgttctcgcccagctgcccctcgatcagcgggccc base pairs
cacgtgccgagctcgcgcagcgcccactccgcgtcgagcaacaagagcgggtcgacggggagctagtcgcccggg 54226 to 54300
 VneI BmyI Sfr274I BsoBI BbvI AluI TaqI Bsp143I DraII
 ApaLI Bbv12I XhoI TthHB8I MspA1I NdeII NspBII
 AspHI Ama87I PaeR7I Bst71I Sau3AI Bsp12

 09I ApaI MvaI BpiI EcoRII EagI Bsh1285I
 Eco24I Bst2UI Csp6I Bst2UI BstZI BstMCI TaqI
 SduI FriOI BstOI MboII BstNI SfcI MaeII BsmFI EclXI AluI HgaI SfaNI
tggtcttcgtaccgaaagctgaccagggggcggctgtagcacgtccccggccgcgagctgacgcgcatcgagttc base pairs
accagaagcatggctttcgactggtcccccgccgacatcgtgcaggggccggcgctcgactgcgcgtagctcaag 54301 to 54375
 BmyI BstNI BbsI AfaI MvaI BstSFI EaeI Eco52I TthHB8I
EcoRII BanII BpuAI RsaI BstOI CfrI BsiEI
 0I Bsp1286I Bbv16II AluI XmaIII BsaOI

 Bsp143I MaeII Alw26I BbiII AcyI BsmBI BsePI
 MboI Kzo9I BmyI BsaAI CfrI Hsp92II AatII NlaIII SphI
 DpnII MaeII SduI Eco72I EaeI NlaIII MaeII Esp3I BbuI
tgcacgatcacgttgtccggggcgacgggcacgcacgtggagacggccatgacgtctccgagcatgcgcgcgctc base pairs
acgtgctagtgcaacaggccccgctgcccgtgcgtgcacctctgccggtactgcagaggctcgtacgcgcgcgag 54376 to 54450
 NdeII Bsp1286I BbrPI Esp3I Hin1I BsaHI PaeI NspI
 Sau3AI MslI PmaCI BsmBI Msp17I Alw26I BssHII
 BsgI DpnI PmlI BsmAI Hsp92I BsmAI Hsp92II

 BsrFI CfrI NaeI BstMCI
 BssAI EaeI EclXI BsaOI
 HphI Bse118I XmaIII AluI HinfI AluI
acccgccggccgacggtggcggaggcgatggcgttggggttgagcttgcgggcctcgttccagagagtcagctcg base pairs
tgggcggccggctgccaccgcctccgctaccgcaaccccaactcgaacgcccggagcaaggtctctcagtcgagc 54451 to 54525
 MroNI Cfr10I Bsh1285I PleI
 NgoMI BstZI Eco52I
 NgoAIV EagI BsiEI

 Bst71I PmlI
 SfcI BbvI NspI MslI BbrPI
 BsiI PstI SfaNI NlaIII MaeII Bsp143II MaeII
tggttctgcagctcgcaccacgcgacggcgatgcgccccagcatgtcattcacgtggcgctgtatgtggttatac base pairs
accaagacgtcgagcgtggtgcgctgccgctacgcggggtcgtacagtaagtgcaccgcgacatacaccaatatg 54526 to 54600
 BssSI AluI BcgI Hsp92II Eco72I HaeII
 BstSFI BsaAI BstH2I
 PmaCI

SnaBI MboI Kzo9I Bsh1285I BanI
BsaAI BstSFI DpnII DpnI Ple19I DsaI MluI BstH2I AccB1I
 SfcI BbvI TthHB8I TthHB8I BsaOI SfaNI AflIII Bsp143II
gtaaactgcagccgggcgaactcgatcgaggaggtggtcttgatgcgctccacggacgcgttggcgctgggcgcc base pairs
catttgacgtcggcccgcttgagctagctcctccaccagaactacgcgaggtgcctgcgcaaccgcgacccgcgg 54601 to 54675
Eco105I PstI TaqI Bsp143I TaqI BseRI BstDSI XcmI HaeII BshNI
BstSNI Bst71I NdeII BspCI PvuI HgaI Eco64I
 Sau3AI BsiEI BstMCI KasI

Hin1I AcyI TspRI Eco47I RsaI AfaI
 NarI BbeI BsaMI HgiEI Hsp92II Csp6I
 BbiII EheI BsmI SinI NlaIII MaeII AluI
tcccgcagtggcgcgggcgtggcattccggggcttgcggtcctgctcccgcatgtactcccgcacgtacagctcg base pairs
agggcgtcaccgcgcccgcaccgtaaggccccgaacgccaggacgagggcgtacatgagggcgtgcatgtcgagc 54676 to 54750
 BsaHI BstH2I Mva1269I Bme18I Csp6I BsaAI
 Hsp92I HaeII AvaII NspI RsaI
Msp17I Bsp143II AfaI

 MluNI Csp6I
 BstDEI AfaI EaeI BstNI RsaI
 DdeI BseRI Csp6I BstDSI EcoRII Acc113I
gcgagcgtgttgctgaggaggggctggtacgcgatgaggaagccccccgtggccaggtagtactgcggctggccc base pairs
cgctcgcacaacgactcctccccgaccatgcgctactccttcggggggcaccggtccatcatgacgccgaccggg 54751 to 54825
 BcgI RsaI DsaI MscI Bst2UI Eco255I
 CfrI BstOI AfaI
 BalI MvaI ScaI

 BsiEI BstZI Bsh1285I
 AfaI NspI TaqI FokI XmaIII
 MslI Csp6I BssHII NlaIII BstMCI BglI SfaNI EagI BstMCI
accttgatgtgcgtggcgttgtacttgcgcgcaaacatgcggtcgatggcctcgcgggcatcccggccgatgcag base pairs
tggaactacacgcaccgcaacatgaacgcgcgtttgtacgccagctaccggagcgcccgtagggccggctacgtc 54826 to 54900
 RsaI BsePI Hsp92II BsaOI BstF5I BsiEI
 TthHB8I EaeI EclXI
 Bsh1285I CfrI Eco52I

 MvaI AccI HgaI ScaI BsaHI
BsaOI BstOI HincII RsaI BbiII
 BstNI TaqI Csp6I HphI TthHB8I Hin1I
tcgcccaggtcgacgcgcgagagcgagtactcggtcaggttggtggtgaaggtggtcgagatggcgtcggaggag base pairs
agcgggtccagctgcgcgctctcgctcatgagccagtccaaccaccacttccaccagctctaccgcagcctcctc 54901 to 54975
 EcoRII TthHB8I Acc113I TaqI Msp17I
SfaNI Bst2UI HindII AfaI Hsp92I
 SalI Eco255I AcyI HgaI

 Cfr1
 AfaI BsgI AhdI
BseRI Csp6I SfaNI NlaIII BssAI
aagcggaaggagccgccgtactcggcgcggagcatctcgtccacctcctgccacttggtcatggtgcagaccgcc base pairs
ttcgccttcctcggcggcatgagccgcgcctcgtagagcaggtggaggacggtgaaccagtaccacgtctggcgg 54976 to 55050
 RsaI Hsp92II BsrFI
 Bse118I
 EclHKI

 0I Bsh1285I BsrI BstOI
 BsiEI BanI BseNI BsmFI
 Eam1105I AccB1I EcoRII BstDSI MaeII
ggtcgcttcggcacccagtcccaggccacggtaaacttgggggtcgtcagcaagttgcgggtcgtcggcgacgtg base pairs
ccagcgaagccgtgggtcagggtccggtgccatttgaacccccagcagtcgttcaacgcccagcagccgctgcac 55051 to 55125
 AspEI Eco64I BsrSI Bst2UI XcmI
 BstMCI Bse1I MvaI
 BsaOI BshNI BstNI DsaI

 Eco88I HincII
 PspAI PspALI AccI BstMCI
 Ama87I SmaI HphI BssHII SalI BspMI BsiEI AluI
gcccgggccttcgtggtgaggtcgcgcgcgtagaagccgtcgacctgcttgaagcggtcggcggcgtagctggtg base pairs
cgggcccggaagcaccactccagcgcgcgcatcttcggcagctggacgaacttcgccagccgccgcatcgaccac 55126 to 55200
 BcoI XmaI BsePI TthHB8I Bsh1285I
 Cfr9I SrfI TaqI BsaOI
 AvaI BsoBI HindII

 Bbv12I Bsp1407I AfaI
 AspHI BsmFI Csp6I
 SduI BsiHKAI BspLU11I Hsp92II
tgctcggtgtgcgacccctcccggtagccgtaaaacggggacatgtacacaaagtcgcccgtcgccagcacaaac base pairs
acgagccacacgctggggagggccatcggcattttgcccctgtacatgtgtttcagcgggcagcggtcgtgtttg 55201 to 55275
 Bsp1286I AflIII NlaIII
 BmyI SspBI NspI
 Alw21I BsrGI RsaI

 SunI AfaI AhdI
 Pfl23II RsaI BstMCI HgaI BseRI HindII AfaI
 SplI RsaI BsiEI Eam1105I TthHB8I SfaNI Csp6I
tcatcgtacgggtacaccgaccgcgcgtccacctcctcgacgatgcagttgaccgtcgtgccgtaccgatggaac base pairs
agtagcatgcccatgtggctggcgcgcaggtggaggagctgctacgtcaactggcagcacggcatggctaccttg 55276 to 55350
 PspLI Csp6I Bsh1285I TaqI HincII RsaI
 BsiWI AfaI BsaOI AspEI
 Csp6I EclHKI

 NgoAIV
 AfaI NgoMI
 Csp6I BsiI MroNI
gcctccacccgcgaggggttgtacttgaggtcggtggtgtgccacccccggctcgtgcgcgtggcgaccttcgcc base pairs
cggaggtgggcgctccccaacatgaactccagccaccacacggtgggggccgagcacgcgcaccgctggaagcgg 55351 to 55425
 RsaI BssSI BssAI
 BsrFI
 Bse118I

 EcoICRI Eco24I BsiHKAI BsiI NlaIII AfaI
 Ecl136II SacI SstI Eco31I BsaI Csp6I
 Cfr10I AspHI Psp124BI Alw26I BssSI HphI Alw26I MaeII EaeI
ggcttgagctccatgtcggtctcgtggtcgtcccggtgaaacgcggtggtctccatgttgttccgcacgtacttg base pairs
ccgaactcgaggtacagccagagcaccagcagggccactttgcgccaccagaggtacaacaaggcgtgcatgaac 55426 to 55500
 NaeI SduI Bbv12I Alw21I BsaI BsmFI Eco31I BsaAI CfrI
 AluI BmyI FriOI NlaIII BsmAI BsmAI RsaI
 Bsp1286I BanII Hsp92II Hsp92II

 BssT1I MboI Kzo9I Eco47I
 BstDSI BstD102I Tru1I DpnII DpnI TthHB8I HgiEI
 AccBSI Eco130I Tru9I TthHB8I HphI BseRI SinI TthHB8I
gccgtggagcggcagacccccttggtgttaatcttgtcgatcacctcctcgaagggaacgggggcgcggtcctcg base pairs
cggcacctcgccgtctgggggaaccacaattagaacagctagtggaggagcttcccttgcccccgcgccaggagc 55501 to 55575
 BsrBI ErhI MseI TaqI Bsp143I Bme18I
 DsaI StyI NdeII TaqI AvaII
 EcoT14I Sau3AI TaqI

 BsrSI MaeIII
 Esp1396I AspI MaeII AfaI
 AccB7I BsrI EaeI BspMI Tsp45I Csp6I NlaIII
aatatccccataaactgggagtagcggtggccgaaccacacctgcgacacggtcacgtctttgtagtacatggtg base pairs
ttataggggtatttgaccctcatcgccaccggcttggtgtggacgctgtgccagtgcagaaacatcatgtaccac 55576 to 55650
 PflMI Bse1I CfrI Tth111I RsaI Hsp92II
 Van91I AtsI
 BseNI

 Tsp509I BpiI
 TspEI AfaI Bbv16II
 Sse9I Csp6I MboII MslI SfaNI
gccttgaatttgtacggggcgatgttctccttgaagaccaccgcgatgccctccgtgtagttctgcccctccggg base pairs
cggaacttaaacatgccccgctacaagaggaacttctggtggcgctacgggaggcacatcaagacggggaggccc 55651 to 55725
 AcsI RsaI BpuAI
 ApoI BbsI


 KasI BbiII EheI AflIII
 BbvI BstDSI Hin1I AcyI Bsp1286I
 HgaI NspBII BbvI BsgI Eco64I BsaHI HaeII SduI
cgcgtcgggcagcggcgcggctgctcaaactgcaccaccgtggcgcccgtcgggggcgggcacacgtaaaactgg base pairs
gcgcagcccgtcgccgcgccgacgagtttgacgtggtggcaccgcgggcagcccccgcccgtgtgcattttgacc 55726 to 55800
 MspA1I Bst71I MslI BanI Msp17I Bsp143II MaeII
 Bst71I DsaI AccB1I NarI BstH2I BmyI
 BshNI Hsp92I BbeI BsaAI

 NgoMI
Bse1I BsePI MroNI Cfr10I BstMCI
BsrSI TthHB8I BspMI BbvI EaeI NgoAIV BsiEI
gcatcggcgttctcgaccttgatttcccgcaggtgcgcgcgcagcgtggcgtggccggcggcgacggtcgcgttg base pairs
cgtagccgcaagagctggaactaaagggcgtccacgcgcgcgtcgcaccgcaccggccgccgctgccagcgcaac 55801 to 55875
BseNI TaqI BssHII Bst71I CfrI Bse118I Bsh1285I
BsrI SfaNI BssAI NaeI BsaOI
 BsrFI

 Hsp92I BsoBI EcoT14I
 Msp17I Eco88I StyI
 Hin1I HgaI Ama87I BglI Eco130I
gcgtcggggggcggggtcgcctcgggccgcttgggcggctttttggttttccgcttccgggccttggtggtcgcg base pairs
cgcagccccccgccccagcggagcccggcgaacccgccgaaaaaccaaaaggcgaaggcccggaaccaccagcgc 55876 to 55950
 AcyI BcoI ErhI
 BbiII AvaI BssT1I
 BsaHI

 BsoBI Eco24I XmaIII HgiEI Eco47I EaeI BsoBI
 Eco88I BsmFI CfrI BsiEI PpuMI Psp5II BstMCI Eco88I
 Ama87I BanII EaeI Eco52I SinI AvaII BsiEI Ama87I
gggctcgggacggggggcggccgggaggcgggacccccgttcgccgcgacggtcgcggccacgccgcccgaggcg base pairs
cccgagccctgccccccgccggccctccgccctgggggcaagcggcgctgccagcgccggtgcggcgggctccgc 55951 to 56025
 BcoI Bsp1286I BstZI BstMCI Bme18I BsmFI Bsh1285I BcoI
 AvaI BmyI EagI Bsh1285I DraII BsaOI AvaI
 SduI FriOI EclXI BsaOI EcoO109I CfrI

 BsaHI MspA1I Cfr42I
 BbiII DsaI CfrI BstH2I
 SfiI Hin1I BstDSI KspI Bsp143II BssHII
cggggggccgccggggccgccggggccgccgacgccaccgcggccaccagcgcccccacgaccagcgcgcaaatc base pairs
gccccccggcggccccggcggccccggcggctgcggtggcgccggtggtcgcgggggtgctggtcgcgcgtttag 56026 to 56100
 BglI Msp17I BglI Sfr303I HaeII BsePI
 Hsp92I NspBII SstII
 AcyI HgaI EaeI SacII


 NlaIII HgaI MaeI SfcI
aagccccccccgcgcatggcgggcctacgggggcgcgtcgctcccgccgcccgctagtctgggggcgaggtgctg base pairs
ttcggggggggcgcgtaccgcccggatgcccccgcgcagcgagggcggcgggcgatcagacccccgctccacgac 56101 to 56175
 Hsp92II BfaI BstSFI


 PstI Eco47I BsiEI SinI Eco47I
 BbvI AvaII BsmBI BsaOI BstMCI BsrBI AvaII Bst71I
 SinI BstF5I MaeII Alw26I BsiEI AccBSI SfaNI BglI
caggaccgagtagaggatggaaaaaacgtctcggtcgtaaaccacgaccgagcggggtccgatgcagccgtcggg base pairs
gtcctggctcatctcctaccttttttgcagagccagcatttggtgctggctcgccccaggctacgtcggcagccc 56176 to 56250
 HgiEI FokI BsmAI BstMCI Bsh1285I HgiEI BbvI
 Bst71I Esp3I BsaOI BstD102I
 Bme18I Bsh1285I Bme18I

 TaqI BalI BsaAI
 BstD102I MscI AfaI AfaI AfaI
 AccBSI EaeI NspBII Csp6I Csp6I Csp6I
gccgctctcgacgatggccaccagcggacagtcggagttgtacgtgaggtacacgcccggcgggtagcggtacag base pairs
cggcgagagctgctaccggtggtcgcctgtcagcctcaacatgcactccatgtgcgggccgcccatcgccatgtc 56251 to 56325
 BsrBI CfrI MspA1I RsaI RsaI RsaI
 TthHB8I MluNI MaeII


 PstI BsrFI
 BbvI BssAI
 SfcI AluI AgeI Cfr10I HgaI
accttcggaggtcgggcggctgcagtcggggcggcgcaactcaagctccccgcaccggtagaccgacgcaaagag base pairs
tggaagcctccagcccgccgacgtcagccccgccgcgttgagttcgaggggcgtggccatctggctgcgtttctc 56326 to 56400
 BstSFI PinAI AccI
 Bst71I BsaWI
 Bse118I

 Bsp1286I BanII NruI Bst2UI MroI Bsp13I
 EcoICRI Eco24I BsiHKAI BssHII BspEI BsiMI
 Ecl136II SacI Alw21I BstOI HinfI BseAI
tgtggtggcgataatgagctcgcgaatatatcgccaggcggcgcgctgggtgggcgtgattccggaaacaccgtc base pairs
acaccaccgctattactcgagcgcttatatagcggtccgccgcgcgacccacccgcactaaggcctttgtggcag 56401 to 56475
 AluI BmyI FriOI Bsp68I MvaI TfiI BsaWI
 SduI Bbv12I SstI BstNI AccIII
 AspHI Psp124BI EcoRII BsePI Kpn2I


 BstH2I
 Bsp143II NlaIII
aaaacagtagaacttttgaaactcgctgacggcccaatcagcgcccgaaccccccgcgcccatgatgaagcgggc base pairs
ttttgtcatcttgaaaactttgagcgactgccgggttagtcgcgggcttggggggcgcgggtactacttcgcccg 56476 to 56550
 HaeII Hsp92II


 FriOI
 BmyI TaqI SfcI AfaI
 EcoNI SduI MaeII HgaI Csp6I
gagttcctccttgaggtgcggcaggagccccacgttctcgacgctgtagtacagcgcggtgttggggggctgggc base pairs
ctcaaggaggaactccacgccgtcctcggggtgcaagagctgcgacatcatgtcgcgccacaaccccccgacccg 56551 to 56625
 Bsp1286I TthHB8I RsaI
 Eco24I BstSFI
 BanII

 SduI ApaI EcoICRI Bbv12I BanII
 EcoO109I BanII SduI BmyI Psp124BI HphI
 AluI TthHB8I Bsp120I FriOI Ecl136II Eco24I Alw21I SfaNI
gaagctgtgggtggagtggtcgaacaggggcccgttgacgagctcgaagaagcgatgggtgatgctggggagcag base pairs
cttcgacacccacctcaccagcttgtccccgggcaactgctcgagcttcttcgctacccactacgacccctcgtc 56626 to 56700
 TaqI PspOMI Eco24I AluI Bsp1286I FriOI MboII
 DraII BmyI HincII TthHB8I SacI SstI
 Bsp1286I HindII AspHI TaqI BsiHKAI

 Eco47I MvaI PflMI BssAI Eco88I
 HgiEI BstOI Van91I AgeI Bse118I PspAI PspALI
 SinI SexAI AccB7I BbvI HgaI NlaIII BsrFI HgaI Ama87I
ggccgggtccacctggtggcgcagcagcgacgctcgcatgaaccggtgcgcgtcaaacacgcccggggcggcgcg base pairs
ccggcccaggtggaccaccgcgtcgtcgctgcgagcgtacttggccacgcgcagtttgtgcgggccccgccgcgc 56701 to 56775
 Bme18I BstNI Esp1396I Hsp92II Cfr10I BcoI XmaI
 AvaII Bst2UI Bst71I PinAI Cfr9I SmaI
 EcoRII DraIII BsaWI AvaI BsoBI

 BstMCI
 Bsp1286I BsePI BstDSI
 TthHB8I BcgI BglI BssHII BsiEI
gttgtcgatgaccgtgcccgcgcccgccgtcagggcgcagaagcgcgcgcgcgccgcgaagccgttggcgaccgc base pairs
caacagctactggcacgggcgcgggcggcagtcccgcgtcttcgcgcgcgcgcggcgcttcggcaaccgctggcg 56776 to 56850
 TaqI SduI BsePI BssHII Bsh1285I
 BmyI DsaI
 BsaOI

 SstII XhoI PaeR7I EcoICRI Psp124BI
 Sfr303I SfaNI AvaI Ecl136II Bbv12I
 MspA1I BbvI BstDSI HgaI BbvI Sfr274I TthHB8I AspHI FriOI
ggcgaaggtcgcgggcagcacctcgccgtggacgctgacccgcagcatcttctcgagctccccgcgctgctcgcg base pairs
ccgcttccagcgcccgtcgtggagcggcacctgcgactgggcgtcgtagaagagctcgaggggcgcgacgagcgc 56851 to 56925
 Cfr42I Bst71I DsaI Bst71I Eco88I TaqI SduI Eco24I
 NspBII Ama87I MboII Bsp1286I BanII
 KspI SacII BcoI BsoBI AluI BmyI SacI

 Bsp143II MscI BsaOI CspI AfaI BsrFI HaeII
 Alw21I Bst71I Eco88I BalI Bsh1285I Bme18I Csp6I BssAI NaeI
 BbvI Ama87I AvaI MluNI BsiEI CpoI AvaII SgrAI Bse118I MaeII
cacgcagcgcccgaggctggccagcgaccgcttggtcaggcggtccgcgtacagccgccggcgctcccgcacgtc base pairs
gtgcgtcgcgggctccgaccggtcgctggcgaaccagtccgccaggcgcatgtcggcggccgcgagggcgtgcag 56926 to 57000
 SstI BbvI BcoI EaeI XcmI SinI Eco47I MroNI Cfr10I
 Bst71I BstH2I CfrI BstMCI HgiEI RsaI NgoMI Bsp143II
 BsiHKAI HaeII BsoBI DrdI RsrII NgoAIV BstH2I

 KspI SacII MvaI Eco88I
DsaI SstII NruI BstOI BsoBI
 NspBII HgaI AluI EcoRII BcoI
cgcggcggcccgcgtcgcgatgtcgccccagctctccggcccctgcgcccctggctcggggccgcgctccccgtc base pairs
gcgccgccgggcgcagcgctacagcggggtcgagaggccggggacgcggggaccgagccccggcgcgaggggcag 57001 to 57075
BstDSI Cfr42I Bsp68I BstNI AvaI
 MspA1I Bst2UI
 Sfr303I Ama87I

 BsaHI MspA1I BcoI AvaI SduI BanII
 BbiII DsaI Sfr303I Cfr9I SmaI FriOI
 Hin1I BsmFI BseRI SstII PspAI PspALI Eam11
ctcgctcgcgggcgtccccgcgccacgcctccgccccccctcctccgcggcggcccggggctcttcctcctcggc base pairs
gagcgagcgcccgcaggggcgcggtgcggaggcgggggggaggaggcgccgccgggccccgagaaggaggagccg 57076 to 57150
 Msp17I BstDSI Cfr42I Eco88I BmyI SapI
 Hsp92I NspBII Ama87I BsoBI Eco24I EarI
 AcyI HgaI KspI SacII XmaI Bsp1286I Ksp632I

BseRI BsrFI BstH2I AvaI
MboII BstMCI BssAI NaeI Bsp143II BcoI
04I BsiEI MroNI Cfr10I BbvI BseRI TspRI
ccccccggtcgcgccgccggcccccagccgcgccagcacgcggcgcagcgcctcctcgtcgcactgctcggggct base pairs
ggggggccagcgcggcggccgggggtcggcgcggtcgtgcgccgcgtcgcggaggagcagcgtgacgagccccga 57151 to 57225
 Bsh1285I Bse118I Bst71I Ama87I
 BsaOI NgoMI HaeII Eco88I
 NgoAIV BsoBI

 Msp17I TaqI Sau3AI MboI
 NspBII BsaHI BstH2I MboI NdeII
 BbvI Hin1I HgaI Bsp143II Bsp143I
gacgagccgccgcagcagcggcgtcgtcaggtggtggtcgtagcacgcgcgtatcagcgcctcgatctgatcgtc base pairs
ctgctcggcggcgtcgtcgccgcagcagtccaccaccagcatcgtgcgcgcatagtcgcggagctagactagcag 57226 to 57300
 Bst71I Hsp92I HaeII NdeII DpnII
 MspA1I AcyI TthHB8I DpnI
 BbiII BcgI DpnII Kzo9I

 Msp17I EcoRII NdeII Msp17I BstNI Bsp143I
 Hin1I AcyI MvaI DpnII DpnI BbiII BstH2I MvaI MboI
 Kzo9I Hsp92I Bst2UI FbaI Sau3AI BsaHI NlaIII Bsp143II DpnII
gggcgacgtcgcctggccgccgatgatcagggcgtccaccatgtccagcgccgccaggtggcccccgaacgcgcg base pairs
cccgctgcagcggaccggcggctactagtcccgcaggtggtacaggtcgcggcggtccaccgggggcttgcgcgc 57301 to 57375
 DpnI MaeII BstNI CfrI Ksp22I Hin1I HgaI Hsp92II HaeII Bst2UI NdeII
Sau3AI BbiII AatII EaeI BclI Bsp143I AcyI EcoRII Sau3AI
Bsp143I BsaHI BstOI MboI Kzo9I Hsp92I BstOI Kzo9I

 Bsh1285I AspHI DsaI MspA1I Eco31I BbvI
 DpnI TaqI SduI BsiHKAI BstH2I CfrI BglI Cfr42I SfcI AluI
 BspCI PvuI Bsp1286I Bsp143II EaeI BstDSI SstII BsaI BbvI
atcgaagtgctccgcccgccgcccgaacagcgccagctccacggccaccgcggcggtctcctgctgcagctcgcg base pairs
tagcttcacgaggcgggcggcgggcttgtcgcggtcgaggtgccggtggcgccgccagaggacgacgtcgagcgc 57376 to 57450
 TthHB8I BsaOI Alw21I HaeII BstDSI DsaI Sfr303I BsmAI Bst71I
 Ple19I BcgI BmyI AluI NspBII Alw26I PstI
 BsiEI BstMCI Bbv12I KspI SacII BstSFI Bst71I

 BsaHI StyI DsaI Bsp143I Bsh1285I Hsp92I
 BbiII ErhI BstDSI MboI DpnI PvuI BbiII
 BbvI Hin1I HgaI EcoT14I DpnII BspCI BsaOI BsaHI
ctgcgccagcgcgttcaggttgtcggcgaaggcgtccatggtggagtggcgggcgcgatcgccggacgccagcca base pairs
gacgcggtcgcgcaagtccaacagccgcttccgcaggtaccacctcaccgcccgcgctagcggcctgcggtcggt 57451 to 57525
 Bst71I Msp17I BssT1I Hsp92II NdeII BsiEI Hin1I AcyI
 Hsp92I NcoI Bsp19I Sau3AI Ple19I Msp17I
 AcyI Eco130I NlaIII Kzo9I SgfI BstMCI HgaI

 BsaHI
 Bst71I AfaI BpmI BbiII
 AluI Csp6I AflIII NlaIII EcoNI Hin1I
gaagcgcagctcgctgatggcgtacaggccgggcgtagtggcctgaaacacgtcatgcgcctccagcagggcgtc base pairs
cttcgcgtcgagcgactaccgcatgtccggcccgcatcaccggactttgtgcagtacgcggaggtcgtcccgcag 57526 to 57600
 BbvI RsaI MaeII Hsp92II GsuI Msp17I
 Hsp92I
 AcyI

 EarI NgoAIV BstNI KspI SacII
 AluI NgoMI FseI MvaI NspBII CpoI
 BseRI MboII MroNI Cfr10I BstDSI Cfr42I
ggcctcctcgcggacagaagagctatcggcgggcggcgggccggccctggccccgccgcccgccgcggtccgcgc base pairs
ccggaggagcgcctgtcttctcgatagccgcccgccgcccggccgggaccggggcggcgggcggcgccaggcgcg 57601 to 57675
 Eam1104I BssAI NaeI Bst2UI DsaI SstII Bme18I
 Ksp632I BsrFI EcoRII MspA1I SinI
 HgaI SapI Bse118I BstOI Sfr303I HgiEI

 Eco47I BstH2I Bst2UI Eco47I BstH2I BsaHI BshNI
 RsrII HaeII Van91I Bme18I HaeII BbiII Asp718I
 CspI AlwNI Esp1396I AvaII Bsp143II Hin1I Eco64I
cagcgcctggtccagcacacagagcgctcgcgcgcgggcggcgtccgacagcccggcggcgtggggcaggtaccg base pairs
gtcgcggaccaggtcgtgtgtctcgcgagcgcgcgcccgccgcaggctgtcgggccgccgcaccccgtccatggc 57676 to 57750
 Bsp143II BstNI SinI Eco47III Msp17I BanI Csp6I
 AvaII AccB7I BstOI HgiEI AfeI BssHII Hsp92I Acc65I
 EcoRII PflMI MvaI Aor51HI BsePI AcyI HgaI AccB1I

RsaI BsaHI MvaI HgiEI BstOI
 Bst71I BbiII BstOI MaeII SinI Eco47I
 AfaI AluI Hin1I EcoRII Tsp45I MaeIII SfaNI RsrII Bst2UI
tcgcagctcgttggcgtccagccgcacctgggcctgttgggtgacgtggttacagatgcggtccgccaggcggcg base pairs
agcgtcgagcaaccgcaggtcggcgtggacccggacaacccactgcaccaatgtctacgccaggcggtccgccgc 57751 to 57825
 BbvI Msp17I BstNI MaeIII CpoI AvaII MvaI
 BspMI Hsp92I Bst2UI HphI Bme18I BstNI
 KpnI AcyI HgaI CspI EcoRII

 EcoT14I Bsp172
 StyI HphI BseRI CelII
 Eco130I Tsp45I HgaI TthHB8I BsmFI Bpu11
ggcgatggtcgccccttggttcgcggtgacgcacagctcctcgaaacagaccgcgcacgggtgggacgggtcgct base pairs
ccgctaccagcggggaaccaagcgccactgcgtgtcgaggagctttgtctggcgcgtgcccaccctgcccagcga 57826 to 57900
 ErhI MaeIII AluI TaqI DdeI
 BssT1I BlpI
 BstDEI

 0I CfrI XcmI
 BmyI EaeI Eco52I
 02I SduI HgaI GsuI XmaIII
cagctccgggggcacgatgaggcccgaccccaccgccgccaccataaactcccggacgcgctccagcgcggccgt base pairs
gtcgaggcccccgtgctactccgggctggggtggcggcggtggtatttgagggcctgcgcgaggtcgcgccggca 57901 to 57975
 AluI Bsp1286I BpmI EclXI
 BstZI
 EagI BsiEI

 BstMCI AccB1I AcyI Ama87I BstD102I BbvI Tsp509I
 Eco64I BbiII EheI BcoI BsrBI NspBII TspEI
 DsaI BanI Msp17I Bsp143II AccBSI PvuII Sse9I TthHB8I NlaIII
ggcgccgctcgggggggtgatgaggtggcagtagttcagctgcttgagaaaattctcgacatcatgcaggaagca base pairs
ccgcggcgagcccccccactactccaccgtcatcaagtcgacgaactcttttaagagctgtagtacgtccttcgt 57976 to 58050
 BstDSI KasI Hsp92I HaeII Eco88I AluI AcsI TaqI Hsp92II
Bsh1285I BshNI BsaHI BstH2I BsoBI MspA1I ApoI
 BsaOI Hin1I NarI BbeI AvaI HphI Bst71I

 BbiII AatII SunI SfcI DpnII MflI AclWI
 Hin1I AcyI BsiWI MaeII Bst71I Sau3AI AlwI AtsI
 AluI NlaIII MaeII SplI RsaI PstI NdeII XhoII Csp6I Tth111I
cagctccatgcggacgtccccgccgtacgtctgcagccggatctgctggtggtacggacagggtcgggccagacc base pairs
gtcgaggtacgcctgcaggggcggcatgcagacgtcggcctagacgaccaccatgcctgtcccagcccggtctgg 58051 to 58125
 Hsp92II BsaHI PspLI AfaI BbvI MboI DpnI RsaI AspI
 Msp17I BsmFI Csp6I BstYI Bsp143I AfaI
 Hsp92I Pfl23II BstSFI BstX2I Kzo9I

NcoI Bsp19I NlaIII HphI MaeII BsmAI AatII Psp1406I MvaI
StyI DsaI Eam1105I BsmAI BbiII Alw26I HphI MaeII BstOI BstOI BstD1
Eco130I AhdI Hsp92II Hin1I AcyI Esp3I NruI EcoRII EcoRII AccBSI
catggtctcggtgaaaaaggcagagacgtcacccgtggtcgcgaacgtttccaggtggcccaggagccgctcccc base pairs
gtaccagagccactttttccgtctctgcagtgggcaccagcgcttgcaaaggtccaccgggtcctcggcgagggg 58126 to 58200
ErhI BstDSI AspEI BsaI Msp17I MaeIII Bsp68I BstNI BstNI BsrBI
BssT1I BstXI Alw26I Hsp92I BsmBI BstDSI Bst2UI Bst2UI
EcoT14I EclHKI Eco31I BsaHI Tsp45I DsaI MvaI

 MvaI MvaI Eco91I HphI
 02I Csp6I BstOI BstOI BstPI MaeIII
 AflIII EcoRII BpmI EcoRII BpmI Tsp45I HphI DdeI
ctcgcgccacgcgtactccaggagcaactccagggtgaccgacagcggggtgagaaaggcggcggcctgagcctc base pairs
gagcgcggtgcgcatgaggtcctcgttgaggtcccactggctgtcgccccactctttccgccgccggactcggag 58201 to 58275
 MluI RsaI BstNI BstNI BstEII NspBII BstDEI
 AfaI Bst2UI Bst2UI EcoO65I
 GsuI GsuI PspEI MspA1I

 CfrI BsiEI BanI MvaI BpmI MvaI
 EaeI Eco52I BshNI BstOI BstOI
 GsuI XmaIII Eco64I BbvI EcoRII GsuI AluI AluI EcoRII
cagccccggccgcaggtgccgccgcagcacgcgcacctggagcgcgttgagttttagctgggcgagcttccccag base pairs
gtcggggccggcgtccacggcggcgtcgtgcgcgtggacctcgcgcaactcaaaatcgacccgctcgaaggggtc 58276 to 58350
 BpmI EclXI BsaOI AccB1I Bst71I BstNI BstNI
 BstZI BstMCI BspMI Bst2UI Bst2UI
 EagI Bsh1285I

 NdeII BfaI BsiWI AfaI BspMI
 Bsp143I TaqI Bst71I PspLI MaeII Eco88I
 DpnII SfaNI AluI AluI MaeII Csp6I Ama87I
gccgatctgggggtcgcatcgtcgaagcagctctagctgaaaaacgtacgtctgtacctgcccgagcagggccaa base pairs
cggctagacccccagcgtagcagcttcgtcgagatcgactttttgcatgcagacatggacgggctcgtcccggtt 58351 to 58425
 Sau3AI TthHB8I BbvI SplI RsaI RsaI BcoI
 Kzo9I MaeI Pfl23II Csp6I AvaI
 MboI DpnI SunI AfaI BsoBI

 Eco24I DsaI CfrI XmaIII BstMCI NotI Bsh1285I NcoI
 Bsp1286I MspA1I SstII Eco52I BstZI Eco52I ErhI BstDSI
 AlwNI TspRI BanII NspBII Sfr303I Bsh1285I XmaIII BsaOI EcoT14I
cagtttctgtcgggccgcagtgggctcggaaaccgcggccgggggcgcggccgccatggcgagtcacccggccgt base pairs
gtcaaagacagcccggcgtcacccgagcctttggcgccggcccccgcgccggcggtaccgctcagtgggccggca 58426 to 58500
 SduI BcgI EaeI KspI SacII EaeI CciNI BstMCI BssT1I
 BmyI BstDSI EagI EclXI BsaOI EagI BsiEI StyI DsaI
 FriOI BstZI Cfr42I BsiEI CfrI EclXI Eco130I Bsp19I

 BglI MaeIII XmaIII Tru9I SduI
 Tsp45I EagI Bsh1285I BsePI EcoO1
 HinfI EaeI Eco52I Tru1I AscI SfaNI Bsp12
gctgtggtttagttaaggtttgggggggggtgggtcagaggcgcgccccgcgcggactgatgcggcggcgggccc base pairs
cgacaccaaatcaattccaaacccccccccacccagtctccgcgcggggcgcgcctgactacgccgccgcccggg 58501 to 58575
 NlaIII HphI EclXI BsaOI BssHII PspOM
 Hsp92II BstZI BstMCI DraII
 PleI CfrI BsiEI MseI Bsp1286I

 BanII Cfr10I
 09I ApaI BsrFI DraII
0I FriOI BstF5I SgrAI HinfI HinfI EcoO1
ctgacatcccctctttatgcccgtcgcccgcccgcccgccccgccggtgtgccgtgattcgcggagtcggggcct base pairs
gactgtaggggagaaatacgggcagcgggcgggcgggcggggcggccacacggcactaagcgcctcagccccgga 58576 to 58650
I BmyI FokI BssAI TfiI PleI
 Eco24I Bse118I


 MvaI
 Asp700I BstOI
09I HinfI MboII HphI EcoRII
tgtgtttctttctttcccccccgaatccgttctttcttcctcacccccccctccccacacacccacccaggactc base pairs
acacaaagaaagaaagggggggcttaggcaagaaagaaggagtggggggggaggggtgtgtgggtgggtcctgag 58651 to 58725
 TfiI BstNI
 XmnI Bst2UI
 HinfI

 FriOI MaeIII BstSFI Bsp143I
 BmyI AtsI Alw26I TthHB8I MboI
PleI SduI Tth111I BsiI Eco32I DpnII
gccaccacaaggaggcgagagcccgtcgctaacccaaagacacagtcacgagacacgatatcgactgtagttgcg base pairs
cggtggtgttcctccgctctcgggcagcgattgggtttctgtgtcagtgctctgtgctatagctgacatcaacgc 58726 to 58800
 Bsp1286I AspI BssSI EcoRV SfcI NdeII
 Eco24I Tsp45I BsmAI TaqI Sau3AI
 BanII Kzo9I

 PvuI
 DpnI BstMCI
 BspCI BsaOI TthHB8I MaeI MaeII
atcgtttattttatacacaacaccaacctttccttcgaccccccccacccccgcccctagagcatatccaacgtc base pairs
tagcaaataaaatatgtgttgtggttggaaaggaagctggggggggtgggggcggggatctcgtataggttgcag 58801 to 58875
 BsiEI TaqI BfaI
 Bsh1285I
 Ple19I

 EcoO109I Eco47I Bsp143I BsaHI BsrFI
 PpuMI Psp5II HgiEI MboI DpnI BbiII NgoMI
 SinI AvaII BsaWI SinI BsmFI DpnII AclWI Hin1I BssAI
aggtcctttttctccggtggtccctccccaaacggatcgtcgccgtgaaacgcccgctttcgggcgacgccggcc base pairs
tccaggaaaaagaggccaccagggaggggtttgcctagcagcggcactttgcgggcgaaagcccgctgcggccgg 58876 to 58950
 Bme18I Bme18I NdeII AlwI Msp17I NgoAIV
 HgiEI Eco47I AvaII Sau3AI Hsp92I Bse118I
 DraII Kzo9I AcyI MroNI

 BstZI NaeI BsaOI BsaHI
 EaeI EclXI BstMCI BbiII Tru1I
 HgaI XmaIII Hin1I BstF5I Tru9I
gcccccgccgccgccgccaaaccgccgaacgacgccgcgtggtcatcctcgtcgccgaaatccccaaagttaaac base pairs
cgggggcggcggcggcggtttggcggcttgctgcggcgcaccagtaggagcagcggctttaggggtttcaatttg 58951 to 59025
 CfrI BsiEI Msp17I FokI MseI
 EagI Bsh1285I Hsp92I
 Cfr10I Eco52I AcyI HgaI

 AccB1I NarI HaeII BstOI MvaI AccI
 KasI BbiII EheI BglI EcoO109I BssSI BstOI TthHB8I
 Eco64I BsaHI BbeI EcoRII BglI EcoRII BpmI HgaI TaqI
acctccccggcggcgccgagctggctgaccagggcctccgcctcgtgggccacctccagggccgcgtcggtcgac base pairs
tggaggggccgccgcggctcgaccgactggtcccggaggcggagcacccggtggaggtcccggcgcagccagctg 59026 to 59100
 BanI Msp17I Bsp143II MvaI BsiI BstNI SalI
 BshNI Hsp92I AluI BstNI DraII Bst2UI BsiEI
 Hin1I AcyI BstH2I Bst2UI GsuI Bsh1285I

 BsaOI MvaI
 BstOI BstDEI
 HindII NlaIII EcoRII BpmI EcoNI Csp6I GsuI
cactcgccatgcccgcgctccagggcgcgggtggtaaactccatcatttcctcgctcaggtactcgtcctccagc base pairs
gtgagcggtacgggcgcgaggtcccgcgcccaccatttgaggtagtaaaggagcgagtccatgagcaggaggtcg 59101 to 59175
BstMCI Hsp92II BstNI XcmI DdeI RsaI BpmI
 HincII Bst2UI AfaI
 GsuI

 BstH2I BsrI DpnII SfcI AluI AlwNI EcoRII ApaI Bsh1285I
 BbvI BseNI MboI DpnI PvuII EcoO109I FriOI Bst2UI
 Bsp143II TthHB8I Kzo9I MspA1I Bsp120I Eco24I BstOI
agcgccagccagtcctcgatctgcagctgctgggtgcgggggcccaggctcttgacggtcgccacaaacacgctg base pairs
tcgcggtcggtcaggagctagacgtcgacgacccacgcccccgggtccgagaactgccagcggtgtttgtgcgac 59176 to 59250
 HaeII BsrSI NdeII BstSFI Bst71I PspOMI BmyI BstNI BsiEI
 Bst71I Bse1I Sau3AI PstI BbvI DraII Bsp1286I MvaI BsaOI
 TaqI Bsp143I NspBII SduI BanII BstMCI

 PspOMI BmyI
 BstMCI Bst71I BsaMI EcoO109I
 BbvI BsiEI BsrDI AluI BsmI BsiI SduI
ctggcgaccgccgccccgccctccgcaatgatgccccggagctgctcgcacagcgaatgctcgtgggccccgccc base pairs
gaccgctggcggcggggcgggaggcgttactacggggcctcgacgagcgtgtcgcttacgagcacccggggcggg 59251 to 59325
 Bst71I Bsh1285I SfaNI BbvI Mva1269I DraII
 BsaOI Bsp120I Eco24I
 BssSI Bsp1286I

 Ama87I Alw26I BbiII MvaI MvaI Bsp143I
ApaI BcoI BsmAI Msp17I BstOI BstOI MboI
FriOI BsoBI TaqI Hsp92I EcoRII EcoRII DpnII
ccgagactcgacgccgcgcacacaaacccggccctggggcaggccaggacaaacttgcgggtgcggtcaaagatc base pairs
ggctctgagctgcggcgcgtgtgtttgggccgggaccccgtccggtcctgtttgaacgcccacgccagtttctag 59326 to 59400
BanII HinfI PleI BsaHI BstNI BstNI NdeII
 Eco88I Hin1I AcyI Bst2UI Bst2UI Sau3AI
 AvaI TthHB8I HgaI EcoNI Kzo9I

 Bsp1286I MroNI
 DpnI BbvI SduI Bse1I BstMCI NgoAIV
 NspBII AflIII BglI BsrSI BsiEI NgoMI
agcagcgggcacgcgtttttgccgcccagcaggctggcccagttcccggcctgaaacacgcggtcgttgccggcc base pairs
tcgtcgcccgtgcgcaaaaacggcgggtcgtccgaccgggtcaagggccggactttgtgcgccagcaacggccgg 59401 to 59475
 MspA1I MluI BstXI BseNI Bsh1285I Bse118I
 Bst71I BsrI BsaOI BsrFI
 BmyI BssAI

Cfr10I
 NlaIII BstDEI BsePI BspMI
 CfrI DdeI BcgI EaeI BbvI
atgccgtagtatttgctgatgctgaggcccagcacgaccatcgggcgcgcggccatcacgggccgcagcaggttg base pairs
tacggcatcataaacgactacgactccgggtcgtgctggtagcccgcgcgccggtagtgcccggcgtcgtccaac 59476 to 59550
 SfaNI BssHII Bst71I
 NaeI CfrI
EaeI Hsp92II

 NruI BbiII AatII Eco64I Hsp92I HaeII HgaI SduI BanII
 Bst71I Hin1I AcyI Bst2UI Hin1I AcyI TthHB8I BsePI PspOMI BmyI EcoRII
 AluI NlaIII MaeII BstOI BshNI BsaHI BstH2I BssHII EcoO109I ApaI
cagctcgcgaacatggacgtccaggcgccggggtgcgcgtcgagggagtccatcagcgcgcgggccccggcctcc base pairs
gtcgagcgcttgtacctgcaggtccgcggccccacgcgcagctccctcaggtagtcgcgcgcccggggccggagg 59551 to 59625
 BbvI Hsp92II BsaHI MvaI AccB1I NarI BbeI HinfI Bsp120I FriOI
 Bsp68I Msp17I EcoRII KasI BbiII EheI TaqI DraII Eco24I
 Hsp92I BstNI BanI Msp17I Bsp143II PleI Bsp1286I

 GsuI EcoRII ApaI SfiI CfrI EclXI BsgI HgiEI Eco47I XmaIII
 BpmI EcoO109I FriOI Bst2UI NotI BstMCI Bme18I CfrI
 MvaI Bsp120I Eco24I BstOI EagI Eco52I BsmFI DraII BsmFI EaeI
aggcccgcgccgccctgcggggcccaggcggccgccgcctgcacgctggggggacggcgggacccggcgatgacg base pairs
tccgggcgcggcgggacgccccgggtccgccggcggcggacgtgcgacccccctgccgccctgggccgctactgc 59626 to 59700
 BstOI PspOMI BmyI BstNI EaeI XmaIII BsaOI SinI AvaII BstZI
 Bst2UI DraII Bsp1286I MvaI BstZI BsiEI PpuMI Psp5II EagI
BstNI SduI BanII BglI CciNI Bsh1285I EcoO109I EclXI

 TthHB8I MboI Bsp143I Hsp92II MscI
 BsiEI AfaI AfaI BstX2I Kzo9I BalI Csp6I NlaIII
 Eco52I Csp6I Csp6I BstYI MflI EaeI NlaIII EaeI
gccgtgagggtgtttatgaagtacgtcgagtggtcgcagtacctcaagatctggttggccatgtagtacatggcc base pairs
cggcactcccacaaatacttcatgcagctcaccagcgtcatggagttctagaccaaccggtacatcatgtaccgg 59701 to 59775
 BstMCI RsaI RsaI DpnII BglII CfrI XcmI CfrI
 Bsh1285I MaeII NdeII XhoII MscI RsaI Hsp92II
 BsaOI TaqI Sau3AI DpnI MluNI AfaI MluNI

 BseNI MvaI MvaI
 Bse1I BstOI Tru1I BstOI Tru1I
 BalI MaeII EcoRII Tru9I EcoRII Tru9I
agttcgctcacgttattgggggccaggttgataaagttaatcgcgccgtagtccagggagaacctcttaatgaac base pairs
tcaagcgagtgcaataacccccggtccaactatttcaattagcgcggcatcaggtccctcttggagaattacttg 59776 to 59850
 BsrSI BstNI MseI BstNI MseI
 Bst2UI Bst2UI
 BsrI

 HgiEI
 BsaI SinI
 Alw26I AluI GsuI Eco47I
gcgatggtctctatgtcctcgcgcgacaagagccgggcggggagctggttgcgctggagggcggtccagaaccac base pairs
cgctaccagagatacaggagcgcgctgttctcggcccgcccctcgaccaacgcgacctcccgccaggtcttggtg 59851 to 59925
 Eco31I BpmI
 BsmAI Bme18I
 AvaII

 Cfr9I PspALI
 BcoI XmaI
 TspRI TthHB8I AvaI Asp700I
tgcgggttcggctggttcgaccccgggggcttgccgttgggaaagatgaccgcgtggaactgcttcagcaggaag base pairs
acgcccaagccgaccaagctggggcccccgaacggcaaccctttctactggcgcaccttgacgaagtcgtccttc 59926 to 60000
 TaqI Ama87I SmaI XmnI Eco57I
 PspAI BsoBI
 Eco88I

 Bme18I EcoT14I
 CpoI AvaII FokI BstH2I BpmI StyI
 NspBII CspI BseRI Bsp143II Eco13
cccagcggtccgaggaggatgtccacgcgcttgtcgggcttctggtaggcgctctggaggctggcgacccgcgcc base pairs
gggtcgccaggctcctcctacaggtgcgcgaacagcccgaagaccatccgcgagacctccgaccgctgggcgcgg 60001 to 60075
 MspA1I RsrII BstF5I HaeII GsuI ErhI
 SinI Eco47I BssT1I
 HgiEI

 EcoT14I
 HgaI BstH2I StyI
 0I AflIII Bsp143II HgaI Eco130I
ttggcggcctcggacgcgttggcgctcgcgcccgcgaacaacacgcggctcttgacgcgcagttccttgggaaac base pairs
aaccgccggagcctgcgcaaccgcgagcgcgggcgcttgttgtgcgccgagaactgcgcgtcaaggaaccctttg 60076 to 60150
 BglI MluI HaeII ErhI
 BssT1I


 EcoT14I NgoMI EagI Eco52I
 StyI MaeIII Bst71I MroNI Cfr10I BsiEI
 Eco130I MaeII TthHB8I BbvI EaeI NgoAIV EclXI BstMCI
ccaagggtcacgcgggcaacgtcgccctcgaagctgctctcggcgggggccgtctggccggccgttaggctgggg base pairs
ggttcccagtgcgcccgttgcagcgggagcttcgacgagagccgcccccggcagaccggccggcaatccgacccc 60151 to 60225
 ErhI Tsp45I TaqI AluI CfrI Bse118I NaeI BsaOI
 BssT1I BssAI BstZI FseI
 BsrFI XmaIII Bsh1285I

 BpiI Eco47I
 BstMCI Bbv16II HgiEI
 BsiEI HgaI SinI
gcgcagatagccgccccctccgagagcgcgaccgtcagcgtcttcgccgacaggaacccgttgttgaacaggtcc base pairs
cgcgtctatcggcgggggaggctctcgcgctggcagtcgcagaagcggctgtccttgggcaacaacttgtccagg 60226 to 60300
 Bsh1285I BpuAI Bme18I
 BsaOI BbsI AvaII
 MboII

 BssAI TspEI BstMCI
 HgaI AgeI Bse118I BsiEI
 NlaIII BbvI BsrFI TthHB8I
atgacgcgccgccgcagcaccggttggaattgattgcgaaagttgcgcccctcgaccgactgcccggcgaacacc base pairs
tactgcgcggcggcgtcgtggccaaccttaactaacgctttcaacgcggggagctggctgacgggccgcttgtgg 60301 to 60375
 Hsp92II Bst71I Cfr10I TaqI
 PinAI Sse9I Bsh1285I
 BsaWI Tsp509I BsaOI

 BsrI Bst2UI DraII AlwNI BstOI RsaI AvaII BbvI
 DraIII BseNI BstNI HgiEI EcoRII BstNI Csp6I SfcI Bst71I
 BstDSI TspRI BstDEI SinI AvaII AccB7I Bst2UI HgiEI BseRI BbvI
ccgtggcactggctcagggccaggtcctggtacacggcgaggttggaccgccgcgcgaggagctgcagcaggggg base pairs
ggcaccgtgaccgagtcccggtccaggaccatgtgccgctccaacctggcggcgcgctcctcgacgtcgtccccc 60376 to 60450
 DsaI BsrSI EcoRII Bme18I Eco47I Van91I AfaI Eco47I AluI PstI
 Bse1I BstOI EcoO109I PflMI MvaI SinI BstSFI
 DdeI MvaI PpuMI Psp5II Esp1396I Bme18I Bst71I

 Bme18I
 BmyI RsaI Eco47I Tru1I
 SduI BspMI SinI NlaIII EaeI Tru9I
cacggcccgcaggtgtacgggtccagcgacagcgacatggcgtggttggcctcggccagaccgtcgcggaactta base pairs
gtgccgggcgtccacatgcccaggtcgctgtcgctgtaccgcaccaaccggagccggtctggcagcgccttgaat 60451 to 60525
 Bsp1286I Csp6I AvaII Hsp92II CfrI MseI
 AfaI
 HgiEI

 MboI Kzo9I AviII MspA1I MboI Kzo9I MspA1I
 DpnII EcoNI AluI DpnII AclWI Bst71I
 TthHB8I FspI SfaNI Bsp68I DpnI AluI
aagttgcgcccctcgatcaggttgcgcatcagctgttccacctcgcgatccaccagctgcttgatgttgttcacc base pairs
ttcaacgcggggagctagtccaacgcgtagtcgacaaggtggagcgctaggtggtcgacgaactacaacaagtgg 60526 to 60600
 TaqI Bsp143I PvuII NruI Bsp143I BbvI
 NdeII DpnI BcgI NspBII NdeII AlwI NspBII
 Sau3AI Acc16I Sau3AI PvuII

 GsuI MvaI DsaI BshNI EcoRII Bme18I
 BsgI BsmBI BstOI Eco64I BstOI AvaII
HphI DraIII Alw26I EcoRII BstDSI SduI BstNI HgiEI
accgtgtgcagggcctcgcggttgccgataatcgtctccagcctccccagggccgtgggcaccgcctggtccacg base pairs
tggcacacgtcccggagcgccaacggctattagcagaggtcggaggggtcccggcacccgtggcggaccaggtgc 60601 to 60675
 MslI EcoO109I BsmAI BstNI BstXI Bsp1286I SinI
 DraII Esp3I Bst2UI BanI BmyI MvaI Eco47I
 BpmI AccB1I Bst2UI MaeII

 AfaI DraII Eco88I Ecl136II Eco24I BsiHKAI AfaI HgiEI
 RsaI EcoO109I AvaI AluI BmyI SacI SstI Hsp92II BsmBI SinI
 Csp6I Sfr274I TaqI Bsp1286I BanII CfrI CfrI MaeII Alw26I NlaIII Eco47I
tactgcagggcctcgagctcggccatgacgcgctcggtggccgcgcggtacgtctcctgcatgatggtccgggtg base pairs
atgacgtcccggagctcgagccggtactgcgcgagccaccggcgcgccatgcagaggacgtactaccaggcccac 60676 to 60750
BsaAI PstI Ama87I TthHB8I AspHI FriOI EaeI EaeI RsaI BsmAI Hsp92II
 BstSFI XhoI PaeR7I SduI Bbv12I Alw21I HgaI Csp6I Esp3I Bme18I
 SfcI BcoI BsoBI EcoICRI Psp124BI NlaIII AvaII

 Eco47I
 HgiEI RsaI
 SinI BssHII Eco57I BglI MaeII AlwNI
ttctcggacccgtccgcgcgcttcagggccgagaaggcggcgtagttccccagcacgtcgcagtcgctgtacgcg base pairs
aagagcctgggcaggcgcgcgaagtcccggctcttccgccgcatcaaggggtcgtgcagcgtcagcgacatgcgc 60751 to 60825
 Bme18I BsePI Csp6I
 AvaII AfaI


 BpiI Eco88I BstH2I BmyI BbvI
 Bbv16II PspAI PspALI NruI SduI ApaI
 MboII Ama87I SmaI Bsp68I Bsp120I BanII
ctgttcatcgttccgaagaccccaatggccccccgggcggcgctcgcgaacttggggtggcgggcccgcagccgc base pairs
gacaagtagcaaggcttctggggttaccggggggcccgccgcgagcgcttgaaccccaccgcccgggcgtcggcg 60826 to 60900
 BpuAI BcoI XmaI Bsp143II PspOMI FriOI
 BbsI Cfr9I BglI HaeII Bsp1286I
 AvaI BsoBI Eco24I Bst71I

 BsaI RsaI BsoBI
 BsePI Alw26I BmyI Eco88I
 SfaNI HgaI BssHII TthHB8I SduI Ama87I
atcagcgtcgtgtgcgcgcaggcgtggcgggtctcgaaggtacacaggttgcagggcacgtcggtctggcccgag base pairs
tagtcgcagcacacgcgcgtccgcaccgcccagagcttccatgtgtccaacgtcccgtgcagccagaccgggctc 60901 to 60975
 BcgI TaqI Csp6I Bsp1286I BcoI
 Eco31I MaeII AvaI
 BsmAI AfaI HinfI

 Van91I Eco64I Hsp92I HaeII DrdI Bst71I EcoRII
 PflMI Bst2UI Msp17I EheI PleI BbvI HaeII Bst2UI
 PleI MaeII AflIII EcoRII BstOI BshNI BsaHI BbeI Aor51HI BstNI
tccgcgacgtagcgaaacacgtccatctcctggcgcccgacgatgactccgccgtcgcagcgctccaggtaaaac base pairs
aggcgctgcatcgctttgtgcaggtagaggaccgcgggctgctactgaggcggcagcgtcgcgaggtccattttg 60976 to 61050
 MaeII AccB7I MvaI AccB1I AcyI HinfI Eco47III BstOI
 Esp1396I KasI BbiII Bsp143II AfeI BstH2I GsuI
 BstNI BanI Hin1I NarI BstH2I Bsp143II MvaI

 MluNI MscI BstNI AspHI MroNI Cfr10I MvaI
 CfrI NlaIII BstOI BmyI SgrAI Bse118I
BpmI SfaNI SfiI EaeI BalI SduI BsiHKAI NgoAIV BstOI
agcatcttggccagcagggccggagagaacccgcacagcatggccaggtgctcgccggcgaactcctgggttccg base pairs
tcgtagaaccggtcgtcccggcctctcttgggcgtgtcgtaccggtccacgagcggccgcttgaggacccaaggc 61051 to 61125
 EaeI BglI CfrI EcoRII Bsp1286I BssAI NaeI Bst2UI
 MscI Hsp92II MvaI Bbv12I NgoMI EcoRII
 BalI MluNI Bst2UI Alw21I BsrFI BstNI

 AccB1I NarI HaeII BanI Msp17I EheI RsaI Eco88I BanI BmyI PmlI HgiEI
 KasI BbiII EheI DsaI Hin1I NarI BstH2I PspAI PspALI Bsp1286I BbrPI
 Eco64I BsaHI BbeI Eco64I Hsp92I HaeII Ama87I BsoBI SduI BsaAI SinI
ccgacgaggggcgccgtggggcgcccctcgtacccgggcaccacgtggccctcgcggtccagctgcgggttggcc base pairs
ggctgctccccgcggcaccccgcggggagcatgggcccgtggtgcaccgggagcgccaggtcgacgcccaaccgg 61126 to 61200
 BanI Msp17I Bsp143II AccB1I AcyI Csp6I BcoI XmaI BshNI Eco72I Bme18I
 BshNI Hsp92I BstDSI BshNI BsaHI BbeI AfaI AvaI Eco64I MaeII DraIII
 Hin1I AcyI BstH2I KasI BbiII Bsp143II Cfr9I SmaI AccB1I PmaCI AvaII

 NspBII MaeII MslI BssSI Eco47I SinI MroNI Cfr1
 PvuII Bst71I BbrPI BsiI HgiEI StyI HgiEI BsrFI
 Eco47I EaeI PmlI SduI SinI ErhI Bme18I NgoMI
gccacgtgcgtgccgggcacgagaaagaagcggtaaaaggagggcttgctgtggtccttggggtccgccggcccg base pairs
cggtgcacgcacggcccgtgctctttcttcgccattttcctcccgaacgacaccaggaaccccaggcggccgggc 61201 to 61275
 MspA1I Eco72I Bsp1286I Bme18I EcoT14I BssAI NaeI
 AluI BbvI BsaAI BmyI AvaII BssT1I Eco47I Bse118I
 XcmI CfrI PmaCI Eco130I AvaII NgoAIV

 0I Hsp92I NcoI Bsp19I BshNI Hsp92I BbeI
 Hin1I HgaI Tsp509I StyI DsaI NlaIII BbiII Bsp143II
 BglI BsaHI EcoNI Sse9I Eco130I BanI AccB1I NarI HaeII
gcgtcgtccacctcggtcaggtggagggccgaattggtgctgaacaccatggcgcccacgaggcccgcggcgcgc base pairs
cgcagcaggtggagccagtccacctcccggcttaaccacgacttgtggtaccgcgggtgctccgggcgccgcgcg 61276 to 61350
 BbiII TspEI ErhI BstDSI Hsp92II AcyI BstH2I
 AcyI BssT1I Eco64I Msp17I EheI BsiI
 Msp17I EcoT14I KasI Hin1I BsaHI BglI

 BssSI SstII EcoRII RsaI BshNI BsaHI NgoMI HaeII DsaI SstII BstOI BsaAI
 KspI Cfr42I Bst2UI BanI Msp17I SgrAI Bse118I BstDSI Cfr42I MvaI PmlI HphI
 DsaI Sfr303I BstOI BglI Hin1I NarI BssAI Bsp143II KspI SacII EcoNI BbrPI
gccaggtacgccccgacggcgccggcgcgggccgcgggcgtttcctggccctcaagcaggggccacgtggtgatg base pairs
cggtccatgcggggctgccgcggccgcgcccggcgcccgcaaaggaccgggagttcgtccccggtgcaccactac 61351 to 61425
 MspA1I BsePI MvaI Eco64I Hsp92I MroNI Cfr10I NaeI Sfr303I Bst2UI PmaCI
 NspBII BssHII Csp6I KasI BbiII EheI NgoAIV BbeI NspBII EcoRII MaeII DraIII
 BstDSI SacII BstNI AfaI AccB1I AcyI BsrFI BstH2I MspA1I BstNI Eco72I

 BstOI BstOI BbiII
 XcmI PleI GsuI MvaI Hsp92I
 TthHB8I HinfI MvaI EcoRII Hin1I HgaI
tcggggggcggctcgtcaaagaccgccatcgacacgatggactccagggccagggcggcgtcgcccgccatcacc base pairs
agccccccgccgagcagtttctggcggtagctgtgctacctgaggtcccggtcccgccgcagcgggcggtagtgg 61426 to 61500
 TaqI EcoRII BpmI BglI AcyI
 BstNI BstNI Msp17I
 Bst2UI Bst2UI BsaHI

 MvaI BstH2I DraII Eco24I BsaHI
 BstOI HaeII PspOMI BmyI BbiII BsePI
HphI BstNI Bsp143II BglI SduI BanII Hin1I BssHII NspBII EcoRII
gaggccaggcgctgctcaaacccgcccgccgggcccttgttcccggcgtcgcgcgcgccccgctggggcttaccc base pairs
ctccggtccgcgacgagtttgggcgggcggcccgggaacaagggccgcagcgcgcgcggggcgaccccgaatggg 61501 to 61575
 EcoRII AlwNI Bsp120I FriOI Msp17I MspA1I
 Bst2UI BbvI EcoO109I ApaI Hsp92I
 Bst71I Bsp1286I AcyI HgaI

MvaI MvaI
BstOI SfiI BstOI
 TthHB8I MaeII BsiI TthHB8I BstNI
tggctggcctcgaaggccgtgaacgtaatgtcggcggggagggccgcgccctcgtggttttcgtcgaacgccagg base pairs
accgaccggagcttccggcacttgcattacagccgcccctcccggcgcgggagcaccaaaagcagcttgcggtcc 61576 to 61650
BstNI TaqI BssSI TaqI EcoRII
Bst2UI BglI Bst2UI


 EagI Eco52I BstDSI AcyI BmyI BsiEI
 CfrI EclXI BsaOI BbiII MaeII BmyI DsaI SduI ApaI TaqI
 EaeI XmaIII SfiI Hin1I HgaI SduI BstDSI Bsp120I BanII TthHB8I
tgggcggccgcgcgggccacggcgtccacgttccgggcacgcagggccacggcggcgggcccgacgaccgcctcg base pairs
acccgccggcgcgcccggtgccgcaggtgcaaggcccgtgcgtcccggtgccgccgcccgggctgctggcggagc 61651 to 61725
 BstZI BsiEI BglI Msp17I Bsp1286I BglI PspOMI FriOI BstMCI
 CciNI Bsh1285I Hsp92I Bsp1286I BsaOI
 BglI NotI BstMCI DsaI BsaHI Eco24I Bsh1285I

 Tsp509I Bsp143I Bsp106I
 TspEI MboI DpnI Bsa29I
 Sse9I DpnII BspCI BsiEI
aacagcaggcgggcgagggggcggttgaaaaacggaagggggtagttgaaattctccccgatcgatcggtggttg base pairs
ttgtcgtccgcccgctcccccgccaactttttgccttcccccatcaactttaagaggggctagctagccaccaac 61726 to 61800
 AcsI NdeII TthHB8I Bsh1285I
 ApoI Sau3AI BspDI BspXI
 Kzo9I BanIII Ple19I

BscI PvuI Sau3AI Tru1I Kzo9I BbvI
 TaqI BstMCI Kzo9I DpnII DpnI PstI SfaNI
 Bsu15I DpnII Tru9I Sau3AI SfcI NspBII MslI BstF5I
cagttaaacggatcggcgatgacccggctaaaatccggcataaacatctgcagcggatacacggggatgcggtga base pairs
gtcaatttgcctagccgctactgggccgattttaggccgtatttgtagacgtcgcctatgtgcccctacgccact 61801 to 61875
 BsaOI Bsp143I Bsp143I BstSFI FokI
 BseCI NdeII MseI MboI AclWI MspA1I
 ClaI MboI DpnI NdeII AlwI Bst71I

 EcoO65I
 BstEII
 HgaI Eco91I BstF5I SfaNI SfaNI
acctccgcgtccccgatggttaccttgtccatcccgcccagatgcaggaaggtgttgctgatgcacacggcctcc base pairs
tggaggcgcaggggctaccaatggaacaggtagggcgggtctacgtccttccacaacgactacgtgtgccggagg 61876 to 61950
HphI BsmFI MaeIII FokI
 BstPI
 PspEI

 MboI Kzo9I CspI Bme18I BsrI
 BclI Bsp143I Bme18I AvaII BseNI BstD102I
 FbaI Sau3AI CpoI AvaII Eco47I AccBSI HgaI
cggaagccctccgtgatcaccagatacagcaaggcccggtccgggtccagtccgagccgctcgcacagcgcgtcc base pairs
gccttcgggaggcactagtggtctatgtcgttccgggccaggcccaggtcaggctcggcgagcgtgtcgcgcagg 61951 to 62025
 DpnII DpnI SinI Eco47I BsrSI BsrBI
 NdeII HphI HgiEI SinI Bse1I
 Ksp22I RsrII HgiEI

 BsiI
 BsmBI BsePI BstD1
 Alw26I AscI AccBSI
cccgtcgtctcgtgctttaggtcgcagggccggggcgcgtagtccgcgaagccaaaatgcgggcgcgcccgctcg base pairs
gggcagcagagcacgaaatccagcgtcccggccccgcgcatcaggcgcttcggttttacgcccgcgcgggcgagc 62026 to 62100
 BsmFI BsmAI BssHII BsrBI
 Esp3I
 BssSI

 BstOI MvaI EagI Eco52I
 02I EcoRII BstOI CfrI EclXI BsaOI
 HgaI EcoO109I EcoRII EaeI XmaIII MaeII
cagagccgcgtcaggttgggggcctgggtgctgggggccaggtggcggccgccgtgaaagacgtaaacggacggg base pairs
gtctcggcgcagtccaacccccggacccacgacccccggtccaccgccggcggcactttctgcatttgcctgccc 62101 to 62175
 DraII MvaI BstNI BstZI Bsh1285I
 BstNI Bst2UI CciNI BstMCI
 Bst2UI NotI BsiEI

 NspBII SinI BsmFI EcoT14I EcoO109I BstDSI
 DsaI Sfr303I AvaII BssT1I PpuMI Psp5II BstMCI
 SfcI HindIII BsmFI KspI SacII Eco130I SinI AvaII BsiEI
ctgtagtgcgagggcataagcttgagggacaccgcggtccccccaaggcccgtcgtgcgggacccgacgaccgcg base pairs
gacatcacgctcccgtattcgaactccctgtggcgccaggggggttccgggcagcacgccctgggctgctggcgc 62176 to 62250
 BstSFI AluI BstDSI SstII HgiEI StyI Bme18I Bsh1285I
 DrdI Cfr42I Eco47I HgiEI Eco47I BsaOI
 MspA1I Bme18I ErhI DraII BsmFI DsaI

 EaeI SstII DsaI Hsp92I
 Sfr303I BglI BstD102I Msp17I
 NspBII Cfr42I AccBSI DrdI BsaHI HgaI
gccacgttggcctcaaacccgctctccacggtcaggccgacgatgaggggcgcgacggcgacgtccgcgtcgccg base pairs
cggtgcaaccggagtttgggcgagaggtgccagtccggctgctactccccgcgctgccgctgcaggcgcagcggc 62251 to 62325
 KspI SfiI BsrBI Hin1I AcyI
 CfrI SacII BstDSI BbiII AatII
 MspA1I MaeII MaeII

 BbvI BstNI Pme55I EagI BstMCI RsaI
MspA1I Bst71I GsuI SseBI XmaIII BglI BsaAI
 BssHII AluI Bst2UI Eco147I Eco52I EaeI AflIII
ctgcgcgccgacagtagcgacagcagctccaggccttcggccggacaggcgcggccatacacgtaccccatcggc base pairs
gacgcgcggctgtcatcgctgtcgtcgaggtccggaagccggcctgtccgcgccggtatgtgcatggggtagccg 62326 to 62400
NspBII BbvI MvaI AatI CfrI BsiEI CfrI MaeII
 BsePI EcoRII BpmI EaeI EclXI BsaOI Csp6I
 Bst71I BstOI StuI BstZI Bsh1285I AfaI

 NcoI Bsp19I Sau3AI Cfr10I
 StyI DsaI NdeII Bse118I
 PshAI Eco130I Hsp92II Kzo9I BssAI BstMCI
cccggaggaaccttgacggtggtcgtcgttttgggcttggtgtccatggctttcgggagatcggcgaccggcagg base pairs
gggcctccttggaactgccaccagcagcaaaacccgaaccacaggtaccgaaagccctctagccgctggccgtcc 62401 to 62475
 ErhI BstDSI DpnII BsrFI BsaOI
 BssT1I NlaIII MboI DpnI BsiEI
 EcoT14I Bsp143I Bsh1285I

 SduI ApaI HincII
 EcoO109I BanII BstMCI AccI
 Bsp120I FriOI BsiEI SalI BbvI
aacgggggcccggcaagacgaccgggggcagacgggggaggccgcgcgtggtcgacggctgctgcccgccgtcgt base pairs
ttgcccccgggccgttctgctggcccccgtctgccccctccggcgcgcaccagctgccgacgacgggcggcagca 62476 to 62550
 PspOMI Eco24I Bsh1285I TthHB8I Bst71I
 DraII BmyI BsaOI TaqI
 Bsp1286I HindII

 BsrFI BsaMI BstH2I BstNI
 BsmBI BssAI BsmI HaeII BstD102I
 Alw26I TthHB8I Bse118I Bsp143II AccI AccBSI MvaI
ctctccgatggggtcgaatgccggcgctgggggtggggtctacacccgcccgttcgccgagcggcccctggtggg base pairs
gagaggctaccccagcttacggccgcgacccccaccccagatgtgggcgggcaagcggctcgccggggaccaccc 62551 to 62625
 BsmAI TaqI MroNI Cfr10I BsrBI Bst2UI
 Esp3I NgoMI Mva1269I EcoRII
 NgoAIV NaeI BstOI

 Sau3AI Ama87I BsoBI
 NdeII AlwI EcoO109I XmaI
 BstF5I BstF5I BsaWI Kzo9I Bsp120I Eco88I
ggtgggatgggtgggatggggtgggcgagaatggcccgccaccggatcgcgccggacgggggggcccggggttgg base pairs
ccaccctacccaccctaccccacccgctcttaccgggcggtggcctagcgcggcctgcccccccgggccccaacc 62626 to 62700
 FokI FokI DpnII AclWI PspOMI Cfr9I
 MboI DpnI DraII PspAI SduI
 Bsp143I BcoI AvaI

 BanII Eco147I XmaIII
 Eco24I NspBII TthHB8I CfrI
 FriOI SmaI GsuI HinfI Pme55I BstF5I AluI EaeI
gcaaggtttgggcgcaaggctccagcggcgattcgagaggcctgcggatggcggcccagagctgggtatgctcgg base pairs
cgttccaaacccgcgttccgaggtcgccgctaagctctccggacgcctaccgccgggtctcgacccatacgagcc 62701 to 62775
Bsp1286I BpmI TfiI StuI BcgI FokI BstZI
 ApaI MspA1I TaqI AatI EagI
BmyI PspALI SseBI EclXI

 SfiI EagI BsrFI BsaOI BsaHI EcoO109I ApaI HgiEI
 BsiEI BglI EclXI BsiEI RsaI BbiII PspOMI Eco24I SinI Eco47I
 Eco52I EaeI Eco52I Bsh1285I Hin1I Bsp120I FriOI CpoI RsrII
ccggggcggccggtatatgtacggcgtgctgggaggggcggcgtcgggccccgcccacggtccgccacgccccgc base pairs
ggccccgccggccatatacatgccgcacgaccctccccgccgcagcccggggcgggtgccaggcggtgcggggcg 62776 to 62850
 BstMCI CfrI BssAI BstMCI Msp17I DraII BmyI BstDSI AvaII
 Bsh1285I XmaIII Cfr10I AfaI Hsp92I SduI BanII Bme18I
 BsaOI BstZI Bse118I Csp6I AcyI HgaI Bsp1286I DsaI CspI


 HgaI EaeI
gcgtcatcggcagggggcgtggccgcccttctaaaaaaagtgagaacgcgaagcgttcgcactttgtcctaataa base pairs
cgcagtagccgtcccccgcaccggcgggaagatttttttcactcttgcgcttcgcaagcgtgaaacaggattatt 62851 to 62925
 CfrI


 EaeI
tatatatactattaggacaaagtgcgaacgcttcgcgttctcactttttttagaagggcggccacgccccctttg base pairs
atatatatgataatcctgtttcacgcttgcgaagcgcaagagtgaaaaaaatcttcccgccggtgcgggggaaac 62926 to 63000
 CfrI


 MaeII MaeIII AvaI HphI CfrI BssAI BsiEI NaeI KspI SacII
 BbiII Tsp45I Eco88I EaeI EclXI BsrFI BstMCI NspBII Eco64I
 Hin1I AcyI Ama87I SmaI EagI NgoMI Bsh1285I SfiI BstDSI Cfr42I
acgtcacgctcacccgggcggccggccgcccataagcgcggcctgccgggccgataaaaagaaaccgcggcgccc base pairs
tgcagtgcgagtgggcccgccggccggcgggtattcgcgccggacggcccggctatttttctttggcgccgcggg 63001 to 63075
 Msp17I BcoI XmaI BstZI MroNI Cfr10I BglI DsaI SstII
 Hsp92I PspAI BsoBI XmaIII NgoAIV BsaOI MspA1I BanI
 BsaHI AatII Cfr9I PspALI Eco52I Bse118I FseI Sfr303I

 Msp17I EheI DsaI SstII Esp1396I TaqI EcoO109I PspAI BsmFI AvaII
 Hsp92I HaeII NspBII AccB7I BseNI PpuMI Psp5II AvaI SmaI Eco47I
 AccB1I AcyI BstDSI Cfr42I TspRI BsrI SinI AvaII Cfr9I PspALI BsrFI
ccgcggacaccacacactggctctcgaaccccggacgcgcagaagggacccgggcgcgggtccgccggtaagagc base pairs
ggcgcctgtggtgtgtgaccgagagcttggggcctgcgcgtcttccctgggcccgcgcccaggcggccattctcg 63076 to 63150
 KasI BbiII Bsp143II Sfr303I Van91I TthHB8I Bme18I BcoI Eco88I Bme18I Cfr10I
 Hin1I NarI BbeI MspA1I PflMI Bse1I HgiEI Eco47I XmaI SinI BssAI
 BshNI BsaHI BstH2I KspI SacII BsrSI HgaI DraII Ama87I BsoBI HgiEI Bse118I

 Eco88I
 BshNI Ama87I
 Eco64I BstF5I AluI HphI
cggggggaacatcggcaccgccatcccaccccgagctgttgggtgggcgggtgggggggctggtgaggcggtggt base pairs
gccccccttgtagccgtggcggtagggtggggctcgacaacccacccgcccacccccccgaccactccgccacca 63151 to 63225
 BanI FokI BsoBI
 AccB1I BcoI
 AvaI

 BsiEI KspI SacII Eco88I
 Bse118I NspBII PspAI
 BssAI BstMCI BstDSI Cfr42I Ama87I
gggagggggcggcgtatagcaggacaacgaccggcggcgatgttttgtgccgcgggcggcccggcttcccccggg base pairs
ccctcccccgccgcatatcgtcctgttgctggccgccgctacaaaacacggcgcccgccgggccgaagggggccc 63226 to 63300
 BsrFI BsaOI DsaI SstII BcoI
 Cfr10I MspA1I Cfr9I
 Bsh1285I Sfr303I AvaI

 BsrFI BcoI Hsp92I Eco88I
 PspALI Bse118I AvaI Msp17I PspAI PspALI BshNI
 MroNI Cfr10I Hin1I HgaI Ama87I Eco64I
gggaagccggcggctcgggcggcgtctgggttttttgccccccacaacccccggggagccacccagacggcaccg base pairs
cccttcggccgccgagcccgccgcagacccaaaaaacggggggtgttgggggcccctcggtgggtctgccgtggc 63301 to 63375
XmaI BssAI NaeI BsoBI AcyI BcoI XmaI BanI
 SmaI NgoMI Ama87I BbiII Cfr9I SmaI AccB1I
BsoBI NgoAIV Eco88I BsaHI AvaI BsoBI

 NgoAIV Eco88I NgoAIV
 NgoMI DraII BsoBI
 MroNI Cfr10I DdeI BsaWI BbvI EcoO109I MroNI
ccgccttgccgccggcagaacttctacaacccccacctcgctcagaccggaacgcagccaaaggccctcgggccg base pairs
ggcggaacggcggccgtcttgaagatgttgggggtggagcgagtctggccttgcgtcggtttccgggagcccggc 63376 to 63450
 BssAI NaeI BstDEI Bst71I Ama87I BsrFI
 BsrFI BcoI BssAI
 Bse118I AvaI NgoMI

 CelII HaeII BstSNI Tsp509I BsaHI
Bse118I BlpI MaeII Csp6I TspEI BbiII
 Cfr10I BstDEI Eco105I BstSFI Sse9I Hin1I
gctcagcgccatacgtactacagcgagtgcgacgaatttcgatttatcgccccgcgttcgctggacgaggacgcc base pairs
cgagtcgcggtatgcatgatgtcgctcacgctgcttaaagctaaatagcggggcgcaagcgacctgctcctgcgg 63451 to 63525
 DdeI Bsp143II SnaBI AcsI TthHB8I Msp17I
 NaeI Bsp1720I BsaAI AfaI ApoI TaqI Hsp92I
 Bpu1102I BstH2I RsaI SfcI AcyI

 KspI SacII Eco47I XmaIII BsePI BstDEI
 DsaI SstII Bst71I HgiEI CfrI BsiEI BssHII Bse21I RsaI
 BstDSI Cfr42I SinI EaeI Eco52I AscI Eco81I Csp6I BsmFI
cccgcggagcagcgcaccggggtccacgacggccgcctccggcgcgcccctaaggtgtactgcgggggggacgag base pairs
gggcgcctcgtcgcgtggccccaggtgctgccggcggaggccgcgcggggattccacatgacgccccccctgctc 63526 to 63600
 NspBII BbvI Bme18I BstZI BstMCI DdeI Bsu36I
 MspA1I AvaII EagI Bsh1285I CvnI AfaI
 HgaI Sfr303I EclXI BsaOI AocI

 MaeII BmyI BglI Eco47I
 BbiII AatII SduI ApaI HgiEI
 Hin1I AcyI Bsp120I BanII EaeI HgaI BglI SinI NlaIII
cgcgacgtcctccgcgtgggcccggagggcttctggccgcgtcgcttgcgcctgtggggcggtgcggaccatgcc base pairs
gcgctgcaggaggcgcacccgggcctcccgaagaccggcgcagcgaacgcggacaccccgccacgcctggtacgg 63601 to 63675
 Msp17I PspOMI FriOI CfrI Bme18I Hsp92II
 Hsp92I Bsp1286I AvaII
 BsaHI Eco24I

 Eco88I MboII PmlI Csp6I Bst2UI AspHI BsiHKAI Csp6I
 Ama87I MaeIII BbsI AflIII AfaI FokI GsuI Bbv12I PmaCI AfaI
 BsoBI TthHB8I DrdI HphI BpuAI BsaAI RsaI BstOI SduI Alw21I BbrPI
cccgaggggttcgaccccaccgtcaccgtcttccacgtgtacgacatcctggagcacgtggaacacgcgtacagc base pairs
gggctccccaagctggggtggcagtggcagaaggtgcacatgctgtaggacctcgtgcaccttgtgcgcatgtcg 63676 to 63750
 TaqI Tsp45I Bbv16II PmaCI EcoRII MvaI BmyI Eco72I AflIII
 BcoI BpiI Eco72I BstF5I Bsp1286I BsaAI MluI
 AvaI MaeII BbrPI BstNI BpmI MaeII PmlI RsaI

 BsePI BsaHI Eco47I
 BssHII BbiII HgiEI
 PaeI NspI AluI BsiI Hin1I SinI
atgcgcgccgcccagctccacgagcgatttatggacgccatcacgcccgccgggaccgtcatcacgcttctgggt base pairs
tacgcgcggcgggtcgaggtgctcgctaaatacctgcggtagtgcgggcggccctggcagtagtgcgaagaccca 63751 to 63825
 NlaIII BssSI Msp17I Bme18I
 Hsp92II Hsp92I AvaII
 BbuI SphI AcyI HgaI BsmFI

 ScaI
 RsaI
 HgaI MaeII Csp6I NlaIII
ctgacccccgaaggccatcgcgtcgccgttcacgtctacggcacgcggcagtacttttacatgaacaaggcggag base pairs
gactgggggcttccggtagcgcagcggcaagtgcagatgccgtgcgccgtcatgaaaatgtacttgttccgcctc 63826 to 63900
 AccI Acc113I Hsp92II
 AfaI
 Eco255I

 Bsp143I BshNI PstI Sau3AI BstNI
 MboI DpnI AccB1I BmyI NdeII HaeII MvaI
 DpnII AclWI BstSFI SduI DpnII EcoRII Bst2UI HinfI
gtggatcggcacctgcagtgccgtgccccgcgcgatctctgcgagcgcctggcggcggccctgcgcgagtcgccg base pairs
cacctagccgtggacgtcacggcacggggcgcgctagagacgctcgcggaccgccgccgggacgcgctcagcggc 63901 to 63975
 NdeII AlwI SfcI TspRI Bsp1286I Kzo9I Bsp143II PleI
 Sau3AI BanI BspMI MboI DpnI BstH2I
 Kzo9I Eco64I Bsp143I BstOI

 BsaHI KspI SacII KspI SacII TthHB8I AfaI
 BbiII NspBII SfaNI Sfr303I AvaII Csp6I
 Hin1I BstDSI Cfr42I MspA1I Bme18I BssHII MaeII
ggggcgtcgttccgcggcatctccgcggaccacttcgaggcggaggtggtggagcgcgccgacgtgtactattac base pairs
ccccgcagcaaggcgccgtagaggcgcctggtgaagctccgcctccaccacctcgcgcggctgcacatgataatg 63976 to 64050
 Msp17I DsaI SstII BstDSI Cfr42I Eco47I BsePI AflIII
 Hsp92I MspA1I DsaI SstII HgiEI RsaI
 AcyI HgaI Sfr303I NspBII SinI TaqI

 BpiI
 AfaI Bbv16II
 Csp6I HgaI BssHII
gaaacgcgcccgaccctgtactaccgcgtcttcgtgcgaagcgggcgcgcgctggcctacctgtgcgacaacttt base pairs
ctttgcgcgggctgggacatgatggcgcagaagcacgcttcgcccgcgcgcgaccggatggacacgctgttgaaa 64051 to 64125
 RsaI BpuAI BsePI
 BbsI
 MboII

 Bsp143I BsaHI TaqI Msp17I MvaI Eco88I MaeIII
 MboI DpnI AfaI BbiII AccI Hin1I AcyI BstOI PspAI PspALI
 DpnII Csp6I Hin1I TthHB8I Hsp92I EcoRII Ama87I Tsp45I
tgccccgcgatcaggaagtacgaggggggcgtcgacgccaccacccggtttatcctggacaacccggggtttgtc base pairs
acggggcgctagtccttcatgctccccccgcagctgcggtggtgggccaaataggacctgttgggccccaaacag 64126 to 64200
 NdeII RsaI Msp17I HgaI BbiII BstNI BcoI XmaI
 Sau3AI Hsp92I HincII BsaHI Bst2UI Cfr9I SmaI
 Kzo9I AcyI SalI HindII HgaI AvaI BsoBI

 BshNI RsaI XmaIII BstMCI SstII NgoMI
 Asp718I CfrI BsiEI BsaOI Cfr42I Bse118I
 Eco64I AfaI EaeI Eco52I MspA1I BssAI NaeI
accttcggctggtaccgcctcaagcccggccgcgggaacgcgccggcccaaccgcgccccccgacggcgttcgga base pairs
tggaagccgaccatggcggagttcgggccggcgcccttgcgcggccgggttggcgcggggggctgccgcaagcct 64201 to 64275
 HphI BanI Csp6I BstZI BstDSI KspI SacII NgoAIV
 Acc65I KpnI EagI Bsh1285I Sfr303I BsrFI
 AccB1I EclXI DsaI NspBII MroNI Cfr10I

 Eco88I Msp17I TthHB8I MvaI NgoAIV
 XhoI PaeR7I MaeII TaqI BstOI TaqI Tsp45I NgoMI
 Sfr274I Hin1I AcyI Tru9I BsgI EcoRII CfrI NlaIII MroNI
acctcgagcgacgtcgagtttaactgcacggcggacaacctggccgtcgagggggccatgtgtgacctgccggcc base pairs
tggagctcgctgcagctcaaattgacgtgccgcctgttggaccggcagctcccccggtacacactggacggccgg 64276 to 64350
 Ama87I TaqI Hsp92I MseI BstNI TthHB8I Hsp92II BssAI
 BcoI BsoBI BbiII AatII Bst2UI MaeIII BsrFI
 AvaI TthHB8I BsaHI Tru1I EaeI Bse118I

 TaqI BsaOI
NaeI TthHB8I BsaMI Bsh1285I
Cfr10I NlaIII Eco32I BsmI AluI BsaWI
tacaagctcatgtgcttcgatatcgaatgcaaggccgggggggaggacgagctggcctttccggtcgcggaacgc base pairs
atgttcgagtacacgaagctatagcttacgttccggccccccctcctgctcgaccggaaaggccagcgccttgcg 64351 to 64425
BspMI AluI Hsp92II EcoRV Mva1269I BsiEI
 TaqI TthHB8I BstMCI


 BpiI BstX2I XhoII Eco88I SduI Alw21I
 Bbv16II BstYI Bsp143I XhoI PaeR7I Bbv12I
 MboII AspI BstF5I Sau3AI Sfr274I Bsp1286I
ccggaagacctcgtcatccagatctcctgtctgctctacgacctgtccaccaccgccctcgagcacatcctcctg base pairs
ggccttctggagcagtaggtctagaggacagacgagatgctggacaggtggtggcgggagctcgtgtaggaggac 64426 to 64500
 BpuAI FokI MboI Kzo9I Ama87I TaqI BmyI
 BbsI AtsI DpnII MflI DpnI BcoI BsoBI AspHI
 Tth111I NdeII BglII AvaI TthHB8I BsiHKAI

 MboI Bsp143I BcoI Sau3AI MvaI EcoO109I Bse118I
 BstX2I XhoII Ama87I BstDEI Kzo9I BstOI MroNI Cfr10I
BstF5I BstYI BamHI AlwI BsoBI BsmFI DpnII EcoRII BpmI BsrFI
ttttcgctcggatcctgcgacctccccgagtcccacctcagcgatctcgcctccaggggcctgccggcccccgtc base pairs
aaaagcgagcctaggacgctggaggggctcagggtggagtcgctagagcggaggtccccggacggccgggggcag 64501 to 64575
FokI DpnII MflI DpnI AvaI PleI DdeI NdeII BstNI DraII NgoAIV
 NdeII BstI AclWI HinfI MboI DpnI Bst2UI BssAI NaeI
 Sau3AI Kzo9I Eco88I Bsp143I GsuI NgoMI

 MvaI BpmI ApoI TaqI BsoBI
 BstOI EcoRI Bst71I NlaIII AfaI Eco88I
 EcoRII GsuI Sse9I SfaNI BspHI PshAI Csp6I Ama87I
gtcctggagtttgacagcgaattcgagatgctgctggccttcatgaccttcgtcaagcagtacggccccgagttc base pairs
caggacctcaaactgtcgcttaagctctacgacgaccggaagtactggaagcagttcgtcatgccggggctcaag 64576 to 64650
 BstNI AcsI TthHB8I BbvI RcaI RsaI BcoI
 Bst2UI TspEI Hsp92II AvaI
 Tsp509I

 BsrI MboI Bsp143I DraII
 AfaI BseNI BstX2I Kzo9I EcoO1
Tsp45I Csp6I TthHB8I AluI BstYI MflI SinI
gtgaccgggtacaacatcatcaacttcgactggcccttcgtcctgaccaagctgacggagatctacaaggtcccg base pairs
cactggcccatgttgtagtagttgaagctgaccgggaagcaggactggttcgactgcctctagatgttccagggc 64651 to 64725
MaeIII RsaI TaqI BsrSI DpnII BglII Bme18I
 Bse1I NdeII XhoII PpuMI
 Sau3AI DpnI HgiEI

 TthHB8I AfaI CfrI BsiEI
 09I AccBSI EaeI Eco52I CfrI
AvaII TaqI RsaI NlaIII XmaIII BsaOI BsmFI
ctcgacgggtacgggcgcatgaacggccggggtgtgttccgcgtgtgggacatcggccagagccactttcagaag base pairs
gagctgcccatgcccgcgtacttgccggccccacacaaggcgcacaccctgtagccggtctcggtgaaagtcttc 64726 to 64800
 BsmFI BsrBI Hsp92II EclXI EaeI
 Psp5II BstD102I BstZI BstMCI
 Eco47I Csp6I EagI Bsh1285I

 MboI DpnI AflIII RsaI
 DpnII HphI MslI Csp6I AspI
 BbvI Bsp143I HphI BstF5I TthHB8I NspI SfaNI HphI
cgcagcaagatcaaggtgaacgggatggtgaacatcgacatgtacggcatcatcaccgacaaggtcaaactctcc base pairs
gcgtcgttctagttccacttgccctaccacttgtagctgtacatgccgtagtagtggctgttccagtttgagagg 64801 to 64875
 Bst71I Kzo9I FokI TaqI NlaIII Tth111I
 NdeII BspLU11I AfaI AtsI
 Sau3AI Hsp92II

 NdeII Kzo9I AluI
 BpmI DpnII XhoII AlwI
 AluI AluI MboII Sau3AI DdeI BstF5I
agctacaagctgaacgccgtcgccgaggccgtcttgaaggacaagaagaaggatctgagctaccgcgacatcccc base pairs
tcgatgttcgacttgcggcagcggctccggcagaacttcctgttcttcttcctagactcgatggcgctgtagggg 64876 to 64950
 GsuI BstYI MflI AclWI FokI
 BstX2I DpnI BstDEI
 MboI Bsp143I

 BmyI BbvI Bsp143I
 SduI ApaI MboI DpnI
 Bsp120I BanII DpnII HphI BsgI PleI BbvI
gcctactacgcctccgggcccgcgcagcgcggggtgatcggcgagtattgtgtgcaggactcgctgctggtcggg base pairs
cggatgatgcggaggcccgggcgcgtcgcgccccactagccgctcataacacacgtcctgagcgacgaccagccc 64951 to 65025
 PspOMI FriOI NdeII HinfI Bst71I
 Bsp1286I Sau3AI
 Eco24I Bst71I Kzo9I

 BbvI MvaI GsuI Bst2UI
 NspBII BstOI EcoRII
 PvuII MboII EcoRII AluI BssHII BglI SfaNI HphI
cagctgttcttcaagtttctgccgcacctggagctttccgccgtcgcgcgcctggcgggcatcaacatcacccgc base pairs
gtcgacaagaagttcaaagacggcgtggacctcgaaaggcggcagcgcgcggaccgcccgtagttgtagtgggcg 65026 to 65100
 AluI BstNI BsePI BstNI
 MspA1I Bst2UI BstOI
 Bst71I BpmI MvaI

 MboI Bsp143I BbsI Eco72I
 BstX2I DpnI BpuAI PmlI
 EaeI BstYI MflI AlwI MboII BbrPI BglI Asp700I BstF5I
accatctacgacggccagcagatccgcgtcttcacgtgcctcctgcgccttgcgggccagaagggcttcatcctg base pairs
tggtagatgctgccggtcgtctaggcgcagaagtgcacggaggacgcggaacgcccggtcttcccgaagtaggac 65101 to 65175
 CfrI DpnII XhoII HgaI MaeII XmnI FokI
 NdeII Kzo9I Bbv16II PmaCI
 Sau3AI AclWI BpiI BsaAI

 MvaI AccB1I NarI HaeII EaeI EclXI Ama87I
 BstOI TthHB8I KasI BbiII EheI HaeII EagI BstMCI AvaI
 EcoRII EcoO109I Eco64I BsaHI BbeI BstH2I Eco52I Eco88I
ccggacacccaggggcggtttcggggcctcgacaaggaggcgcccaagcgcccggccgtgcctcggggggaaggg base pairs
ggcctgtgggtccccgccaaagccccggagctgttcctccgcgggttcgcgggccggcacggagccccccttccc 65176 to 65250
 BstNI DraII EcoNI BanI Msp17I Bsp143II CfrI Bsh1285I BsoBI
 Bst2UI TaqI BshNI Hsp92I Bsp143II XmaIII BsaOI
 Hin1I AcyI BstH2I BstZI BsiEI BcoI

 EagI BsrBI BstMCI
 BstZI BstD102I
 EaeI Eco52I BsaOI BsmFI BsmFI BsmFI
gagcggccgggggacgggaacggggacgaggataaggacgacgacgaggacggggacgaggacggggacgagcgc base pairs
ctcgccggccccctgcccttgcccctgctcctattcctgctgctgctcctgcccctgctcctgcccctgctcgcg 65251 to 65325
 EclXI BsiEI
 CfrI AccBSI BsmFI
 XmaIII Bsh1285I

 NgoMI MaeII Acc65I EcoRII EcoO109I AvaI BmyI PspALI
 BsePI Eco31I Bse118I BanI Csp6I BstOI Ama87I BsoBI BanII Bme18I
 BseRI Alw26I NgoAIV Eco64I AfaI MvaI DraII Eco88I FriOI SinI
gaggaggtcgcgcgcgagaccgggggccggcacgttgggtaccagggggcccgggtcctcgaccccacctccggg base pairs
ctcctccagcgcgcgctctggcccccggccgtgcaacccatggtcccccgggcccaggagctggggtggaggccc 65326 to 65400
 BssHII BsmAI MroNI Cfr10I AccB1I KpnI Bsp120I PspAI SduI ApaI
 BsaI BssAI NaeI Asp718I BstNI PspOMI Cfr9I Bsp1286I PpuMI
 BsrFI BshNI RsaI Bst2UI BcoI XmaI Eco24I SmaI

 EcoO109I TthHB8I BstOI
 TthHB8I TaqI AfaI BstF5I
 AvaII MaeII HindII Csp6I SfaNI Bst2UI
tttcacgtcgaccccgtggtggtgtttgactttgccagcctgtaccccagcatcatccaggcccacaacctgtgc base pairs
aaagtgcagctggggcaccaccacaaactgaaacggtcggacatggggtcgtagtaggtccgggtgttggacacg 65401 to 65475
HgiEI Eco47I AccI DsaI RsaI EcoRII MvaI
 Psp5II SalI BstDSI BstNI
 DraII TaqI HincII FokI

 BsoBI MvaI BpmI RsrII Bst2UI Kzo9I TaqI
 RsaI Eco88I BstOI SinI Eco47I BstNI Sau3AI TthHB8I
 Eco57I Ama87I EcoRII GsuI CspI BsmFI DpnII GsuI
ttcagtacgctctccctgcggcccgaggccgtcgcgcacctggaggcggaccgggactacctggagatcgaggtg base pairs
aagtcatgcgagagggacgccgggctccggcagcgcgtggacctccgcctggccctgatggacctctagctccac 65476 to 65550
 Csp6I BcoI BstNI CpoI AvaII EcoRII NdeII DpnI
 AfaI AvaI Bst2UI Bme18I BstOI Bsp143I
 HgiEI MvaI MboI BpmI

 Pfl23II Bsp1720I EaeI
 BsaAI RsaI CelII FokI Bst71I Bse1I
 MboII MaeII SunI Bpu1102I BstF5I BsrSI
gggggccgacggctgttcttcgtgaaggcccacgtacgcgagagcctgctgagcatcctgctgcgcgactggctg base pairs
cccccggctgccgacaagaagcacttccgggtgcatgcgctctcggacgactcgtaggacgacgcgctgaccgac 65551 to 65625
 SplI Csp6I DdeI SfaNI BbvI BseNI
 PspLI AfaI BlpI BsrI
 BsiWI BstDEI CfrI

 Hsp92II MboI Bsp143I BstYI Sau3AI Kzo9I BmyI Ama87I
 NlaIII BstX2I DpnI BsrBI NdeII XhoII Bsp1286I Eco88I BseRI
 MluNI BstYI MflI AlwI BstX2I MflI AlwI Bbv12I AvaI TthHB8I
gccatgcgaaagcagatccgctcgcggatcccccagagcacccccgaggaggccgtcctcctcgacaagcaacag base pairs
cggtacgctttcgtctaggcgagcgcctagggggtctcgtgggggctcctccggcaggaggagctgttcgttgtc 65626 to 65700
 BalI DpnII XhoII AccBSI MboI BstI SduI BsiHKAI BseRI TaqI
 MscI NdeII Kzo9I BstD102I BamHI DpnI AspHI BcoI
 Sau3AI AclWI DpnII Bsp143I AclWI Alw21I BsoBI

 MboII
 AfaI Bst71I BbsI
 MslI Csp6I HphI BsgI BpuAI MaeII
gccgccatcaaggtggtgtgcaactcggtgtacgggttcaccggggtgcagcacggtcttctgccctgcctgcac base pairs
cggcggtagttccaccacacgttgagccacatgcccaagtggccccacgtcgtgccagaagacgggacggacgtg 65701 to 65775
 BstXI RsaI MslI BbvI Bbv16II
 BpiI


PmaCI XmaIII PspLI MaeII Bsp1286I
BsaAI CfrI BsiEI NruI BsePI BsiWI BsaAI AspHI BsePI
 BbrPI Tsp45I EaeI Eco52I SfaNI Bsp68I SplI RsaI ApaLI Alw21I
gtggccgccaccgtgacgaccatcggccgcgagatgctcctcgcgacgcgcgcgtacgtgcacgcgcgctgggcg base pairs
caccggcggtggcactgctggtagccggcgctctacgaggagcgctgcgcgcgcatgcacgtgcgcgcgacccgc 65776 to 65850
Eco72I MaeIII BstZI BstMCI BseRI BssHII Csp6I Alw44I Bbv12I
PmlI EaeI EagI Bsh1285I HgaI SunI AfaI SduI BsiHKAI
BsgI CfrI EclXI BsaOI Pfl23II VneI BmyI BssHII

 MboI Kzo9I AlwNI BsaWI CfrI MroNI Bse118I NlaIII SphI RsrII NlaIII
 DpnII DpnI MspA1I MroI Bsp13I EclXI BsrFI BstMCI BsePI HgiEI Csp6I
 TthHB8I AluI BbvI BspEI BsiMI XmaIII NgoAIV BsaOI NspI Bme18I RsaI
gagttcgatcagctgctggccgactttccggaggcggccggcatgcgcgcccccggtccgtactccatgcgcatc base pairs
ctcaagctagtcgacgaccggctgaaaggcctccgccggccgtacgcgcgggggccaggcatgaggtacgcgtag 65851 to 65925
 TaqI Bsp143I Bst71I AccIII EaeI Eco52I BsiEI NaeI Hsp92II CspI AfaI
 NdeII PvuII EaeI Kpn2I BstZI NgoMI Bsh1285I BssHII CpoI AvaII Hsp92II
 Sau3AI NspBII CfrI BseAI EagI BssAI Cfr10I PaeI BbuI SinI Eco47I

 KspI SacII EclXI DsaI KspI EcoRII MscI
 SfaNI NspBII BstZI BstDSI MspA1I BstNI CfrI
 AviII BsmFI PleI BstDSI Cfr42I XmaIII BstMCI SstII Bst2UI
atctacggggacacggactccattttcgttttgtgccgcggcctcacggccgcgggcctggtggccatgggcgac base pairs
tagatgcccctgtgcctgaggtaaaagcaaaacacggcgccggagtgccggcgcccggaccaccggtacccgctg 65926 to 66000
 Acc16I HinfI DsaI SstII EaeI Eco52I NspBII SacII EaeI
 MspA1I CfrI BsiEI BsaOI Cfr42I MvaI
FspI Sfr303I EagI Bsh1285I Sfr303I BstOI

 BssT1I NlaIII Bsp143I XhoI PaeR7I
 StyI DsaI MboI DpnI Eco88I
 Eco130I Bsp19I BssHII DpnII AluI AvaI MaeII HphI
aagatggcgagccacatctcgcgcgcgctgttcctccccccgatcaagctcgagtgcgaaaaaacgttcaccaag base pairs
ttctaccgctcggtgtagagcgcgcgcgacaaggaggggggctagttcgagctcacgcttttttgcaagtggttc 66001 to 66075
 ErhI BstDSI BsePI NdeII Sfr274I TaqI Psp1406I
 BalI NcoI Hsp92II Sau3AI Ama87I TthHB8I
MluNI EcoT14I Kzo9I BcoI BsoBI

 BsaHI SfaNI MboI Bsp143I
 Bst71I AfaI BbiII BsrBRI BstX2I
 BbvI Csp6I Hin1I MamI BstYI
ctgctgctcatcgccaagaaaaagtacatcggcgtcatctgcgggggcaagatgctcatcaagggcgtggatctg base pairs
gacgacgagtagcggttctttttcatgtagccgcagtagacgcccccgttctacgagtagttcccgcacctagac 66076 to 66150
AluI RsaI Msp17I BsaBI DpnII
 Hsp92I Bsh1365I NdeII
 AcyI HgaI Bse8I Sau3AI

 MvaI EcoO109I FriOI Bst2UI BspMI
 DpnI AviII BstOI BpmI Bsp1286I BstNI AccI
MflI AlwI EcoRII PspOMI BmyI ApaI MvaI HincII
gtgcgcaaaaacaactgcgcgtttatcaaccgcacctccagggccctggtcgacctgctgttttacgacgatacc base pairs
cacgcgtttttgttgacgcgcaaatagttggcgtggaggtcccgggaccagctggacgacaaaatgctgctatgg 66151 to 66225
XhoII FspI BstNI GsuI SduI BanII SalI HindII
Kzo9I Acc16I Bst2UI DraII Eco24I BstOI TaqI
 AclWI Bsp120I EcoRII BstXI TthHB8I

 BsaWI CfrI EclXI BsiEI AvaI
 MroI Bsp13I XmaIII Bsh1285I BstH2I EcoNI BsmFI
 BspEI BsiMI EagI BsrBI Bsp143II BseRI Ama87I SfcI
gtatccggagcggccgccgcgttagccgagcgccccgcagaggagtggctggcgcgacccctgcccgagggactg base pairs
cataggcctcgccggcggcgcaatcggctcgcggggcgtctcctcaccgaccgcgctggggacgggctccctgac 66226 to 66300
 AccIII EaeI NotI BstD102I HaeII BcoI BstSFI
 Kpn2I BstZI Eco52I BstMCI Eco88I
 BseAI CciNI AccBSI BsaOI BsoBI

 Hsp92I BstOI AspI
 Msp17I BstF5I
 AccI AcyI SfaNI BsmFI Bst2UI
caggcgttcggggccgtcctcgtagacgcccatcggcgcatcaccgacccggagagggacatccaggactttgtc base pairs
gtccgcaagccccggcaggagcatctgcgggtagccgcgtagtggctgggcctctccctgtaggtcctgaaacag 66301 to 66375
 Hin1I HgaI HphI EcoRII MvaI
 PstI BbiII BstNI Tth111I
 BsaHI FokI AtsI

 BstH2I
 AfaI Bsp143II
 HphI DdeI BssHII RsaI XcmI BstOI
ctcaccgccgaactgagcagacacccgcgcgcgtacaccaacaagcgcctggcccacctgacggtgtattacaag base pairs
gagtggcggcttgactcgtctgtgggcgcgcgcatgtggttgttcgcggaccgggtggactgccacataatgttc 66376 to 66450
 BstDEI BsePI Csp6I EcoRII Bst2UI
 HaeII MvaI
 BstNI

 EcoO109I Eco47I MboI MflI DpnI BsiWI BsaAI Kzo9I
 PpuMI Psp5II HgiEI DpnII BamHI AlwI SunI MaeII Bsp143I
AluI NlaIII BssHII SinI AvaII SinI BstYI Bsp143I SplI Csp6I MboI DpnI
ctcatggcccgccgcgcgcaggtcccgtccatcaaggaccggatcccgtacgtgatcgtggcccagacccgcgag base pairs
gagtaccgggcggcgcgcgtccagggcaggtagttcctggcctagggcatgcactagcaccgggtctgggcgctc 66451 to 66525
 Hsp92II BsePI Bme18I BsmFI Bme18I NdeII BstI AclWI RsaI DpnII
 HgiEI Eco47I AvaII BstX2I XhoII PspLI AfaI Sau3AI
 DraII BspMI BsaWI Sau3AI Kzo9I Pfl23II NdeII

 Esp3I CfrI BbiII MvaI Eco24I
 BsmAI BstMCI BfaI AcyI BstOI Bsp1286I
 BseRI Bsh1285I AluI Hin1I HgaI BstNI BsmFI BanII
gtagaggagacggtcgcgcggctggccgccctccgcgagctagacgccgccgccccaggggacgagcccgccccc base pairs
catctcctctgccagcgcgccgaccggcgggaggcgctcgatctgcggcggcggggtcccctgctcgggcggggg 66526 to 66600
 Alw26I BsaOI MaeI BsaHI EcoRII SduI
 BsmBI EaeI Msp17I Bst2UI BmyI
 BsiEI Hsp92I FriOI

 BcoI AvaI Hin1I Alw26I Esp3I SphI AvaI SmaI
 BstH2I Eco88I BbiII BsmAI HgaI NspI Cfr9I HgaI
 NspBII EaeI Bsp143II BsoBI Hsp92I AspI PaeI Ama87I BsoBI
ccagcggccctgccctccccggccaagcgcccccgggagacgccgtcgcatgccgaccccccgggaggcgcgtcc base pairs
ggtcgccgggacgggaggggccggttcgcgggggccctctgcggcagcgtacggctggggggccctccgcgcagg 66601 to 66675
 MspA1I CfrI HaeII Cfr9I SmaI AcyI AtsI NlaIII PspAI PspALI
 Ama87I PspALI BsaHI BsmBI Hsp92II Eco88I
 PspAI XmaI Msp17I Tth111I BbuI BcoI XmaI

 MboI Bsp143I Cfr9I BsoBI Ama87I BsoBI
 Bst71I BstX2I XhoII PspAI XmaI AfaI AvaI AccBSI
 AluI AluI BstYI BamHI BcoI AvaI SmaI BcoI XmaI BstD1
aagccccgcaagctgctggtgtccgagctggcggaggatcccgggtacgccatcgcccggggcgttccgctcaac base pairs
ttcggggcgttcgacgaccacaggctcgaccgcctcctagggcccatgcggtagcgggccccgcaaggcgagttg 66676 to 66750
 BbvI DpnII MflI DpnI AclWI Csp6I Cfr9I SmaI
 NdeII BstI Ama87I AlwI RsaI Eco88I BsrBI
 Sau3AI Kzo9I Eco88I PspALI PspAI PspALI

 Bsp143I
 MaeII MboI
 02I BspMI Tsp45I EcoO109I DpnII
acggactattacttctcgcacctgctgggggcggcctgcgtgacgttcaaggccctgtttggaaataacgccaag base pairs
tgcctgataatgaagagcgtggacgacccccgccggacgcactgcaagttccgggacaaacctttattgcggttc 66751 to 66825
 MaeIII DraII NdeII
 Sau3AI
 Kzo9I

 BsoBI Esp3I
 DpnI MseI Eco88I BsmAI BshNI CfrI
 HphI PleI Ama87I Alw26I AccB1I MaeII DdeI
atcaccgagagtctgttaaagaggtttattcccgagacgtggcaccccccggacgacgtggccgcgcggctcagg base pairs
tagtggctctcagacaatttctccaaataagggctctgcaccgtggggggcctgctgcaccggcgcgccgagtcc 66826 to 66900
 HinfI Tru9I BcoI MaeII BanI EaeI BstDEI
 Tru1I AvaI BsmBI
 Eco64I

 KspI SacII Bse118I NgoMI Bsp143II
 NspBII BssAI NaeI NgoAIV BstH2I
 BstDSI Cfr42I NgoAIV MroNI Cfr10I TthHB8I
gccgcggggttcgggccggcgggggccggcgctacggcggaggaaactcgtcgaatgttgcatagagcctttgat base pairs
cggcgccccaagcccggccgcccccggccgcgatgccgcctcctttgagcagcttacaacgtatctcggaaacta 66901 to 66975
 DsaI SstII MroNI Cfr10I Bse118I TaqI
 MspA1I NgoMI BssAI NaeI
 Sfr303I BsrFI BsrFI HaeII

 BmyI TaqI XmaIII Tth111I Sse9I
 SduI TthHB8I CfrI BsiEI AspI BsaOI
 MaeI NlaIII FriOI AluI BsmFI EaeI Eco52I AtsI
actctagcatgagccccccgtcgaagctgatgtcccgcatcttgcaataaatgtctgcggccgacacggtcggaa base pairs
tgagatcgtactcggggggcagcttcgactacagggcgtagaacgttatttacagacgccggctgtgccagcctt 66976 to 67050
 BfaI Hsp92II BanII SfaNI BstZI BstMCI Bsh1285I
 Bsp1286I EagI Bsh1285I BstMCI
 Eco24I EclXI BsaOI BsiEI

 AspHI MaeII Bbv12I BsaAI
 NspBII SduI BsiHKAI Alw21I PmlI
AcsI Tsp509I HgaI BsiI Bbv12I SduI BsiHKAI PmaCI
tttccgcgtccgctggtttctctgcgttgcgtctgaccacgagcacaaacgtgctctgccacacgtgggcggcga base pairs
aaaggcgcaggcgaccaaagagacgcaacgcagactggtgctcgtgtttgcacgagacggtgtgcacccgccgct 67051 to 67125
 HgaI BssSI Alw21I BmyI AflIII BbrPI
 ApoI MspA1I Bsp1286I Bsp1286I Eco72I
 TspEI BmyI MslI AspHI MaeII

 BsrFI MboI Bsp143I BsiEI TaqI PvuI Cfr10I
 BssAI BmyI DpnII DpnI BanIII Bsh1285I BsrFI
 AgeI Cfr10I SduI SfaNI Sau3AI Bsp106I Bsu15I BsaOI BspMI
accggtagccggggcacgcggtcagcatccgatcgatgagccggtagtgcaggtgggccgacgtgccggggaaga base pairs
tggccatcggccccgtgcgccagtcgtaggctagctactcggccatcacgtccacccggctgcacggccccttct 67126 to 67200
 PinAI Bsp1286I BstF5I TthHB8I BspXI BseCI BssAI MaeII
 BsaWI NdeII BspCI Bsa29I BscI ClaI Bse118I
 Bse118I FokI Kzo9I BspDI Ple19I BstMCI BsgI

 SunI AfaI AvaII
 Csp6I NspI Pfl23II SinI
MboII RsaI NlaIII SplI RsaI HgiEI
tgacgtacagcatgtggcccccgtacgtggggtccgggtaaaaaagaaaccgggggtcgcacgccccccctccgc base pairs
actgcatgtcgtacaccgggggcatgcaccccaggcccattttttctttggcccccagcgtgcgggggggaggcg 67201 to 67275
 MaeII Hsp92II PspLI MaeII Eco47I
 AfaI BsiWI BsaAI
 Csp6I Bme18I

 Bsp143I ApaLI Alw21I Ama87I Bsp1286I Alw21I CfrI SinI AvaII BstOI
 MboI DpnI Bsp1286I AluI AvaI Bbv12I BanII EaeI MvaI HgiEI EcoRII BpmI
 DpnII AclWI AspHI Ecl136II AspHI SacI BsiHKAI Bst2UI DraII BstNI
gcaggatcgtgtgcacgaaaaagagctcgggctggccgagcgtatcggccaggaggtcctggaggggggtgctgt base pairs
cgtcctagcacacgtgctttttctcgagcccgaccggctcgcatagccggtcctccaggacctccccccacgaca 67276 to 67350
 NdeII AlwI SduI BsiHKAI Eco88I BmyI FriOI EaeI BstNI PpuMI Psp5II MvaI
 Sau3AI Alw44I Bbv12I EcoICRI SduI Psp124BI CfrI BstOI EcoO109I Bst2UI
 Kzo9I VneI BmyI BcoI BsoBI Eco24I SstI EcoRII Bme18I Eco47I GsuI

 EaeI MvaI Bbv12I MboI NdeII Bst71I
 BstMCI BstOI AspHI NdeII DpnI Bsp143I
 BsiEI EcoRII SduI BsiHKAI DpnII DpnI BsgI
ggcggtcggccagcacgaccagggaggccagaaaggtgcggtgctcaaagatcgtattgatctgctgcacgaagg base pairs
ccgccagccggtcgtgctggtccctccggtctttccacgccacgagtttctagcataactagacgacgtgcttcc 67351 to 67425
 Bsh1285I BstNI Bsp1286I Bsp143I Sau3AI
 BsaOI Bst2UI BmyI DpnII Kzo9I Kzo9I BbvI
 CfrI Alw21I Sau3AI MboI

 MvaI BalI Bsp143I
 BstOI EcoO109I MscI BsgI MboI
EcoRII BstF5I EaeI BbvI BbvI DpnII
ccaggatgagggcctcgcggctgacggtggccagccgcccgtcgcccgcgctgcacgcggggcagcagcccccga base pairs
ggtcctactcccggagcgccgactgccaccggtcggcgggcagcgggcgcgacgtgcgccccgtcgtcgggggct 67426 to 67500
 BstNI FokI CfrI Bst71I Bst71I NdeII
 Bst2UI DraII MluNI Sau3AI
 Kzo9I

 BstOI Eco88I Van91I Eco47I
 DpnI Bst2UI Ama87I BstDSI SinI EcoRII Eco57I
 AclWI NlaIII EaeI AccB7I AvaII MvaI
tccccaggtagtagcccatgcccgagagggtcaggcagttgtcggccacggtctggtccaggctgaaggggagcg base pairs
aggggtccatcatcgggtacgggctctcccagtccgtcaacagccggtgccagaccaggtccgacttcccctcgc 67501 to 67575
 AlwI MvaI Hsp92II CfrI PflMI HgiEI Bst2UI
 BstNI BcoI BsoBI DsaI Esp1396I BstNI
 EcoRII AvaI Bme18I BstOI

 MboII HphI Bse8I Sau3AI Hsp92I
 BbsI BstNI Bsp1286I BsrBRI Bsp143I Msp17I
 DrdI BpuAI Bst2UI MamI NdeII BseRI AcyI HgaI
acacgggggtcgtcttcaccaggggcacggagagcgagcgcacgatggcgatctcctcggagggcgtctgggcga base pairs
tgtgcccccagcagaagtggtccccgtgcctctcgctcgcgtgctaccgctagaggagcctcccgcagacccgct 67576 to 67650
 Bbv16II MvaI BsaBI MboI Hin1I
 BpiI BstOI BmyI Bsh1365I DpnI BbiII
 EcoRII SduI DpnII Kzo9I BsaHI

 MspA1I BssSI Bsp1286I BanII BpmI
 DsaI SstII BstH2I EcoICRI Eco24I BsiHKAI
 MboII KspI SacII Bsp143II Ecl136II SacI Alw21I
gggcggcgaagaagccgcggtagcgacggcgctcgtgcaggcagagctccagcctgcgcgcgtgcgacggcaggc base pairs
cccgccgcttcttcggcgccatcgctgccgcgagcacgtccgtctcgaggtcggacgcgcgcacgctgccgtccg 67651 to 67725
 BstDSI Cfr42I HaeII BsgI AluI BmyI FriOI GsuI
 NspBII BsiI SduI Bbv12I SstI BsePI
 Sfr303I AspHI Psp124BI BssHII


 BstH2I BstMCI
 Bsp143II BsiEI
tcttgcgggaggcccggcgctccacgccggggttcccggcggcggaaaagcgcgaccgccgccgggtcttgtcgc base pairs
agaacgccctccgggccgcgaggtgcggccccaagggccgccgccttttcgcgctggcggcggcccagaacagcg 67726 to 67800
 HaeII Bsh1285I
 BsaOI


XmaIII Bsp120I Eco88I BmyI PspALI Bbv12I BstOI
CfrI BsiEI PspOMI XmaI FriOI SrfI AfaI AspHI BstNI
EaeI Eco52I Ama87I BsoBI ApaI Csp6I SduI EcoRII
ggccgggcccgggccgggagccggagcgacggggggcgatgtcatacataggtacagagggtgtgctccagggac base pairs
ccggcccgggcccggccctcggcctcgctgccccccgctacagtatgtatccatgtctcccacacgaggtccctg 67801 to 67875
BstZI BstMCI PspAI SduI BanII RsaI Bsp1286I
EagI Bsh1285I Cfr9I Bsp1286I SmaI BmyI BsiHKAI
EclXI BsaOI BcoI AvaI Eco24I Alw21I Bst2UI

 NdeII TaqI MroNI Cfr10I BalI
 BpmI Bsp143I BbvI BsrFI MscI
 GsuI Sau3AI DdeI BssHII NgoAIV EaeI BstXI
aggagagagatcgagtgtcgtctgagcagcgcgccggcctcgcggacaaatgtggccagcgcggtgggcttcggc base pairs
tcctctctctagctcacagcagactcgtcgcgcggccggagcgcctgtttacaccggtcgcgccacccgaagccg 67876 to 67950
 MvaI DpnII TthHB8I BstDEI BsePI NgoMI CfrI
 BsmFI Kzo9I Bst71I Bse118I MluNI
 MboI DpnI BssAI NaeI

 Bst2UI SduI ApaI
 BstNI MaeII EcoO109I BanII
 SexAI Csp6I Bsp120I FriOI TthHB8I
acaaatacctggtacgtcttgaaggtgtagatgagggcccgcagggctatacagacccgcccctcgaactcgttg base pairs
tgtttatggaccatgcagaacttccacatctactcccgggcgtcccgatatgtctgggcggggagcttgagcaac 67951 to 68025
 EcoRII RsaI PspOMI Eco24I TaqI
 BstOI DraII BmyI BglI
 MvaI AfaI Bsp1286I

 Bst71I BalI EcoO109I
 BstSFI Bst71I MscI PpuMI Psp5II
 BcgI SfiI AluI PstI Bsp68I EaeI SinI AvaII
ccgcaggccaacttggccttgtgaagctgcagctcgtcgcgatggtcggcgcgggggtggccaaacaggacccag base pairs
ggcgtccggttgaaccggaacacttcgacgtcgagcagcgctaccagccgcgcccccaccggtttgtcctgggtc 68026 to 68100
 BglI SfcI AluI NruI CfrI Bme18I
 BbvI MluNI HgiEI Eco47I
 BbvI DraII EcoRII

 BstOI AccI Bsp143I NspI AvaI EcoO109I
 DrdI HindII MboI DpnI BspLU11I BcoI PspOMI
 Bst2UI TaqI MslI DpnII AclWI NlaIII MboII Bsp120I
gggtcgacttccatctccgtgatggcgcacatcggatcgcagaacatgtgcttgaagatggcctcggggcccgcg base pairs
cccagctgaaggtagaggcactaccgcgtgtagcctagcgtcttgtacacgaacttctaccggagccccgggcgc 68101 to 68175
BstNI MvaI HincII XcmI NdeII AlwI Hsp92II Ama87I SduI
 SalI Sau3AI AflIII Eco88I Bsp1286I
 TthHB8I Kzo9I BsoBI DraII

 BstDSI Sfr303I Eco88I BbvI BsoBI BcoI BsoBI Bme18I
Eco24I KspI SacII Bse118I PspAI BsmFI BcoI Bme18I Ama87I TthHB8I BsrFI
BmyI DsaI SstII BssAI Ama87I SmaI Ama87I AvaII Eco88I AluI Eco47I
gcccgaagcaggctcacgaaccggcccccgtccccgggctgcgcctcggggtccgcctcgagctggtccacgacc base pairs
cgggcttcgtccgagtgcttggccgggggcaggggcccgacgcggagccccaggcggagctcgaccaggtgctgg 68176 to 68250
 BanII MspA1I BsrFI BcoI XmaI Bst71I SinI Sfr274I SinI BssAI
 ApaI Cfr42I Cfr10I Cfr9I PspALI AvaI HgiEI XhoI PaeR7I HgiEI
FriOI NspBII AvaI BsoBI Eco88I Eco47I AvaI TaqI AvaII

Cfr10I EarI BsaHI Bse21I BbiII EaeI
 BsaOI Eam1104I BbiII DdeI Bsu36I HgaI
 Bsh1285I TthHB8I Hin1I Eco81I Msp17I CfrI
ggcactatgcagtcgaagaggctggtgttgttctccgagtagcggacgacggacgccctcaggcgtcgcatggcc base pairs
ccgtgatacgtcagcttctccgaccacaacaagaggctcatcgcctgctgcctgcgggagtccgcagcgtaccgg 68251 to 68325
 BstMCI TaqI MboII Msp17I CvnI Hin1I AcyI
Bse118I Ksp632I Hsp92I BstDEI Hsp92I NlaIII
BsiEI AcyI HgaI AocI BsaHI Hsp92II

 BalI BssAI XmaIII
 MluNI Bse1I BsaMI Cfr10I CfrI BsiEI
 BsrSI BsmI Bse118I EaeI Eco52I
agccagtaggcccgcaccagcaacagattgcacagcaggcattccccgccggtgcgcccgcgcccccggccgtgc base pairs
tcggtcatccgggcgtggtcgttgtctaacgtgtcgtccgtaaggggcggccacgcgggcgcgggggccggcacg 68326 to 68400
 MscI BseNI Mva1269I BstZI BstMCI
 BsrI SgrAI EagI Bsh1285I
 BsrFI EclXI BsaOI

 BalI EcoRII BstNI HgiEI BstOI FriOI
 CfrI Bsp120I FriOI Bst2UI BstNI BmyI Acc16I
 Eco57I MscI NspBII Bsp1286I BstOI Eco47I SduI FspI
ttcagcacggtggccatcagcgggcccaggtccaggtcgggctggggctggggctcggcgaactgcgcaaaacgc base pairs
aagtcgtgccaccggtagtcgcccgggtccaggtccagcccgaccccgaccccgagccgcttgacgcgttttgcg 68401 to 68475
 EaeI MspA1I BmyI ApaI SinI EcoRII Bsp1286I AviII
 MluNI PspOMI Eco24I MvaI AvaII MvaI Eco24I
 SduI BanII Bme18I Bst2UI BanII

 BsePI MspA1I MvaI KspI SacII BsaHI NspBII
 BssHII DsaI SstII EcoRII NspBII BbiII DsaI
 HgaI PaeI NspI KspI SacII BstNI PleI BstDSI Cfr42I Hin1I BstDSI
ggggccgcgtcgcgcatgcgcgccccgcggtgcgcttcccaggactcgctgaccgcggcgcggcgggcgtccgcg base pairs
ccccggcgcagcgcgtacgcgcggggcgccacgcgaagggtcctgagcgactggcgccgcgccgcccgcaggcgc 68476 to 68550
 NlaIII BstDSI Cfr42I BstOI DsaI SstII Msp17I MspA1I
 Hsp92II NspBII Bst2UI MspA1I Hsp92I KspI
 BbuI SphI Sfr303I HinfI Sfr303I AcyI HgaI

 BssHII EcoO109I ApaI MroNI Cfr10I Sau3AI SunI
 SstII Bsp120I FriOI BshNI BsrFI NdeII AlwI Pfl23II
 SacII BbvI SduI BanII Eco64I NgoAIV BbvI DpnII AclWI SplI
gcggcgcgcagccggggccccgactcccagacggcgggggtgccggcgagcagcagcaggatcaggtcggcgtac base pairs
cgccgcgcgtcggccccggggctgagggtctgccgcccccacggccgctcgtcgtcgtcctagtccagccgcatg 68551 to 68625
 BsePI Bst71I Bsp1286I PleI BanI NgoMI Bst71I Kzo9I PspLI
 Cfr42I PspOMI Eco24I AccB1I Bse118I MboI DpnI BsiWI
 Sfr303I DraII BmyI HinfI BssAI NaeI Bsp143I Csp6I

 AfaI
 BsmBI BstH2I
 RsaI Alw26I HphI Bsp143II TthHB8I BssHII
gcccacgtctccggctcacccccctgcgccagcgccccggcggcggcctcgaactccccgttgcgggcggcggcg base pairs
cgggtgcagaggccgagtggggggacgcggtcgcggggccgccgccggagcttgaggggcaacgcccgccgccgc 68626 to 68700
 MaeII BsmAI HaeII TaqI BsePI
 Esp3I BglI


 PvuII
 Bst71I Alw26I TaqI Bse1I
 BsgI NspBII BsgI BsrSI
cgcgtgcagcagctgtctccgcccccgcgcttgccctcggtgcagtcgagcaggcgggcgcagtccttccagttc base pairs
gcgcacgtcgtcgacagaggcgggggcgcgaacgggagccacgtcagctcgtccgcccgcgtcaggaaggtcaag 68701 to 68775
 BbvI MspA1I TthHB8I BseNI
 AlwNI BsmAI BsrI
 AluI

 BsoBI FriOI
 Eco88I BmyI
 HphI Ama87I SduI
atcagggcggtggtgagggagggttgcgttcccgagcccccgcccgcccccgcccccgccccgtcatcgcccccg base pairs
tagtcccgccaccactccctcccaacgcaagggctcgggggcgggcgggggcgggggcggggcagtagcgggggc 68776 to 68850
 BcoI Bsp1286I
 AvaI Eco24I
 BanII

 MvaI PpuMI Psp5II Ama87I BsoBI BanII ScaI BssT1I
 BstOI EcoO109I EcoO109I XmaI Eco24I SmaI RsaI ErhI
 EcoRII Bme18I Eco47I BcoI Eco88I FriOI Csp6I Eco130I
gaggccagggtcccgatgagggcccgggttgcggactgcgcgaggaaggaatagttggagtactgcaccttggcg base pairs
ctccggtcccagggctactcccgggcccaacgcctgacgcgctccttccttatcaacctcatgacgtggaaccgc 68851 to 68925
 BstNI HgiEI BsmFI DraII AvaI BmyI PspALI Acc113I EcoT14I
 Bst2UI DraII Bsp120I Cfr9I Bsp1286I AfaI StyI
 SinI AvaII PspOMI PspAI SduI ApaI Eco255I BsgI

KasI BbiII EheI PspAI BsoBI Hsp92I BstOI AccB1I NarI Bsp143II XmaI BanI BmyI
Eco64I BsaHI BstH2I AvaI Hin1I AcyI MvaI KasI BbiII EheI PspAI BsoBI BshNI
 AccB1I NarI Bsp143II XmaI BbiII EcoRII Eco64I BsaHI BstH2I AvaI SmaI SduI
gcgcccggggagggcgtcggcctgggttgcttctgggcgtggcgcccgggcaccccgccgtcggtccggaagcag base pairs
cgcgggcccctcccgcagccggacccaacgaagacccgcaccgcgggcccgtggggcggcagccaggccttcgtc 68926 to 69000
 Hin1I AcyI HaeII Eco88I Msp17I BstNI BanI Msp17I Ama87I BbeI PspALI Bsp1286I
BanI Msp17I Ama87I BbeI PspALI HgaI BshNI Hsp92I BcoI Cfr9I SrfI AccB1I
BshNI Hsp92I BcoI Cfr9I SmaI BsaHI Bst2UI Hin1I AcyI HaeII Eco88I Eco64I CpoI

 CspI BspEI BsiMI BsrFI
SinI AvaII BsaWI TspRI FokI
 Bme18I AccIII BbvI Bse118I BssHII PshAI
cagtggagaaagaaatgccggtggatgtcgttgatggtcagggcgaagcgcgcgaaggagccgacaagggtcgcc base pairs
gtcacctctttctttacggccacctacagcaactaccagtcccgcttcgcgcgcttcctcggctgttcccagcgg 69001 to 69075
 Eco47I BseAI BssAI BstF5I BsePI
 HgiEI MroI Bsp13I Cfr10I
 RsrII Kpn2I Bst71I

 Eco47I
 Acc16I HgiEI MaeII CfrI
 FspI SinI NlaIII TthHB8I MslI SfaNI
ttcttggtgcgcaggaagtggtggtccatgacgtagacgaactcgaaggcggccacgaagatgctcgcggcgcag base pairs
aagaaccacgcgtccttcaccaccaggtactgcatctgcttgagcttccgccggtgcttctacgagcgccgcgtc 69076 to 69150
 AviII Bme18I Hsp92II TaqI EaeI MboII
 AvaII AccI


 MvaI TspRI SseBI
 BssHII BstOI Esp1396I AatI
 TspRI EcoRII BstXI AflIII EaeI AccB7I Bse1I Pme55I
tggggcgcgcccaggcacttggcgcagaggaacgcgtaatcggccacccactggggcgagaggcggtaggcctgc base pairs
accccgcgcgggtccgtgaaccgcgtctccttgcgcattagccggtgggtgaccccgctctccgccatccggacg 69151 to 69225
 AscI BstNI MluI CfrI PflMI BseNI StuI
 BsePI Bst2UI Van91I BsrI Eco147I
 BsrSI

 RsaI Eco47I HincII BsaHI MspA1I
 BsrGI TthHB8I HgiEI AccI Msp17I NspBII
 SspBI AluI SinI SalI Hin1I HgaI Sfr3
ttgtacagctcgatggtgcggcagaccagacaggggcggtccagcgcgaaggtgtcgacggacgccgcggcgaag base pairs
aacatgtcgagctaccacgccgtctggtctgtccccgccaggtcgcgcttccacagctgcctgcggcgccgcttc 69226 to 69300
 Bsp1407I Bme18I TthHB8I Hsp92I KspI
 Csp6I TaqI AvaII TaqI BbiII BstDSI
 AfaI HindII AcyI DsaI SstII

 PspOMI BmyI ApaI KspI SacII
 Bsp120I Eco24I DsaI NspBII SinI
 03I DraII BanII PleI BstDSI BstDSI Cfr42I Eco47I
ggccccgtgtccaagagtccctctgccgtggggtctgcgggcgggccgcgggcggaccccggcccccgccccccc base pairs
ccggggcacaggttctcagggagacggcaccccagacgcccgcccggcgcccgcctggggccgggggcggggggg 69301 to 69375
Cfr42I Bsp1286I BsmFI DsaI SstII HgiEI
SacII SduI FriOI MspA1I Bme18I
 EcoO109I HinfI Sfr303I AvaII

 EagI Eco52I NspBII SacII MaeII
 CfrI EclXI DsaI Sfr303I Hsp92I BssHII
 BssHII EaeI XmaIII BsaOI SstII BbiII MaeII TthHB8I
gaagcctcgcgcgcggccccgcgcggccgcgggggggcgggcgcgacgtcgctctccacgtcctcgtcgagcgcg base pairs
cttcggagcgcgcgccggggcgcgccggcgcccccccgcccgcgctgcagcgagaggtgcaggagcagctcgcgc 69376 to 69450
 BsePI BstZI Bsh1285I KspI Hin1I AcyI TaqI
 CciNI BstDSI MspA1I Msp17I AatII BsePI
 NotI BsiEI BstMCI Cfr42I BsaHI

 PmaCI MvaI AluI BmyI FriOI EcoO109I
 BsaAI BstOI Bsp1286I BanII DraII
 MaeII MaeIII BstNI SduI Bbv12I Alw21I Tru1I
ctcgcgggcggcacgcctaccacgtgacaggccgccaggagctcggcgcacagggcctcgttaagagccagaagg base pairs
gagcgcccgccgtgcggatggtgcactgtccggcggtcctcgagccgcgtgtcccggagcaattctcggtcttcc 69451 to 69525
 Eco72I EcoRII Ecl136II SacI SstI Tru9I
 PmlI BbrPI Bst2UI AspHI Eco24I BsiHKAI
 Tsp45I EcoICRI Psp124BI MseI

 Bsp143I AlwNI MboII
 MboI DpnI TaqI NspBII Ksp632I
 DpnII TthHB8I HgaI PvuII HinfI Eam1104I
tcgggatcgaaggccacatacggacgctcgaacgcgccctccttccagctgctgcccggcgactcttcgcgcacg base pairs
agccctagcttccggtgtatgcctgcgagcttgcgcgggaggaaggtcgacgacgggccgctgagaagcgcgtgc 69526 to 69600
 NdeII TaqI TthHB8I AluI Bst71I PleI
 Sau3AI AlwI MspA1I EarI
 Kzo9I AclWI BbvI

 TaqI BanI PspAI BsoBI MaeII ErhI BstDSI BssAI BsiEI AccBSI
 BstH2I AccB1I AvaI Msp17I Eco130I Bsp19I Cfr10I BstMCI HgaI Psp14
 Bsp143II Ama87I SmaI Hsp92I StyI DsaI NlaIII TthHB8I BstD102I
gcggcgctcgacggcacccccggggcggacgtcgccatggccggtcgagcggggcgcacgcgtccgcgaacgtta base pairs
cgccgcgagctgccgtgggggccccgcctgcagcggtaccggccagctcgccccgcgtgcgcaggcgcttgcaat 69601 to 69675
 HaeII Eco64I Eco88I Hin1I AcyI NcoI EaeI BsrFI Bsh1285I MluI MaeII
 TthHB8I BcoI XmaI BbiII AatII EcoT14I Hsp92II TaqI BsrBI MaeIII
 BshNI Cfr9I PspALI BsaHI BssT1I CfrI Bse118I BsaOI AflIII

 MboI DpnI HgiEI AccB7I MvaI Bsp68I
06I DpnII AclWI SinI BsmFI Van91I XbaI BsePI BsmBI
 BsmFI Bsp143I BssHII BbvI Eco47I Esp1396I MaeI BssHII MaeII Alw26I
cgggacgcgatccccgactgcgcgctgcggtcccagaccctggaaagtctagacgcgcgctacgtctcgcgagac base pairs
gccctgcgctaggggctgacgcgcgacgccagggtctgggacctttcagatctgcgcgcgatgcagagcgctctg 69676 to 69750
 HgaI Kzo9I BsePI Bst71I EcoRII BstNI BfaI HgaI BsmAI
 NdeII AlwI Bme18I AlwNI BstOI Esp3I
 Sau3AI AvaII PflMI Bst2UI NruI

 CfrI DrdI BstMCI Hsp92I
 BsmBI BstZI Bsh1285I Msp17I
Alw26I BsmFI XmaIII BsaOI NlaIII MaeI BstDSI
ggcgcgggggacgcggccgtctggttcgaggacatgacccccgccgaactagaggttatattcccgaccacggac base pairs
ccgcgccccctgcgccggcagaccaagctcctgtactgggggcggcttgatctccaatataagggctggtgcctg 69751 to 69825
BsmAI EaeI EclXI TthHB8I Hsp92II BfaI DsaI Hin1I
 Esp3I HgaI Eco52I TaqI BbiII
 EagI BsiEI BsaHI

 Bst71I BsiWI
 MspA1I PspLI BstH2I
AcyI HgaI HgaI AlwNI MaeII Csp6I Bsp143II
gccaagctgaactacctctcgcggacgcagcggctggcctccctcctgacgtacgccgggcctataaaagcgccc base pairs
cggttcgacttgatggagagcgcctgcgtcgccgaccggagggaggactgcatgcggcccggatattttcgcggg 69826 to 69900
 AluI NspBII SplI RsaI HaeII
 BbvI Pfl23II
 SunI AfaI

 Bsp1286I
 VneI BmyI AluI
 DraIII SduI BsiHKAI
gacggccccgccgccccacatacgcaggacaccgcgtgcgtgcacggcgagctgctcgcccgaaagcgcgaacgg base pairs
ctgccggggcggcggggtgtatgcgtcctgtggcgcacgcacgtgccgctcgacgagcgggctttcgcgcttgcc 69901 to 69975
 Alw44I Alw21I BbvI
 ApaLI Bbv12I Bst71I
 AspHI

 PinAI EcoRII Bme18I DpnII MflI AclWI SduI
 Tru1I Bse118I MvaI BspMI MboI XhoII HgaI BshNI
 Tru9I BsrFI Bst2UI Eco47I NdeII DpnI AlwI BssHII Eco64I
ttcgcggcggtcattaaccggttcctggacctgcaccagatcctgcggggctgacgcgcgcttcggcggggcacc base pairs
aagcgccgccagtaattggccaaggacctggacgtggtctaggacgccccgactgcgcgcgaagccgccccgtgg 69976 to 70050
 MseI BsaWI BstNI HgiEI BstYI Bsp143I BsePI BanI
 AgeI Cfr10I SinI BsgI Sau3AI AlwNI AccB1I
 BssAI BstOI AvaII BstX2I Kzo9I Bsp1286I

 Cfr10I Bme18I Bsp68I
 BssAI BanI AvaII BstMCI
 BsrFI AccB1I BsmFI BsiEI
ggcaccgggaccgacttgttttacataacagtagggggtgggggaacgcgcacccttgcccggtcgcgatggcgg base pairs
ccgtggccctggctgaacaaaatgtattgtcatcccccacccccttgcgcgtgggaacgggccagcgctaccgcc 70051 to 70125
BmyI Eco64I HgiEI Bsh1285I
 Bse118I SinI BsaOI
 BshNI Eco47I NruI

 EagI Eco52I PspAI BsoBI TthHB8I
 CfrI EclXI BsaOI XmaI BsmFI BsaI
 BstF5I EaeI XmaIII BcoI AvaI AflIII Alw26I SfaNI
ggatggggaagccctacggcggccgcccgggggacgcgttcgagggtctcgttcagcgcatcaggctcattgttc base pairs
cctaccccttcgggatgccgccggcgggccccctgcgcaagctcccagagcaagtcgcgtagtccgagtaacaag 70126 to 70200
 FokI BstZI Bsh1285I Eco88I MluI Eco31I
 CciNI BstMCI Cfr9I SmaI HgaI BsmAI
 NotI BsiEI Ama87I PspALI TaqI

 DraII Eco24I Eco88I
 Bsp120I FriOI XhoI PaeR7I
 BbvI HinfI EcoO109I ApaI BstF5I Sfr274I
ccgccacgctgcgcggcgggggtggggagtcgggcccctactcgccatccaacccgccctcgagatgtgccttcc base pairs
ggcggtgcgacgcgccgcccccacccctcagcccggggatgagcggtaggttgggcgggagctctacacggaagg 70201 to 70275
 Bst71I PleI SduI BanII FokI Ama87I TaqI
 PspOMI BmyI BcoI BsoBI
 Bsp1286I AvaI TthHB8I

 BstDSI BstOI Bme18I SseBI Sau3AI Ple19I Csp6I
 Bse1I BstNI FokI Eco47I AatI MboI DpnI TaqI RsaI RcaI Bse1I
 BsrSI CfrI MvaI SinI Pme55I DpnII BspCI PvuI AfaI NlaIII BsrSI
agttccacggccaggatgggtccgacgaggccttcccgatcgagtacgtcctgcggctcatgaacgactgggccg base pairs
tcaaggtgccggtcctacccaggctgctccggaagggctagctcatgcaggacgccgagtacttgctgacccggc 70276 to 70350
 BseNI EcoRII BstF5I StuI NdeII TthHB8I BsaOI BspHI BseNI
 BsrI EaeI Bst2UI AvaII Eco147I Kzo9I BsiEI BstMCI Hsp92II BsrI
 DsaI XcmI HgiEI Bsp143I Bsh1285I MaeII

 Bse118I BsrFI
 BmyI BssAI Tru1I
 SduI BspMI BsgI Tru9I
atgtgccctgcaacccctacctgcgcgtgcagaacaccggcgtttcggtgctgtttcaggggttttttaaccggc base pairs
tacacgggacgttggggatggacgcgcacgtcttgtggccgcaaagccacgacaaagtccccaaaaaattggccg 70351 to 70425
 Bsp1286I SgrAI MseI Cfr10I
 BsrFI BssAI
 Cfr10I Bse118I

 KasI BbiII EheI BcoI BsoBI Sau3AI
 DsaI AccB1I NarI BstH2I Eco88I MboI TfiI BsmBI
 BstDSI Hin1I AcyI Ama87I AvaI DpnII DpnI MaeII BsgI Alw26I DrdI
cccacggcgccccggggggcgcgatcacggcggagcagaccaacgtgattctgcactccaccgagacgacgggac base pairs
gggtgccgcggggccccccgcgctagtgccgcctcgtctggttgcactaagacgtgaggtggctctgctgccctg 70426 to 70500
 Eco64I BsaHI HaeII Cfr9I SmaI Bsp143I HinfI BsmAI
 BanI Msp17I Bsp143II XmaI NdeII Esp3I
 BshNI Hsp92I BbeI PspAI PspALI Kzo9I

 BstOI AspI BbiII AatII Hin1I AcyI EcoRII BbiII EaeI Eco52I CfrI
 BsmAI Bst2UI Hsp92I KasI BbiII EheI BstNI Msp17I BstZI BsiEI
 BsmFI EcoRII BsaI Hin1I AcyI AccB1I NarI BbeI BstOI Hsp92I EclXI BsaOI
tgtccctcggagacctggacgacgtcaaggggcgcctcggcctggacgcccggccgatgatggccagcatgtgga base pairs
acagggagcctctggacctgctgcagttccccgcggagccggacctgcgggccggctactaccggtcgtacacct 70501 to 70575
BsmFI Alw26I MvaI Msp17I Eco64I Hsp92I HaeII Bst2UI AcyI CfrI Bsh1285I
 BstNI Tth111I BsaHI BshNI BsaHI BstH2I MvaI BsaHI EagI BstMCI
 Eco31I AtsI MaeII BanI Msp17I Bsp143II Hin1I HgaI XmaIII EaeI

 Esp1396I MboI AlwI BbvI Acc16I Ama87I BsoBI Bst71I DraII Eco24I ApaI AcyI
MscI Van91I NdeII AclWI Bst71I Hsp92II AvaI SmaI NlaIII Ama87I FriOI BbiII
 AccB7I NspI Bsp143I MspA1I NlaIII PspAI PspALI Bsp120I Bsp1286I BsoBI BsaHI
tcagctgctttgtgcgcatgccccgggtgcagctcgcgtttcggttcatgggccccgaggacgccgttcgcacgc base pairs
agtcgacgaaacacgcgtacggggcccacgtcgagcgcaaagccaagtacccggggctcctgcggcaagcgtgcg 70576 to 70650
 PflMI DpnII Kzo9I NspBII PaeI SphI Eco88I BsgI Hsp92II BmyI AvaI Hin1I
 MluNI Hsp92II DpnI PvuII AviII NspI Cfr9I AluI PspOMI SduI Eco88I Msp17I
 BalI NlaIII Sau3AI AluI FspI BbuI BcoI XmaI BbvI EcoO109I BcoI BanII Hsp92I

 NdeII BstI AclWI SinI BstNI Bme18I Bst2UI
 DpnII MflI DpnI DraII Bse118I EcoRII HgiEI BstOI
 BstYI BamHI AlwI BglI BssAI HgiEI Bst2UI EcoRII FokI
ggcggatcctgtgtcgcgccgccgagcaggccctcgcccgtcgccgccggtccaggcggtcccaggatgactacg base pairs
ccgcctaggacacagcgcggcggctcgtccgggagcgggcagcggcggccaggtccgccagggtcctactgatgc 70651 to 70725
HgaI Sau3AI Kzo9I EcoO109I BsrFI AvaII MvaI Eco47I MvaI
 BstX2I XhoII Cfr10I Eco47I SinI BstNI BstF5I
 MboI Bsp143I Bme18I BstOI AvaII BsmFI

 BsaWI Ksp632I BcoI AvaI SmaI BsoBI Eco24I
 MroI Bsp13I HaeII Cfr9I SrfI Eco88I Bsp1286I
 BspEI BsiMI Bsp143II BsoBI Ama87I SduI BanII
gggcggtggtggtggcggcggcgcaccactcttccggagcgcccgggccgggggtcgccgcctcgggcccgccag base pairs
cccgccaccaccaccgccgccgcgtggtgagaaggcctcgcgggcccggcccccagcggcggagcccgggcggtc 70726 to 70800
 AccIII MboII BstH2I XmaI BcoI PspOMI ApaI
 Kpn2I Eam1104I PspAI PspALI AvaI BmyI
 BseAI EarI Ama87I Eco88I Bsp120I FriOI

 BstH2I Eco47I DrdI DsaI SduI BanII Bsp1286I
 Bsp143II HgiEI Bse118I EcoO109I ApaI DraII
 SinI BsrFI BstDSI SfaNI BsgI Bsp120I FriOI PspOMI
cgccgcccggacggggaccggcccgtccgtggcatcaggccgtgcagttgttccgggccccgcgtccgggccccc base pairs
gcggcgggcctgcccctggccgggcaggcaccgtagtccggcacgtcaacaaggcccggggcgcaggcccggggg 70801 to 70875
 Bme18I BsmFI PspOMI BmyI HgaI SduI
 HaeII AvaII Cfr10I DraII Eco24I EcoO109I
 BssAI Bsp1286I Bsp120I

 HaeII
 Eco24I
 BmyI Bsp143II BssHII EcoNI
cggcgcttctgttgctggcggcggggctgtttctgggggccgctatctggtgggcggttggcgcgcgcctatgaa base pairs
gccgcgaagacaacgaccgccgccccgacaaagacccccggcgatagaccacccgccaaccgcgcgcggatactt 70876 to 70950
 BanII BsePI
 ApaI
 FriOI BstH2I

 TspRI Msp17I
 Bse1I Hin1I HgaI BstD102I
 BsmFI BsrSI BstF5I AcyI BstF5I AccBSI
agggggcgagccaccgtcccgcccgccagtgcatcccagacgcccgcgagccgcacatcccctccgctcccgcct base pairs
tcccccgctcggtggcagggcgggcggtcacgtagggtctgcgggcgctcggcgtgtaggggaggcgagggcgga 70951 to 71025
 BseNI FokI Hsp92I FokI BsrBI
 BsrI SfaNI BsaHI
 BbiII

 EcoT14I PpuMI Psp5II CfrI
 StyI Eam1105I AvaII EaeI
 HinfI Eco130I Bme18I Eco47I HphI
ccggcccgattcttacggcgcgacccaaggtcccgatggccgccccgcagtttcaccgccccagcaccattaccg base pairs
ggccgggctaagaatgccgcgctgggttccagggctaccggcggggcgtcaaagtggcggggtcgtggtaatggc 71026 to 71100
 TfiI ErhI EclHKI HgiEI XcmI
 BssT1I AspEI DraII BsmFI
 AhdI SinI EcoO109I

 BbuI FriOI EaeI
 AtsI BstH2I NlaIII BmyI BssSI
 Tth111I Bsp143II SphI SduI BsiI BalI DdeI NlaIII
ccgacaacgtccgggcgctcggcatgcgcgggctcgtgttggccaccaacaacgctcagttcatcatggataaca base pairs
ggctgttgcaggcccgcgagccgtacgcgcccgagcacaaccggtggttgttgcgagtcaagtagtacctattgt 71101 to 71175
 AspI HaeII PaeI Bsp1286I MluNI BstDEI Hsp92II
 MaeII Hsp92II Eco24I MscI
 NspI BanII CfrI

 KspI SacII HgiEI
 FokI NspBII Bsp143II
 AluI SfaNI MboII BstDSI Cfr42I Bme18I
gctacccgcatccgcacggaacgcagggtgcggtgcgagagtttcttcgcgggcaggccgcggcgctgacggacc base pairs
cgatgggcgtaggcgtgccttgcgtcccacgccacgctctcaaagaagcgcccgtccggcgccgcgactgcctgg 71176 to 71250
 BstF5I DsaI SstII BstH2I
 MspA1I HaeII AvaII
 Sfr303I SinI

 Eco88I Tsp45I BsaHI NspBII Sfr303I
 Ama87I BstPI HphI BbiII DsaI CfrI Cfr42I
 AvaI EcoO65I AflIII BbvI Hin1I BstDSI KspI SacII
tcggggtgacccacgccaacaacacgttcgccccgcagcctatgttcgcgggcgacgccgcggccgaatggctgc base pairs
agccccactgggtgcggttgttgtgcaagcggggcgtcggatacaagcgcccgctgcggcgccggcttaccgacg 71251 to 71325
 Eco47I BstEII MaeII Bst71I Msp17I MspA1I SstII
 BsoBI PspEI Hsp92I EaeI EagI EclXI
 BcoI Eco91I MaeIII AcyI HgaI BstZI XmaIII

 BsiEI MspCI
 Bsh1285I Bst98I MaeII
 BbvI BspTI MseI Bsp68I DraIII
ggccctcgttcggtcttaagcgcacgtattccccctttgtcgttcgcgaccccaagacccccagcaccccgtgag base pairs
ccgggagcaagccagaattcgcgtgcataagggggaaacagcaagcgctggggttctgggggtcgtggggcactc 71326 to 71400
Eco52I Bst71I AflII Tru1I NruI HinfI
 BstMCI Vha464I BsaAI
 BsaOI BfrI Tru9I

 EcoO109I DsaI EagI XmaIII BsaOI Esp3I Eco88I
 PpuMI Psp5II EaeI Sfr303I Bsh1285I PspAI PspALI
 PleI SinI AvaII NspBII KspI SacII Alw26I Ama87I
tcctcggcgggtccctccgcggccgtctctcgttgcccccctttcccccttcccgggtggttcaataaaaaacac base pairs
aggagccgcccagggaggcgccggcagagagcaacggggggaaagggggaagggcccaccaagttattttttgtg 71401 to 71475
 Bme18I BstDSI CfrI SstII Eco52I BsmBI BcoI XmaI
 HgiEI Eco47I BstZI Cfr42I BstMCI Cfr9I SmaI
 DraII BsmFI MspA1I EclXI BsiEI BsmAI AvaI BsoBI

 SduI ApaI Bsp143I BstMCI
 EcoO109I BanII Kzo9I Bsh1285I
 MaeII Bsp120I FriOI MboI BspCI BsaOI
caacatacgatattcgcgtttgatacgtttattggggggggtgtagggcccaacgatcggcgattaacaacacca base pairs
gttgtatgctataagcgcaaactatgcaaataaccccccccacatcccgggttgctagccgctaattgttgtggt 71476 to 71550
 PspOMI Eco24I Sau3AI Ple19I Tru1I
 DraII BmyI DpnII DpnI PvuI MseI
 Bsp1286I NdeII BsiEI Tru9I

 BsrI AviII PmlI
 BseNI NspI MaeII
 TthHB8I HgaI MaeIII NlaIII BsaAI BbvI
aacaatcgagcgcgtctaacccagtaacatgcgcacgtgatgtaggctggtcagcacggcgttgctgcgctgaaa base pairs
ttgttagctcgcgcagattgggtcattgtacgcgtgcactacatccgaccagtcgtgccgcaacgacgcgacttt 71551 to 71625
 TaqI BsrSI Hsp92II PmaCI Bst71I
 Bse1I FspI Eco72I
 Acc16I BbrPI

 AvaII BstSFI NspBII NspI MboI DpnI MvaI
 BstH2I Bme18I BbvI MspA1I Hsp92II Kzo9I BstOI BstH2I
 Bsp143II NspBII PvuII PaeI DpnII AclWI BstNI Bsp143II
cagcgccctgcgggtccgctgcagctgttgttgtatgcggcggcatgcgcggatcaaaaccgccagggcgctacg base pairs
gtcgcgggacgcccaggcgacgtcgacaacaacatacgccgccgtacgcgcctagttttggcggtcccgcgatgc 71626 to 71700
 HaeII SinI MspA1I AluI NlaIII Bsp143I EcoRII HaeII
 HgiEI SfcI PstI BbvI BbuI NdeII AlwI Bst2UI
 Eco47I Bst71I Bst71I SphI Sau3AI

 PinAI BsiEI Pfl23II BsaAI NruI
 BsrFI BsaOI SunI MaeII Bsp68I AfaI Tsp509I
 Bse118I SplI RsaI SnaBI Csp6I Sse9I Tsp45I
accggtgcttcgtacgtagcgtcgcgacaagacggcatttgcctgtacgggcaaggggccaaattgcgagtgtgg base pairs
tggccacgaagcatgcatcgcagcgctgttctgccgtaaacggacatgcccgttccccggtttaacgctcacacc 71701 to 71775
 BsaWI BstMCI BsiWI Eco105I RsaI TspEI MaeIII
 AgeI Cfr10I PspLI AfaI HgaI
 BssAI Bsh1285I Csp6I BstSNI

 BsrI
 HphI GsuI
 BsrSI EaeI BsaWI
tgactggaggtggtcggcggccaatgggccgggtggttcgtcggcggggggcaagtgcggttccggtgggagggg base pairs
actgacctccaccagccgccggttacccggcccaccaagcagccgccccccgttcacgccaaggccaccctcccc 71776 to 71850
 BseNI CfrI
 Bse1I
 BpmI


 HaeII HinfI
 TthHB8I BstF5I HinfI HgaI BseRI
gtcgagcgcctcggtatcatccgagtccgagaaacgcagggagtctgcgtcggagtgttcatcatcggaggagat base pairs
cagctcgcggagccatagtaggctcaggctctttgcgtccctcagacgcagcctcacaagtagtagcctcctcta 71851 to 71925
 TaqI Bsp143II FokI PleI PleI
 BstH2I


 HgaI TaqI
 Bst71I Bst71I TthHB8I
 BsgI Eco57I SfaNI HinfI HindII Tsp45I
gtgcagcgtctgaagcagcgatgcgggtgggggcgcggagtcgacgtgaagcgcgagagaggaagcccacgaagt base pairs
cacgtcgcagacttcgtcgctacgcccacccccgcgcctcagctgcacttcgcgctctctccttcgggtgcttca 71926 to 72000
 BbvI BbvI SalI PleI MaeIII
 AlwNI AccI MaeII
 HincII

 BsrI Eco88I Msp17I SinI Tth111I
 BseNI Alw26I Hin1I HgaI Eco47I Alw26I
 NspBII TspRI HinfI AvaI BbiII Bme18I AspI BsmAI
cacagcggacactgggaggtgggtgtttgtatgtgtgggagactcgggcgtcgggaccgagtctcggctctgggg base pairs
gtgtcgcctgtgaccctccacccacaaacatacacaccctctgagcccgcagccctggctcagagccgagacccc 72001 to 72075
 MspA1I BsrSI Ama87I DrdI AcyI HgiEI AtsI
 Bse1I BcoI BsoBI Hsp92I BsmFI PleI
 BsmAI PleI BsaHI AvaII HinfI


 HgaI
tgtaagcgtccgagttacgggcggcaggggcggctggggcaggggcggctggggcaggggcggctggggcagggg base pairs
acattcgcaggctcaatgcccgccgtccccgccgaccccgtccccgccgaccccgtccccgccgaccccgtcccc 72076 to 72150
 MaeIII


cggctggggcaggggcggctggggcaggggcggctggggcaggggcggctggggcaggggcggctggggcagggg base pairs
gccgaccccgtccccgccgaccccgtccccgccgaccccgtccccgccgaccccgtccccgccgaccccgtcccc 72151 to 72225


 SduI
 BshNI BsePI SfaNI
 Eco64I BssHII BstF5I
cggctggggcaggggcggctggggcaggggcggctggggcaccgagcgcgcgcggatgcgcgtccgcgcggcggg base pairs
gccgaccccgtccccgccgaccccgtccccgccgaccccgtggctcgcgcgcgcctacgcgcaggcgcgccgccc 72226 to 72300
 BanI BmyI FokI HgaI
 AccB1I
 Bsp1286I

 BsrI
 HphI BmyI
 Tsp45I BsrSI SduI
tttggtcgcgggtgactggggtggggggcggcgggcaaccgggcctccgggcacgacccaaccgcacaaaggctc base pairs
aaaccagcgcccactgaccccaccccccgccgcccgttggcccggaggcccgtgctgggttggcgtgtttccgag 72301 to 72375
 MaeIII BseNI Bsp1286I
 Bse1I


 BsoBI MvaI
 Eco88I BstOI
 Ama87I EcoRII
gctcggggcaaccgggcctggggccaaaggcggggggctggtctggacggcggaggtcgggggggcaaggcccgg base pairs
cgagccccgttggcccggaccccggtttccgccccccgaccagacctgccgcctccagcccccccgttccgggcc 72376 to 72450
 BcoI BstNI
 AvaI Bst2UI


 Bst71I EaeI EagI XmaIII BsaOI HgiEI Ama87I PspALI
 BglI NspBII KspI EclXI BstMCI AvaII PspAI BsoBI
 TspRI NspBII BstDSI CciNI NotI Bsh1285I DraII BcoI XmaI
agaaggcggcactgccgccgctgcggcggaaaccgcggccgcgtggtcggctgggtcccggggagaggggaggga base pairs
tcttccgccgtgacggcggcgacgccgcctttggcgccggcgcaccagccgacccagggcccctctcccctccct 72451 to 72525
 MspA1I DsaI CfrI Cfr42I Eco52I PpuMI Psp5II Eco88I
 BbvI MspA1I Sfr303I BsiEI Bme18I Eco47I AvaI
 BstZI SstII SacII SinI EcoO109I Cfr9I BsmFI

 BstMCI SstII BshNI Hsp92I BbeI NgoMI BstZI FseI
 BstDSI Sfr303I BanI Msp17I Bsp143II Cfr10I NaeI
 BsiEI MspA1I Eco64I BsaHI HaeII BsrFI CfrI Eco52I
gttcaacgaggccgagagcgaggcgaccgcggggcgcgtgaggcgccggggtgggccggccgcggggccccgggg base pairs
caagttgctccggctctcgctccgctggcgccccgcgcactccgcggccccacccggccggcgccccggggcccc 72526 to 72600
 Bsh1285I Cfr42I AccB1I NarI BstH2I Bse118I EclXI
SmaI DsaI NspBII KasI BbiII EheI BssAI EaeI XmaIII
 BsaOI KspI SacII Hin1I AcyI MroNI NgoAIV EagI BsiEI

 BstMCI SstII EcoO109I Eco24I BsoBI PpuMI Eco47I CciNI BsiEI NspBII SacII
Bsh1285I Sfr303I Ama87I Cfr9I XmaI SinI AvaII MspA1I NotI BstDSI MspA1I BsiEI
 BsaOI Cfr42I DraII PspAI FriOI SmaI EcoO109I EaeI XmaIII BstMCI SstII Bsh1285I
gggtgtcggcgagggacccgctgttgtctggcggcggccgcggcggcggtcgcccccggggacgaccgctccttc base pairs
cccacagccgctccctgggcgacaacagaccgccgccggcgccgccgccagcgggggcccctgctggcgaggaag 72601 to 72675
 BstDSI KspI Bsp120I BmyI Eco88I PspALI DraII NspBII EagI Bsh1285I Sfr303I
 DsaI MspA1I PspOMI Bsp1286I BanII Bme18I Psp5II BstZI Eco52I BsaOI Cfr42I
 NspBII SacII SduI BcoI AvaI ApaI HgiEI BsmFI CfrI EclXI DsaI KspI BglI

Ama87I XmaI BsiEI BstD102I
 PspAI PspALI BsaOI FokI
 BsoBI BstMCI BstF5I EcoO109I
ggcgggcggaggcgggatgggcgcgagcgtgggggcgggaaaggccccgcgagccgaggcggggccgggcggaag base pairs
ccgcccgcctccgccctacccgcgctcgcacccccgccctttccggggcgctcggctccgccccggcccgccttc 72676 to 72750
BstMCI AvaI BsmFI BsrBI DraII
BsaOI Eco88I Bsh1285I
BcoI Cfr9I SmaI AccBSI


 HinfI HgaI
gggcaaagcagaaacccaagccgggggcgcggactccggggtgggcggctggtcgggaggacgcgcggaagcggc base pairs
cccgtttcgtctttgggttcggcccccgcgcctgaggccccacccgccgaccagccctcctgcgcgccttcgccg 72751 to 72825
 PleI


 NgoAIV BbiII Eco88I Eco88I BsePI
 BstMCI BstMCI NgoMI Hin1I HgaI PspAI PspALI
 BsiEI BsiEI MroNI Cfr10I AcyI BsoBI Ama87I BssHII
gaccggggcgaccggggcggggagtgccggcggacgccacccctcggggggggcggaggcccggggcgcgcgcga base pairs
ctggccccgctggccccgcccctcacggccgcctgcggtggggagccccccccgcctccgggccccgcgcgcgct 72826 to 72900
 Bsh1285I Bsh1285I BssAI NaeI BsaHI Ama87I BcoI XmaI
 BsaOI BsaOI BsrFI Msp17I BcoI Cfr9I SmaI
 Bse118I Hsp92I AvaI AvaI BsoBI

 EcoO109I BspMI
 PpuMI Psp5II
 AflIII SinI AvaII AviII
tttggcacgcgtccggcgggacctgcgcacgcgcggcacggcggcggagaaagcggcggcagagccggaaaaggc base pairs
aaaccgtgcgcaggccgccctggacgcgtgcgcgccgtgccgccgcctctttcgccgccgtctcggccttttccg 72901 to 72975
 MluI HgaI Bme18I FspI Acc16I
 HgiEI Eco47I
 DraII BsmFI

 FokI Sfr303I
 DsaI MspA1I BsmFI
 SfaNI KspI HinfI BstDSI Bsp68I BstDSI
cgggggaggaagcgcggcatccgcggggggactcggtgtgggtggcgagggccgtgggtcgtcgcgaggggccac base pairs
gccccctccttcgcgccgtaggcgcccccctgagccacacccaccgctcccggcacccagcagcgctccccggtg 72976 to 73050
 BstDSI SstII PleI DsaI NruI DsaI
 BstF5I Cfr42I
 NspBII SacII

 AspEI BsaOI BsrFI NaeI
 BmyI EclHKI BssAI BbvI
 SduI BsmFI BsiEI Bsp68I AluI Bse118I SfiI
gggcacgcgccccgtgttttgttgaggcgggacactcggtcgtgtttcgcgagccgtagctgccggcccgatggg base pairs
cccgtgcgcggggcacaaaacaactccgccctgtgagccagcacaaagcgctcggcatcgacggccgggctaccc 73051 to 73125
 Bsp1286I AhdI BstMCI NruI MroNI Cfr10I
 Eam1105I NgoMI Bst71I
 Bsh1285I NgoAIV BglI

 KspI SacII BsrI Sau3AI
 DsaI SstII AfaI MaeII NdeII
 NspBII Csp6I BsrSI BsmFI Kzo9I MboII
ccgcggtgcgtactgggacgtggggacggactgatcggtggcgggggggggaagaagggccggggccggattggg base pairs
ggcgccacgcatgaccctgcacccctgcctgactagccaccgcccccccccttcttcccggccccggcctaaccc 73126 to 73200
 BstDSI Cfr42I BseNI DpnII
 MspA1I RsaI Bse1I MboI DpnI
 Sfr303I BsmFI Bsp143I

 BsrFI Msp17I HgaI AcyI Bst2UI PflMI NdeII MflI DpnI BstOI BsmFI
 BssAI NaeI Hsp92I Hsp92I BstNI DsaI BstYI Sau3AI Kzo9I Bst2UI
 SgrAI Bse118I Hin1I HgaI EcoRII GsuI Esp1396I BamHI EcoRII Hsp92II
cgtggggccgccggcgtcgtcggacgccagctcctccaggccgtggatccaggcccacatgcgaggggggacggg base pairs
gcaccccggcggccgcagcagcctgcggtcgaggaggtccggcacctaggtccgggtgtacgctcccccctgccc 73201 to 73275
 MroNI Cfr10I AcyI BbiII BseRI BpmI Van91I MboI XhoII BstNI NspI
 NgoMI Hin1I BsaHI BsaHI BstOI AccB7I BstX2I BstI AclWI MvaI
 NgoAIV BbiII Msp17I AluI MvaI BstDSI DpnII Bsp143I AlwI NlaIII

 BanII Cfr10I BsaHI EcoO109I PspAI PspALI Eco47I
 BmyI BssAI BbiII PpuMI Psp5II AvaI SmaI
 SduI SgrAI Hin1I HgaI BseRI SinI AvaII Cfr9I DrdI AvaII NspBII
ctcgccggtggtggcgtcggtgaggagagtgggggcgaggacccccgggtccgcctgccgtgcggggggggcagc base pairs
gagcggccaccaccgcagccactcctctcacccccgctcctgggggcccaggcggacggcacgccccccccgtcg 73276 to 73350
 Bsp1286I Msp17I HphI Bme18I BcoI Eco88I Bme18I MspA1I
 Eco24I Bse118I AcyI HgiEI Eco47I XmaI SinI
 FriOI BsrFI Hsp92I DraII Ama87I BsoBI HgiEI

 SinI AvaII Eco88I EcoO109I MboI AclWI HgiEI BstDSI Sfr303I BstMCI EcoO1
BbvI PpuMI Eco47I SinI DraII DpnII DpnI Bme18I Eco47I KspI SacII PpuMI
 HgiEI Ama87I PpuMI Psp5II Sau3AI FokI EcoO109I NspBII BsiEI SinI
ggggtcctcgggacccgatccgccatccccccccgcaaggtcccgcgggtcgcgggcggcggtcggggcagaggg base pairs
ccccaggagccctgggctaggcggtaggggggggcgttccagggcgcccagcgcccgccgccagccccgtctccc 73351 to 73425
 EcoO109I AvaI HgiEI BsmFI Bsp143I SinI DraII DsaI SstII Bsh1285I Bme18I
Bst71I DraII BcoI Bme18I Eco47I Kzo9I BstF5I AvaII BsmFI Cfr42I HgiEI
 Bme18I Psp5II BsoBI AvaII NdeII AlwI PpuMI Psp5II MspA1I BsaOI DraII

 09I Eco88I BsaHI
 Psp5II BstH2I PspAI PspALI BbiII
 AvaII BglI Bsp143II Ama87I SmaI Hin1I
acctgcctcgtcggcgagggggcgctggtaaaccgggtgtcccgggaacagctcccccgtcaggagggaggcgtc base pairs
tggacggagcagccgctcccccgcgaccatttggcccacagggcccttgtcgagggggcagtcctccctccgcag 73426 to 73500
 BspMI HaeII BcoI XmaI AluI Msp17I
 Eco47I Cfr9I BsmFI Hsp92I
 BsmFI AvaI BsoBI AcyI

 BsoBI AvaII MspA1I
 HgaI Eco88I Bme18I BstH2I DsaI
 TaqI Ama87I BstF5I MboII HgaI Bsp143II MaeII BsmFI
gaagggccgcccgaggatggcccgcgcgaagaaggggtccgcgtcggcggcgctcgccgcgagaacgtcccccgc base pairs
cttcccggcgggctcctaccgggcgcgcttcttccccaggcgcagccgccgcgagcggcgctcttgcagggggcg 73501 to 73575
 BcoI FokI SinI HaeII BstDSI
 AvaI HgiEI NspBII
 TthHB8I Eco47I

 FriOI
 SstII BmyI
 Sfr303I AluI BseRI BbvI MaeII SduI
ggtagccacaaacggaagctcctcggtggcctcgctgcccacaaaccgcacgtcaggggggccggggggctccgg base pairs
ccatcggtgtttgccttcgaggagccaccggagcgacgggtgtttggcgtgcagtccccccggccccccgaggcc 73576 to 73650
 Cfr42I Bst71I Bsp1286I
 Eco24I
KspI SacII BanII

 PshAI HgiEI BstOI SduI ApaI
 BstMCI MslI Bme18I MvaI EcoO109I BanII
 BsiEI NlaIII BsiI Eco47I Bsp120I FriOI
ggcttcccacaagaccgcgaccggggtcatggagatgtccacgaggaccaggcacgggggcccgtcggcgagagg base pairs
ccgaagggtgttctggcgctggccccagtacctctacaggtgctcctggtccgtgcccccgggcagccgctctcc 73651 to 73725
 Bsh1285I Hsp92II SinI EcoRII PspOMI Eco24I
 BsaOI BssSI BstNI DraII BmyI
 AvaII Bst2UI Bsp1286I

 MboI Kzo9I PvuI MboI
 BstH2I BstH2I Bst71I DpnII DpnI Bsh1285I
 Bsp143II Bsp143II AluI AflIII TthHB8I BsiEI DpnII
gcgctcggcgatgagcgccgacaggcgcgggagctgcgccgccagacacgcgttttcgatcgggttgagatcggt base pairs
cgcgagccgctactcgcggctgtccgcgccctcgacgcggcggtctgtgcgcaaaagctagcccaactctagcca 73726 to 73800
 HaeII HaeII BbvI MluI TaqI Bsp143I BstMCI
 NdeII BspCI BsaOI
 Sau3AI Ple19I NdeII

 BsmBI Hin1I BsaHI BsaHI
 DpnI TaqI Eam1105I AatII BbiII
 Bsp143I BseRI MaeII Alw26I AhdI BbiII Hin1I
gtggaggaggccgacggcccacgtctcgatgtcggacgacacgacgtcgcgcagggcggcgtccggcccgccggg base pairs
cacctcctccggctgccgggtgcagagctacagcctgctgtgctgcagcgcgtcccgccgcaggccgggcggccc 73801 to 73875
 Kzo9I TthHB8I EclHKI MaeII Msp17I
Sau3AI BsmAI AspEI Hsp92I Hsp92I
 Esp3I Msp17I AcyI AcyI HgaI

 EarI HinfI
 TaqI Eam1104I Bse1I BstH2I
 HinfI MboII BsrSI PleI BstDSI Bsp143II
gcgcgagtcgaagagcgtcaggcacagttccagttccgactcgcgggagaaggccgtggtgttgcggagcgccac base pairs
cgcgctcagcttctcgcagtccgtgtcaaggtcaaggctgagcgccctcttccggcaccacaacgcctcgcggtg 73876 to 73950
 TthHB8I SapI BseNI DsaI HaeII
 PleI Ksp632I BsrI
 HgaI

 BmyI BstDSI KspI EcoRII Bme18I StyI Bsp19I MaeIII
 BsePI Bsp1286I MspA1I SacII Bst2UI ErhI EcoT14I Hsp92II
 AscI BseRI Alw21I EaeI Cfr42I SinI Eco130I DsaI CfrI
cacgacgggcgcgccgaggagcaccgcggccagaaccaggtccatggccgtaacgcgcgcggcgggggtgcggtg base pairs
gtgctgcccgcgcggctcctcgtggcgccggtcttggtccaggtaccggcattgcgcgcgccgcccccacgccac 73951 to 74025
 BssHII SduI BsiHKAI Sfr303I BstOI AvaII NcoI BstXI BssHII
 AspHI DsaI CfrI SexAI MvaI Eco47I BstDSI NlaIII
 Bbv12I NspBII SstII BstNI HgiEI BssT1I EaeI BsePI

 PmaCI Van91I Eco88I BsaHI
 EaeI BsaAI Esp1396I Ama87I BbiII
 EaeI SfiI MaeII AccB7I EcoO109I EcoNI Hin1I
ggtcgcggcggccagcacggccacgtgctggcccgtgggtcggtagagggcgtggggggcctcggggagggacgc base pairs
ccagcgccgccggtcgtgccggtgcacgaccgggcacccagccatctcccgcaccccccggagcccctccctgcg 74026 to 74100
 CfrI BglI Eco72I BstDSI DraII BsoBI Msp17I
 CfrI PmlI PflMI BcoI Hsp92I
 BbrPI DsaI AvaI AcyI

 MluNI BstOI BstOI Hsp92I
 CfrI PleI GsuI BstNI BbiII TaqI AfaI
 HgaI HgaI HinfI MvaI EaeI MvaI Hin1I TthHB8I Csp6I
ctcgcgcccccccgccgggccgagcgtctggccagactccaggcgtgcggccaggagggcgtcgaagctgtcgta base pairs
gagcgcgggggggcggcccggctcgcagaccggtctgaggtccgcacgccggtcctcccgcagcttcgacagcat 74101 to 74175
 EaeI EcoRII BpmI EcoRII Msp17I AluI RsaI
 MscI BstNI CfrI BglI AcyI
 BsmFI BalI Bst2UI Bst2UI BsaHI HgaI

 Bme18I BcoI
 NspI AvaII BstH2I NspI AvaI
 NlaIII Eco47I EaeI BglI Bsp143II NlaIII BsoBI
ctcggtgtagtcgtcgggaaacatgcaggtccacagcgcggccaaagcggcgctcggcagacacatgcgcccgag base pairs
gagccacatcagcagccctttgtacgtccaggtgtcgcgccggtttcgccgcgagccgtctgtgtacgcgggctc 74176 to 74250
 Hsp92II BspMI CfrI HaeII Hsp92II
 SinI Ama87I
 HgiEI Eco88I

 MvaI DraII Esp1396I Eco88I Msp17I AatII Bme18I Eco47I
 BstOI AlwNI Van91I AluI BsoBI BstH2I MaeII HaeII HgiEI
 HgaI HphI BstNI EcoRII BstNI DdeI Ama87I Bsp143II BsaHI Bsp143II AvaII
gacgctcaccgccgccagggcctgggccggactgagcttcccgagcgccgggacgtcccggcgctgggtcccgag base pairs
ctgcgagtggcggcggtcccggacccggcctgactcgaagggctcgcggccctgcagggccgcgacccagggctc 74251 to 74325
 EcoRII EcoO109I MvaI BstDEI HaeII Hsp92I BsmFI SinI DraII
 Bst2UI PflMI BstOI BcoI Hin1I AcyI BstH2I EcoO1
 BglI AccB7I Bst2UI AvaI BbiII BsmFI PpuMI Psp5II

 BsmFI AspHI FriOI Eco130I BstH2I Eco64I Hsp92I HaeII AluI Hin1I NarI
 Ama87I AluI BmyI Psp124BI StyI HaeII Bst2UI Hin1I AcyI NspBII BshNI BsaHI
 Eco88I SduI Bbv12I Alw21I EcoT14I BstOI AccB1I NarI BbeI Eco64I Hsp92I
ctccaaggccgagcgccagggcgccagcgggtcggtttcggacagcttgccccggcgccagtcggccagccgcgt base pairs
gaggttccggctcgcggtcccgcggtcgcccagccaaagcctgtcgaacggggccgcggtcagccggtcggcgca 74326 to 74400
 BcoI Ecl136II Eco24I BsiHKAI Bsp143II BanI Msp17I Bsp143II BanI Msp17I Bsp143II
 09I AvaI EcoICRI SacI SstI BssT1I BstNI KasI BbiII EheI MspA1I AccB1I AcyI
 BsoBI Bsp1286I BanII ErhI EcoRII MvaI BshNI BsaHI BstH2I KasI BbiII EheI

BbeI Bse1I PspAI BsoBI Msp17I
BstH2I EaeI Ama87I SmaI SfaNI AcyI
HaeII BsrI EcoO109I XmaI EcoO109I MaeII MslI BstF5I BsaHI
gccgaacaggaggccccgggtcggggggcctccgtccaaaaacgtcggcaacacgcggatgcgggcgtcgggatg base pairs
cggcttgtcctccggggcccagccccccggaggcaggtttttgcagccgttgtgcgcctacgcccgcagccctac 74401 to 74475
 DraII Eco88I DraII FokI Hsp92I
 BsrSI CfrI BcoI AvaI Hin1I HgaI
 BseNI Cfr9I PspALI BbiII

 RsaI
 SfaNI HaeII HinfI MspA1I TaqI Hsp92II
BstF5I AlwNI NlaIII NspBII HgaI TthHB8I HphI NlaIII
cggggtcaggcgctggacgaacagcatggactccgctgcgtcctcgaacgcgcgttcgagggtgaggtgcatgta base pairs
gccccagtccgcgacctgcttgtcgtacctgaggcgacgcaggagcttgcgcgcaagctcccactccacgtacat 74476 to 74550
FokI Bsp143II Hsp92II BbvI TthHB8I TaqI Csp6I
 BstH2I PleI Bst71I NspI
 AfaI

 Eco47I Eco64I Msp17I Bsp143II BsaWI NgoMI CfrI
 HgiEI BstOI AccB1I NarI BbeI AccIII BssAI EaeI
 BsiI SinI BstNI KasI Hsp92I HaeII BspEI BsiMI NgoAIV
ctcgtgctggcgaacgaggtccaggcgccagaagttgtagatgtgttccggaacgccggccaccagcgcgaccag base pairs
gagcacgaccgcttgctccaggtccgcggtcttcaacatctacacaaggccttgcggccggtggtcgcgctggtc 74551 to 74625
 BssSI Bme18I MvaI BshNI BsaHI BstH2I BseAI MroNI Cfr10I
 AvaII Bst2UI Hin1I AcyI MroI Bsp13I Bse118I
 EcoRII BanI BbiII EheI Kpn2I BsrFI NaeI

 Bme18I Eco47I Eco88I PspOMI Eco24I BanI
 HaeII HgiEI BsmFI AvaI Bsp120I BmyI HgaI
 DrdI HgaI Bsp143II AvaII BcoI XmaI EcoO109I ApaI BshNI
cacgtcgttctcgttgaaggcgacgcagtggcgctgggacccccgggggcccggcggcggacgcggcgccgccgc base pairs
gtgcagcaagagcaacttccgctgcgtcaccgcgaccctgggggcccccgggccgccgcctgcgccgcggcggcg 74626 to 74700
 MaeII TspRI SinI DraII Ama87I BsoBI DraII BanII AccB1I
 BstH2I EcoO109I PspAI PspALI Bsp1286I Eco64I
 PpuMI Psp5II Cfr9I SmaI SduI FriOI KasI

 Msp17I Bsp143II Bsp13I Msp17I PvuII Bsp1286I
Hin1I AcyI BspEI BsaWI BstD102I HgaI PspOMI ApaI RsaI
 Hsp92I BbeI MroI AccBSI Hsp92I Bsp120I BanII MaeII
tccggacgcccagcccagctgggcccagcgacacccaaactcgcgcgtgagggtggtggcgacgagggcgacgta base pairs
aggcctgcgggtcgggtcgacccgggtcgctgtgggtttgagcgcgcactcccaccaccgctgctcccgctgcat 74701 to 74775
 NarI HaeII Kpn2I BsrBI BsaHI NspBII BmyI Csp6I
 BsaHI BstH2I BseAI Hin1I AcyI MspA1I Eco24I AfaI
 BbiII EheI AccIII BsiMI BbiII AluI SduI FriOI

 EagI Bsh1285I KasI BbiII EheI EcoRII MspA1I
 BstZI BstMCI TaqI AccB1I NarI BstH2I Bst2UI BstMCI AluI
 AluI EclXI HgaI Eco64I BsaHI HaeII BstOI BsiEI PvuII
cagctcggccgccgcgtccatcgaggcgccccacgtcgcctggcgatggcgcacgaagcgaccgaacagctgaaa base pairs
gtcgagccggcggcgcaggtagctccgcggggtgcagcggaccgctaccgcgtgcttcgctggcttgtcgacttt 74776 to 74850
 EaeI Eco52I TthHB8I Hin1I AcyI MaeII MvaI Bsh1285I
 CfrI BsiEI BanI Msp17I Bsp143II BsaOI NspBII
 XmaIII BsaOI BshNI Hsp92I BbeI BstNI

 MvaI BbiII DdeI GsuI RsaI Tsp45I
 BstOI BsaHI NspBII Cfr10I Csp6I NlaIII
 EcoRII Msp17I BstDEI PvuII BssAI MaeII EaeI
gttggcggcctgggcgtcgctgagggccagctggagccggttcacgacggtcagcacgtacatggccgtgaccgt base pairs
caaccgccggacccgcagcgactcccggtcgacctcggccaagtgctgccagtcgtgcatgtaccggcactggca 74851 to 74925
 BstNI Hsp92I AluI BsrFI BsaAI CfrI
 Bst2UI AcyI MspA1I BpmI MslI Hsp92II
 Hin1I HgaI Bse118I AfaI MaeIII

 Hsp92I BsoBI
 TthHB8I BsaHI Eco88I SfaNI BbvI
 HinfI Hin1I AcyI Ama87I BsmFI BssHII
cggggccgattcgaggacgtccgtcggaagcgggggccgcacgcaggccgcctcgggacgcatcagcagcgcgcc base pairs
gccccggctaagctcctgcaggcagccttcgcccccggcgtgcgtccggcggagccctgcgtagtcgtcgcgcgg 74926 to 75000
 TfiI Msp17I BcoI HgaI BsePI
 TaqI BbiII AatII AvaI Bst71I
 MaeII PshAI

 BstZI Bsh1285I BstSFI
 HphI EclXI AfaI MspA1I BstXI
 Tsp45I EagI BstMCI Csp6I PstI XcmI
gagtttgtcggtgacggccgggaagcatagcgcgtactgcagcggcgttccgtccggggccaaaaagctggtggc base pairs
ctcaaacagccactgccggcccttcgtatcgcgcatgacgtcgccgcaaggcaggccccggtttttcgaccaccg 75001 to 75075
 MaeIII XmaIII BsaOI RsaI NspBII AluI
 EaeI Eco52I SfcI BbvI
 CfrI BsiEI Bst71I

 MboI Bsp143I Bsp143II BstOI DraII BcoI AvaI ApaI BsaI BssAI
 BstX2I DpnI Eco47III EcoRII PspOMI BmyI Eco88I PspALI MroNI
 BstYI MflI AlwI BstH2I BbvI MvaI Ama87I Cfr9I BanII Alw26I NgoMI
gaacggcagatccagagcgctgacggcctcacgcagcaccaggggccccgggtctccgccggcgcgcagatacgc base pairs
cttgccgtctaggtctcgcgactgccggagtgcgtcgtggtccccggggcccagaggcggccgcgcgtctatgcg 75076 to 75150
 DpnII XhoII Aor51HI Bst71I Bsp120I Bsp1286I XmaI Eco31I BsrFI
 NdeII Kzo9I AfeI BstNI EcoO109I Eco24I BsoBI BsmAI NgoAIV
 Sau3AI AclWI HaeII Bst2UI SduI PspAI FriOI SmaI SgrAI

 NaeI AluI Bst71I Eco88I BcoI Eco88I Msp17I
 Cfr10I AlwNI SalI HindII Ama87I Ksp632I AvaI SmaI
 BssHII BbvI MspA1I TaqI BsiI BsoBI MboII Ama87I BsoBI BbiII
ctcgccccggcggcgcagcagctgcgggtcgacctcgtggccctcgggggaagaagaggcccgggcgcgggcgtc base pairs
gagcggggccgccgcgtcgtcgacgcccagctggagcaccgggagcccccttcttctccgggcccgcgcccgcag 75151 to 75225
 BsePI BglI Bst71I BbvI HincII BssSI Eam1104I PspALI Hsp92I
 PvuII TthHB8I BcoI EarI Cfr9I SrfI BsaHI
Bse118I NspBII AccI AvaI PspAI XmaI Hin1I

 HgaI Bsp143I NgoAIV BstOI
 AcyI MboI DpnI CfrI NgoMI EcoRII
 TaqI DpnII MboII HinfI BglI MroNI Cfr10I
gagggcgcgaagatcaacgagcaggggcgcgggcgcggactccgcgcccgcgcccgtctggccgccggccctggc base pairs
ctcccgcgcttctagttgctcgtccccgcgcccgcgcctgaggcgcgggcgcgggcagaccggcggccgggaccg 75226 to 75300
 NdeII PleI EaeI BssAI NaeI
 Sau3AI BsrFI BstNI
 TthHB8I Kzo9I Bse118I Bst2UI

 BsiWI
 PspLI AfaI BglI
 SplI RsaI NlaIII BstF5I BssHII BseRI BstDSI EcoRII
gtacgcgctatataagcccatgcggtattggatgagttcccgcgcgccccggaactcctccaccgcccacggggc base pairs
catgcgcgatatattcgggtacgccataacctactcaagggcgcgcggggccttgaggaggtggcgggtgccccg 75301 to 75375
MvaI Csp6I Hsp92II FokI BsePI DsaI
 Pfl23II
 SunI

 MvaI HgiEI NspBII Sfr303I Bsh1285I DraII Bst2UI AcyI MvaI BshNI
 BstOI Eco47I CfrI KspI EclXI BsaOI EcoO109I Hin1I HgaI BstOI SduI
 Bme18I MspA1I Cfr42I BsiEI TaqI EcoRII Msp17I EcoRII BanI
caggtccgcggccgccgcgtcgaactccgccagcaggccccccagggcgtcaaagttcatctcccagggcaccct base pairs
gtccaggcgccggcggcgcagcttgaggcggtcgtccggggggtcccgcagtttcaagtagagggtcccgtggga 75376 to 75450
 BstNI AvaII EaeI EagI XmaIII BstMCI BstNI Hsp92I BstNI AccB1I
 Bst2UI BstDSI CciNI NotI Eco52I HgaI BstOI BsaHI Bst2UI Bsp1286I
 SinI DsaI BstZI SstII SacII TthHB8I MvaI BbiII Eco64I

 AviII BsePI
 FspI Bst71I HphI DsaI
BmyI BstF5I Tsp45I HgaI AluI BseRI
gcgcaccacctcatcccgcagccgggcgcacagggcggtgtgcttggtgacgcgcgcgcccagctcctccacggc base pairs
cgcgtggtggagtagggcgtcggcccgcgtgtcccgccacacgaaccactgcgcgcgcgggtcgaggaggtgccg 75451 to 75525
 FokI BbvI MaeIII BstDSI
 BssHII
 Acc16I

 AccB1I NarI HaeII BssT1I AccB1I AcyI EcoRII BbiII EcoRII Asp718I
 KasI BbiII EheI ErhI BanI Hin1I NarI BbeI MvaI BsaHI BstOI BshNI
 BssHII Eco64I BsaHI BbeI StyI BshNI BsaHI HaeII BstOI Hsp92I MvaI Acc65I
ctccgcgcgctcggcgcccttggcgcccaggacgccctggtacctggcggaaaggcgctcgtaggccggctgggc base pairs
gaggcgcgcgagccgcgggaaccgcgggtcctgcgggaccatggaccgcctttccgcgagcatccggccgacccg 75526 to 75600
 BsePI BanI Msp17I Bsp143II Eco64I Hsp92I BstH2I Bst2UI HgaI Eco64I Csp6I
 BshNI Hsp92I Eco130I KasI Msp17I EheI BstNI Msp17I BstNI BanI
 Hin1I AcyI BstH2I EcoT14I BbiII Bsp143II Hin1I AcyI Bst2UI AccB1I

 EcoRII HaeII BsrFI BglI Eco24I SfcI BssHII BbvI BsoBI
 AfaI MvaI NgoMI SfiI PspOMI ApaI Sse8387I AluI NlaIII Eco88I
 KpnI Bsp143II Bse118I BmyI BanII SbfI PvuII BsiI Ama87I
ccgcagccccgacaccgtgttggtggtgtcctgcagggcgcgcagctgctcgtgcatggcgcggaacccctcggg base pairs
ggcgtcggggctgtggcacaaccaccacaggacgtcccgcgcgtcgacgagcacgtaccgcgccttggggagccc 75601 to 75675
 BstNI BstH2I Cfr10I Bsp1286I Bst71I BsePI Bst71I BssSI BcoI
RsaI BstOI MroNI NgoAIV SduI FriOI BstSFI NspBII Hsp92II AvaI
 Bst2UI BssAI NaeI Bsp120I BbvI PstI MspA1I

 Bst2UI Hin1I AcyI EaeI BstOI
 BstNI BshNI BsaHI BstH2I AtsI BseRI
 BsmFI Eco64I Msp17I EheI HgaI Tth111I EcoNI
ggacttccaggcgcccccccggacgcggccaaagcgaccccagacctcgtcccactccgcctcggcctcctccag base pairs
cctgaaggtccgcgggggggcctgcgccggtttcgctggggtctggagcagggtgaggcggagccggaggaggtc 75676 to 75750
 EcoRII BanI BbiII Bsp143II AspI BsmFI EcoRII
 BstOI AccB1I NarI BbeI CfrI BstNI
 MvaI KasI Hsp92I HaeII Bst2UI

 Bme18I Psp5II BsaHI HgaI BanI Msp17I Bsp143II BstNI CfrI MroNI Bse118I
 BpmI DraII Hin1I SalI HindII AccB1I AcyI Bsp143II MvaI XmaIII NgoAIV BsaOI
 MvaI HgiEI BsmFI Hsp92I HincII BshNI BsaHI BstH2I BstOI EagI BssAI BsiEI NaeI
ggacctccgcagggcgtcgacgcggcgccgagtatcaaagagcgcccccaggcggccggcgtgccgcgccagggg base pairs
cctggaggcgtcccgcagctgcgccgcggctcatagtttctcgcgggggtccgccggccgcacggcgcggtcccc 75751 to 75825
 GsuI EcoO109I BbiII AccI HgaI Hin1I NarI BbeI EcoRII BstZI NgoMI Bsh1285I
 SinI AvaII Msp17I TthHB8I KasI BbiII EheI HaeII Bst2UI EclXI BsrFI BstMCI
 PpuMI Eco47I AcyI TaqI Eco64I Hsp92I HaeII BstH2I EaeI Eco52I Cfr10I EcoRII

BstNI KspI SacII CelII BsmAI Bbv12I
 NspBII Bpu1102I TthHB8I AspHI BfaI
 MvaI BstDSI Cfr42I DdeI Bsp1720I Alw26I SduI BsiHKAI
gccggggccgtcgccgcgggcggcgcttagcgggtgcgtctcgaaggtgcgctgggcgtgctctagccagataac base pairs
cggccccggcagcggcgcccgccgcgaatcgcccacgcagagcttccacgcgacccgcacgagatcggtctattg 75826 to 75900
 BstOI DsaI SstII Bsp143II HgaI Esp3I Bsp1286I
 Bst2UI MspA1I HaeII BlpI TaqI BmyI MaeI
 Sfr303I BstH2I BstDEI BsmBI Alw21I

 KspI SacII Ecl136II Eco24I SstI Sau3AI AspI MvaI Bsp143I
 NspBII SduI TaqI Bsp1286I BanII NdeII AlwI SexAI AtsI Sau3AI
BstDSI Cfr42I TthHB8I BmyI SacI BsiHKAI Kzo9I TthHB8I BstOI MboI DpnI
cgcgggcacgtcgagctcgcgcgttttctcggtctgatccaacagaacctcgacctggtcggcgatctccgccac base pairs
gcgcccgtgcagctcgagcgcgcaaaagagccagactaggttgtcttggagctggaccagccgctagaggcggtg 75901 to 75975
DsaI SstII BmyI AluI AspHI FriOI DpnII AclWI TaqI Tth111I DpnII
 MspA1I Bsp1286I SduI Bbv12I Alw21I Bsp143I EcoRII Bst2UI Kzo9I
 Sfr303I MaeII EcoICRI Psp124BI MboI DpnI BstNI NdeII

 Bst2UI MluNI BstMCI BalI
 EcoRII TaqI CfrI BsiEI Hsp92II MscI
 BssHII TthHB8I HgaI BstDSI BglI XcmI NlaIII EaeI
cgagcgcgcctggtcgagcgtcttggccacggtcgccgggacggcgaccaccttcagcatggtcttgaggttggc base pairs
gctcgcgcggaccagctcgcagaaccggtgccagcggccctgccgctggtggaagtcgtaccagaactccaaccg 75976 to 76050
 BsePI BstNI EaeI DsaI BsmFI Eco57I CfrI
 BstOI MscI Bsh1285I MluNI
 MvaI BalI BsaOI EcoRII

 MvaI MboI Kzo9I BmyI Bsp143II
 Bst2UI DpnII DpnI Bsp1286I BssHII BstH2I
 BstNI TthHB8I PspOMI FriOI BstH2I Bsp143II NlaIII
caggccctcggcctcgatctgggcccggcgctcgcgcgcggccagcgcctcccgcaggcccgccatgacccgctc base pairs
gtccgggagccggagctagacccgggccgcgagcgcgcgccggtcgcggagggcgtccgggcggtactgggcgag 76051 to 76125
 BstOI TaqI Bsp143I Eco24I HaeII CfrI HaeII Hsp92II
 EcoO109I NdeII Bsp120I BanII BsePI EcoNI
 DraII Sau3AI SduI ApaI EaeI

 ErhI Alw26I Eco52I MaeIII
BstD102I Eco130I BstZI EclXI BsaOI
AccBSI BssHII BbvI BssHII TspRI BssT1I EaeI BsaI Bsh1285I
ggtggcctccgcgcgctgctgtttggcgcgcaccactgcgtccttggtctcggccgtgtcctgccgggtcacgaa base pairs
ccaccggaggcgcgcgacgacaaaccgcgcgtggtgacgcaggaaccagagccggcacaggacggcccagtgctt 76126 to 76200
BsrBI BsePI Bst71I BsePI XcmI EcoT14I Eco31I BsiEI Tsp45I
 HgaI CfrI BsmAI BstMCI
 StyI EagI XmaIII

 SunI AfaI FriOI HgiEI
 Pfl23II BmyI Bme18I
 SplI RsaI MboII SduI SinI
ggcgacatactcggcgtacgccgtgttcttcacggggctctggtccacgcgctccaacgccgccgcgcacgcgac base pairs
ccgctgtatgagccgcatgcggcacaagaagtgccccgagaccaggtgcgcgaggttgcggcggcgcgtgcgctg 76201 to 76275
 PspLI Bsp1286I
 BsiWI Eco24I Eco47I
 Csp6I BanII AvaII

 PspEI HphI HgiEI AccIII SinI AluI MspA1I
 BstPI MaeIII RsrII Kpn2I Bme18I XcmI Cfr42I
 DrdI HgaI BsmFI Eco91I PshAI CspI MroI BsiMI Eco47I KspI
cagcgcgtcctcgctgggacacggcagggtgaccccggtccggaccagctccgcggtggcctccgggtcattccg base pairs
gtcgcgcaggagcgaccctgtgccgtcccactggggccaggcctggtcgaggcgccaccggaggcccagtaaggc 76276 to 76350
 BstEII CpoI AvaII BsaWI HgiEI DsaI SstII
 EcoO65I SinI Eco47I BseAI AvaII NspBII SacII
 Tsp45I Bme18I BspEI Bsp13I BstDSI Sfr303I

 KspI SacII KspI SacII XmaIII EcoRII Bme18I Bst2UI BstH2I Bsp1286I
 NspBII Eco32I NspBII EaeI NotI BstMCI Bst2UI BstNI SduI Alw44I Bbv12I
 BstDSI Cfr42I BstDSI Cfr42I EagI Bsh1285I SinI EcoRII BmyI VneI BmyI BsiHKAI
ggccgcggatatctgctccgcggcggccgccaggtccaggggcacgccgccgagcgcccggtgcacgtcggcccg base pairs
ccggcgcctatagacgaggcgccgccggcggtccaggtccccgtgcggcggctcgcgggccacgtgcagccgggc 76351 to 76425
 DsaI SstII DsaI SstII CfrI Eco52I BstNI HgiEI BstOI HaeII ApaLI MaeII
 MspA1I EcoRV MspA1I BstZI EclXI BsaOI MvaI Eco47I Bsp1286I SduI Alw21I
 Sfr303I Sfr303I CciNI BsiEI BstOI AvaII MvaI Bsp143II AspHI

 Hin1I EcoRII NdeII BsiEI BsaOI SduI Eco24I BsiHKAI AfaI
 BstF5I AcyI MvaI Bsp143I PvuI AluI BmyI FriOI MaeII Csp6I Cfr10I
 BbiII HgaI DpnII DpnI Ple19I EcoICRI Psp124BI NlaIII MaeII BsrFI
gatggcgtccaggcgatcgcggagctccacgtagtcggcgtagccatgttggaagaacggcacgtaccggcgcag base pairs
ctaccgcaggtccgctagcgcctcgaggtgcatcagccgcatcggtacaaccttcttgccgtgcatggccgcgtc 76426 to 76500
 BglI Hsp92I BstOI Sau3AI SgfI Ecl136II Bbv12I Alw21I MboII BsaAI Bse118I
 FokI BsaHI Bst2UI Kzo9I Bsh1285I AspHI SacI SstI Hsp92II RsaI
 Msp17I BstNI MboI BspCI BstMCI Bsp1286I BanII BsaAI BssAI

 MvaI KasI Hsp92I HaeII BssSI MvaI
 BmyI BstOI AccB1I NarI BbeI BsiI BstOI
 SduI NlaIII EcoRII BanI BbiII EheI DsaI EcoRII
gccgggcacgctcgtcatgtcgtccgccaggcgccccacggcctcgtggtagtcgataaacccgtcgcccgcctg base pairs
cggcccgtgcgagcagtacagcaggcggtccgcggggtgccggagcaccatcagctatttgggcagcgggcggac 76501 to 76575
 Bsp1286I Hsp92II BstNI BshNI BsaHI BstH2I TthHB8I BstNI
 Bst2UI Hin1I AcyI BstDSI TaqI Bst2UI
 Eco64I Msp17I Bsp143II

 MvaI FriOI Bst71I MvaI FriOI BbiII AccI BspLU11I
 BstOI BmyI AviII BstOI BmyI Msp17I TthHB8I NlaIII
 EcoRII SduI FspI BbvI EcoRII SduI Hin1I SalI HindII
ggccatttccaggagcccctccgcgatgcgcagcagccgcgccaggggctcggcgtcgacccgaaacatgtcggc base pairs
ccggtaaaggtcctcggggaggcgctacgcgtcgtcggcgcggtccccgagccgcagctgggctttgtacagccg 76576 to 76650
 BstNI Bsp1286I SfaNI BstNI Bsp1286I AcyI HincII NspI
 Bst2UI Eco24I Acc16I Bst2UI Eco24I BsaHI TaqI AflIII
 BanII BanII Hsp92I HgaI Hsp92II


 BstD102I
 GsuI BstF5I AccBSI NlaIII
gtaggtttcggcggcggcgtggaacgccgcgctccagccgaggcggtggatggcggcgagcggggggagcatggg base pairs
catccaaagccgccgccgcaccttgcggcgcgaggtcggctccgccacctaccgccgctcgcccccctcgtaccc 76651 to 76725
 BpmI FokI BsrBI Hsp92II


 Eco88I MvaI PspEI HphI
 BstH2I BsoBI Tru1I BstOI BstPI MaeIII
 Bsp143II Tru9I EcoRII Eco91I
gtggcgctggttctcgggggtgtaggggttaaacgcgaaggccgtatccagggcgagggtgaccgcctcggcgtt base pairs
caccgcgaccaagagcccccacatccccaatttgcgcttccggcataggtcccgctcccactggcggagccgcaa 76726 to 76800
 HaeII Ama87I MseI BstNI BstEII
 BcoI Bst2UI EcoO65I
 AvaI Tsp45I

 Eco88I SfcI
 BstH2I BsePI PspAI PspALI BmyI HaeII
EaeI Bsp143II Ama87I SmaI SduI Bsp143II
ggccgcgagcgcctgctcggcgcgcttgcggaagtcccgggggttgtagccgtgcgtgcccgccagcgcctgcag base pairs
ccggcgctcgcggacgagccgcgcgaacgccttcagggcccccaacatcggcacgcacgggcggtcgcggacgtc 76801 to 76875
CfrI HaeII BssHII BcoI XmaI Bsp1286I AlwNI
 BglI Cfr9I BsmFI BstH2I
 AvaI BsoBI BstSFI

SbfI TaqI AtsI HgaI Eco47I TaqI BsaHI BsrFI
PstI Bst71I MaeII AspI HgiEI BstH2I Msp17I BssAI
 AluI Tth111I TthHB8I Bme18I Bsp143II Hsp92I NgoMI
gcggcgcagctcgaccacgtcgaactcggcgcggttctcgacgcggtccagcgccgcctcgacgccggcggccca base pairs
cgccgcgtcgagctggtgcagcttgagccgcgccaagagctgcgccaggtcgcggcggagctgcggccgccgggt 76876 to 76950
Sse8387I BbvI AspI TaqI Tth111I HaeII Hin1I SgrAI Bse118I
 TthHB8I TthHB8I AtsI AvaII TthHB8I AcyI NgoAIV
 TaqI SinI BbiII MroNI Cfr1

 Aor51HI Bst71I AvaI BsePI
HgaI AfeI BbvI Eco88I BssHII
 NaeI Bsp143II Ama87I SmaI MboII
gcgctcgctgctgccccgggcgcgctgggccgccatcttcgccgtcaggtcggcgacggcggcctcaagttcgtc base pairs
cgcgagcgacgacggggcccgcgcgacccggcggtagaagcggcagtccagccgctgccgccggagttcaagcag 76951 to 77025
 BstH2I BcoI XmaI
 Eco47III PspAI BsoBI
 0I HaeII Cfr9I PspALI

 BsaHI AccB1I NarI HaeII PstI Eco47I XmaIII
 BbiII KasI BbiII EheI BcgI SfcI BssHII HgiEI EaeI
 Hin1I Eco64I BsaHI BbeI AluI Sse8387I NspBII SinI BsmFI
ggcgcggcgtcgcgtggcgccgatgaccttgcccagctcctgcagggcgcgcccgctgggggaatggtccccggc base pairs
ccgcgccgcagcgcaccgcggctactggaacgggtcgaggacgtcccgcgcgggcgacccccttaccaggggccg 77026 to 77100
 Msp17I BanI Msp17I Bsp143II AlwNI AscI MspA1I Bme18I EagI
 Hsp92I BshNI Hsp92I BstSFI BsePI AvaII BstZI
 AcyI HgaI Hin1I AcyI BstH2I SbfI CfrI

 BsaOI DraII BmyI BsoBI BsiEI
 Eco52I Bst71I SduI Eco88I BbvI BstMCI
 EclXI BsmFI BsgI BsiI Ama87I NspBII BsaOI
cgtcccttcggcgtgcagcaggcccccgaacccagcctcgtgccccgcgaggctttcccgagcagcggtcgtcgc base pairs
gcagggaagccgcacgtcgtccgggggcttgggtcggagcacggggcgctccgaaagggctcgtcgccagcagcg 77101 to 77175
 Bsh1285I BbvI BssSI BcoI MspA1I
 BstMCI EcoO109I Bsp1286I AvaI Bst71I
 BsiEI Bsh1285I

 KspI SacII BspXI ClaI Hsp92II Bst2UI Bst2UI
 NspBII TthHB8I Bsu15I SinI Eco47I BsePI BstOI BstNI
 BstDSI Cfr42I Bsa29I SfaNI AvaII AscI BstNI SexAI
gcgggccgcggcatcgatgagggcggcatggtccccctccggctgggcgcaggcccggcgcgcctggactaccag base pairs
cgcccggcgccgtagctactcccgccgtaccagggggaggccgacccgcgtccgggccgcgcggacctgatggtc 77176 to 77250
 DsaI SstII BspDI TaqI NlaIII BsmFI BssHII EcoRII
 MspA1I BanIII BscI Bme18I EcoRII BstOI
 Sfr303I Bsp106I BseCI HgiEI MvaI DrdI

 CciNI BstMCI BstOI Bsp1286I BanII TaqI MvaI BpmI
 BstZI Bsh1285I MvaI EcoICRI Eco24I BsiHKAI BstOI
 MvaI NotI BsiEI EcoRII Ecl136II SacI Alw21I EcoRII GsuI HinfI
gtcggcggccgccgaccccagggtcgtgagctcgtcgatggccccccgcgcctccagggccagccgagtcgcctt base pairs
cagccgccggcggctggggtcccagcactcgagcagctaccggggggcgcggaggtcccggtcggctcagcggaa 77251 to 77325
 EaeI XmaIII BstNI AluI BmyI FriOI TthHB8I BstNI PleI
 CfrI EclXI BsaOI DrdI SduI Bbv12I SstI Bst2UI
 EagI Eco52I Bst2UI AspHI Psp124BI BglI

 KspI SacII EagI Eco52I
 NspBII Bsp143II CfrI EclXI
 BstDSI Cfr42I CfrI EaeI XmaIII
tacataccccgcggcgctatcggccagcaccgcgaggaaggacaggggcgaggccgggtcgcgggcggccgcgcc base pairs
atgtatggggcgccgcgatagccggtcgtggcgctccttcctgtccccgctccggcccagcgcccgccggcgcgg 77326 to 77400
 DsaI SstII BstH2I BstZI Bsh1285I
 MspA1I HaeII CciNI BstMCI
 Sfr303I EaeI NotI BsiEI

 BstOI Bst2UI Hin1I AcyI NlaIII CfrI BsiEI KspI SacII
 BsaOI Eam1105I BstNI BshNI BsaHI BstH2I BstZI BstMCI DsaI SstII AhdI
 BstNI AhdI HgaI Eco64I Msp17I Bsp143II EagI Bsh1285I NspBII BsmFI
cagggccgacaccgcgtccgccagggcgccatgcgcccgcacggccgcgtccaccgtcgccgcgggacttgccgt base pairs
gtcccggctgtggcgcaggcggtcccgcggtacgcgggcgtgccggcgcaggtggcagcggcgccctgaacggca 77401 to 77475
 MvaI EclHKI EcoRII BanI BbiII EheI Hsp92II Eco52I BstDSI Cfr42I AspEI
 Bst2UI AspEI BstOI AccB1I NarI BbeI BglI XmaIII HgaI MspA1I EclHKI
 EcoRII MvaI KasI Hsp92I HaeII EaeI EclXI BsaOI Sfr303I Eam11

 Bsp143I
 NruI BstH2I MboI DpnI
 Bsp68I Bsp143II DpnII
cgcgacggcggcgctcccggcgttgatggcgtttgacacggctttggcgattgtgggggcgtgatcggaaaagaa base pairs
gcgctgccgccgcgagggccgcaactaccgcaaactgtgccgaaaccgctaacacccccgcactagccttttctt 77476 to 77550
 HaeII NdeII
 Sau3AI
 05I Kzo9I

 BssSI BsrFI BbiII HgaI Alw26I BsaHI MboII BssAI
 SinI BssAI Hin1I Ama87I BsmBI Hsp92I BbsI SfaNI
 BsgI AvaII PshAI Hsp92I BsoBI Msp17I BpuAI BstF5I
ctgcacgaggaccggcgtctcgggggcgtcggcgaacagggtcttcagcaccaccacgaaggcgggatgcaggcc base pairs
gacgtgctcctggccgcagagcccccgcagccgcttgtcccagaagtcgtggtggtgcttccgccctacgtccgg 77551 to 77625
 BsiI Eco47I Msp17I BcoI BsmAI BbiII Bbv16II FokI BsrFI
[truncated: 860,999 more chars]
