# Supplementary material for: Identification of restriction endonuclease with potential ability to cleave the HSV-2 genome: Inherent potential for biosynthetic versus live recombinant microbicides
Source: Theor Biol Med Model. 2008 Aug 7;5:18. doi: 10.1186/1742-4682-5-18 (PMC2526989; doi:10.1186/1742-4682-5-18)
Supplement: Additional File 2 — Protparam physicochemical characterization of EcoRII. The data provided represents the protein parameter prediction on the REase EcoRII computed using the protparam software. [file 1742-4682-5-18-S2.doc]

| [**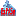ExPASy Home page**](http://www.expasy.ch/) | [**Site Map**](http://www.expasy.ch/sitemap.html) | [**Search ExPASy**](http://www.expasy.ch/ExpasyHunt/) | [**Contact us**](http://www.expasy.ch/contact.html) | [**Proteomics tools**](http://www.expasy.ch/tools/) | [**Swiss-Prot**](http://www.expasy.ch/sprot/) |
| --- | --- | --- | --- | --- | --- |

| Top of Form  Search for  Bottom of Form |
| --- |


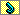
**The ExPASy Server requires Javascript to be fully functional. You may not see all the information available for this page** [**(More information)**](http://www.expasy.ch/javascript.html)**.**

**ProtParam**

[**T2E2_ECOLX**](http://www.expasy.ch/uniprot/P14633) **(P14633)**

DE Type II restriction enzyme EcoRII (EC 3.1.21.4) (Endonuclease EcoRII)
DE (R.EcoRII).

**The parameters have been computed for the following feature:**

FT CHAIN 1 404 Type II restriction enzyme EcoRII.

The computation has been carried out on the complete sequence (**404** amino acids).

Warning: All computation results shown below do **not** take into account any annotated post-translational modification.

[References](http://www.expasy.ch/tools/protpar-ref.html) and [documentation](http://www.expasy.ch/tools/protparam-doc.html) are available.


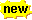
Please note the [modified algorithm for extinction coefficient](http://www.expasy.ch/tools/protparam-doc.html).

**Number of amino acids:** 404

**Molecular weight:** 45611.0

**Theoretical pI:** 6.10

Top of Form

**Amino acid composition:** 
Ala (A) 32 7.9%

Arg (R) 24 5.9%

Asn (N) 15 3.7%

Asp (D) 19 4.7%

Cys (C) 5 1.2%

Gln (Q) 12 3.0%

Glu (E) 33 8.2%

Gly (G) 26 6.4%

His (H) 19 4.7%

Ile (I) 28 6.9%

Leu (L) 45 11.1%

Lys (K) 19 4.7%

Met (M) 5 1.2%

Phe (F) 18 4.5%

Pro (P) 16 4.0%

Ser (S) 26 6.4%

Thr (T) 18 4.5%

Trp (W) 5 1.2%

Tyr (Y) 13 3.2%

Val (V) 26 6.4%

Pyl (O) 0 0.0%

Sec (U) 0 0.0%

(B) 0 0.0%

(Z) 0 0.0%

(X) 0 0.0%

Bottom of Form

**Total number of negatively charged residues (Asp + Glu):** 52

**Total number of positively charged residues (Arg + Lys):** 43

**Atomic composition:**

Carbon C 2053

Hydrogen H 3205

Nitrogen N 565

Oxygen O 593

Sulfur S 10

**Formula:** C_2053_H_3205_N_565_O_593_S_10_

**Total number of atoms:** 6426

**Extinction coefficients:**

Extinction coefficients are in units of M^-1^ cm^-1^, at 280 nm measured in water.

Ext. coefficient 47120

Abs 0.1% (=1 g/l) 1.033, assuming ALL Cys residues appear as half cystines

Ext. coefficient 46870

Abs 0.1% (=1 g/l) 1.028, assuming NO Cys residues appear as half cystines

**Estimated half-life:**

The N-terminal of the sequence considered is M (Met).

The estimated half-life is: 30 hours (mammalian reticulocytes, in vitro).

>20 hours (yeast, in vivo).

>10 hours (Escherichia coli, in vivo).

**Instability index:**

The instability index (II) is computed to be 45.04

This classifies the protein as unstable.

**Aliphatic index:** 97.05

**Grand average of hydropathicity (GRAVY):** -0.183

| [**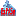ExPASy Home page**](http://www.expasy.ch/) | [**Site Map**](http://www.expasy.ch/sitemap.html) | [**Search ExPASy**](http://www.expasy.ch/ExpasyHunt/) | [**Contact us**](http://www.expasy.ch/contact.html) | [**Proteomics tools**](http://www.expasy.ch/tools/) | [**Swiss-Prot**](http://www.expasy.ch/sprot/) |
| --- | --- | --- | --- | --- | --- |
